# Supplementary material for: Challenges in Defining a Reference Set of Differentially Expressed lncRNAs in Ulcerative Colitis by Meta-Analysis
Source: Curr Issues Mol Biol. 2024 Apr 5;46(4):3164–74. doi: 10.3390/cimb46040198 (PMC11049510; doi:10.3390/cimb46040198)
Supplement: Supplementary file 1 [file cimb-46-00198-s001.zip › Supplementary files/Figure S1.pdf]

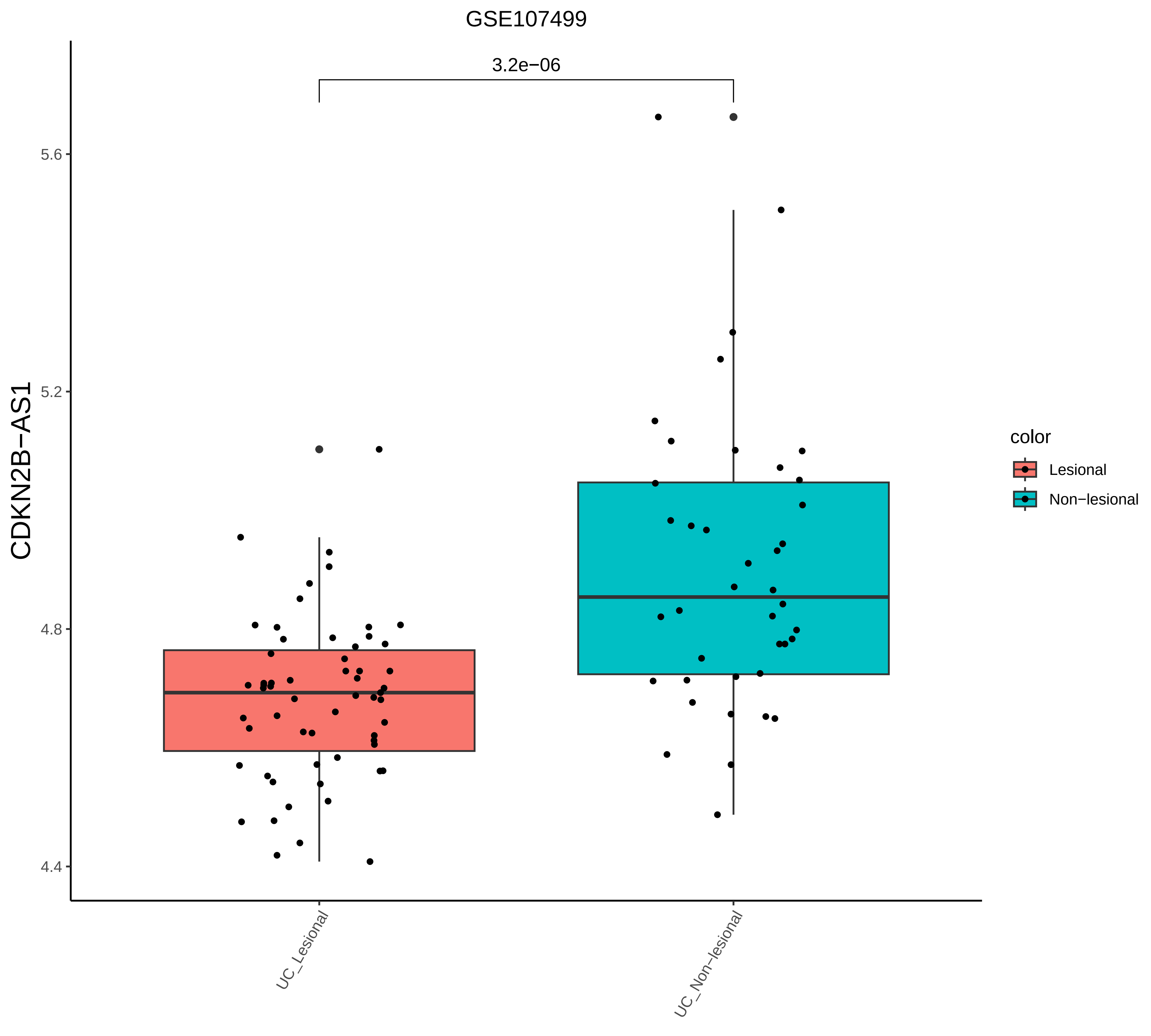

GSE107499
3.2e−06
CDKN2B−AS1
5.6
5.2
4.8
4.4
UC_Lesional
UC_Non−lesional
color
Lesional
Non−lesional

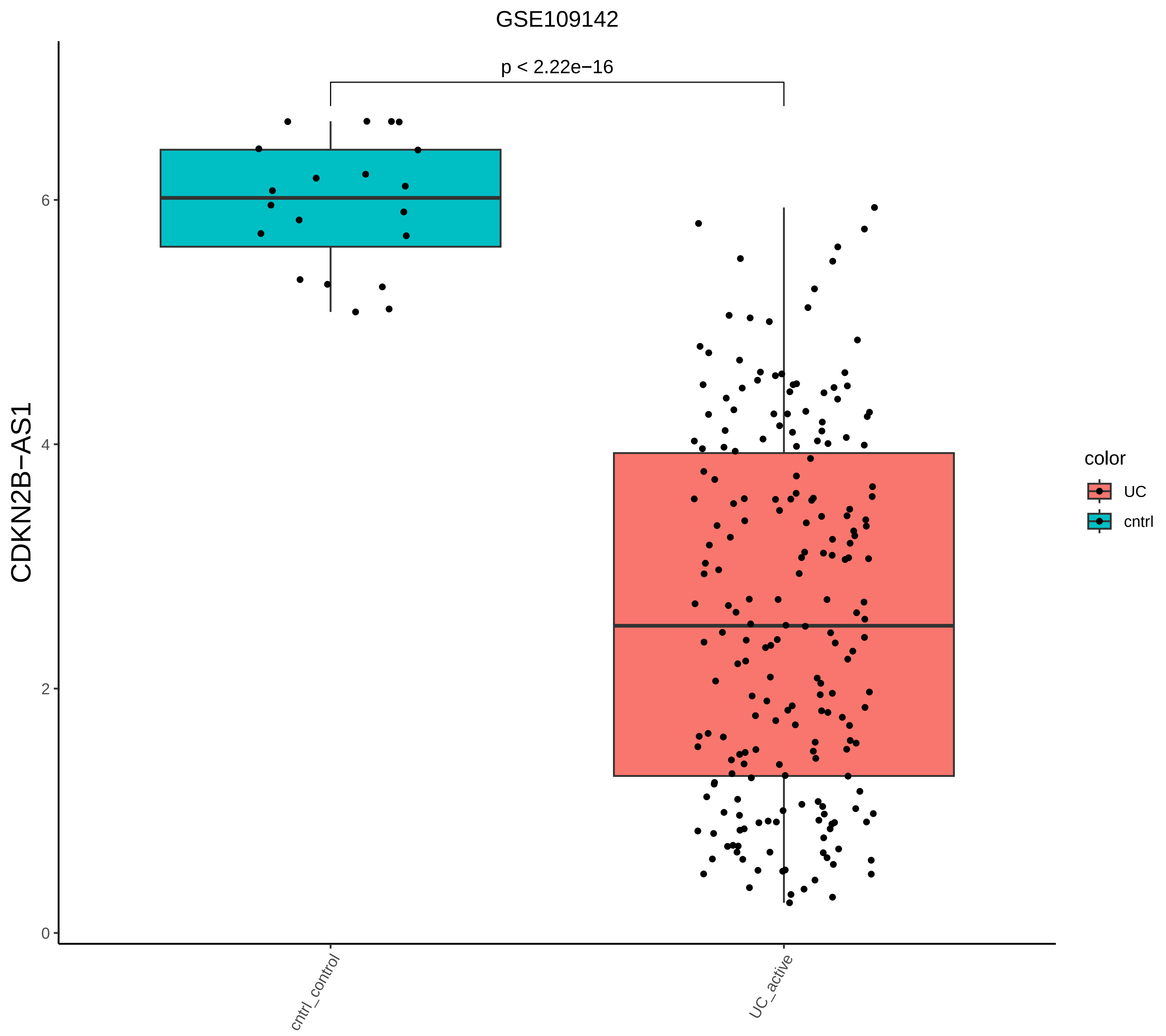
GSE109142
p < 2.22e−16
CDKN2B−AS1
0
2
4
6
cntrl_control
UC_active
color
UC
cntrl

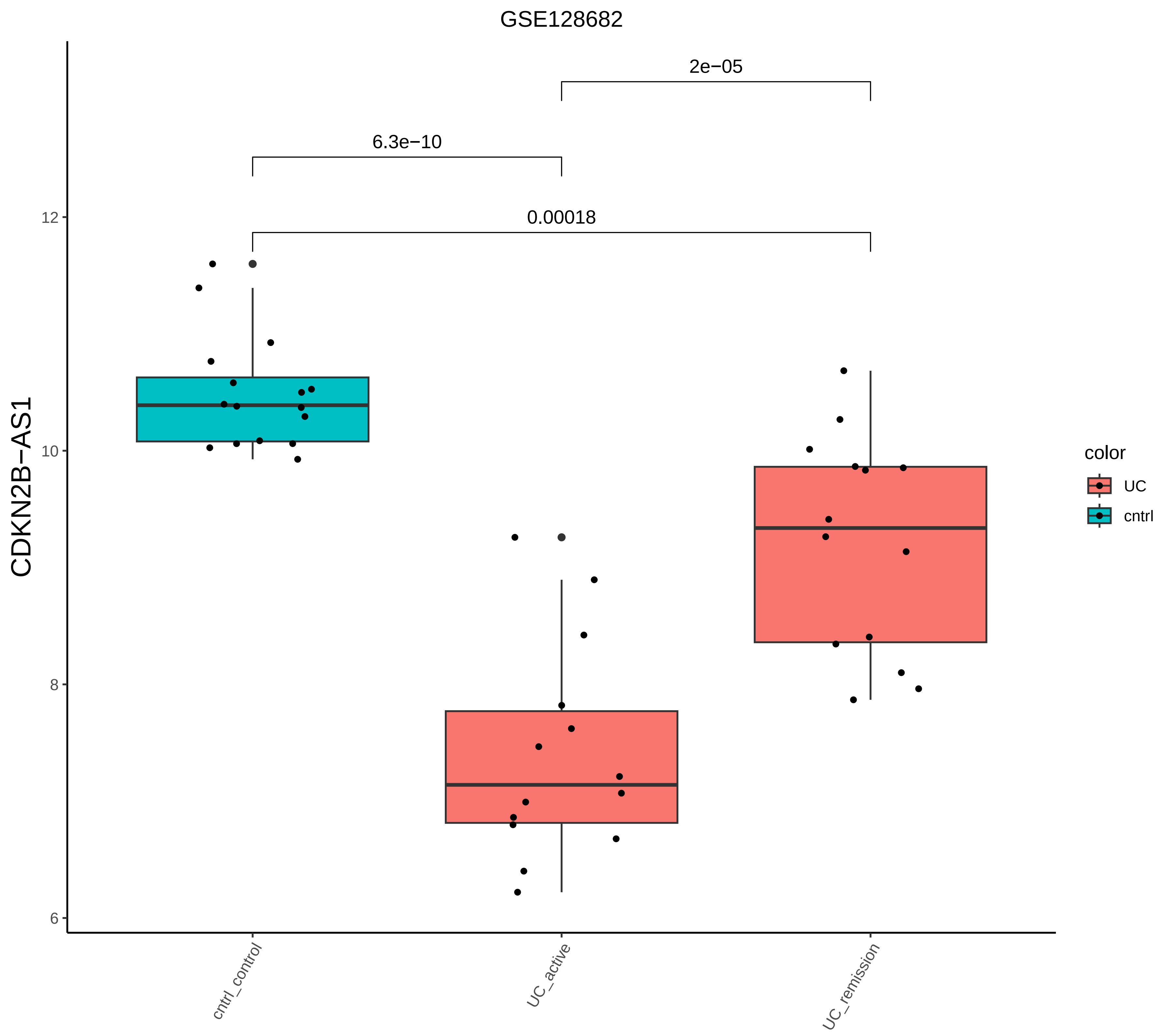
GSE128682
2e−05
6.3e−10
0.00018
CDKN2B−AS1
12
10
8
6
cntrl_control
UC_active
UC_remission
color
UC
cntrl

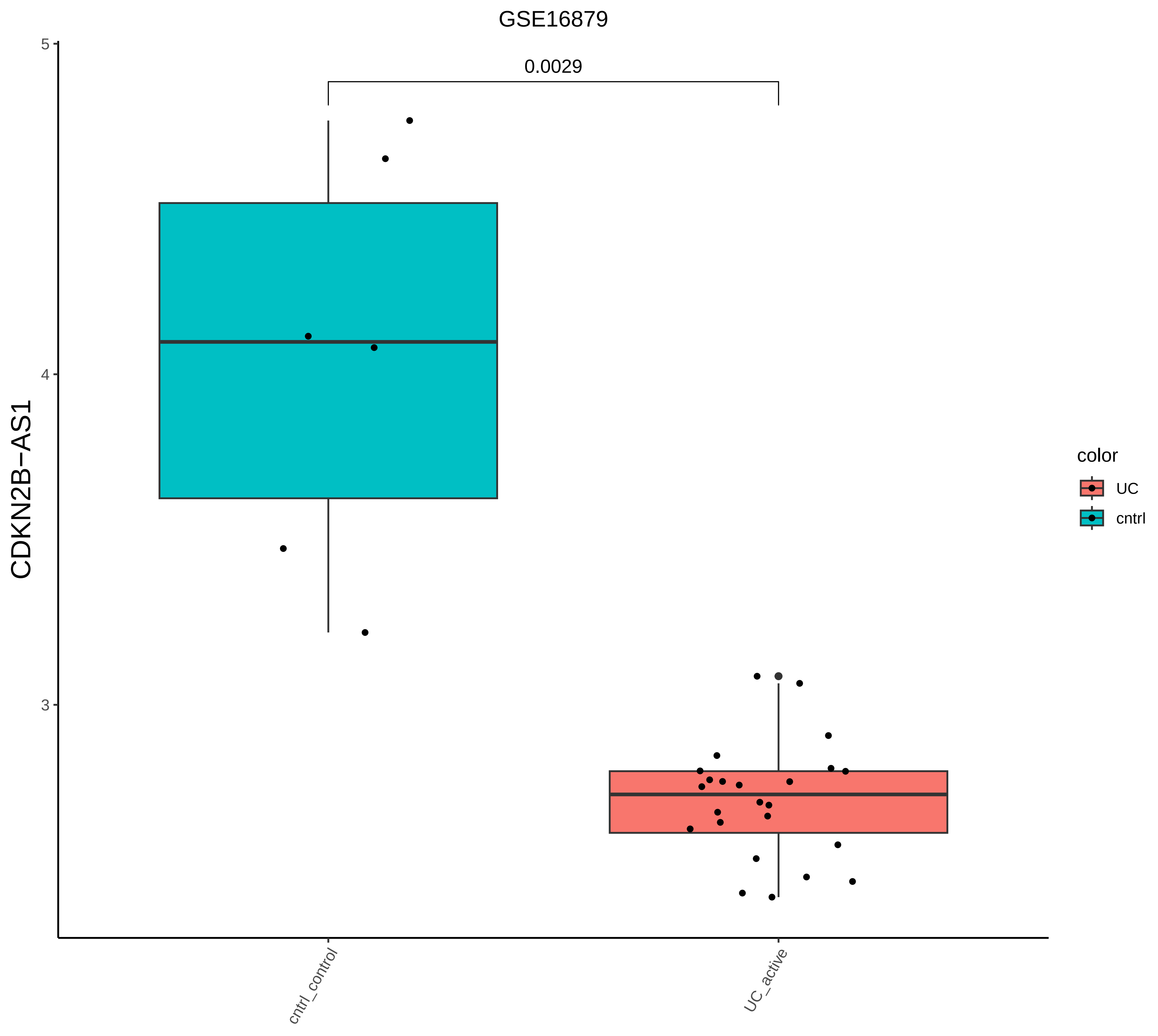

GSE16879
0.0029
CDKN2B−AS1
5
4
3
cntrl_control
UC_active
color
UC
cntrl

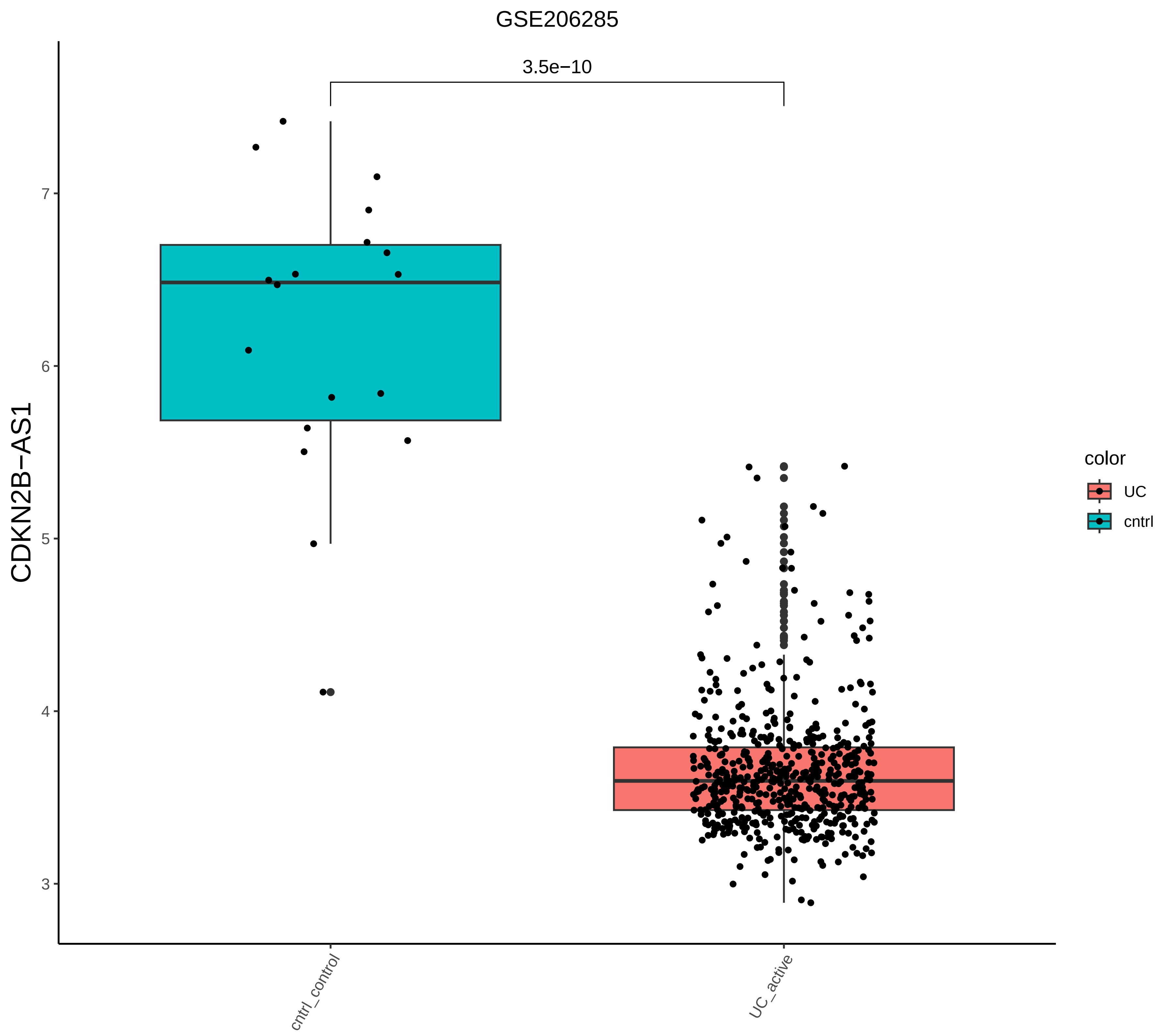
GSE206285
3.5e−10
CDKN2B−AS1
7
6
5
4
3
cntrl_control
UC_active
color
UC
cntrl

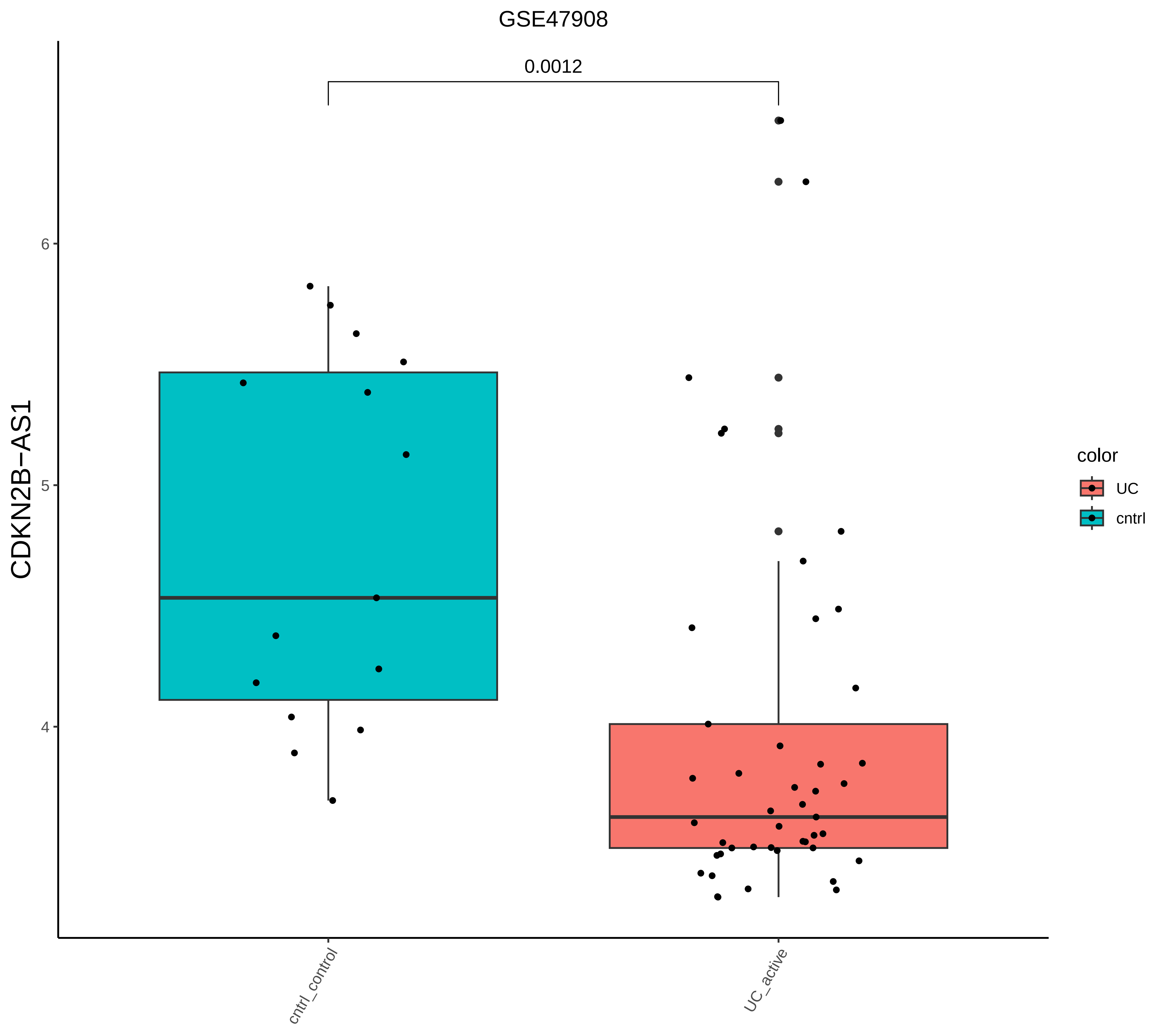

GSE47908
0.0012
CDKN2B−AS1
6
5
4
cntrl_control
UC_active
color
UC
cntrl

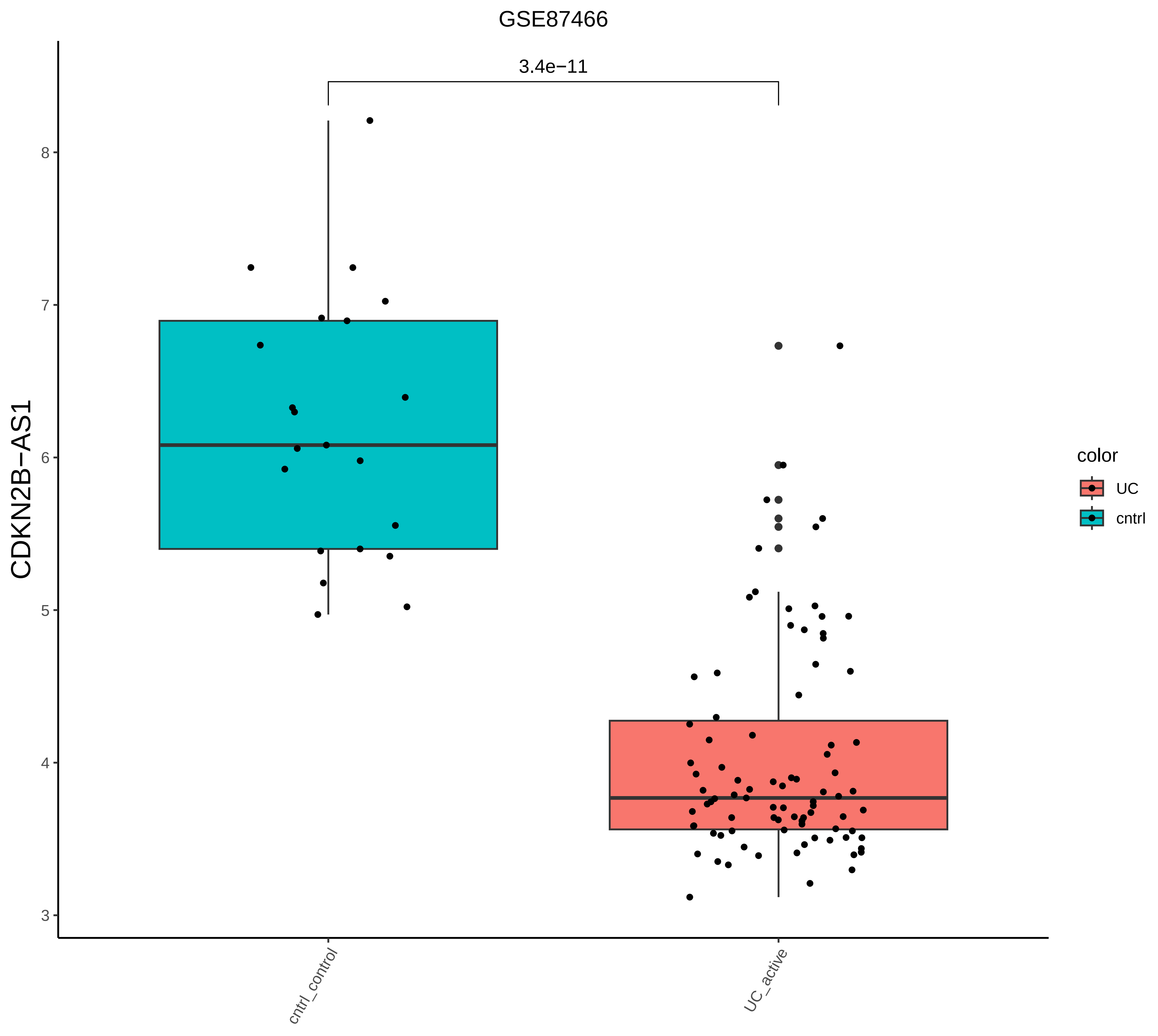
GSE87466
3.4e−11
CDKN2B−AS1
8
7
6
5
4
3
cntrl_control
UC_active
color
UC
cntrl

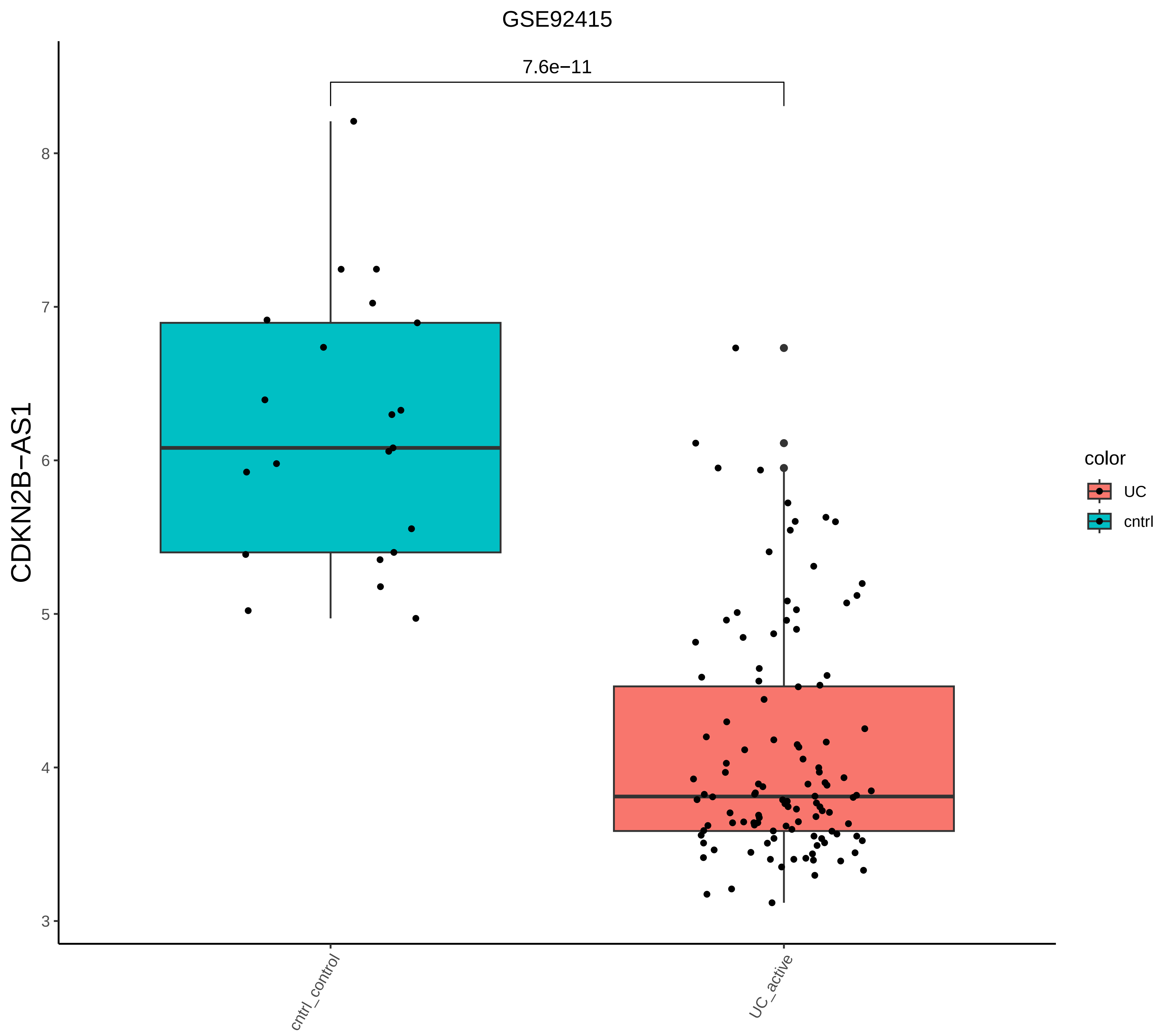

GSE92415
7.6e−11
CDKN2B−AS1
8
7
6
5
4
3
cntrl_control
UC_active
color
UC
cntrl

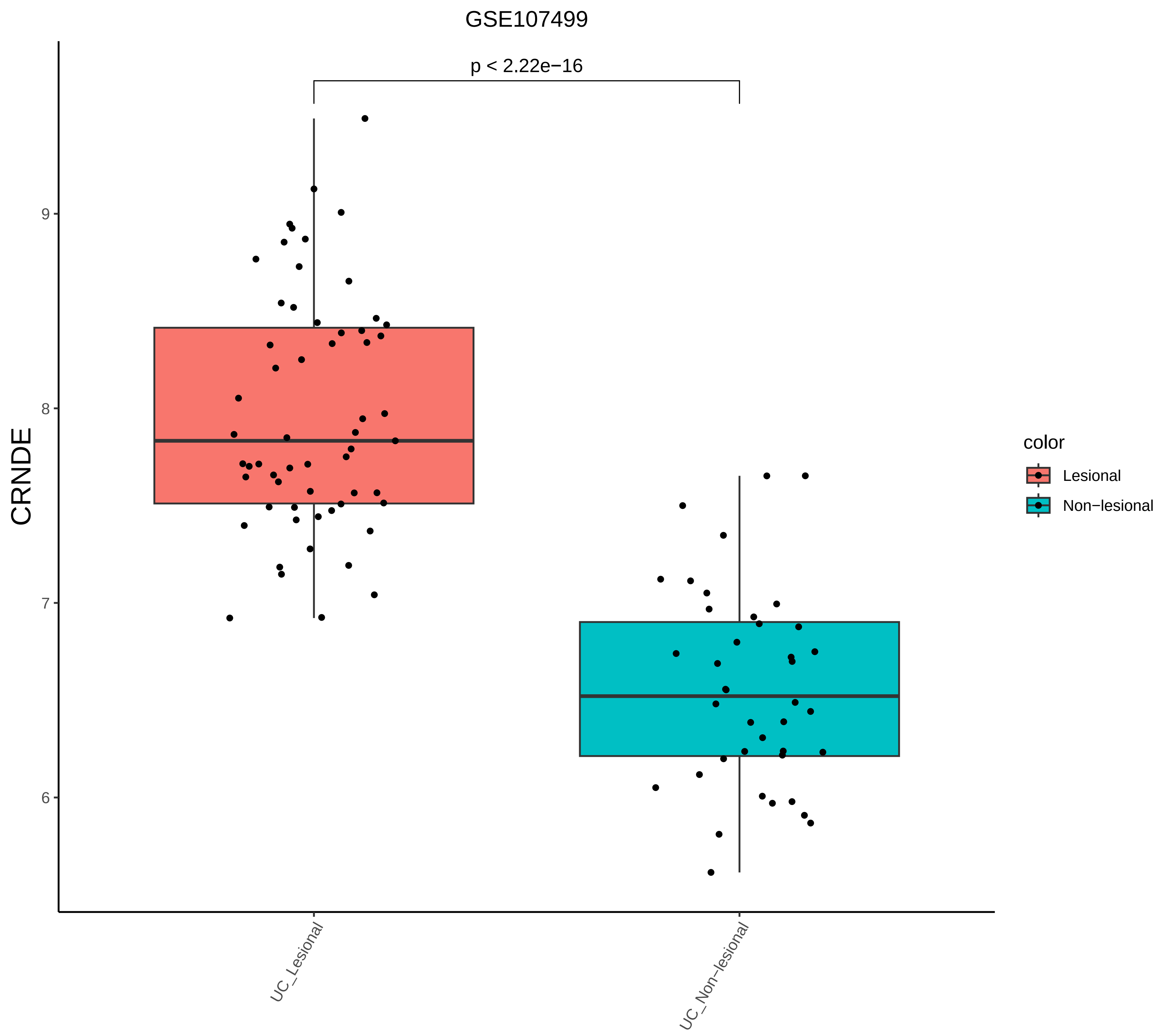
GSE107499
p < 2.22e−16
CRNDE
9
8
7
6
UC_Lesional
UC_Non−lesional
color
Lesional
Non−lesional

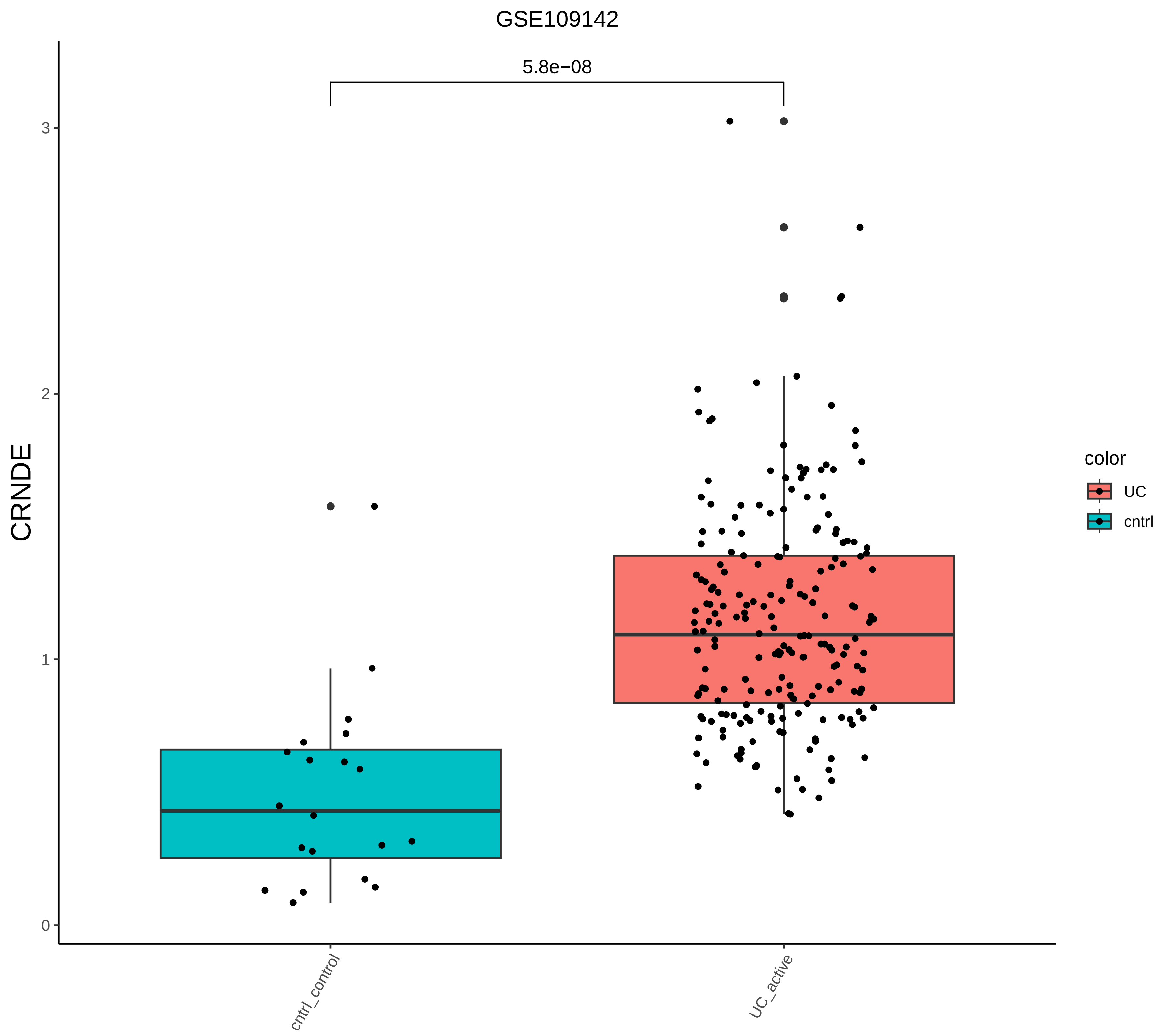
GSE109142
5.8e−08
CRNDE
3
2
1
0
cntrl_control
UC_active
color
UC
cntrl

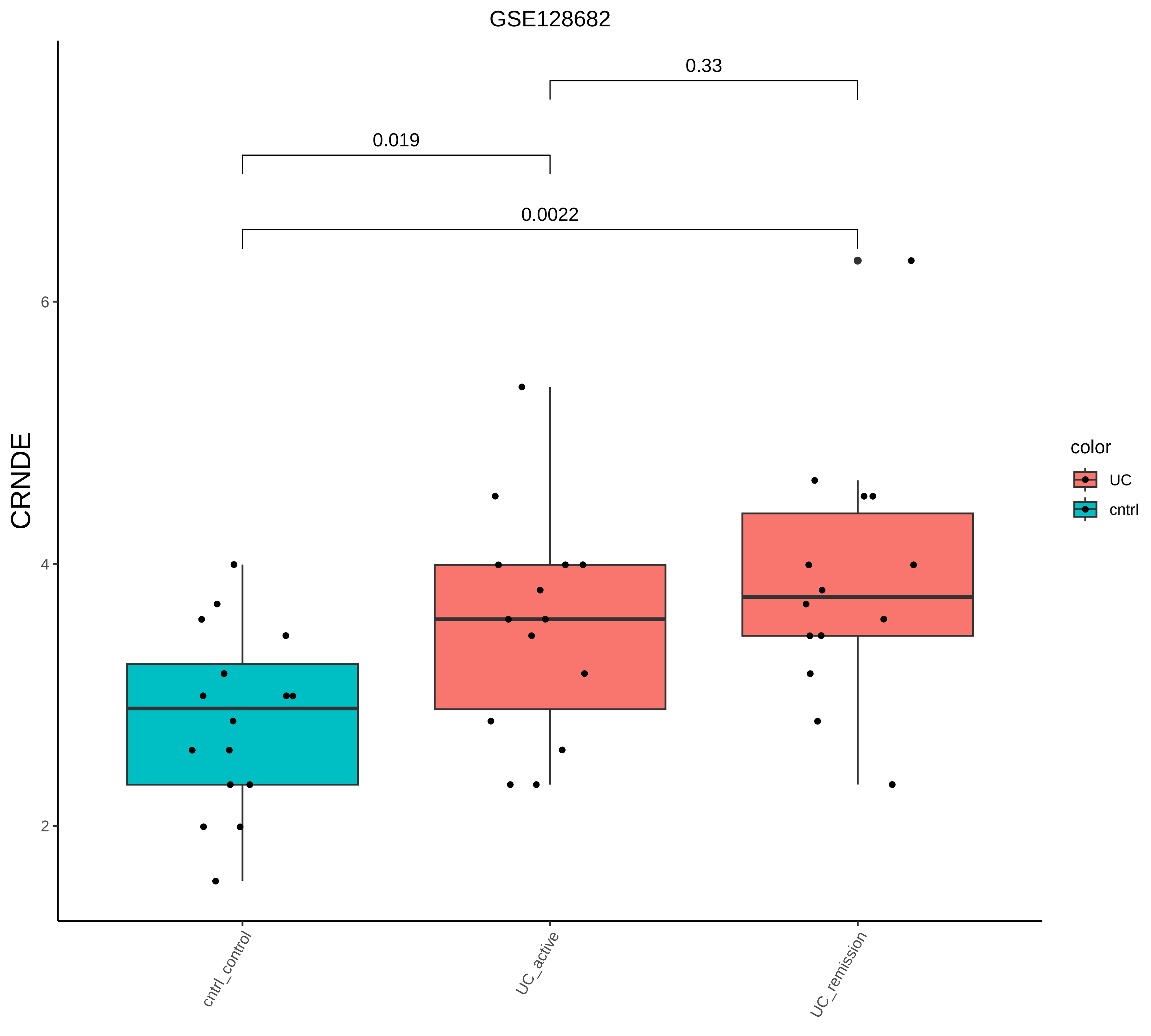
GSE128682
0.33
0.019
0.0022
CRNDE
6
4
2
color
UC
cntrl
cntrl_control
UC_active
UC_remission

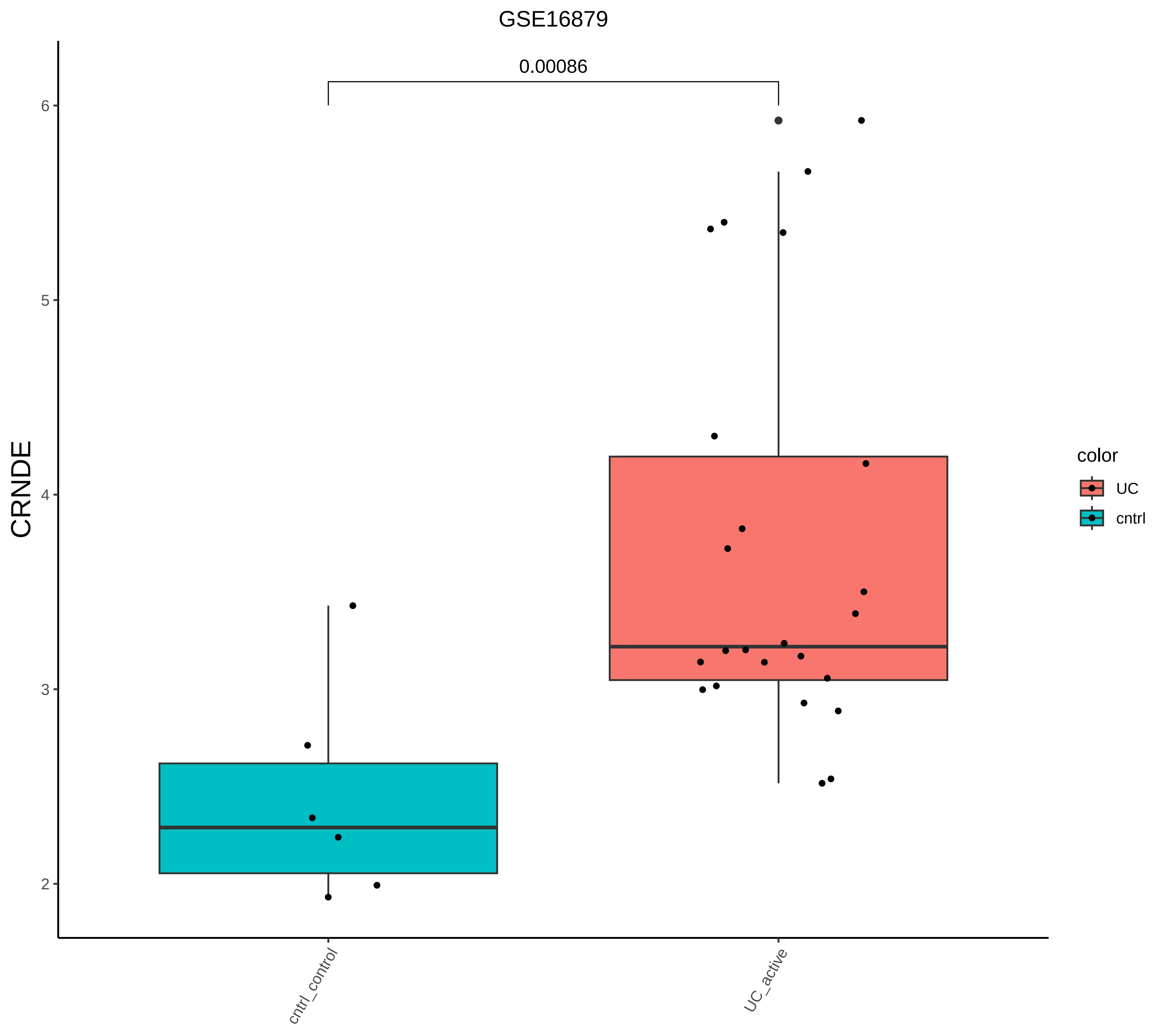
GSE16879
0.00086
CRNDE
6
5
4
3
2
cntrl_control
UC_active
color
UC
cntrl

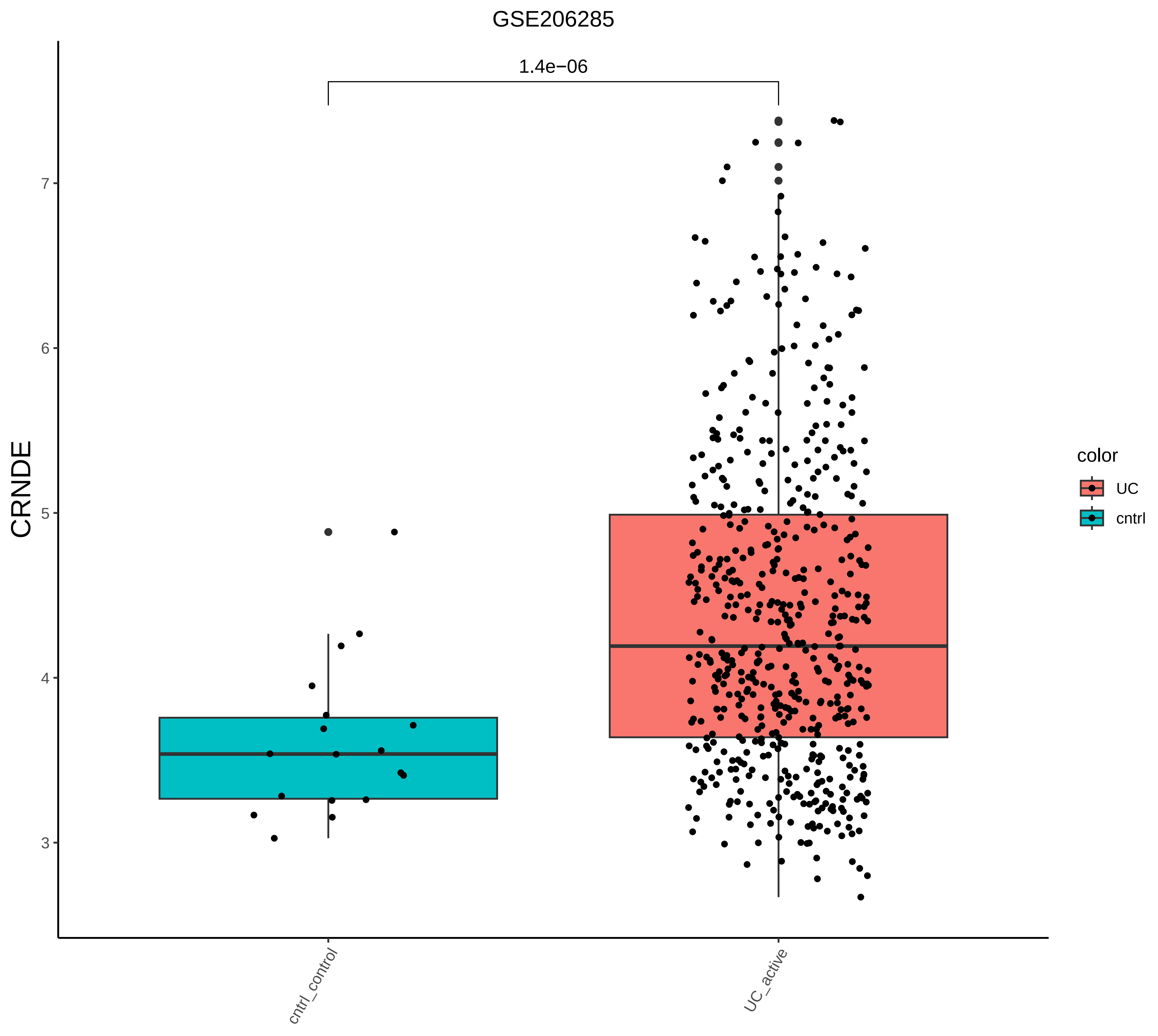

GSE206285
1.4e−06
CRNDE
7
6
5
4
3
cntrl_control
UC_active
color
UC
cntrl

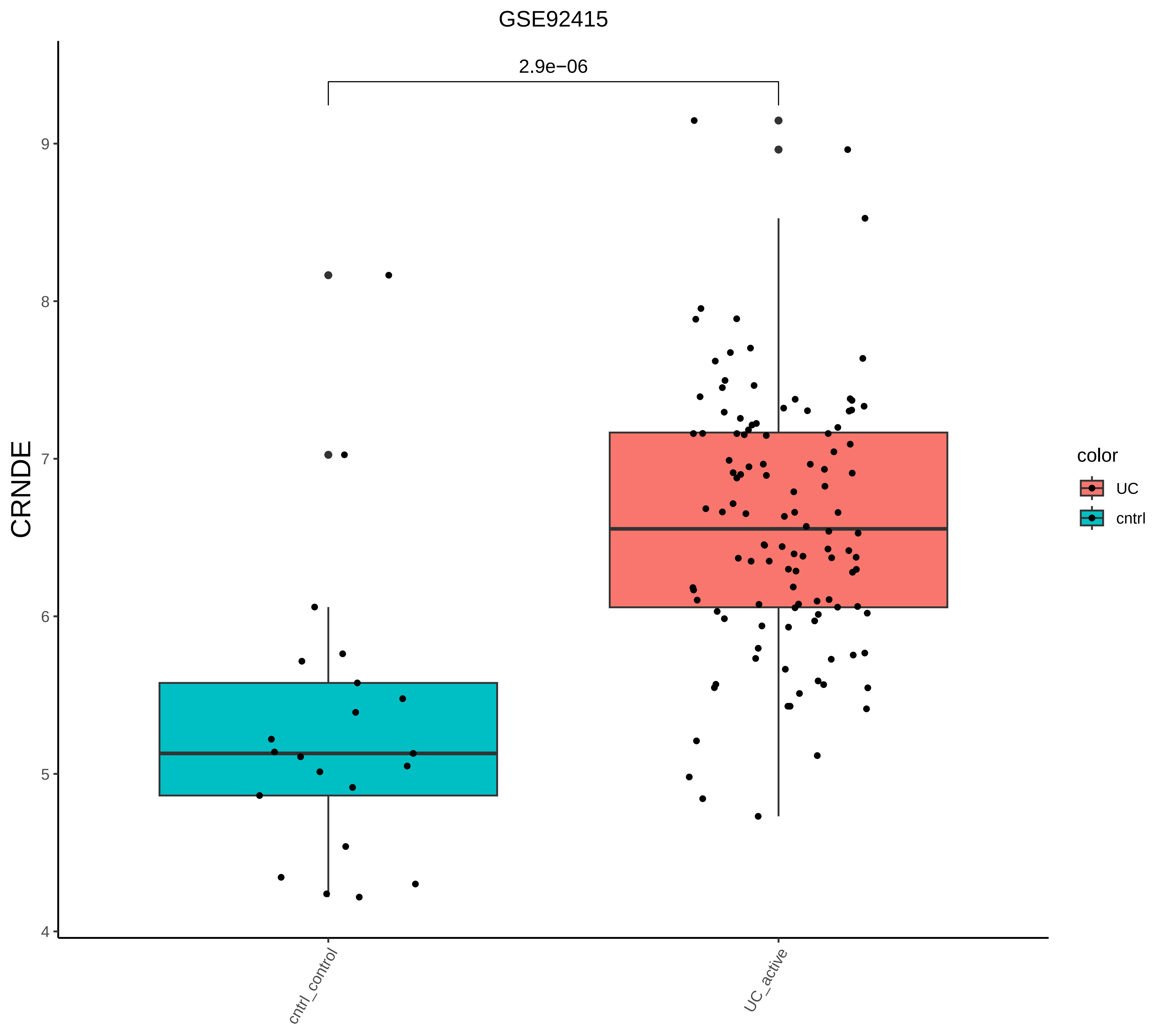
GSE92415
2.9e−06
CRNDE
9
8
7
6
5
4
cntrl_control
UC_active
color
UC
cntrl

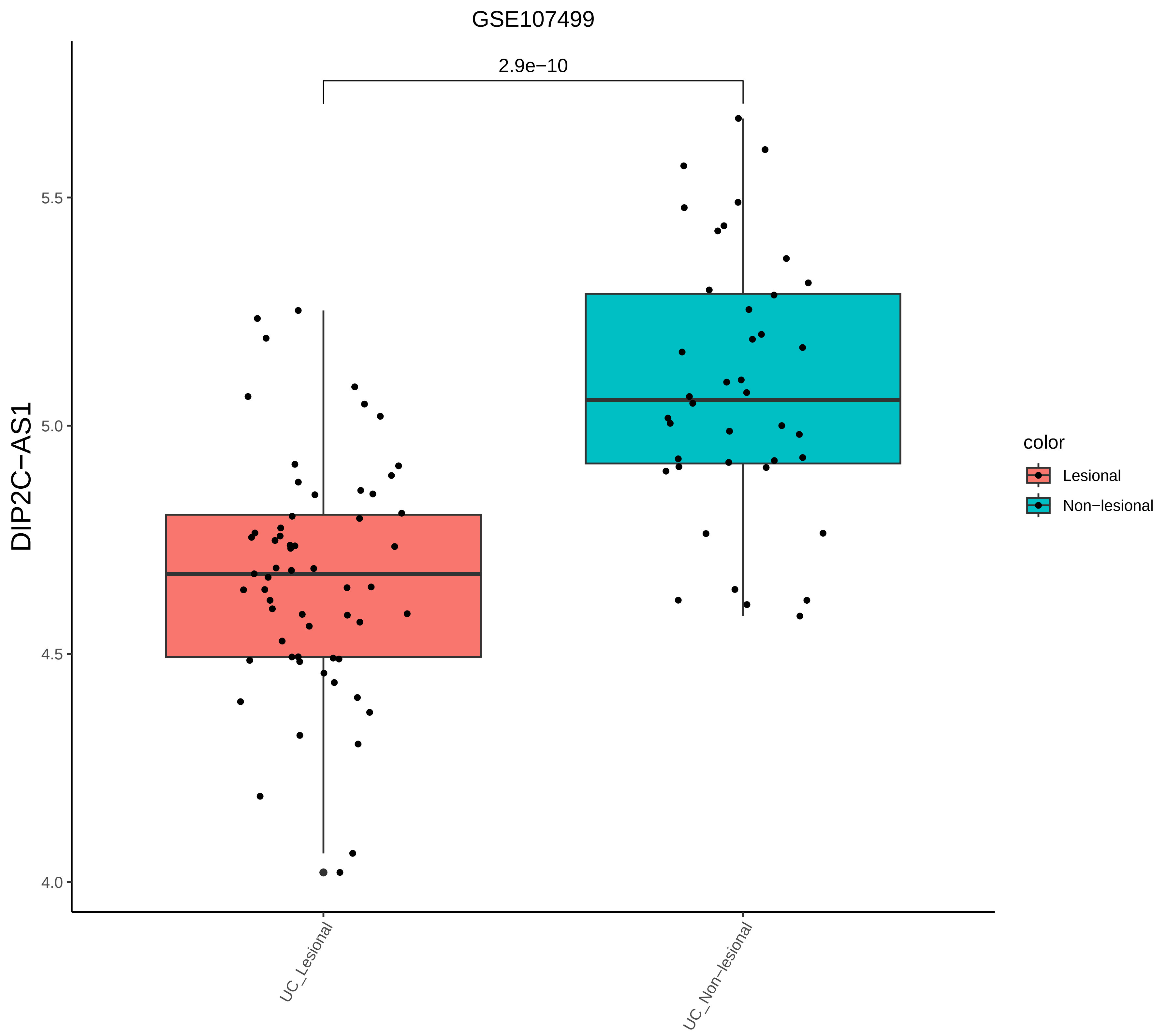
GSE107499
2.9e−10
DIP2C−AS1
5.5
5.0
4.5
4.0
UC_Lesional
UC_Non−lesional
color
Lesional
Non−lesional

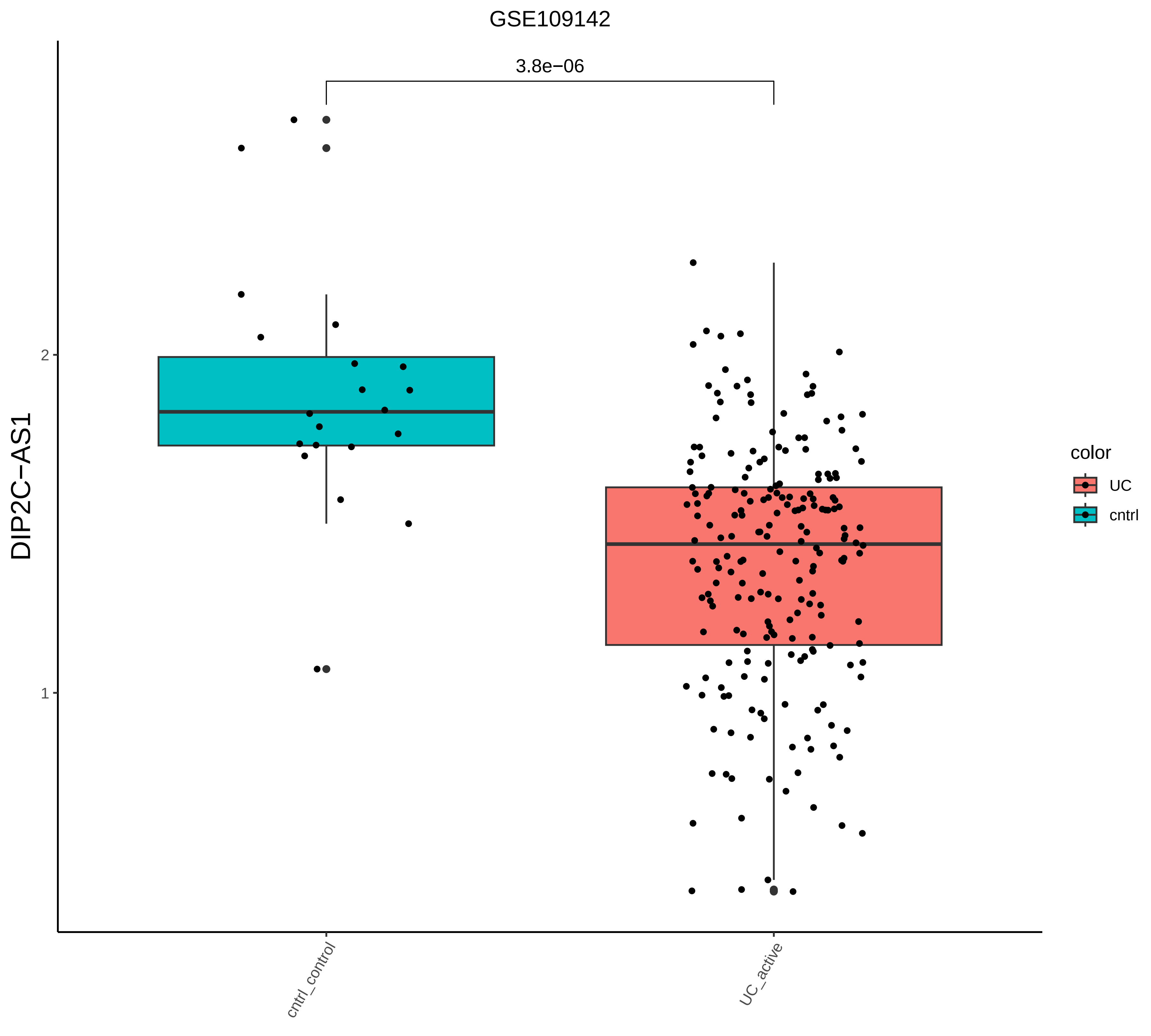

GSE109142
3.8e−06
DIP2C−AS1
2
1
cntrl_control
UC_active
color
UC
cntrl

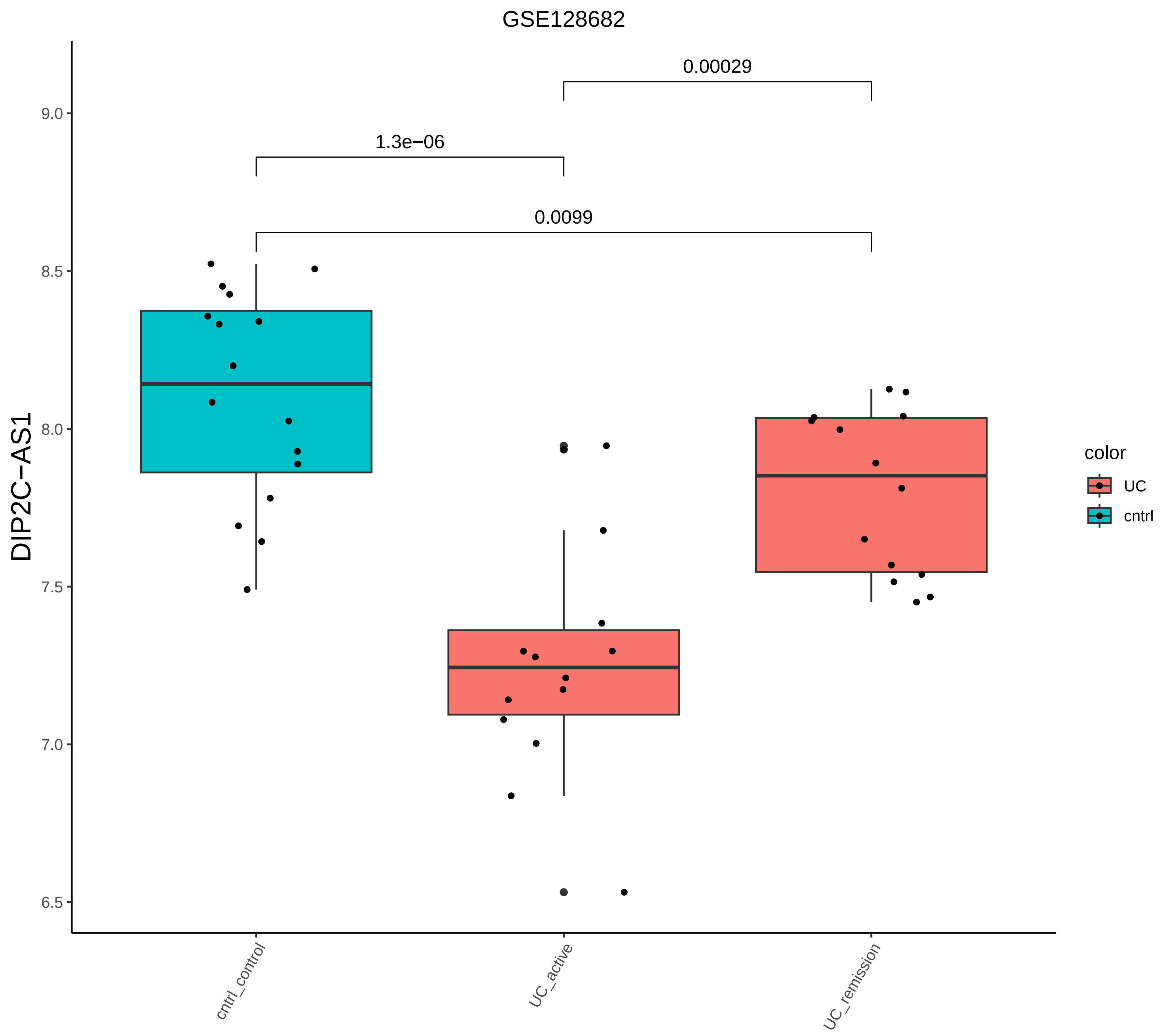
GSE128682
0.00029
1.3e−06
0.0099
DIP2C−AS1
9.0
8.5
8.0
7.5
7.0
6.5
cntrl_control
UC_active
UC_remission
color
UC
cntrl

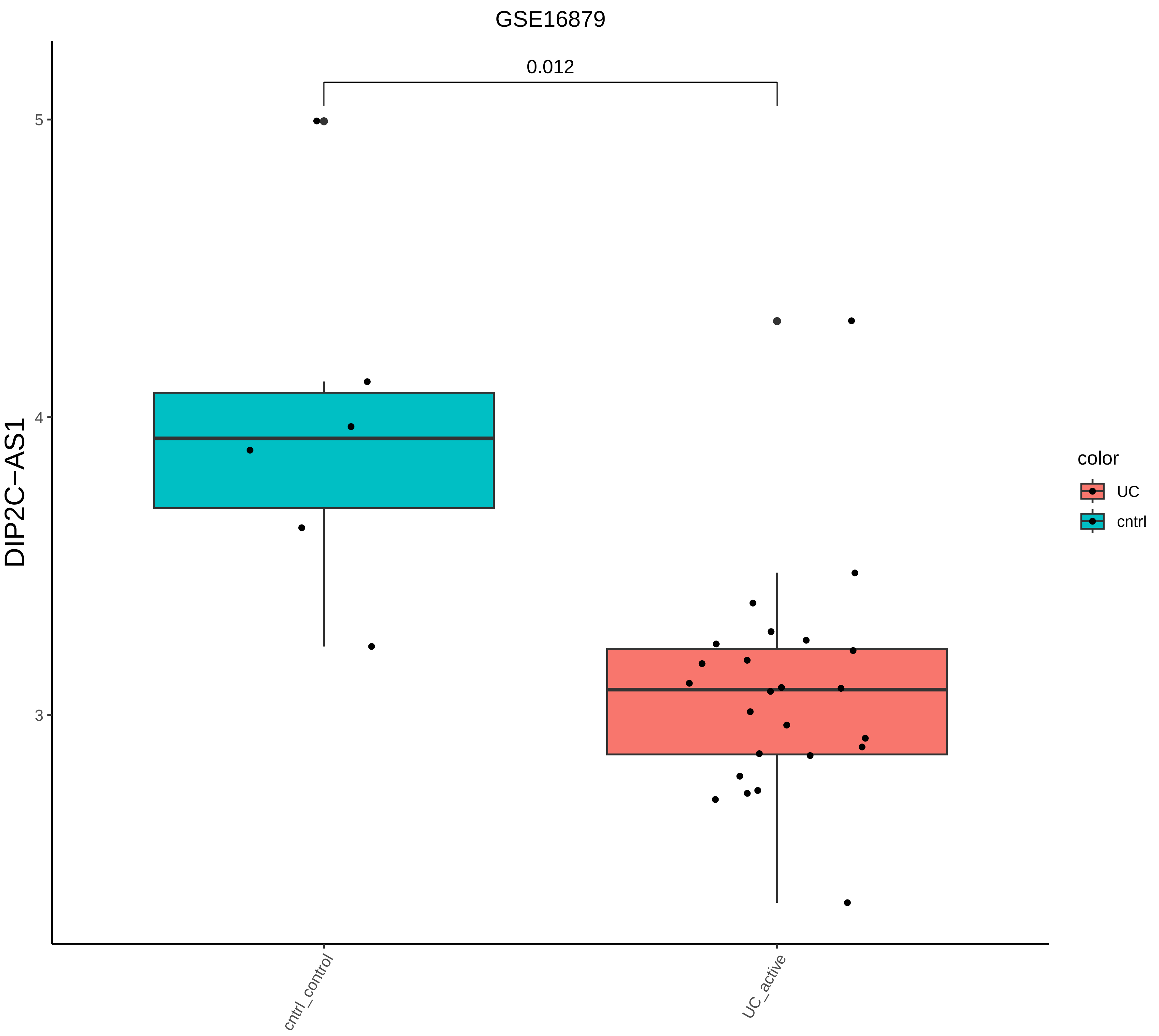

GSE16879
0.012
DIP2C−AS1
5
4
3
cntrl_control
UC_active
color
UC
cntrl

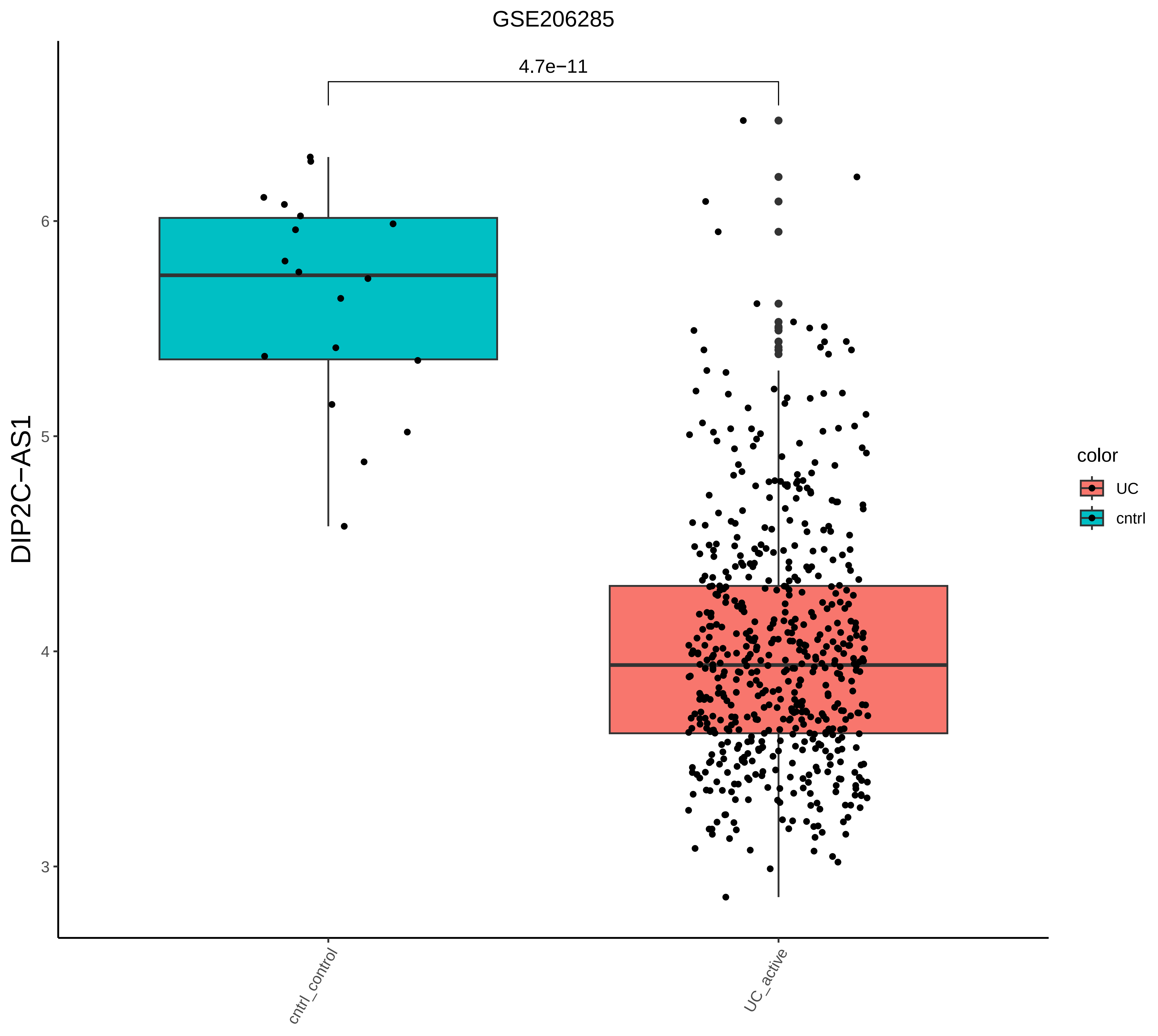

GSE206285
4.7e−11
DIP2C−AS1
6
5
4
3
cntrl_control
UC_active
color
UC
cntrl

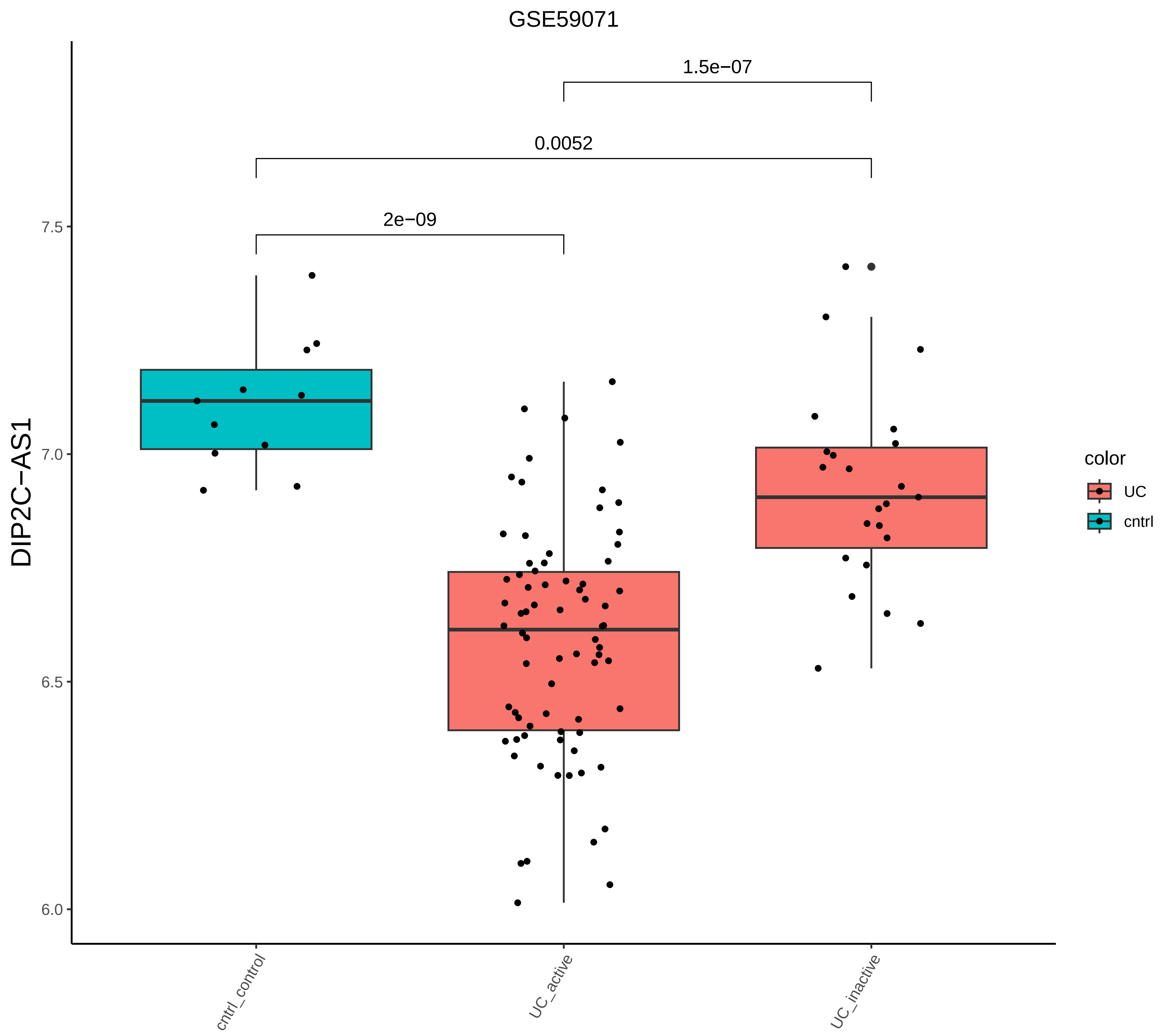

GSE59071
1.5e−07
0.0052
2e−09
DIP2C−AS1
7.5
7.0
6.5
6.0
cntrl_control
UC_active
UC_inactive
color
UC
cntrl

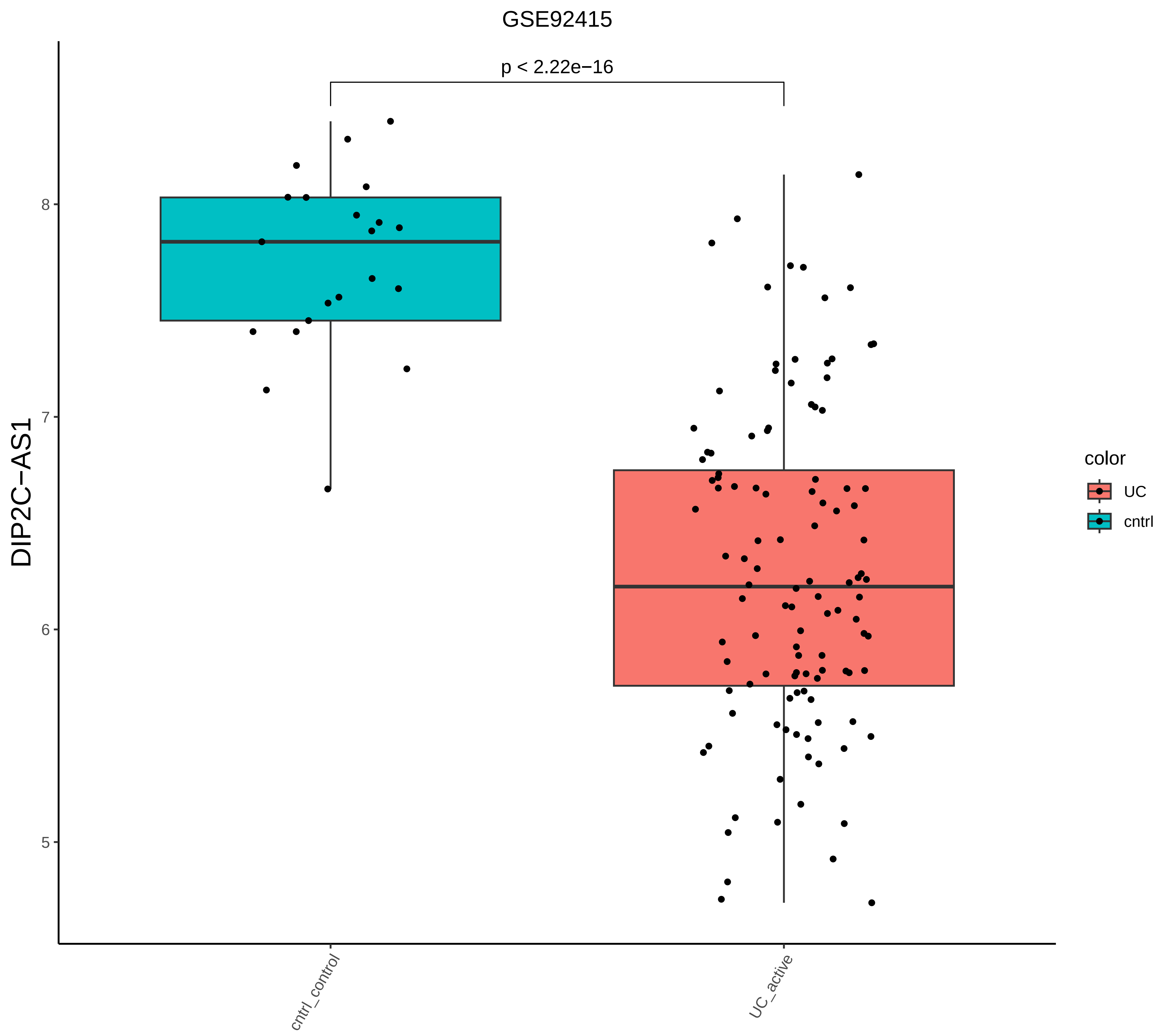

GSE92415
p < 2.22e−16
DIP2C−AS1
8
7
6
5
cntrl_control
UC_active
color
UC
cntrl

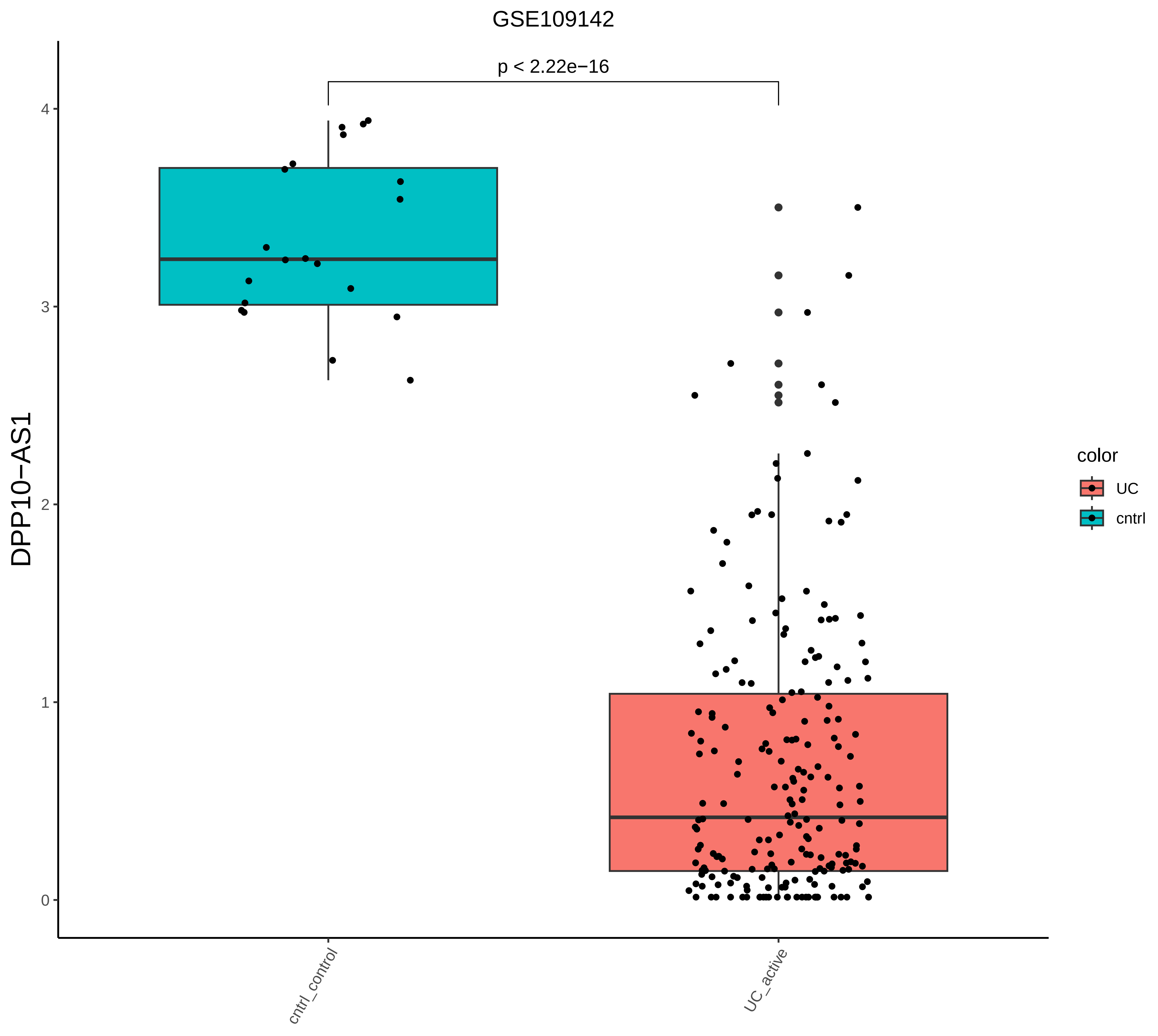
GSE109142
p < 2.22e−16
DPP10−AS1
0
1
2
3
4
cntrl_control
UC_active
color
UC
cntrl

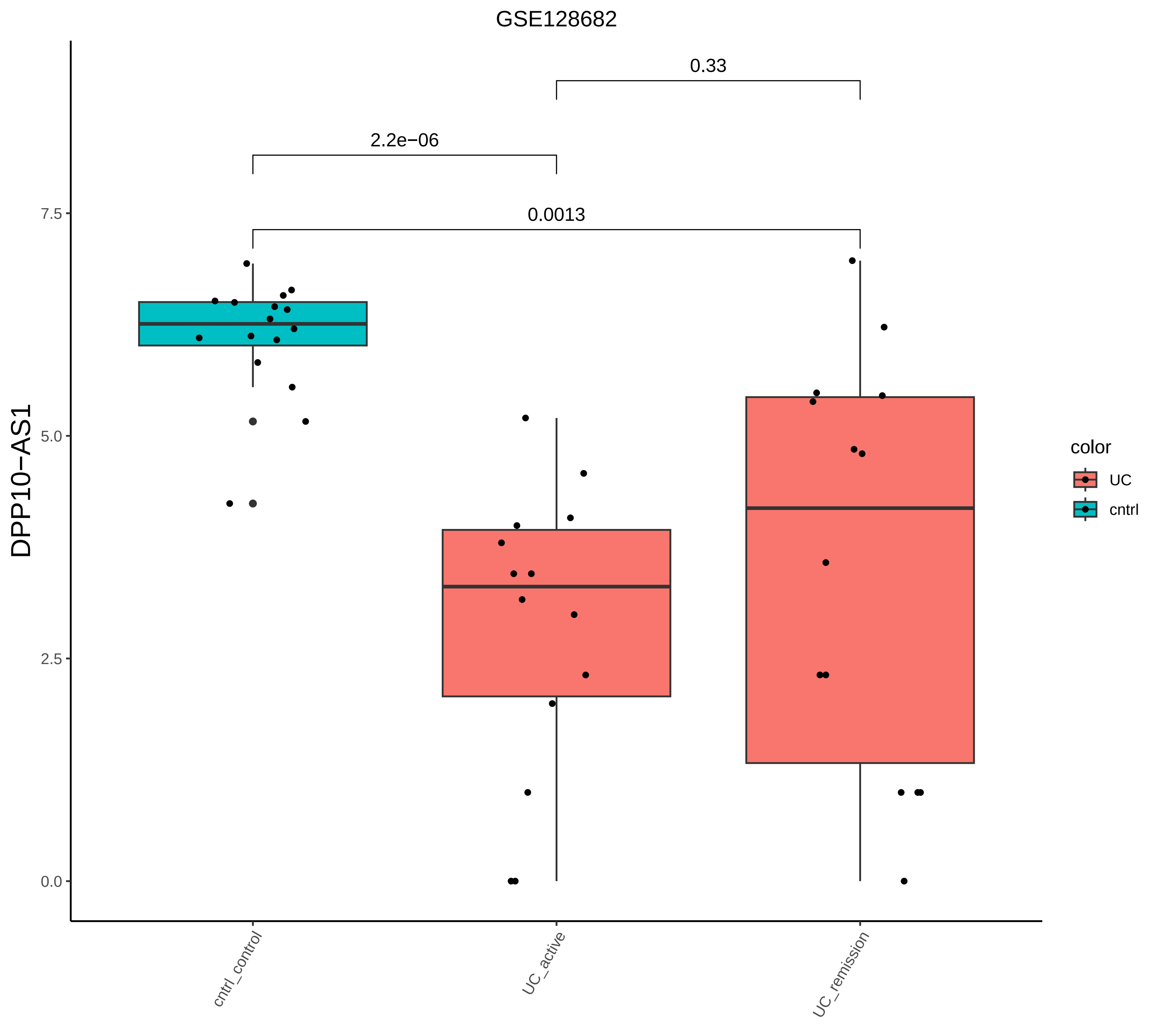
GSE128682
0.33
2.2e−06
0.0013
DPP10−AS1
7.5
5.0
2.5
0.0
cntrl_control
UC_active
UC_remission
color
UC
cntrl

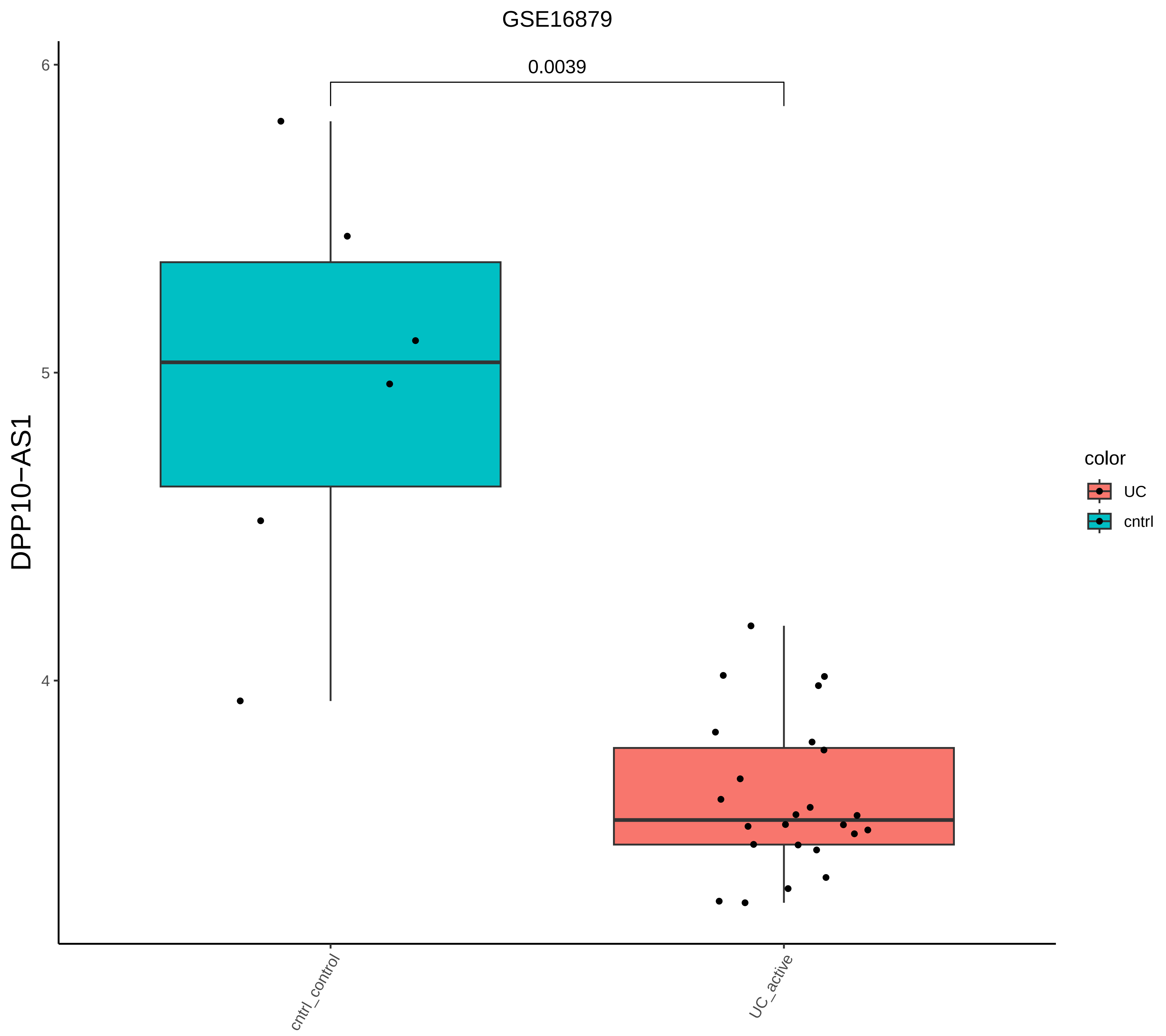
GSE16879
0.0039
DPP10−AS1
6
5
4
cntrl_control
UC_active
color
UC
cntrl

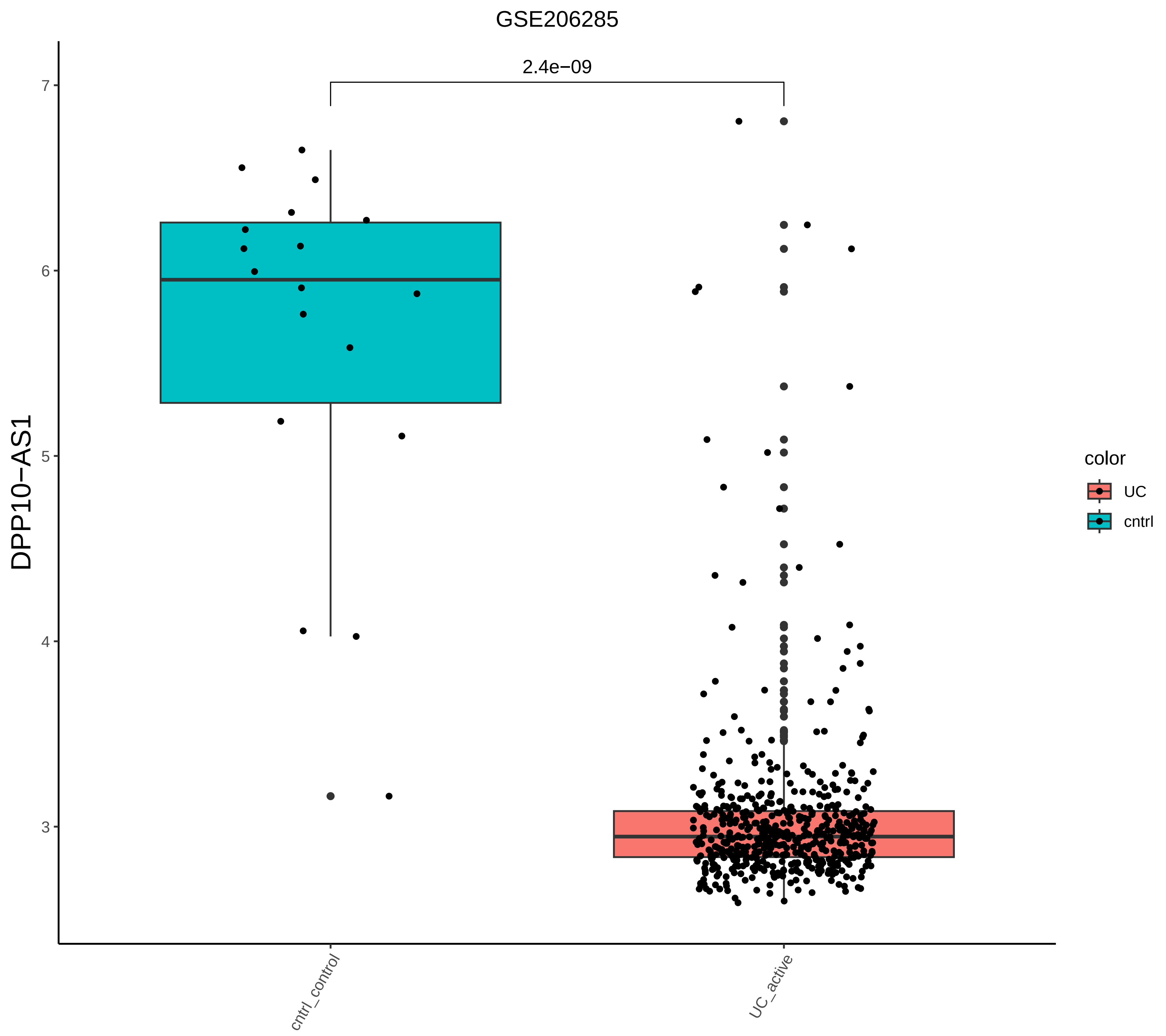

GSE206285
2.4e−09
DPP10−AS1
7
6
5
4
3
cntrl_control
UC_active
color
UC
cntrl

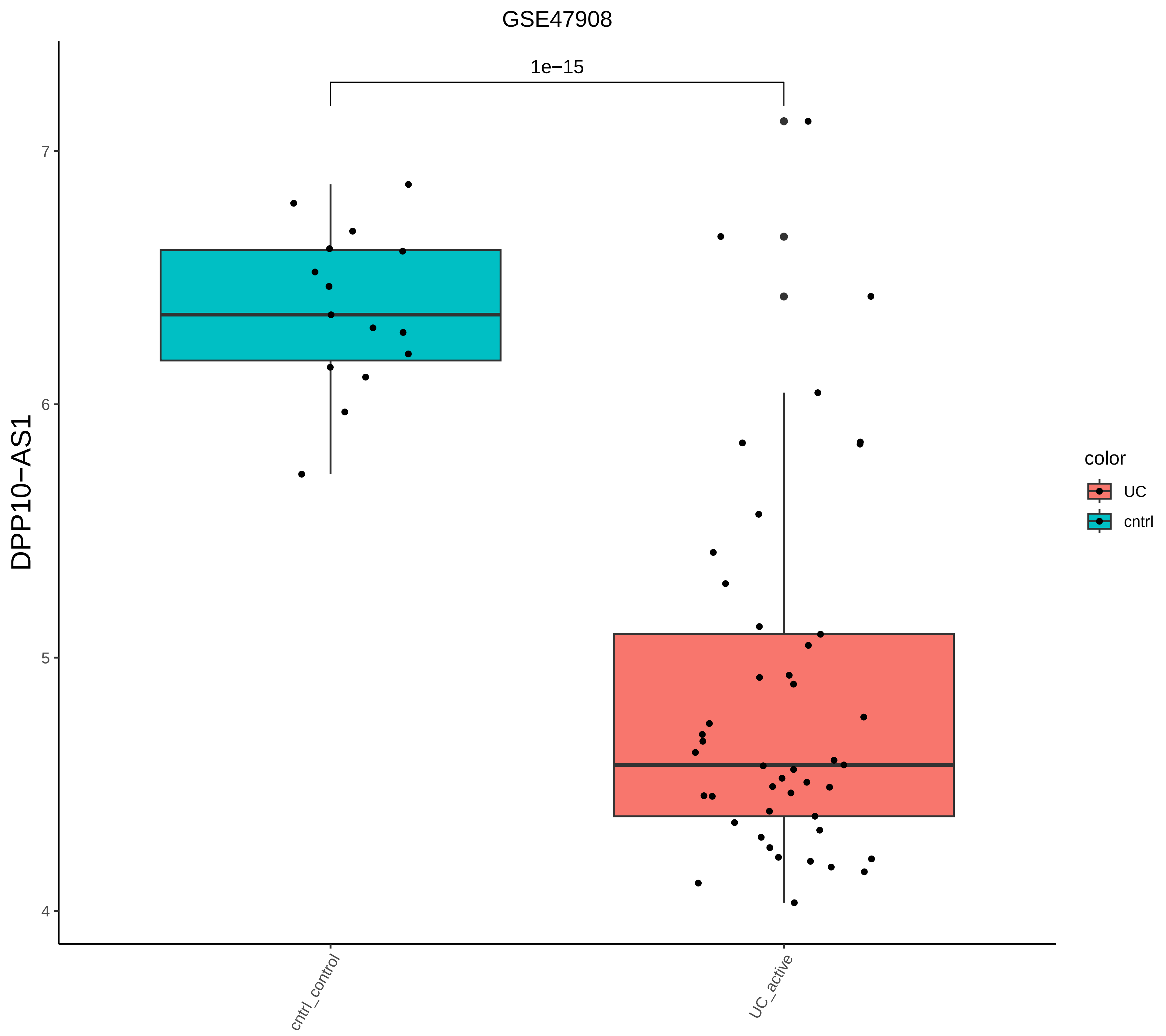

GSE47908
1e−15
DPP10−AS1
7
6
5
4
cntrl_control
UC_active
color
UC
cntrl

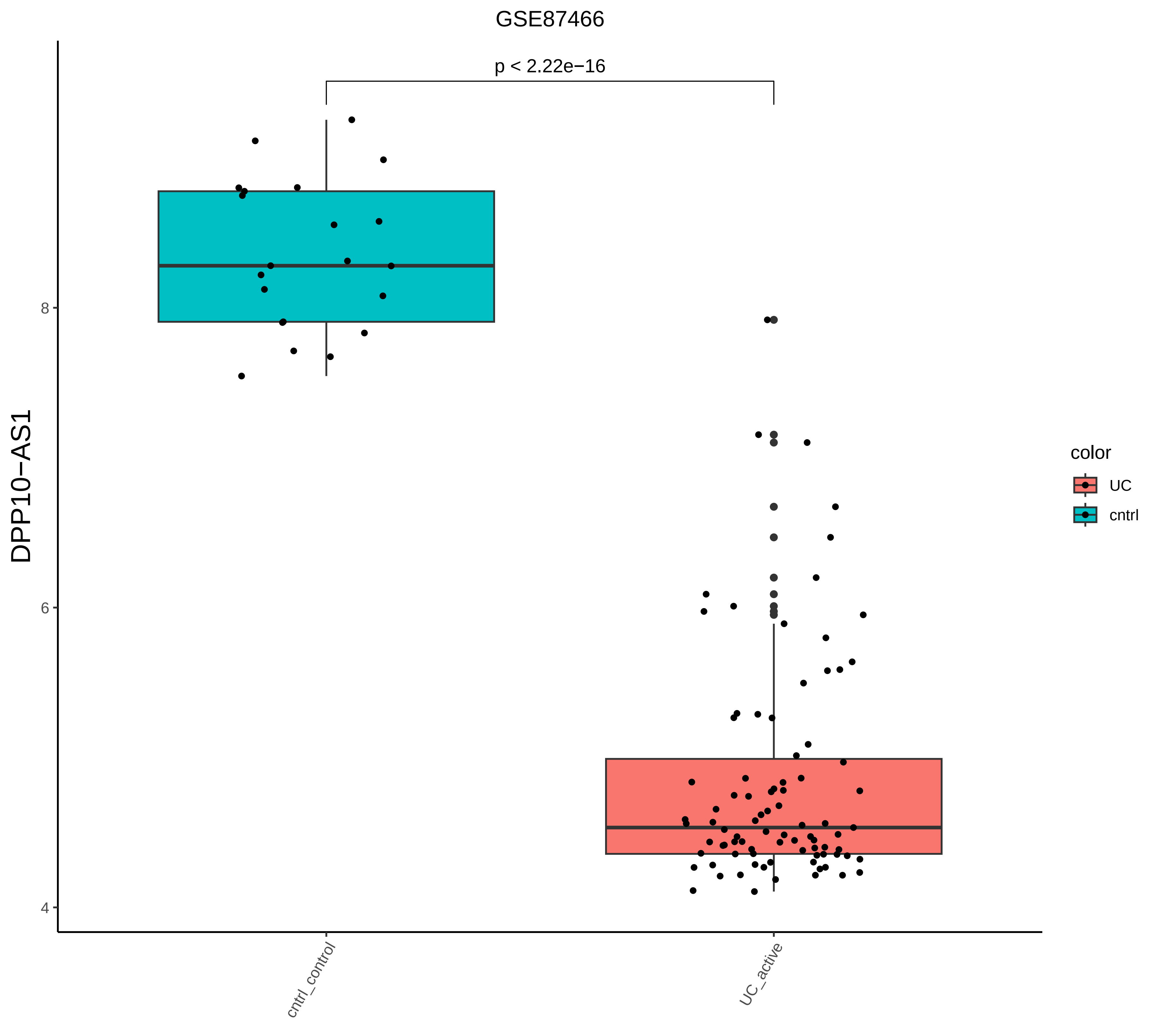

GSE87466
p < 2.22e−16
DPP10−AS1
8
6
4
cntrl_control
UC_active
color
UC
cntrl

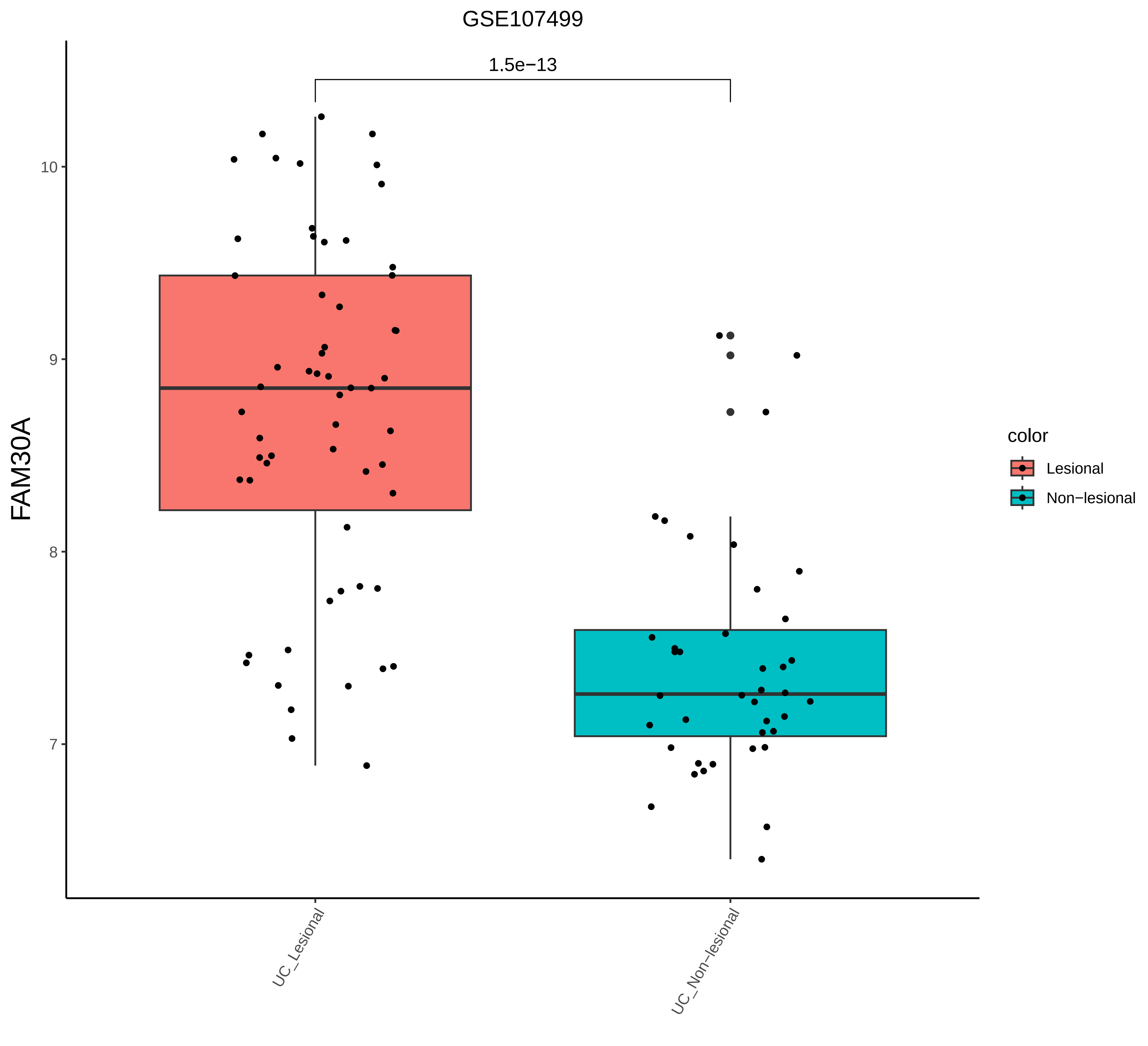

GSE107499
1.5e−13
FAM30A
10
9
8
7
UC_Lesional
UC_Non−lesional
color
Lesional
Non−lesional

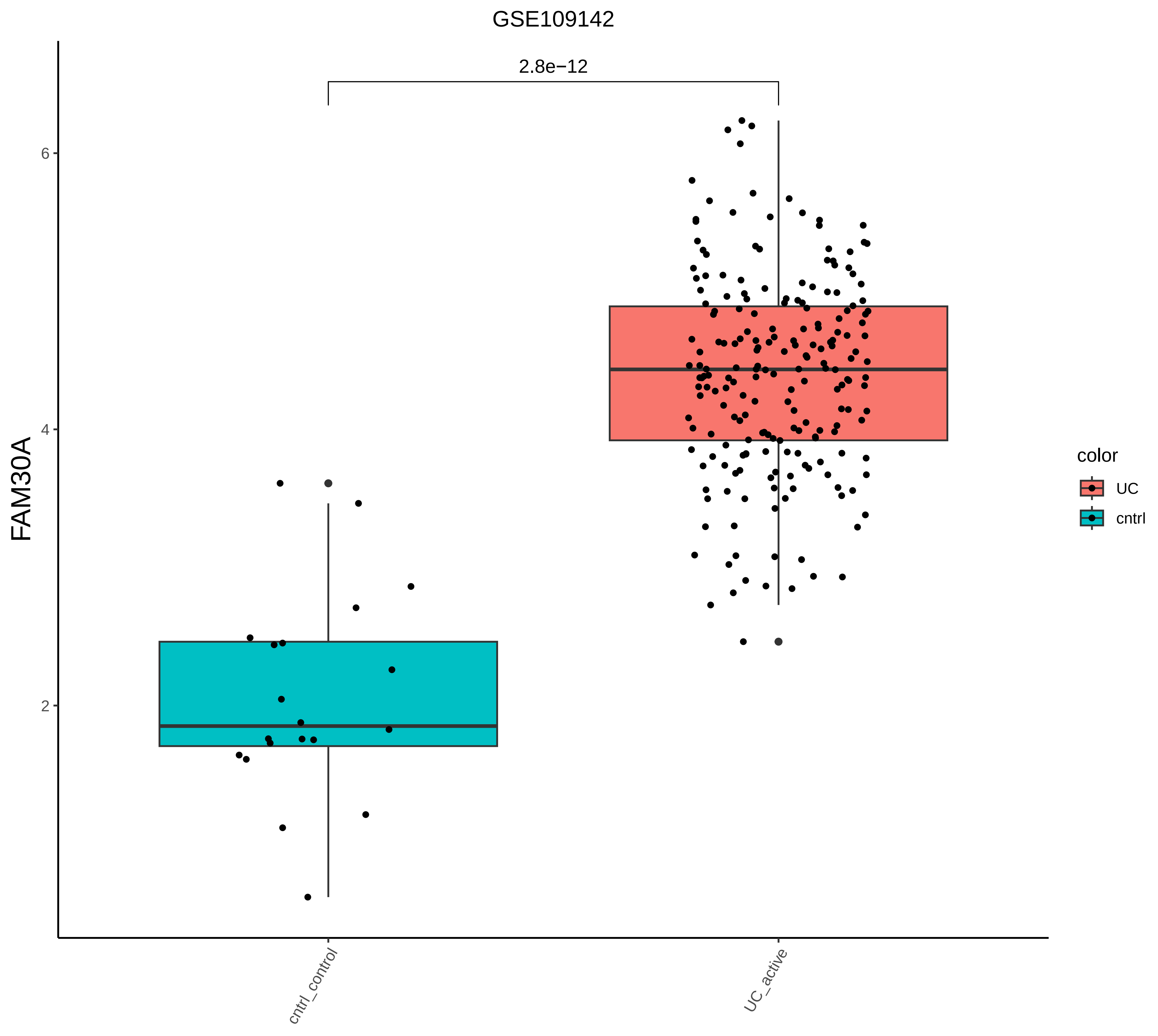

GSE109142
2.8e−12
FAM30A
6
4
2
cntrl_control
UC_active
color
UC
cntrl

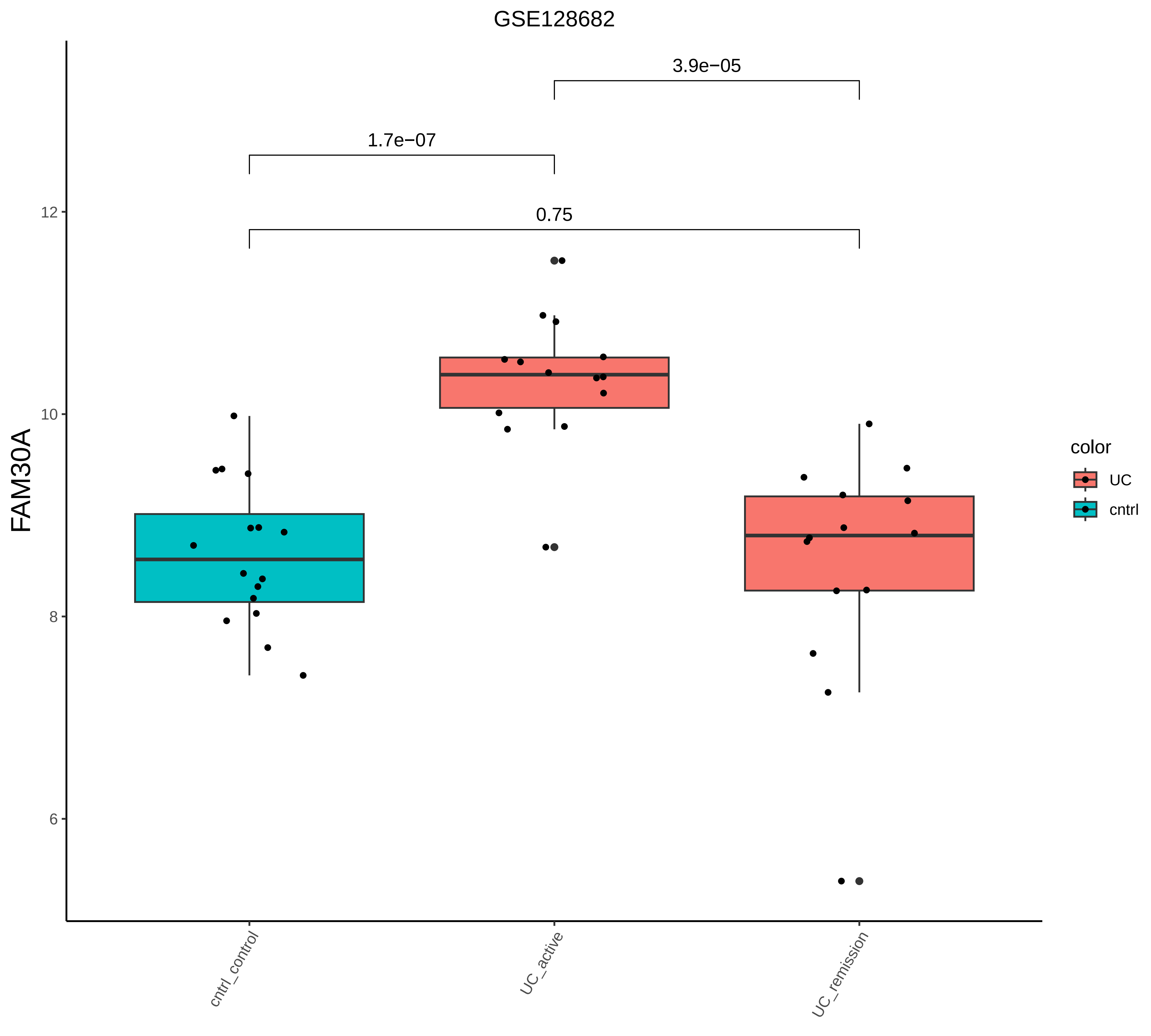
GSE128682
3.9e−05
1.7e−07
0.75
FAM30A
12
10
8
6
cntrl_control
UC_active
UC_remission
color
UC
cntrl

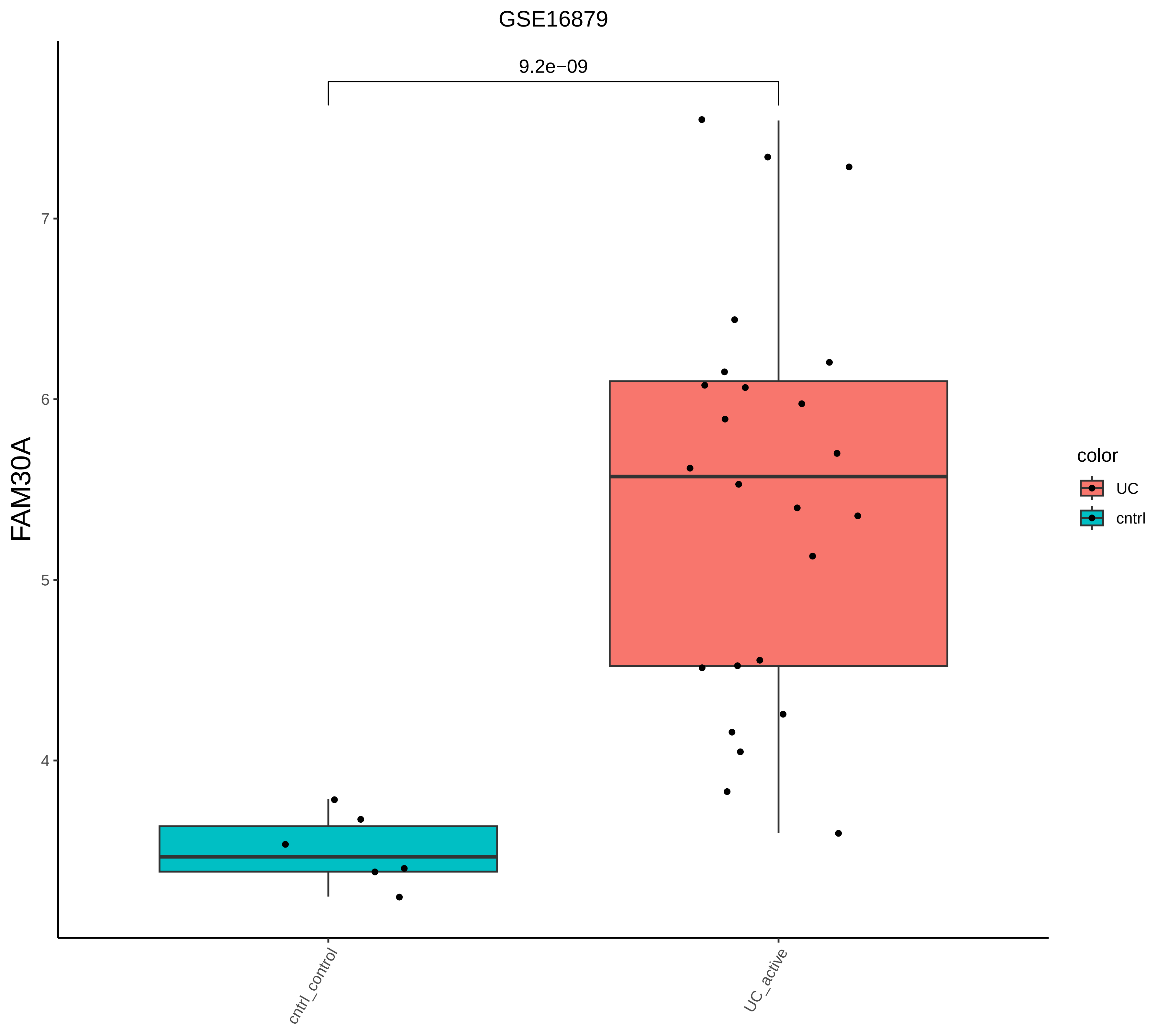

GSE16879
9.2e−09
FAM30A
7
6
5
4
cntrl_control
UC_active
color
UC
cntrl

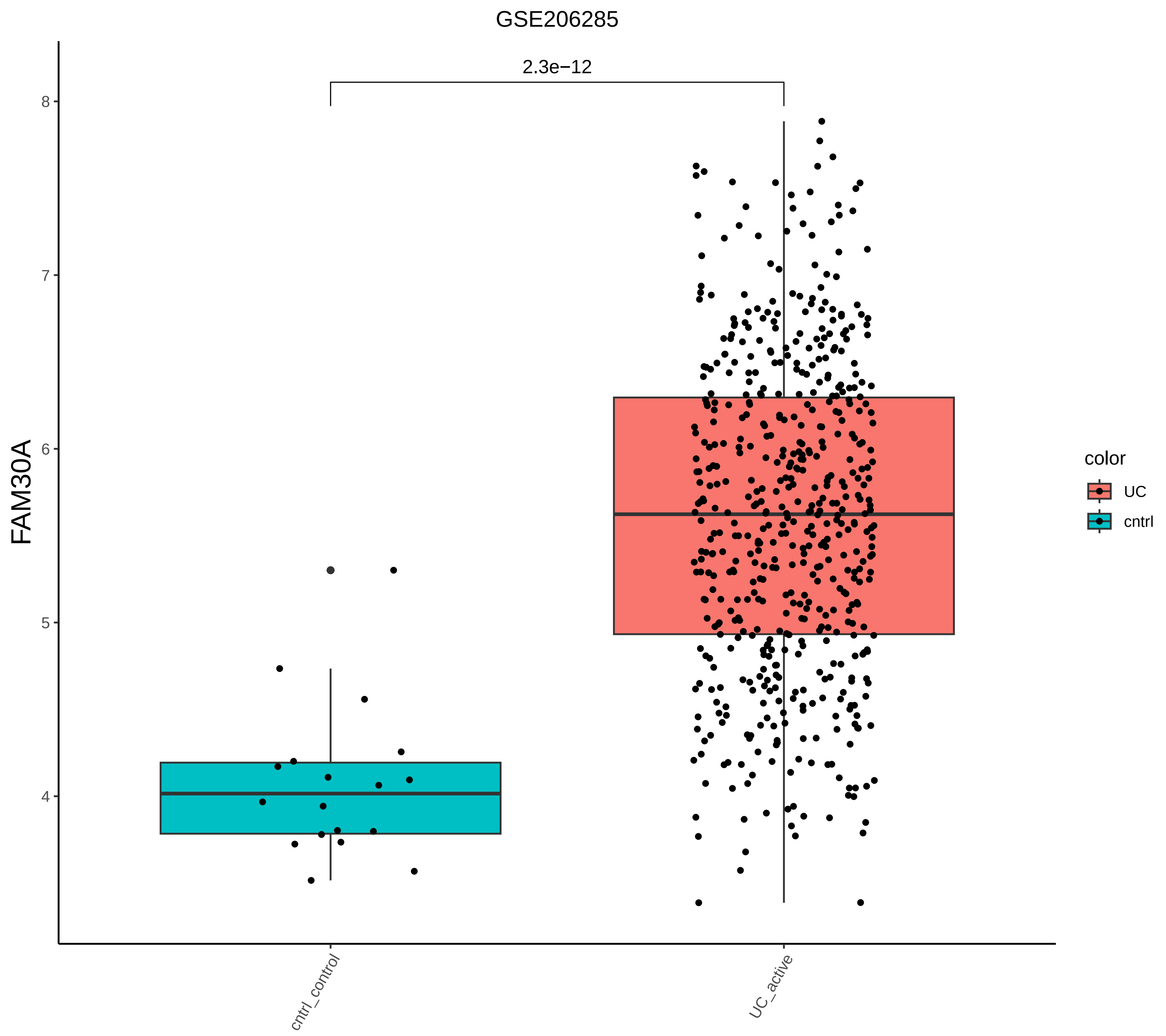
GSE206285
2.3e−12
FAM30A
8
7
6
5
4
cntrl_control
UC_active
color
UC
cntrl

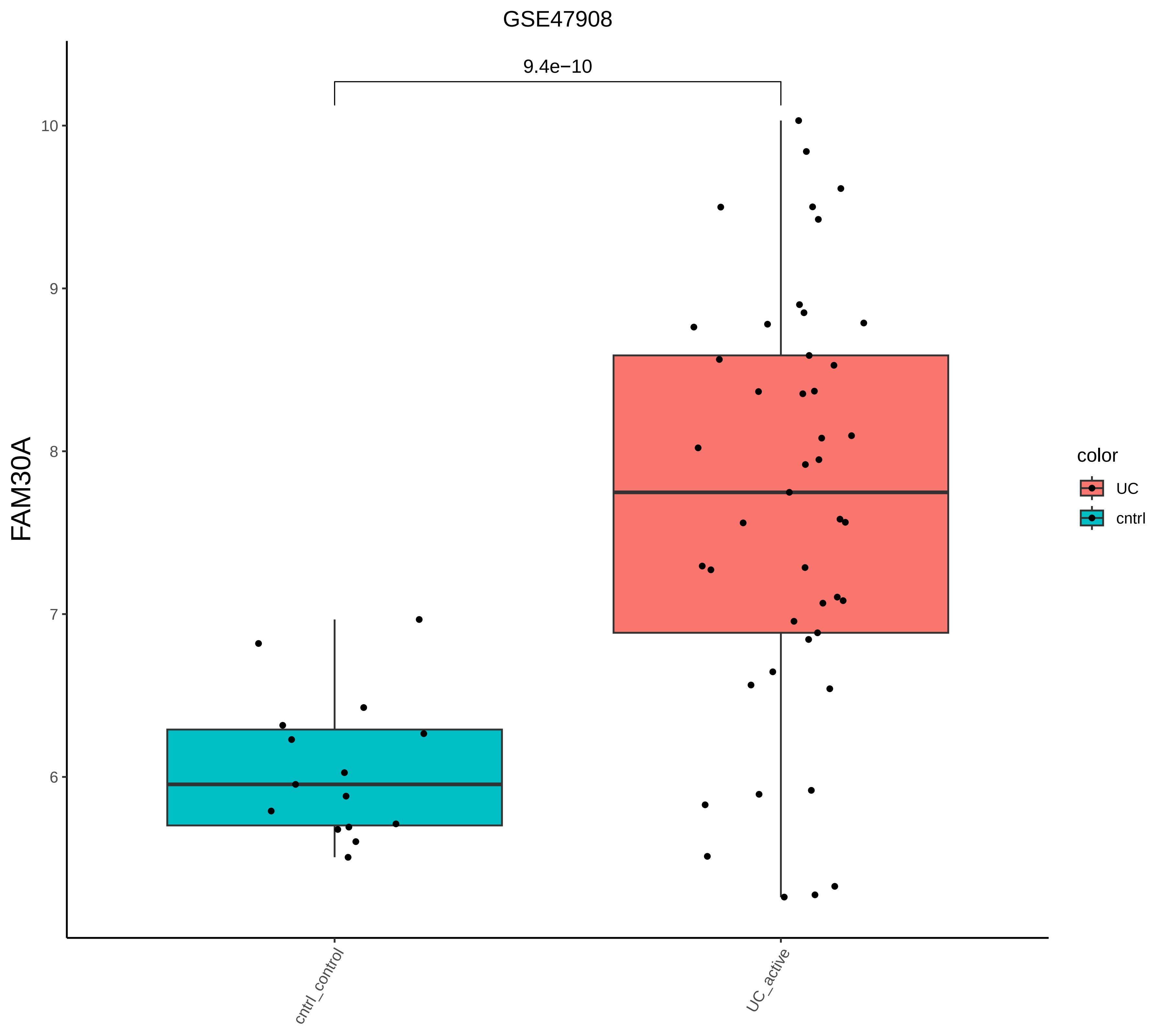

GSE47908
9.4e−10
FAM30A
10
9
8
7
6
cntrl_control
UC_active
color
UC
cntrl

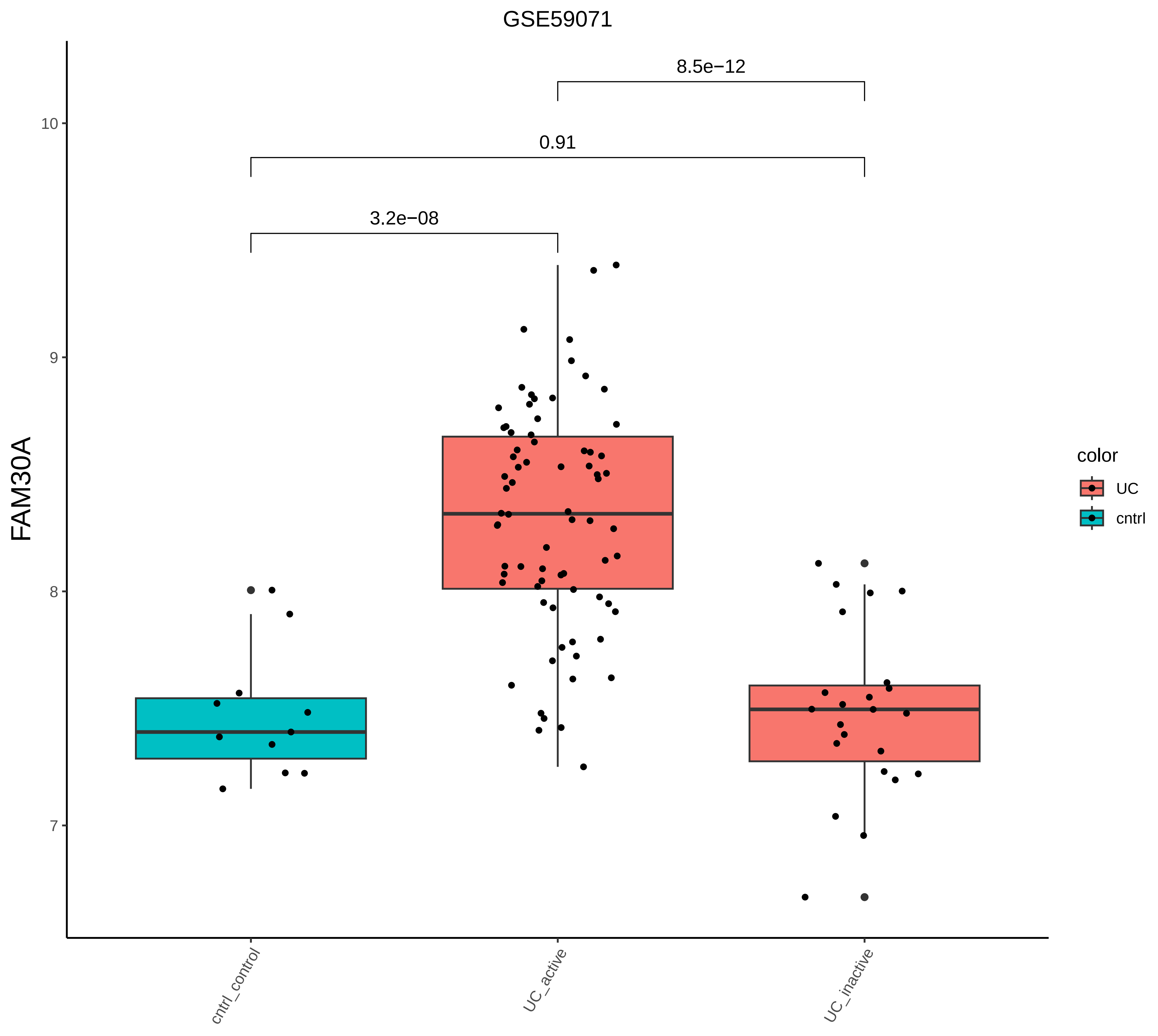
GSE59071
8.5e−12
0.91
3.2e−08
FAM30A
10
9
8
7
cntrl_control
UC_active
UC_inactive
color
UC
cntrl

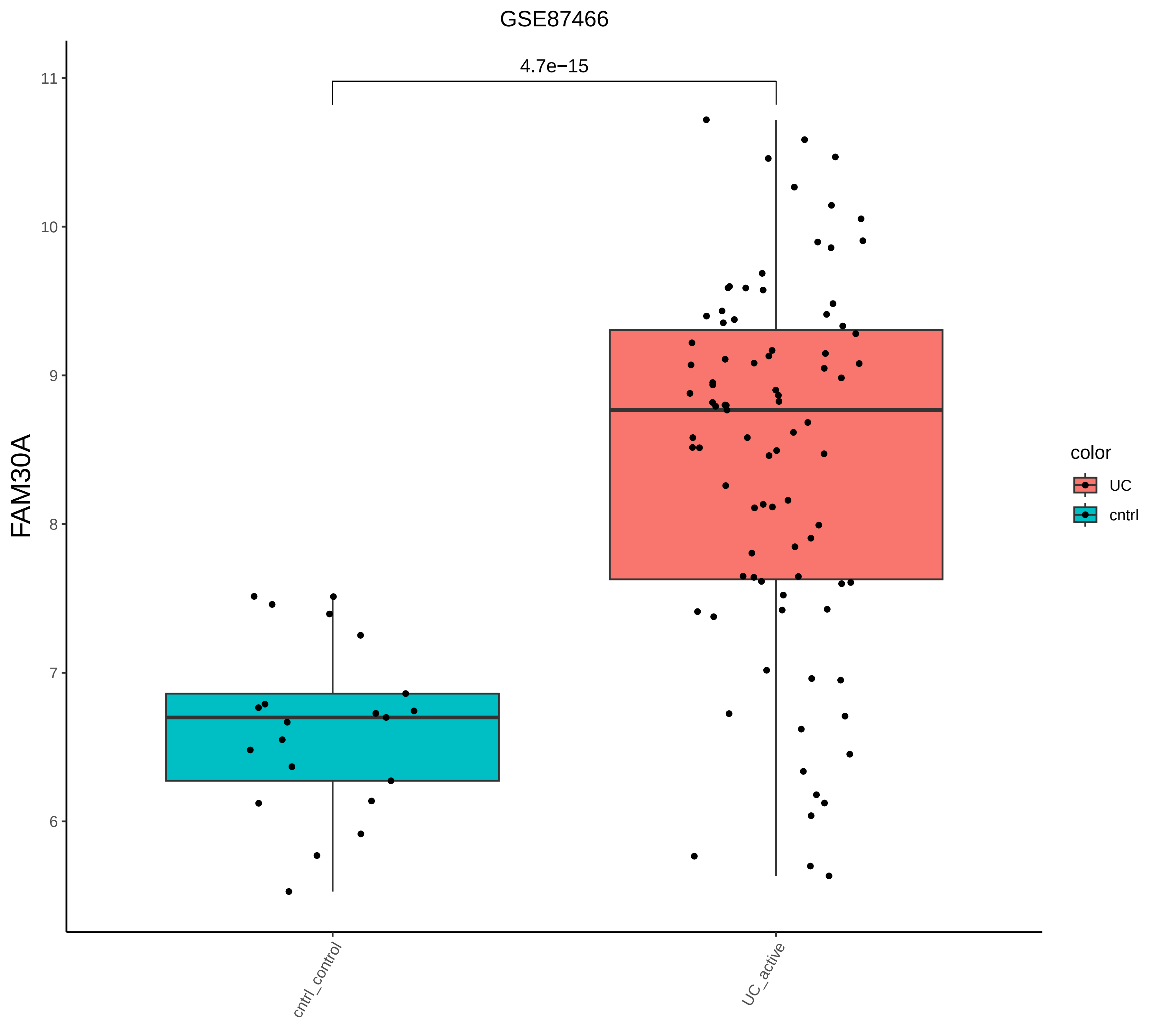

GSE87466
4.7e−15
FAM30A
11
10
9
8
7
6
cntrl_control
UC_active
color
UC
cntrl

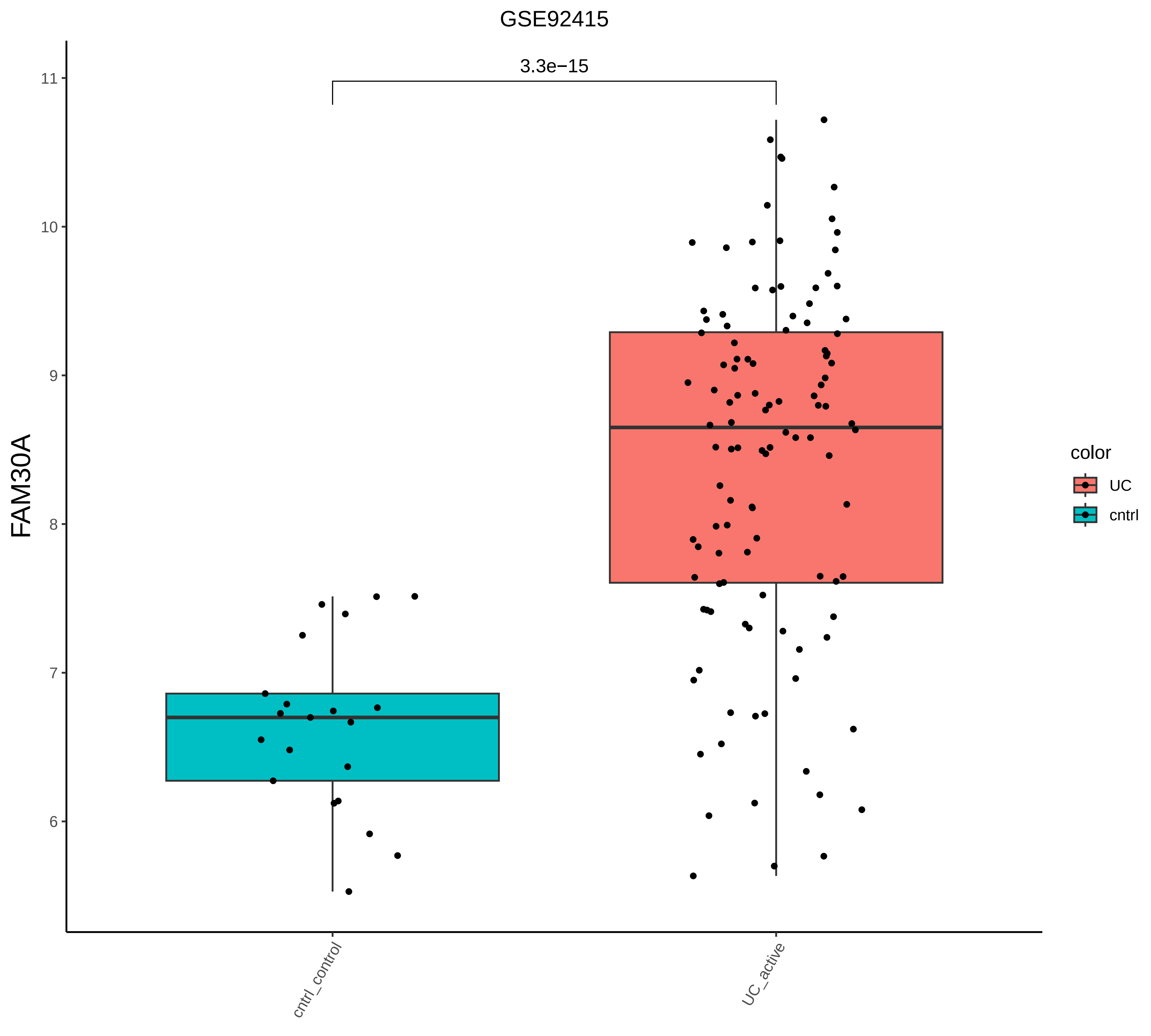
GSE92415
3.3e−15
FAM30A
11
10
9
8
7
6
cntrl_control
UC_active
color
UC
cntrl

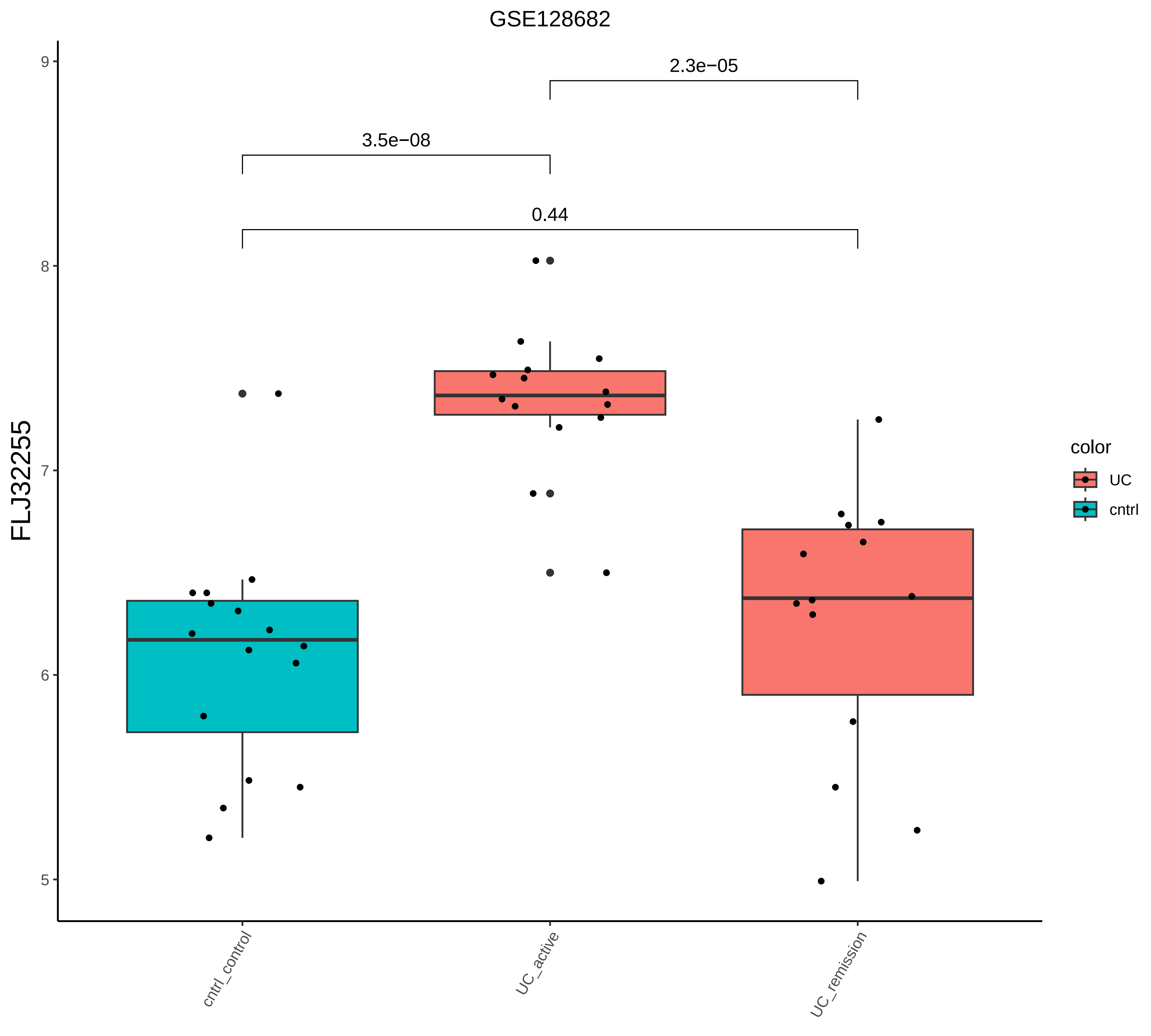

GSE128682
FLJ32255
2.3e−05
3.5e−08
0.44
9
8
7
6
5
cntrl_control
UC_active
UC_remission
color
UC
cntrl

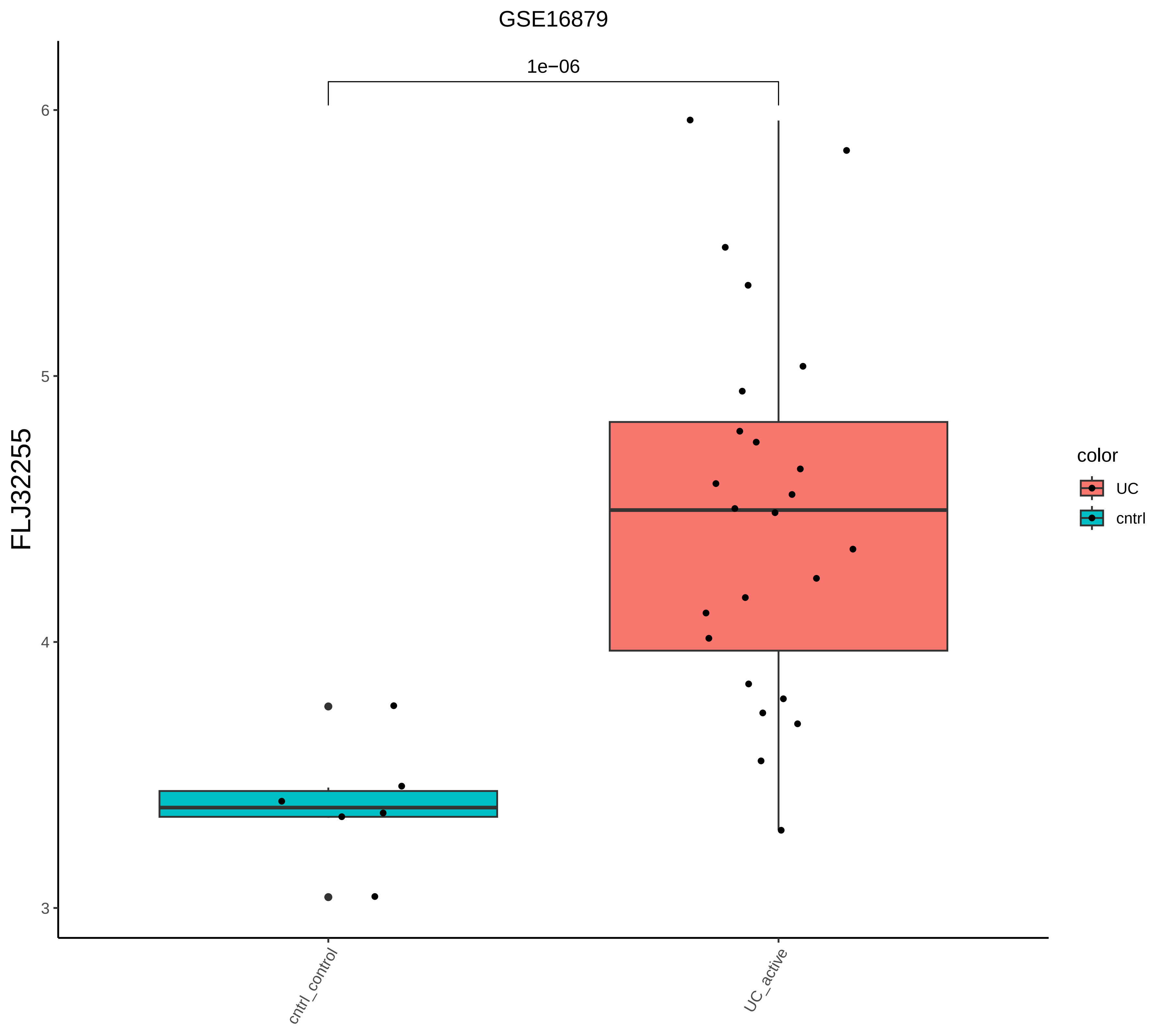
GSE16879
1e−06
FLJ32255
6
5
4
3
cntrl_control
UC_active
color
UC
cntrl

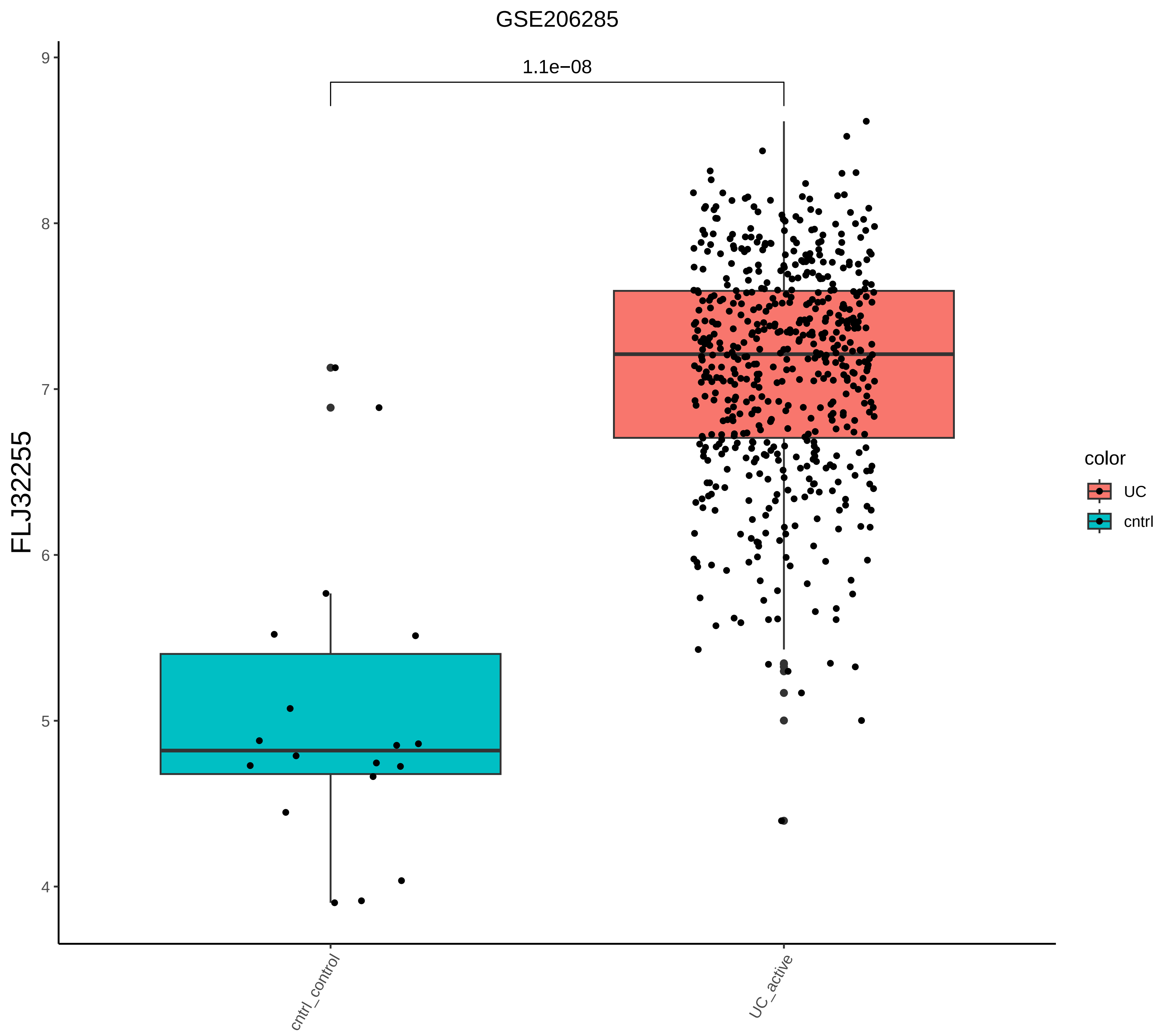
GSE206285
1.1e−08
FLJ32255
9
8
7
6
5
4
cntrl_control
UC_active
color
UC
cntrl

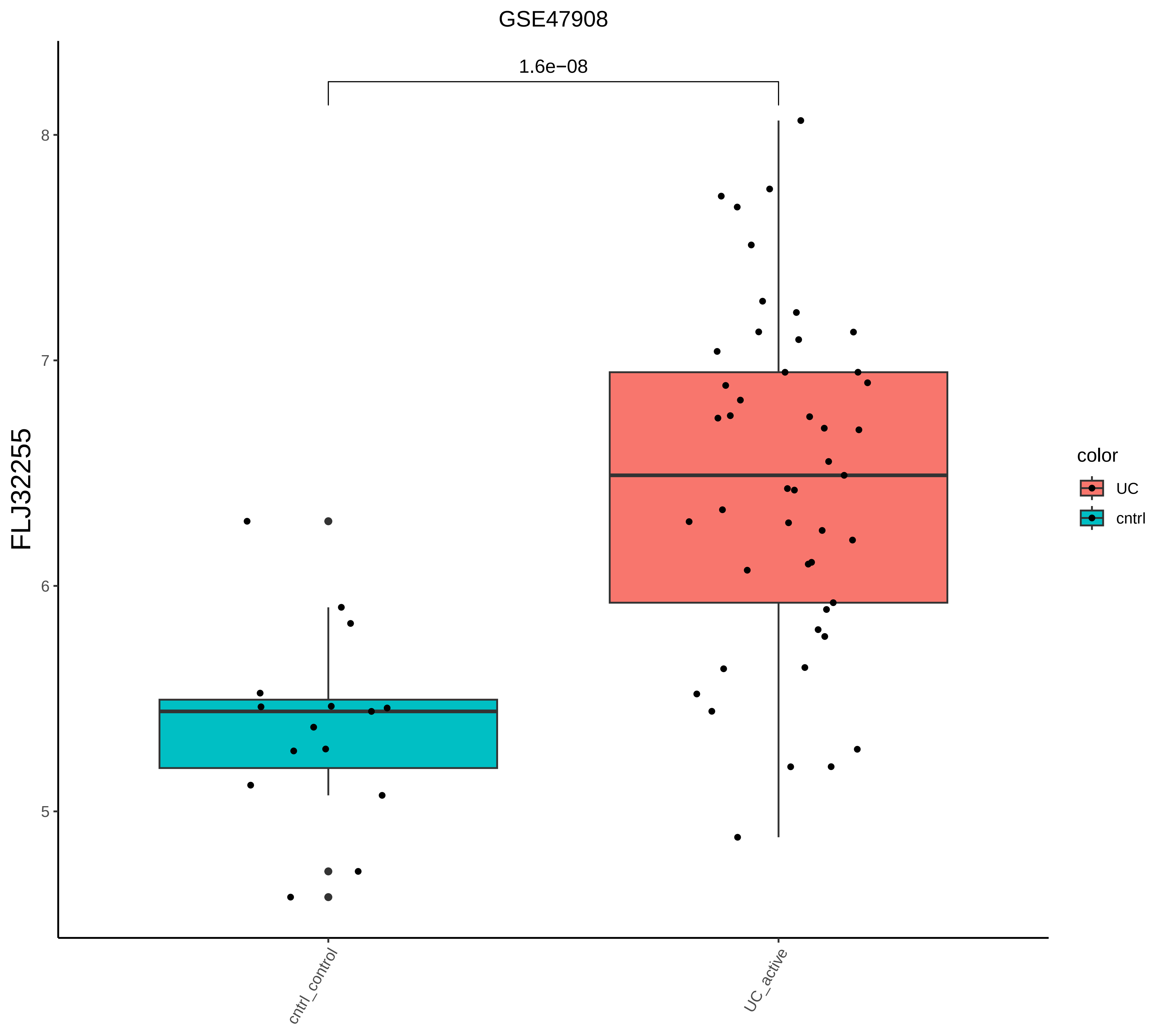
GSE47908
1.6e−08
FLJ32255
8
7
6
5
cntrl_control
UC_active
color
UC
cntrl

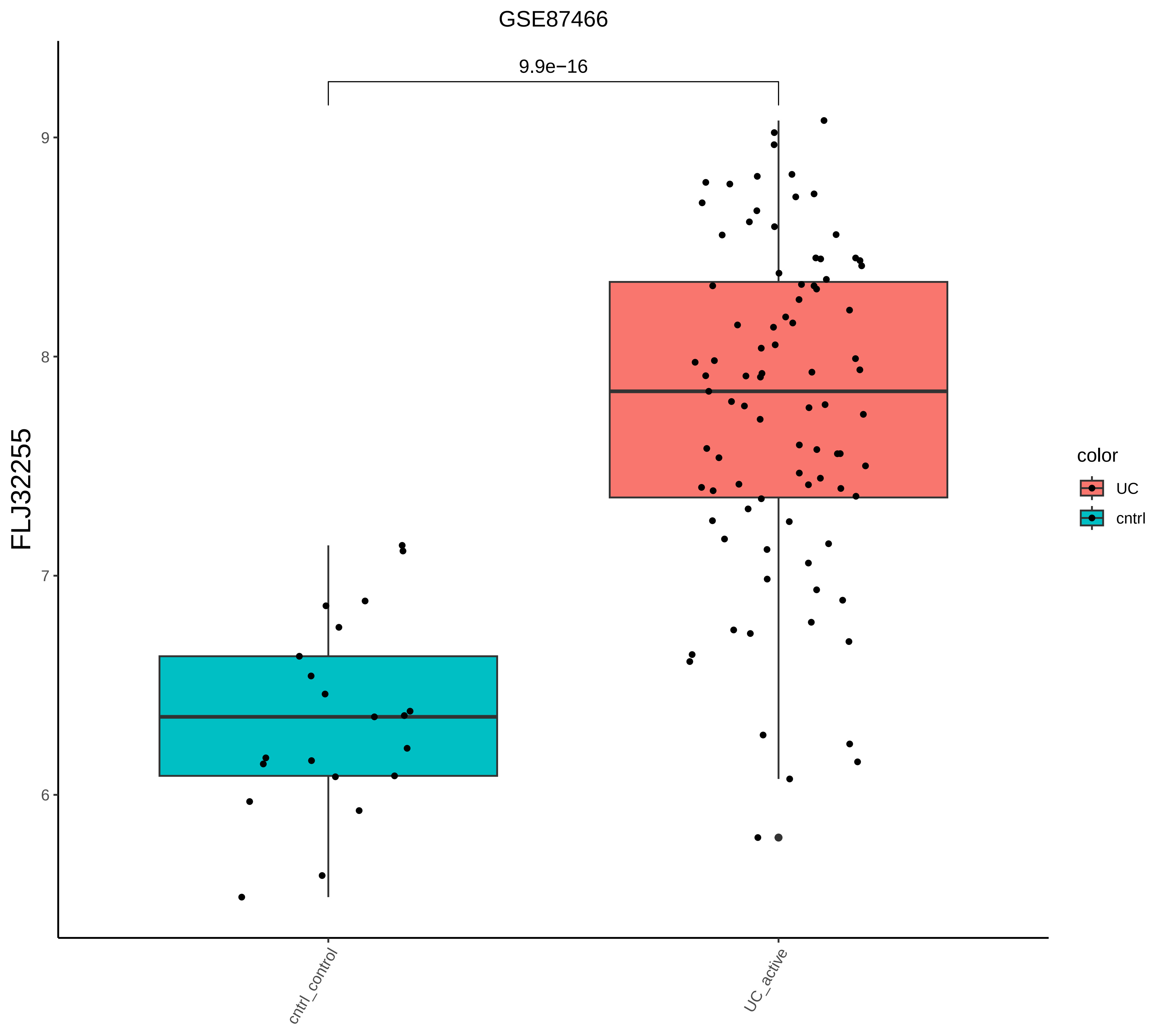

GSE87466
9.9e−16
FLJ32255
9
8
7
6
cntrl_control
UC_active
color
UC
cntrl

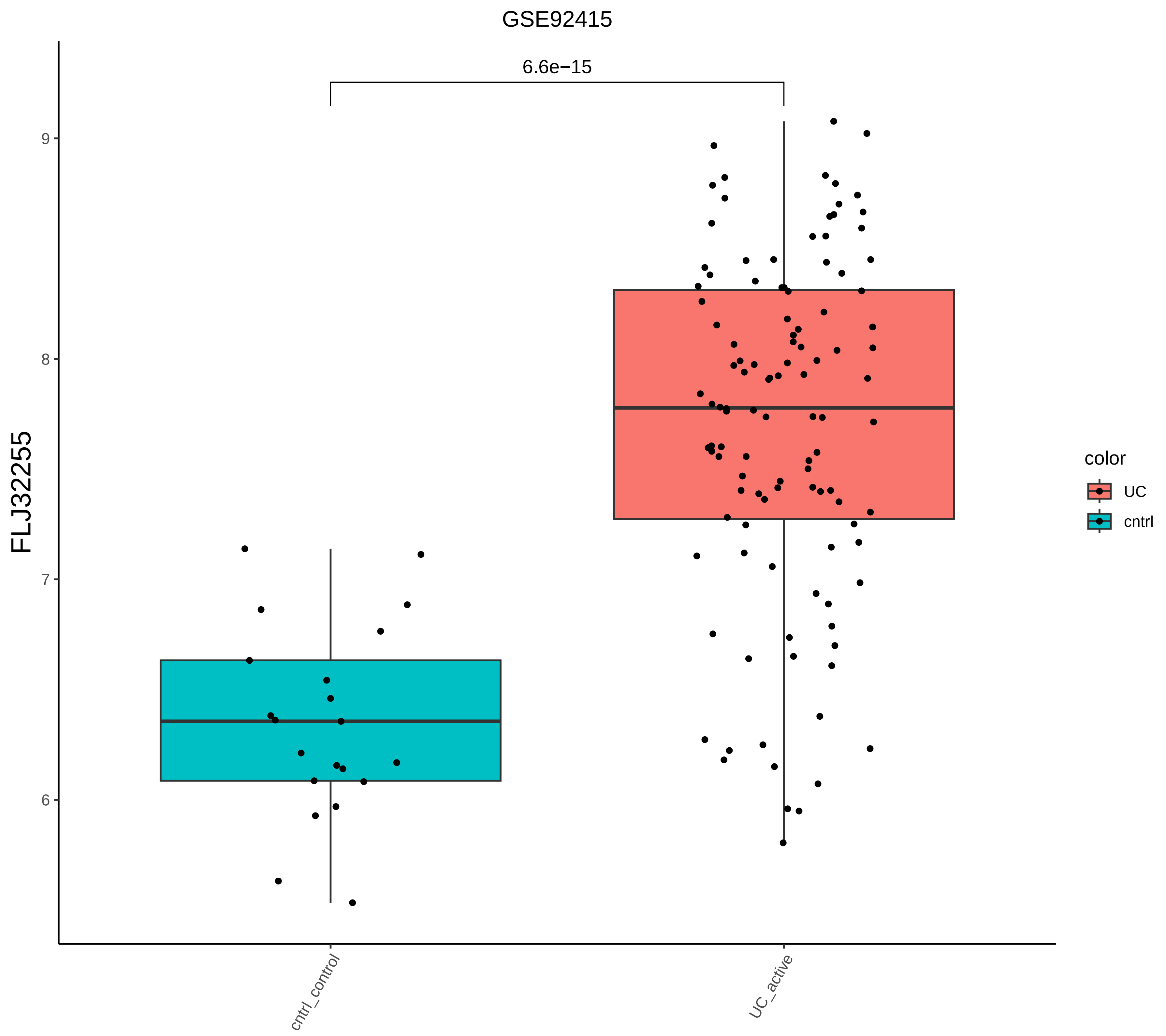
GSE92415
6.6e−15
FLJ32255
9
8
7
6
cntrl_control
UC_active
color
UC
cntrl

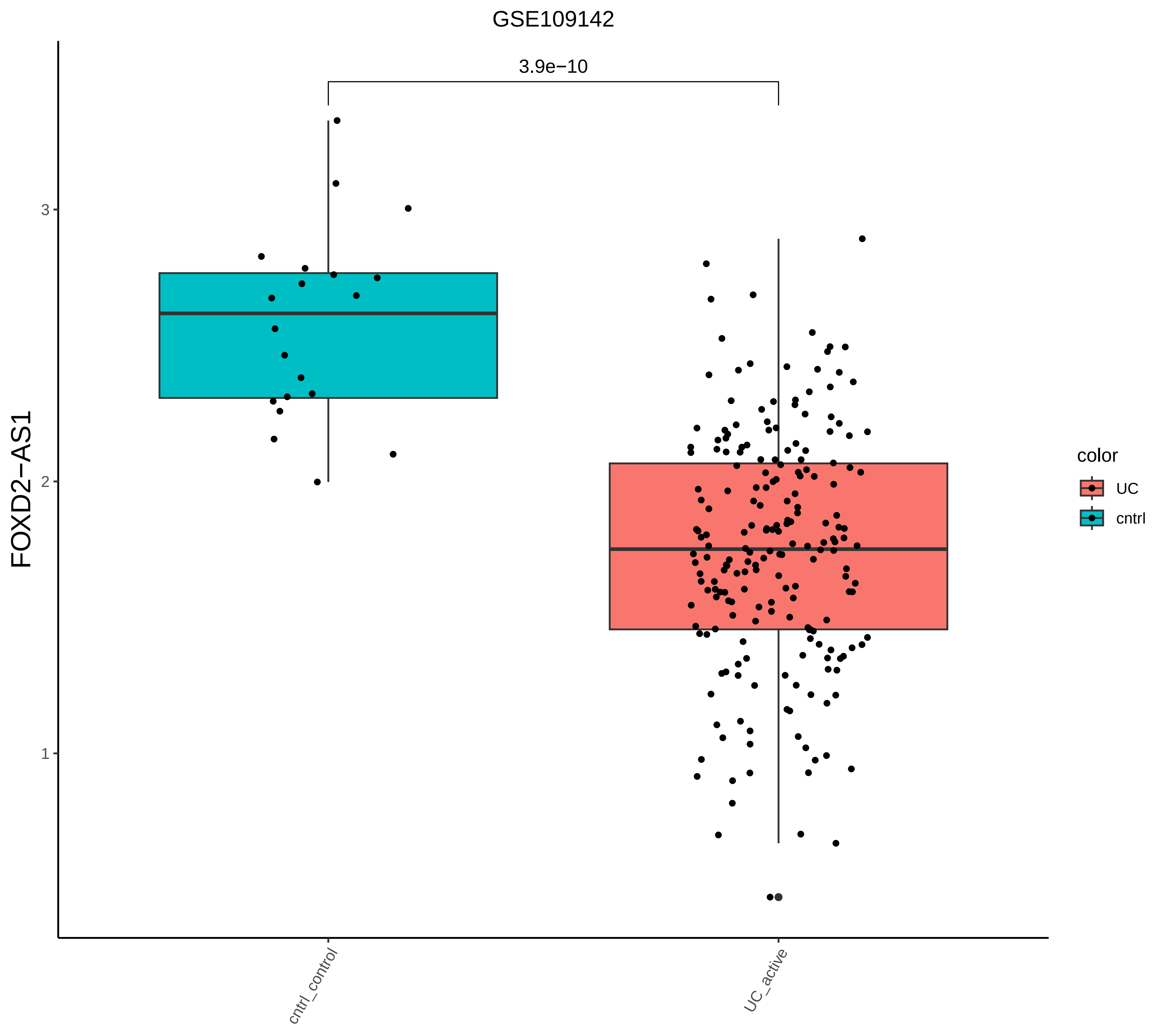
GSE109142
3.9e−10
FOXD2−AS1
3
2
1
cntrl_control
UC_active
color
UC
cntrl

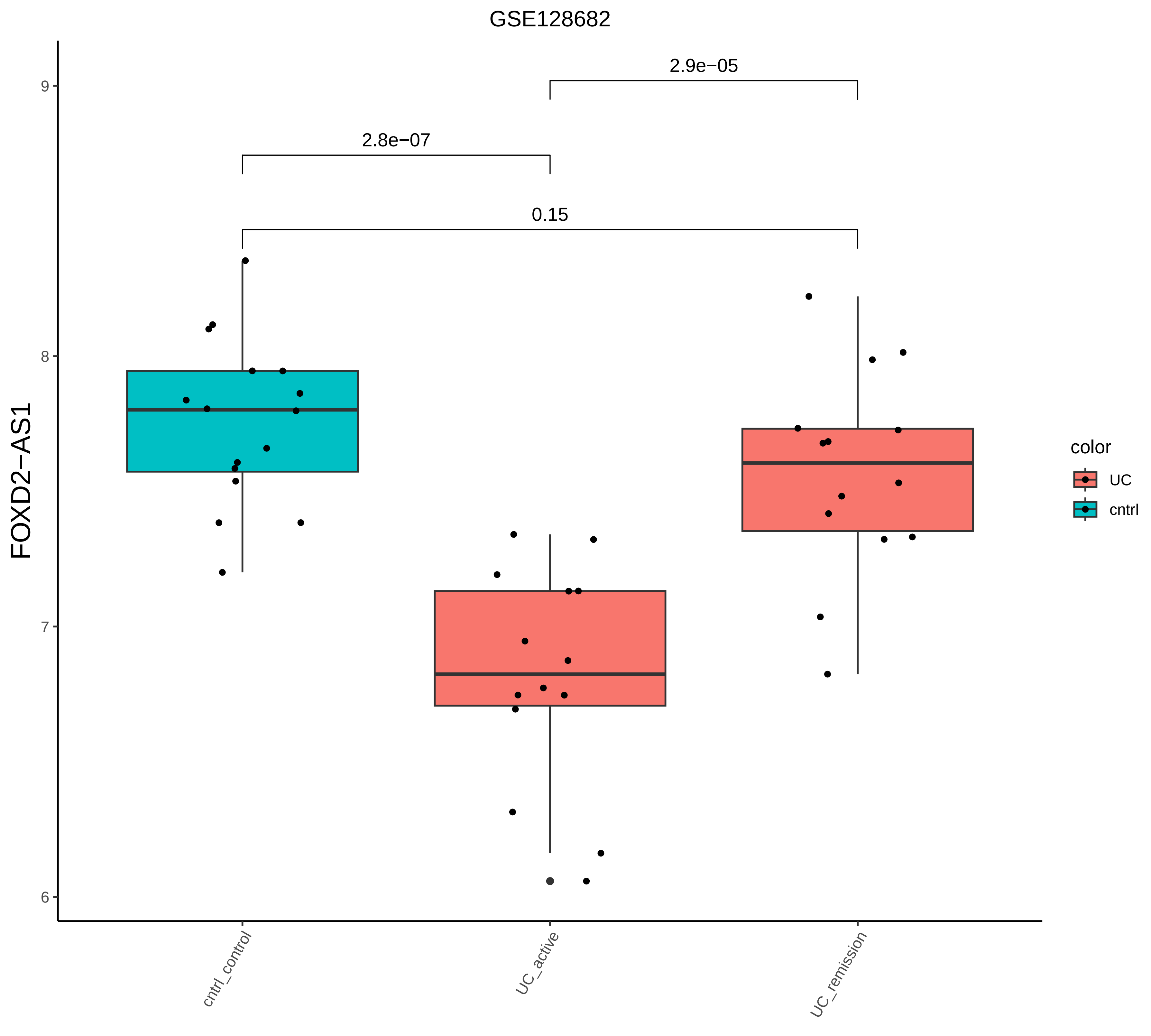
GSE128682
2.9e−05
2.8e−07
0.15
FOXD2−AS1
9
8
7
6
cntrl_control
UC_active
UC_remission
color
UC
cntrl

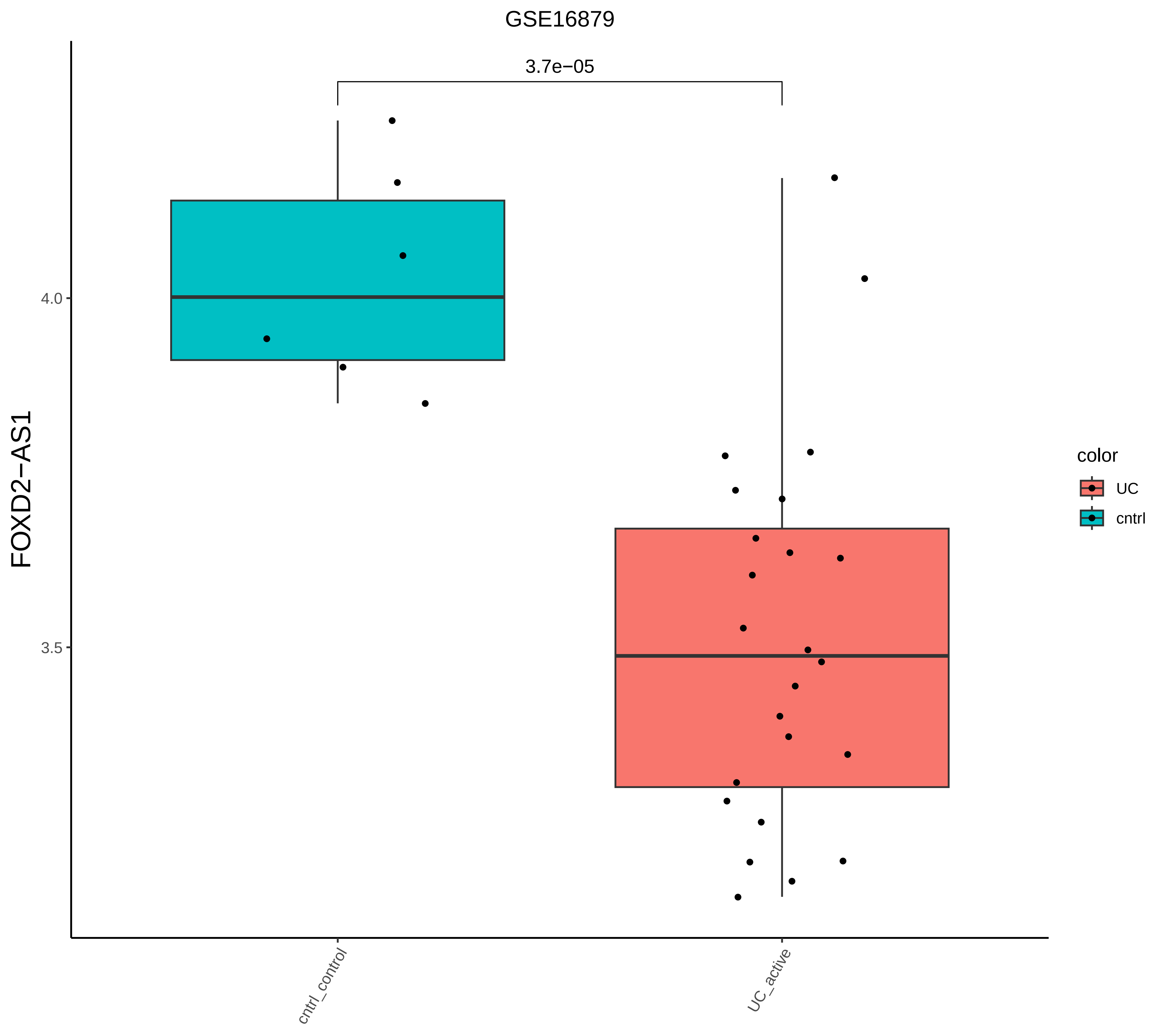
GSE16879
3.7e−05
FOXD2−AS1
4.0
3.5
cntrl_control
UC_active
color
UC
cntrl

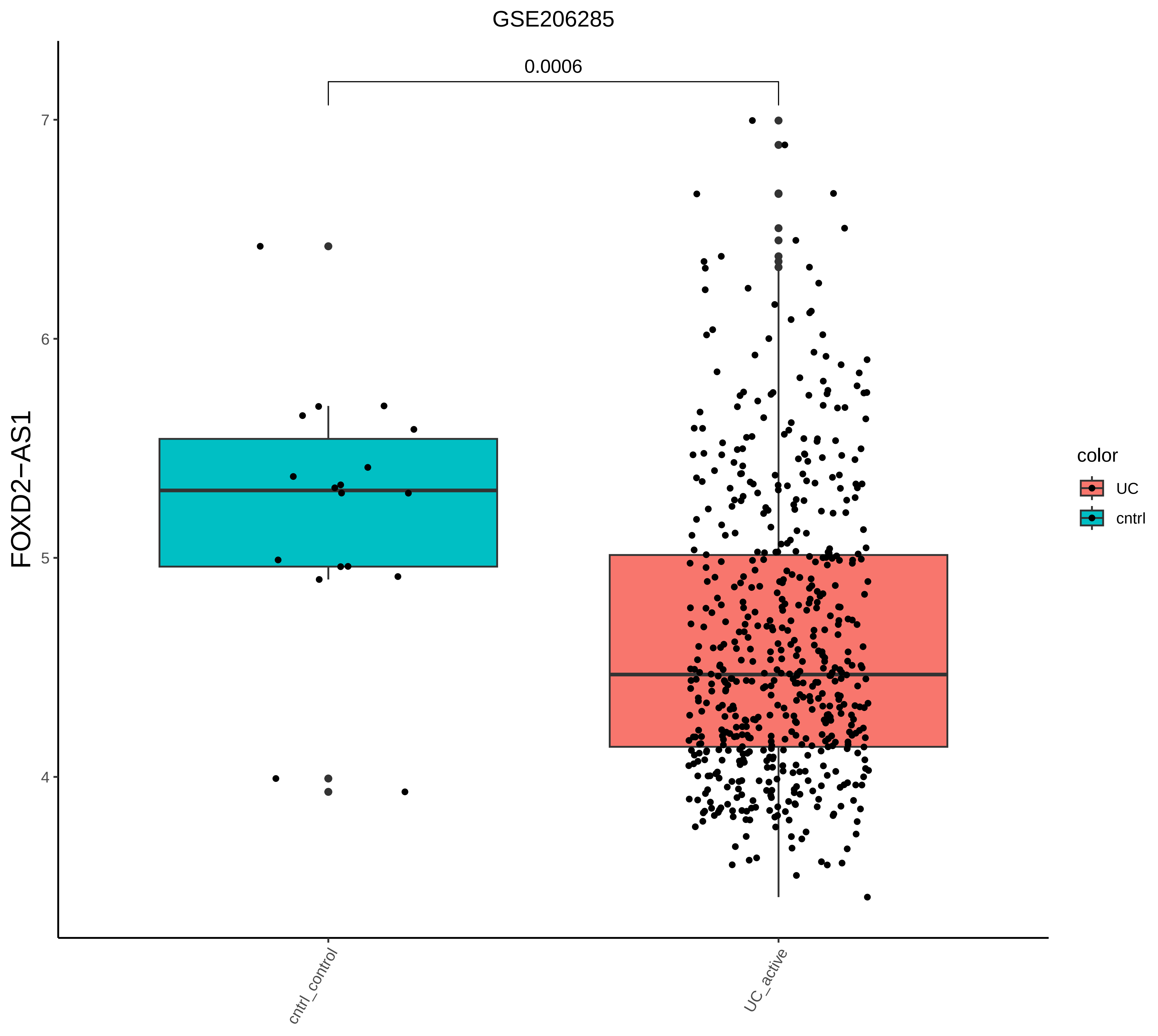
GSE206285
0.0006
FOXD2−AS1
7
6
5
4
cntrl_control
UC_active
color
UC
cntrl

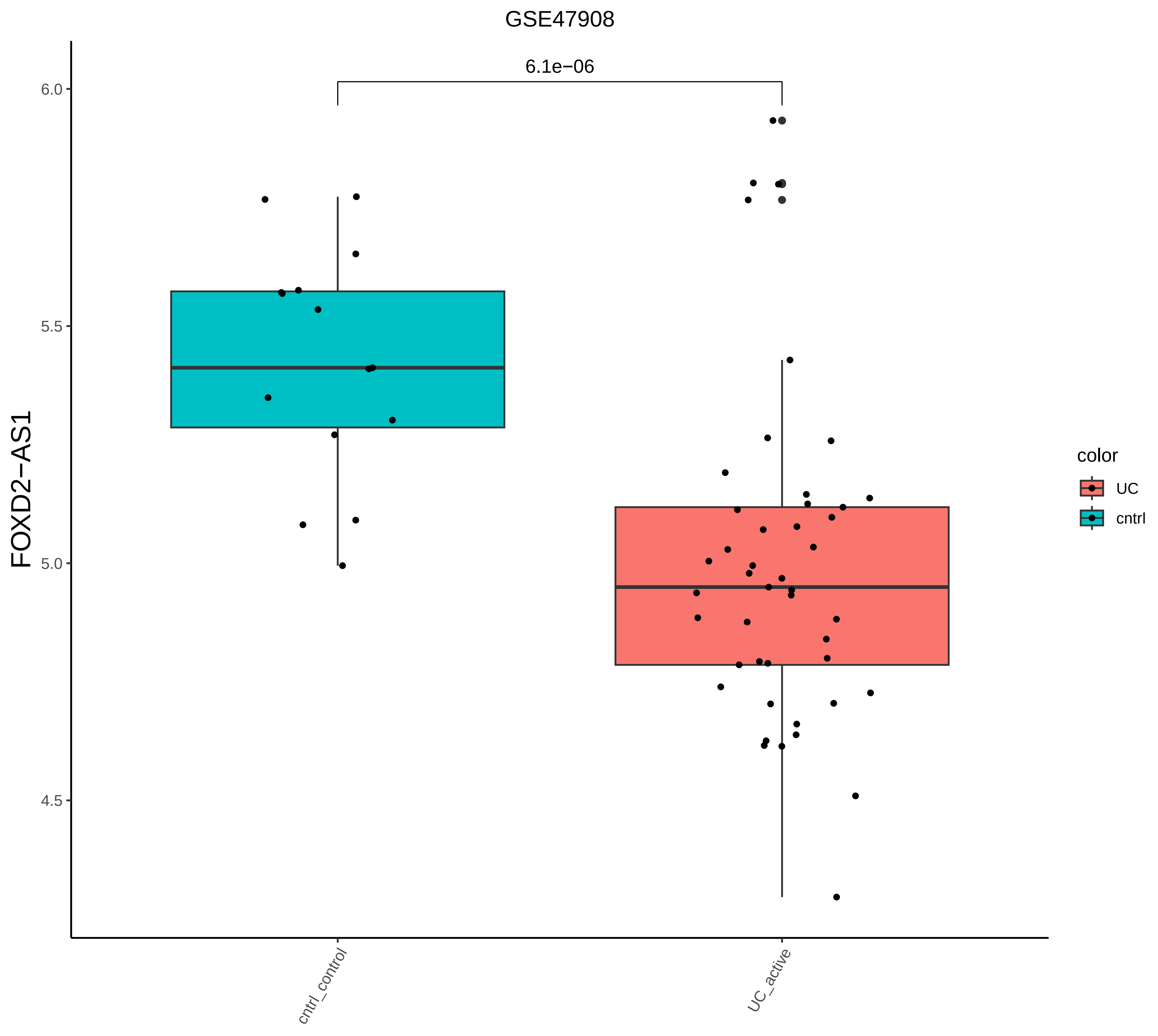
GSE47908
6.1e−06
FOXD2−AS1
6.0
5.5
5.0
4.5
cntrl_control
UC_active
color
UC
cntrl

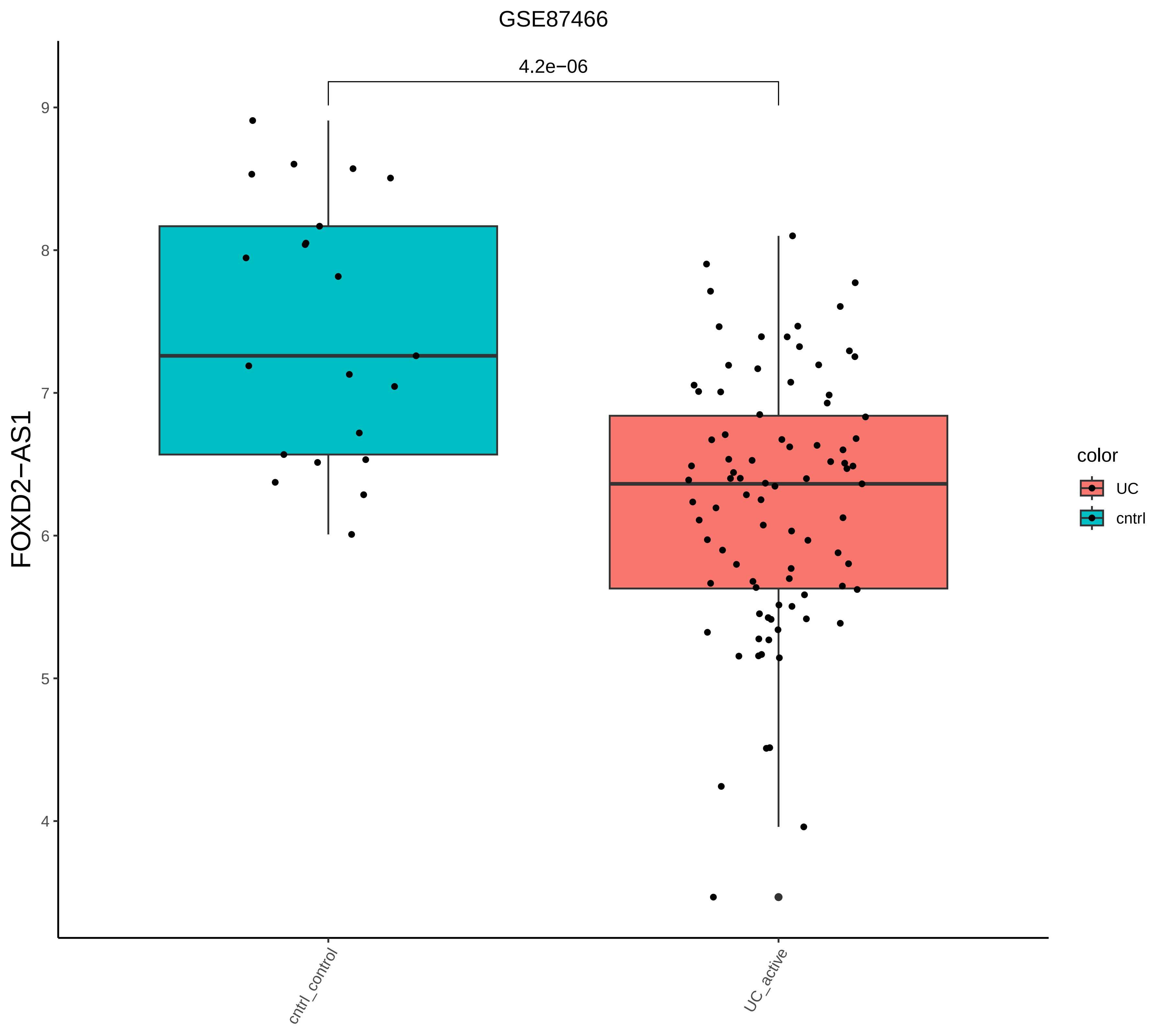
GSE87466
4.2e−06
FOXD2−AS1
9
8
7
6
5
4
cntrl_control
UC_active
color
UC
cntrl

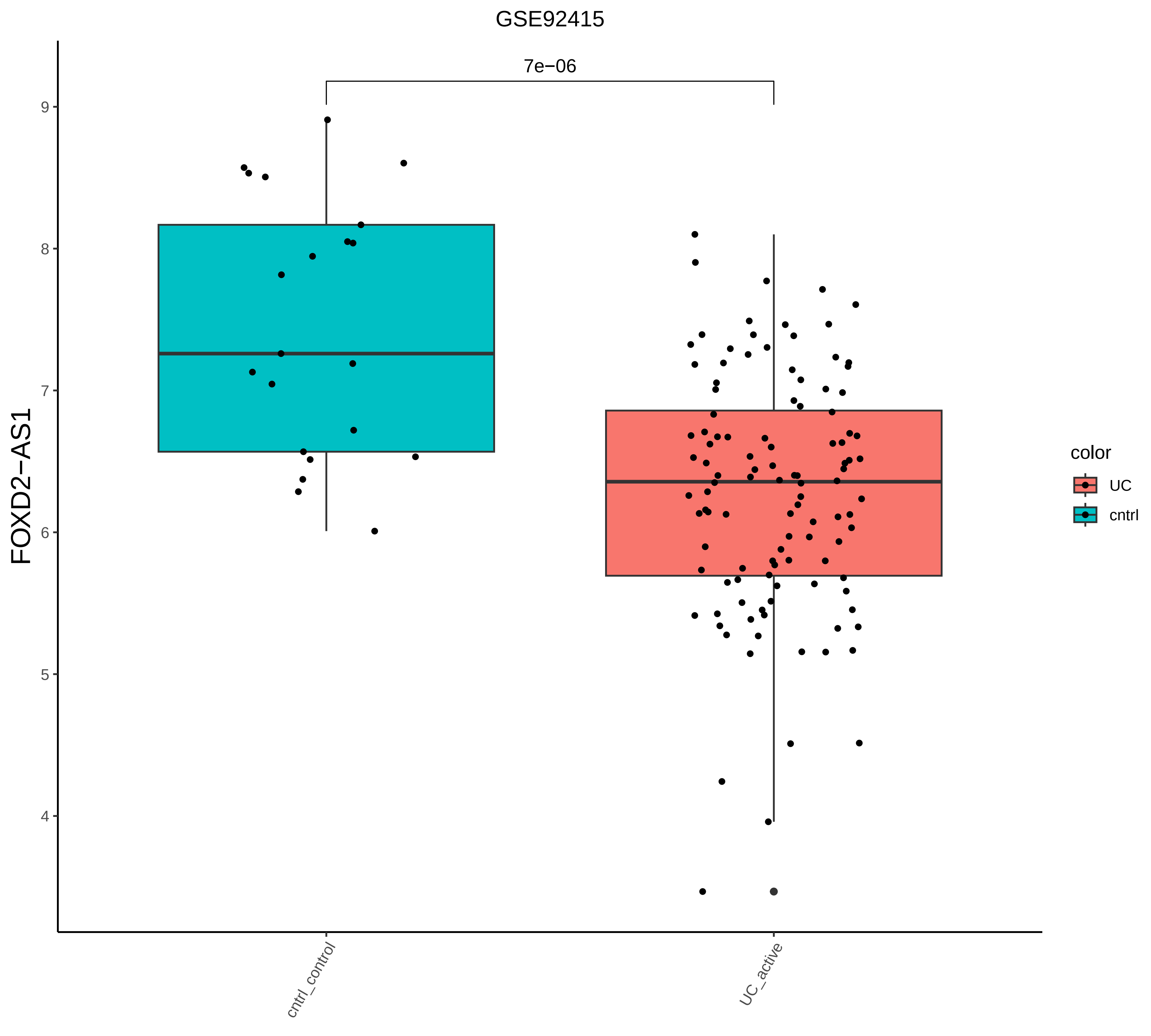
GSE92415
7e−06
FOXD2−AS1
9
8
7
6
5
4
cntrl_control
UC_active
color
UC
cntrl

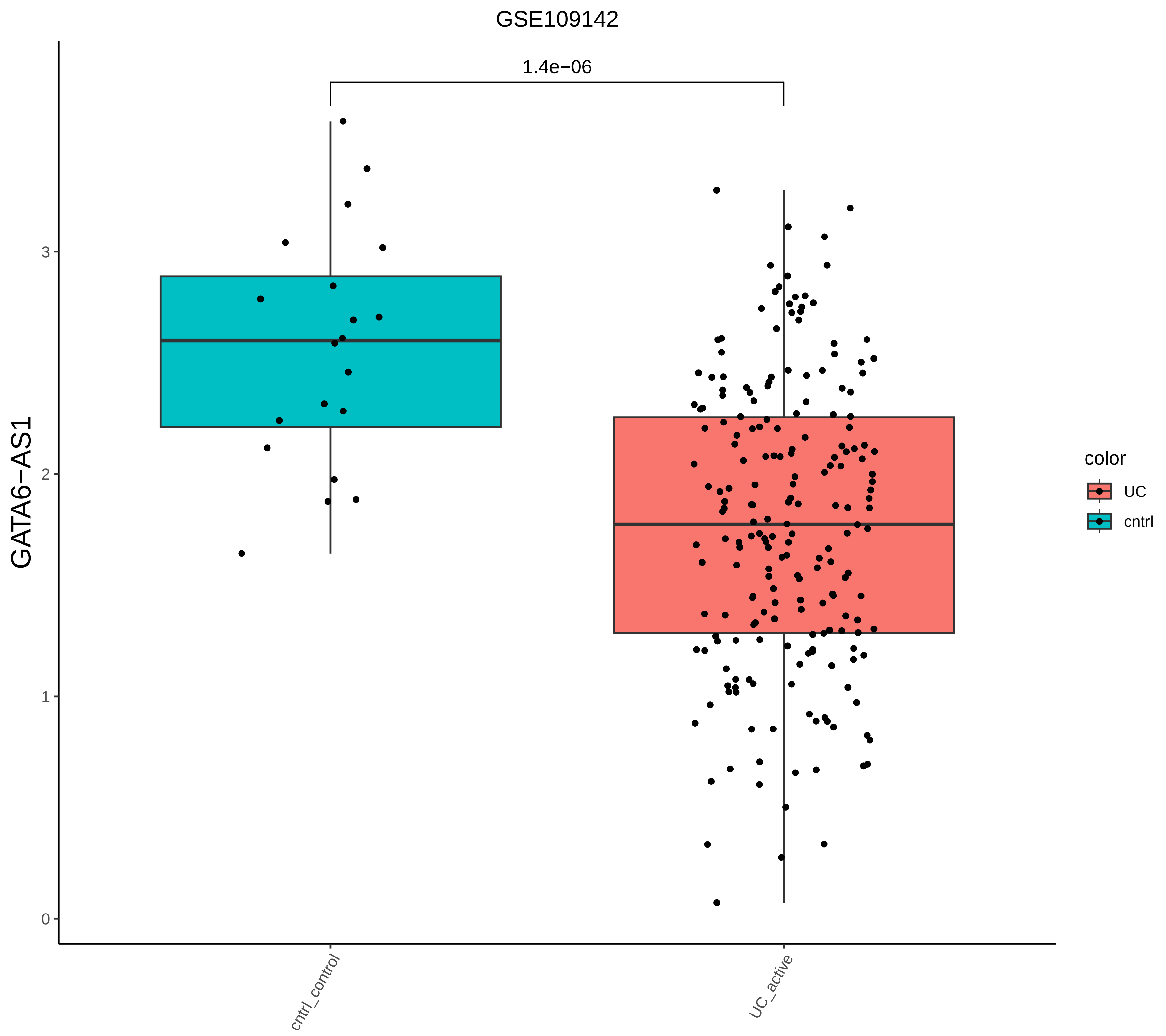
GSE109142
1.4e−06
GATA6−AS1
0
1
2
3
cntrl_control
UC_active
color
UC
cntrl

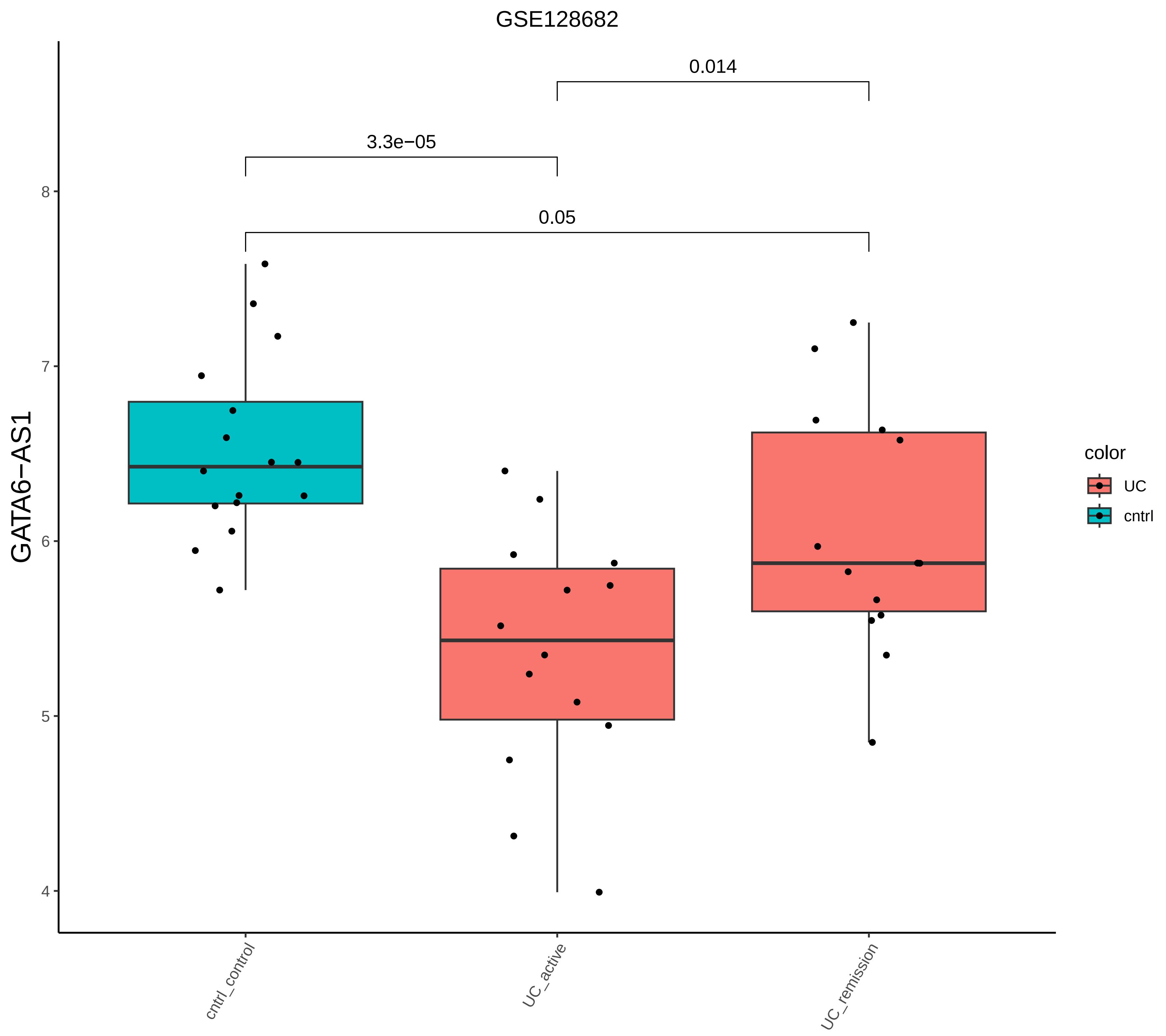

GSE128682
0.014
3.3e−05
0.05
GATA6−AS1
8
7
6
5
4
cntrl_control
UC_active
UC_remission
color
UC
cntrl

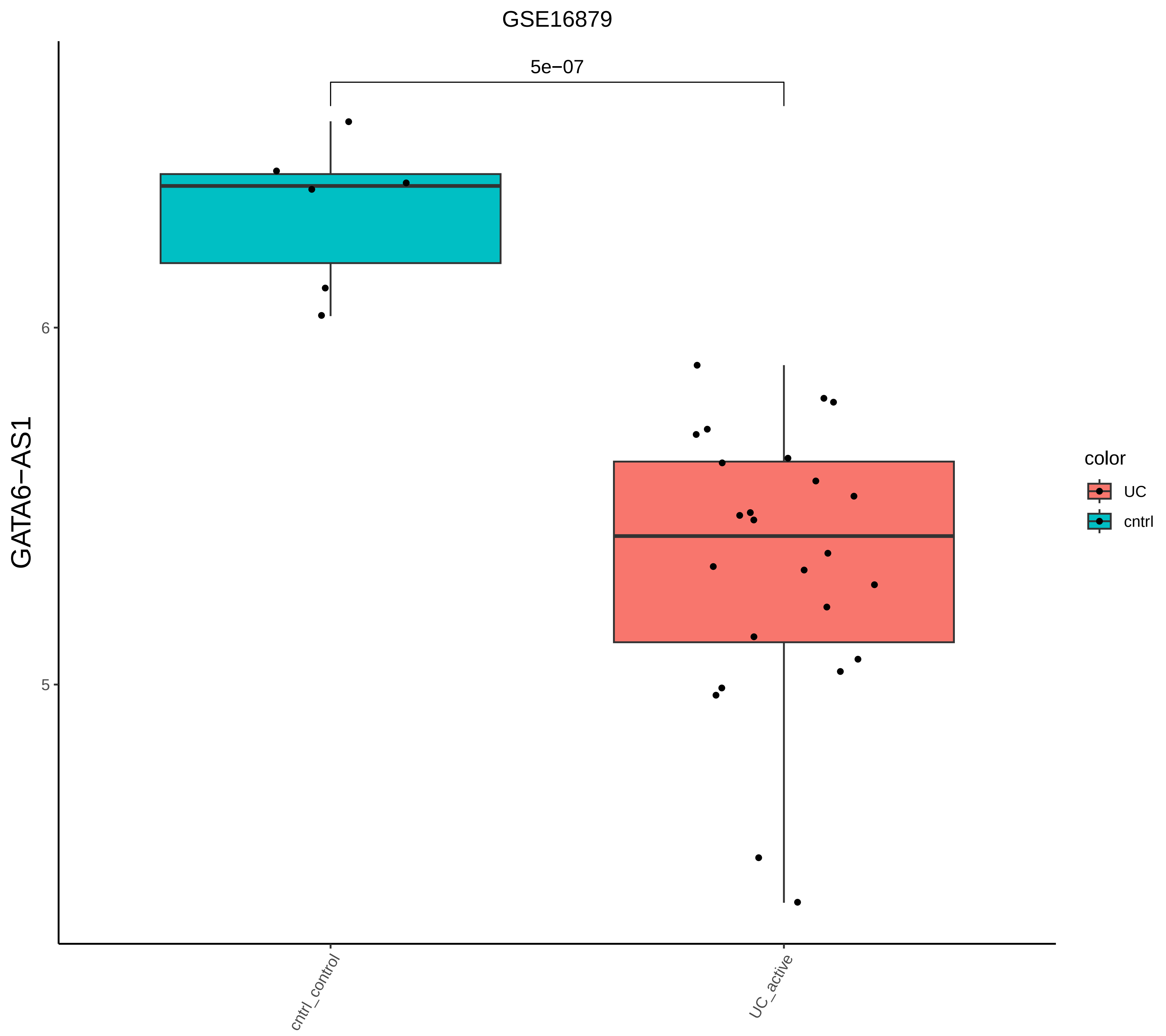

GSE16879
5e−07
GATA6−AS1
6
5
cntrl_control
UC_active
color
UC
cntrl

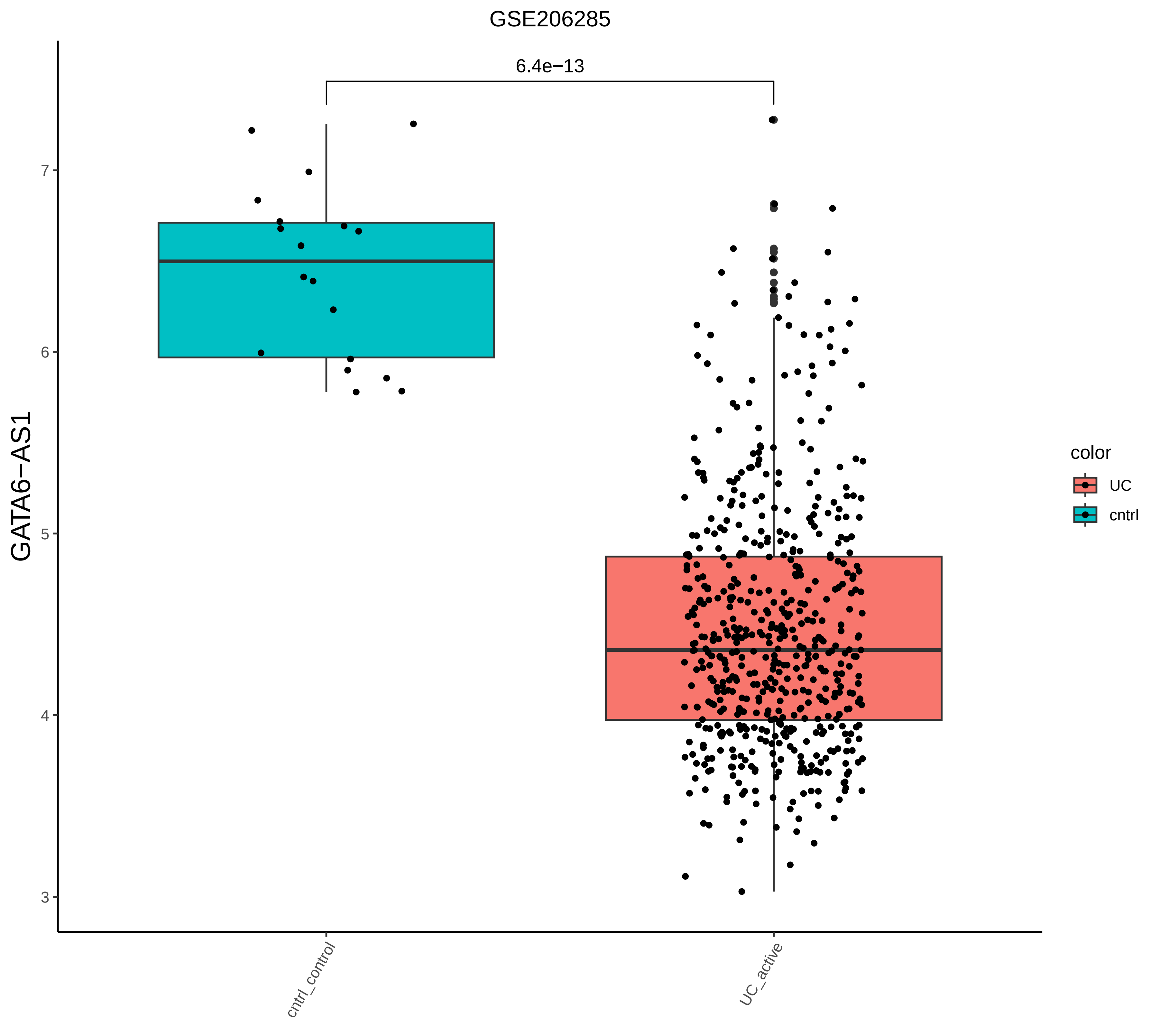

GSE206285
6.4e−13
GATA6−AS1
7
6
5
4
3
cntrl_control
UC_active
color
UC
cntrl

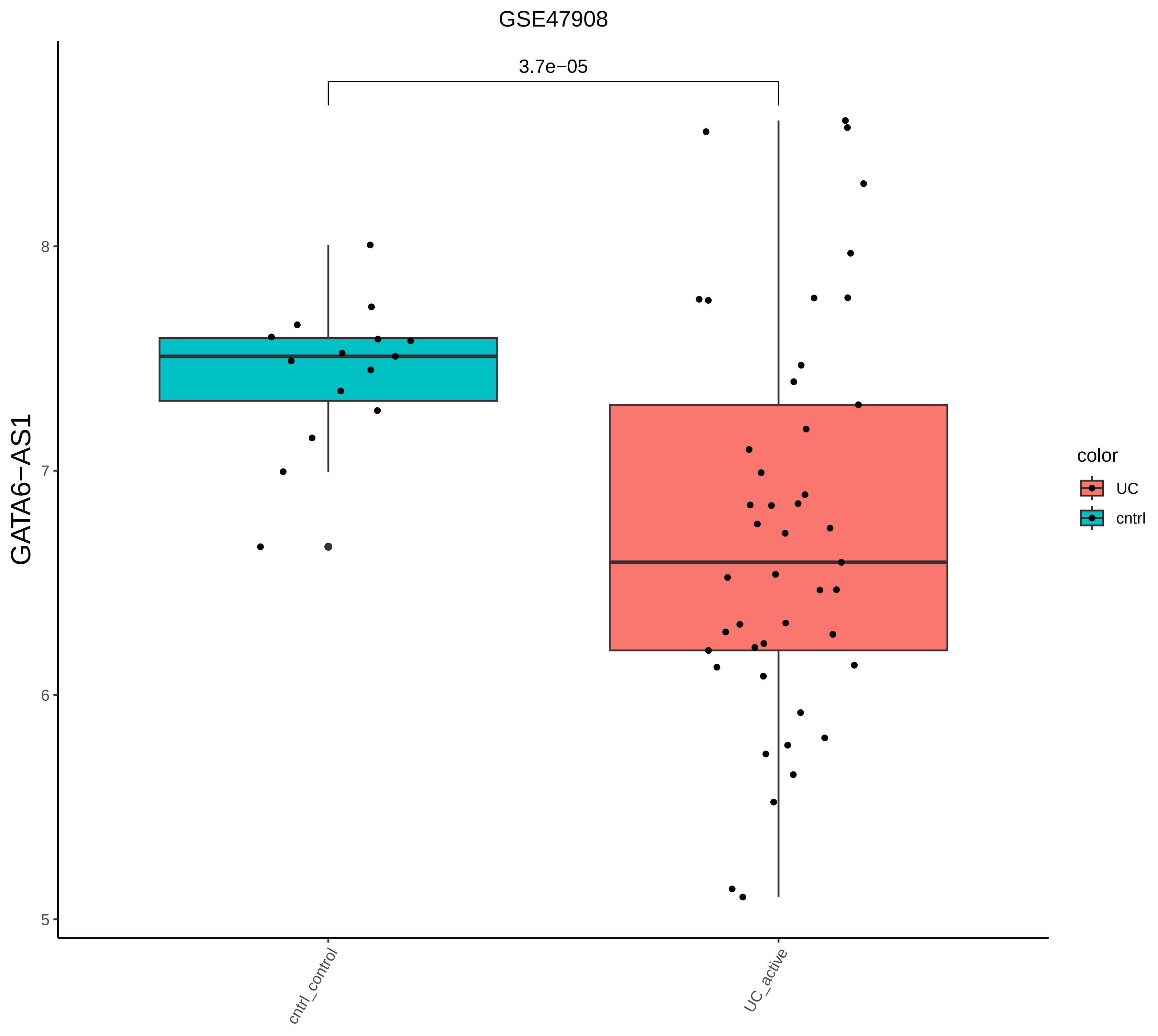

GSE47908
3.7e−05
GATA6−AS1
8
7
6
5
cntrl_control
UC_active
color
UC
cntrl

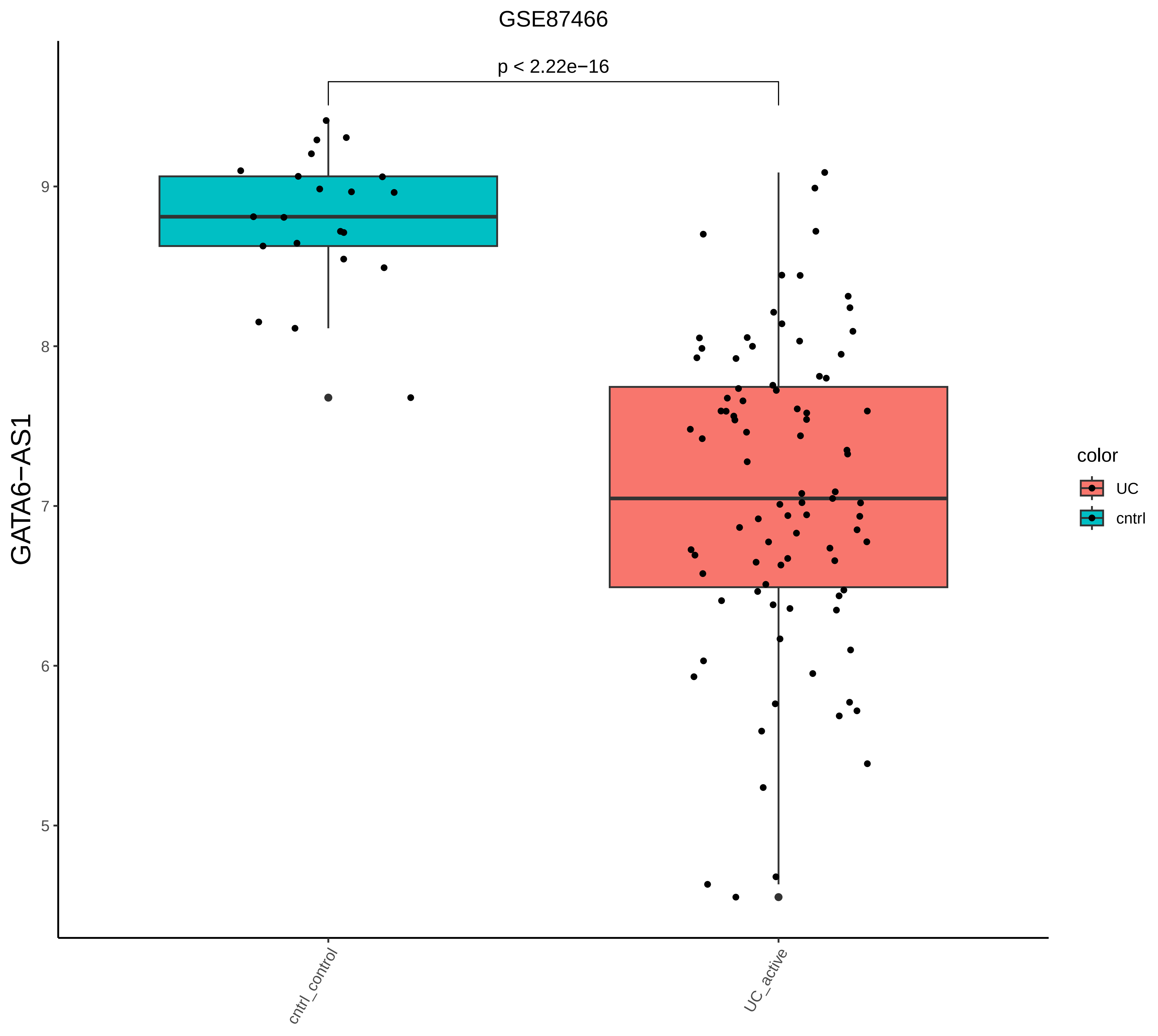

GSE87466
p < 2.22e−16
GATA6−AS1
9
8
7
6
5
cntrl_control
UC_active
color
UC
cntrl

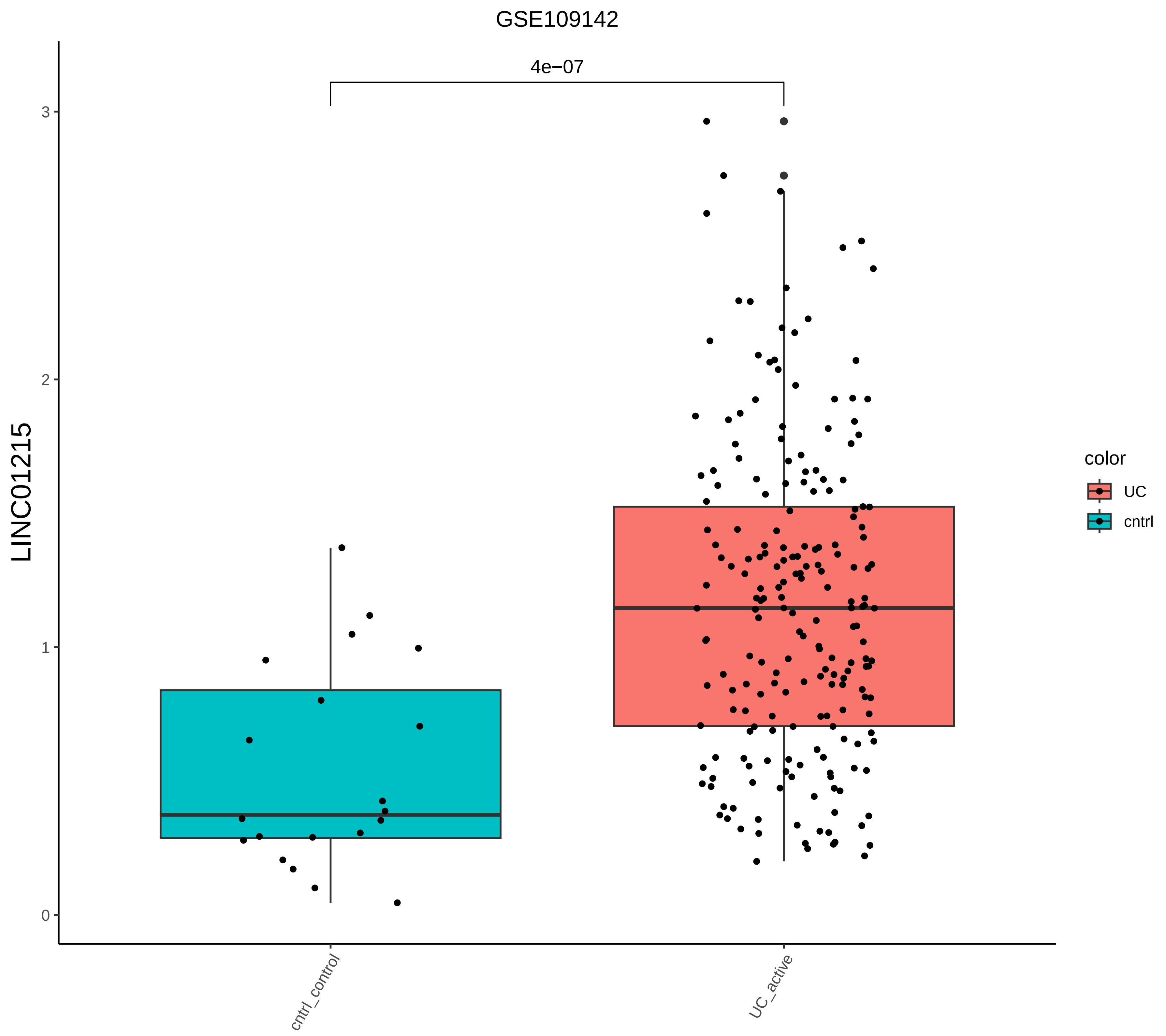
GSE109142
4e−07
LINC01215
0
1
2
3
cntrl_control
UC_active
color
UC
cntrl

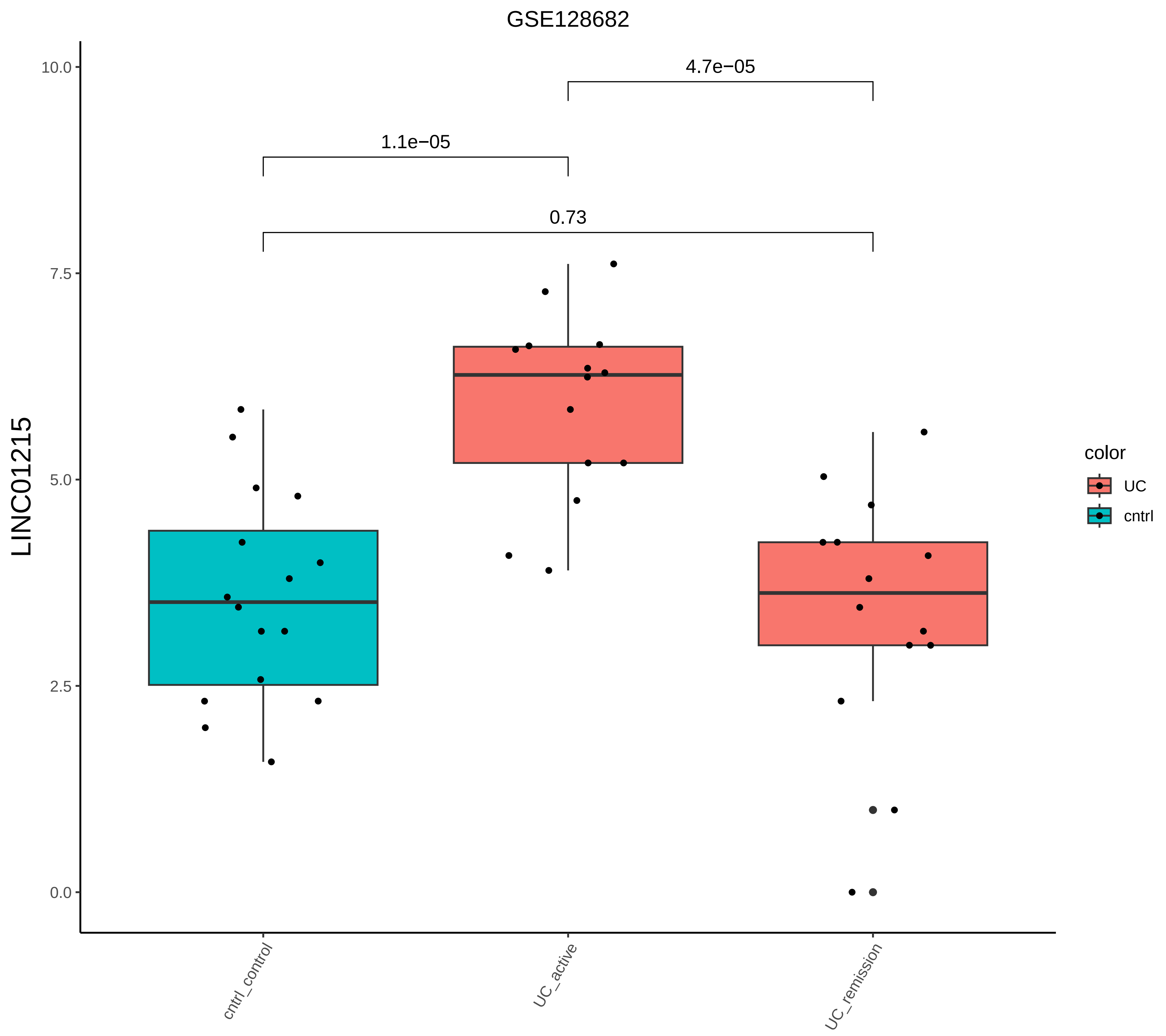
GSE128682
4.7e−05
1.1e−05
0.73
LINC01215
10.0
7.5
5.0
2.5
0.0
cntrl_control
UC_active
UC_remission
color
UC
cntrl

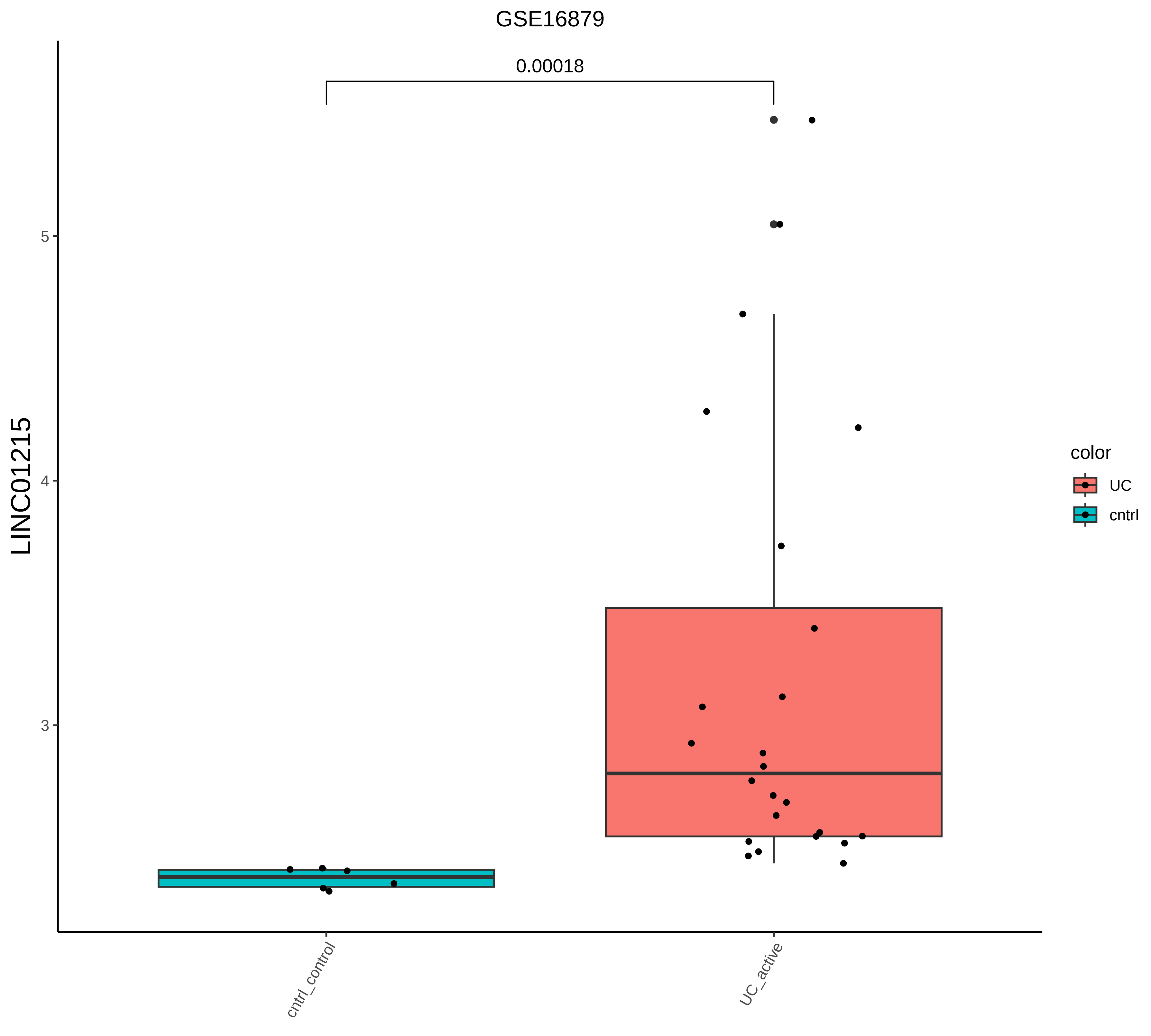

GSE16879
0.00018
LINC01215
5
4
3
cntrl_control
UC_active
color
UC
cntrl

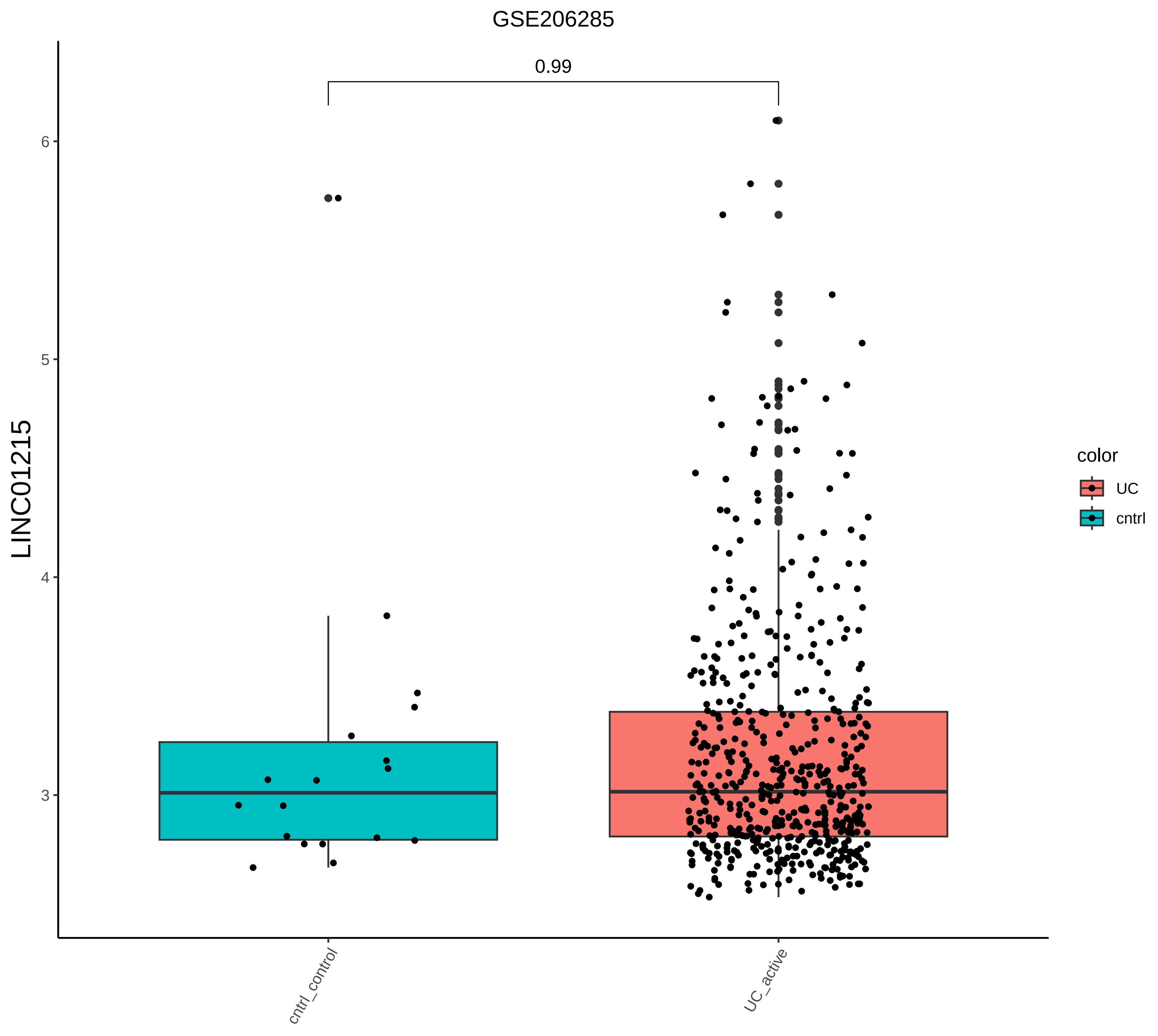

GSE206285
0.99
LINC01215
6
5
4
3
cntrl_control
UC_active
color
UC
cntrl

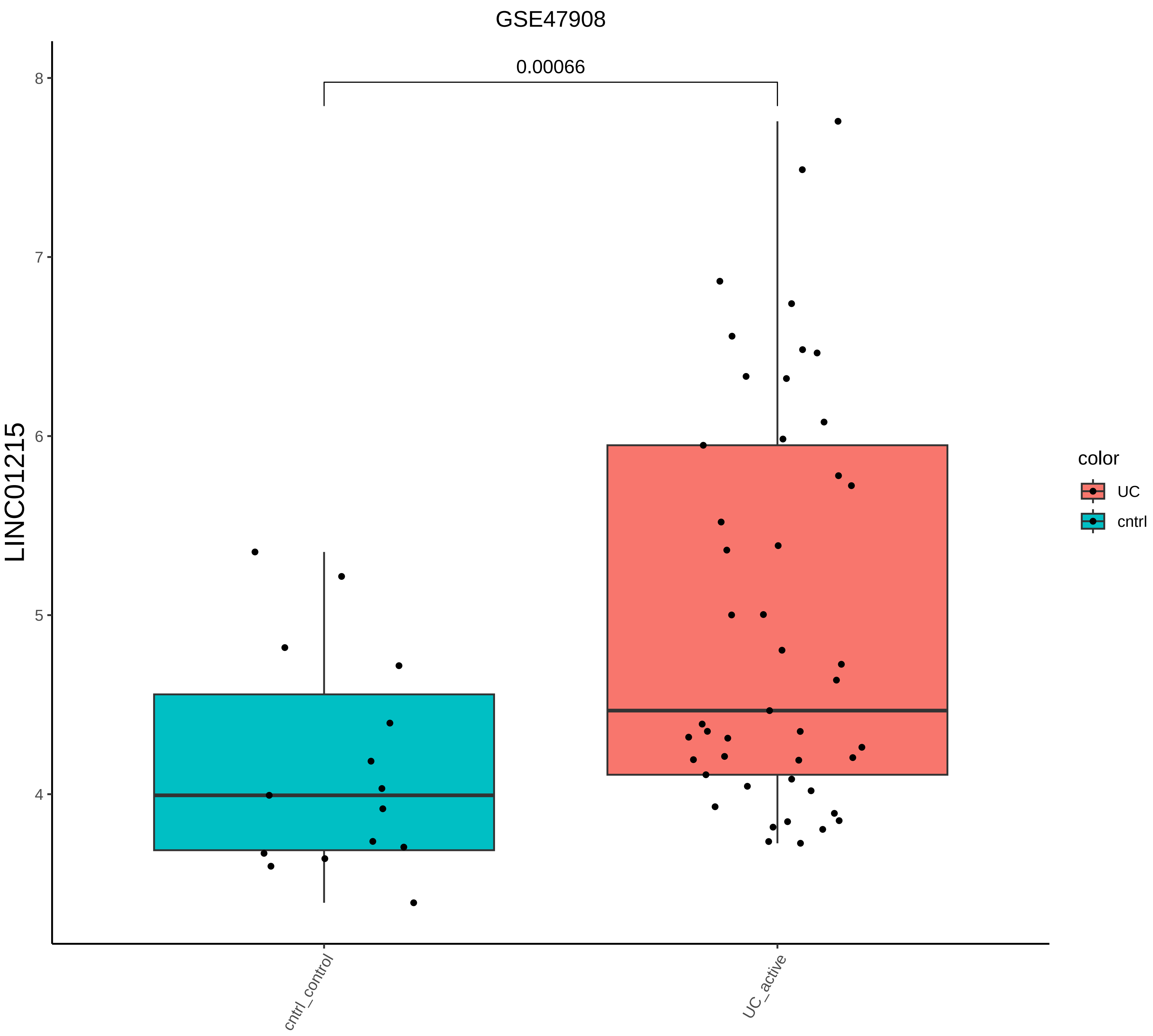
GSE47908
0.00066
LINC01215
8
7
6
5
4
cntrl_control
UC_active
color
UC
cntrl

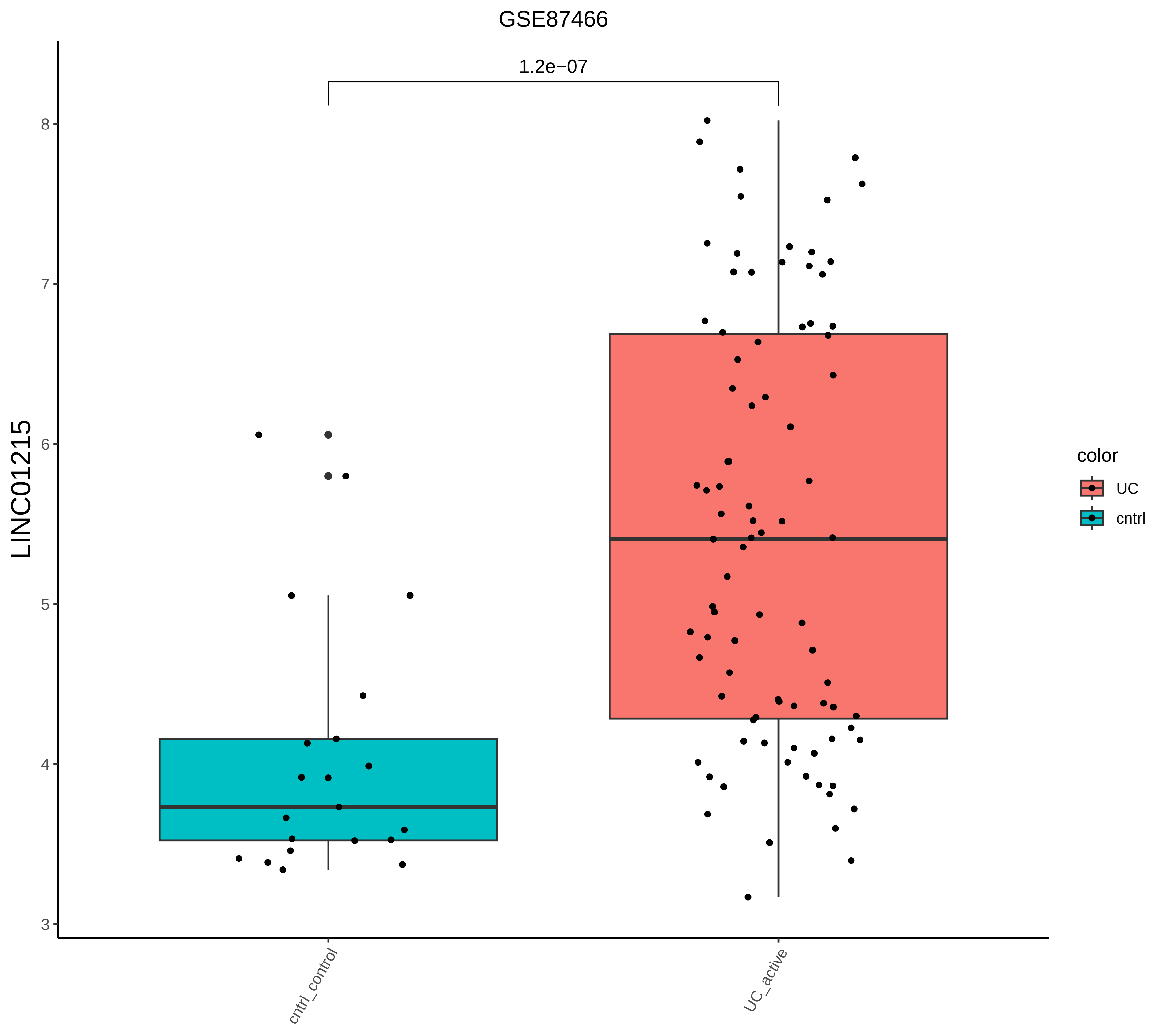

GSE87466
1.2e−07
LINC01215
8
7
6
5
4
3
cntrl_control
UC_active
color
UC
cntrl

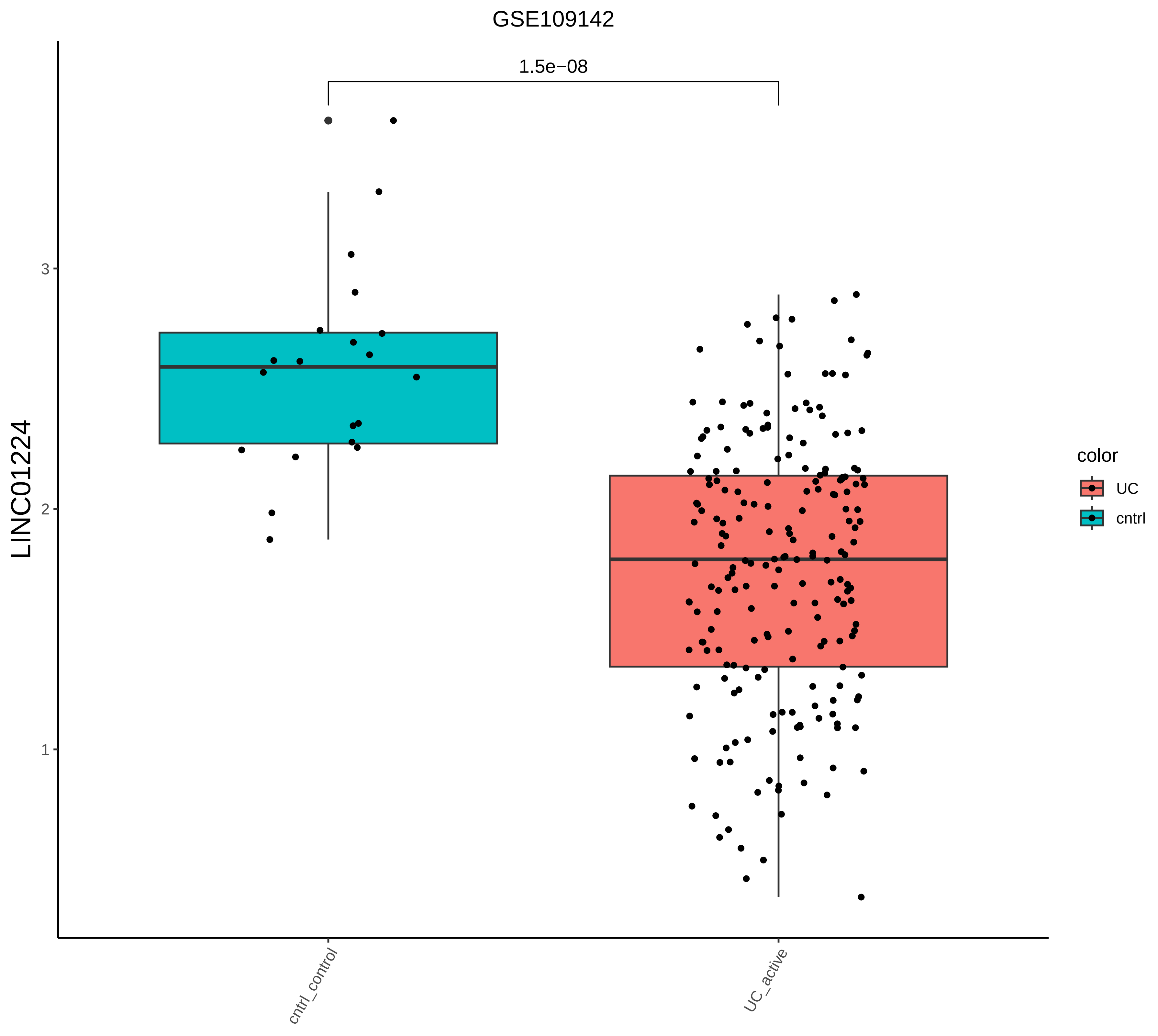
GSE109142
1.5e−08
LINC01224
3
2
1
cntrl_control
UC_active
color
UC
cntrl

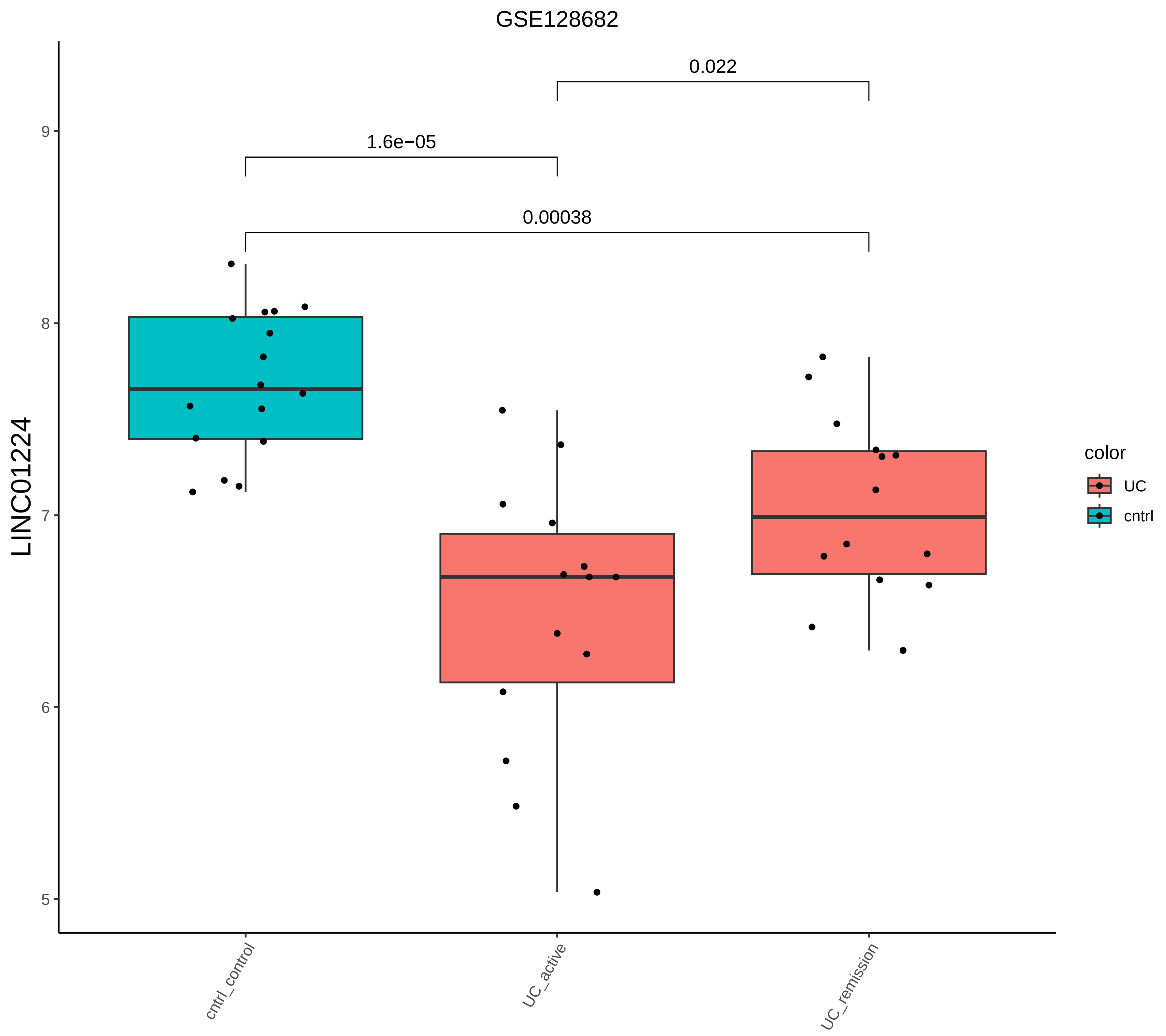

GSE128682
0.022
1.6e−05
0.00038
LINC01224
9
8
7
6
5
cntrl_control
UC_active
UC_remission
color
UC
cntrl

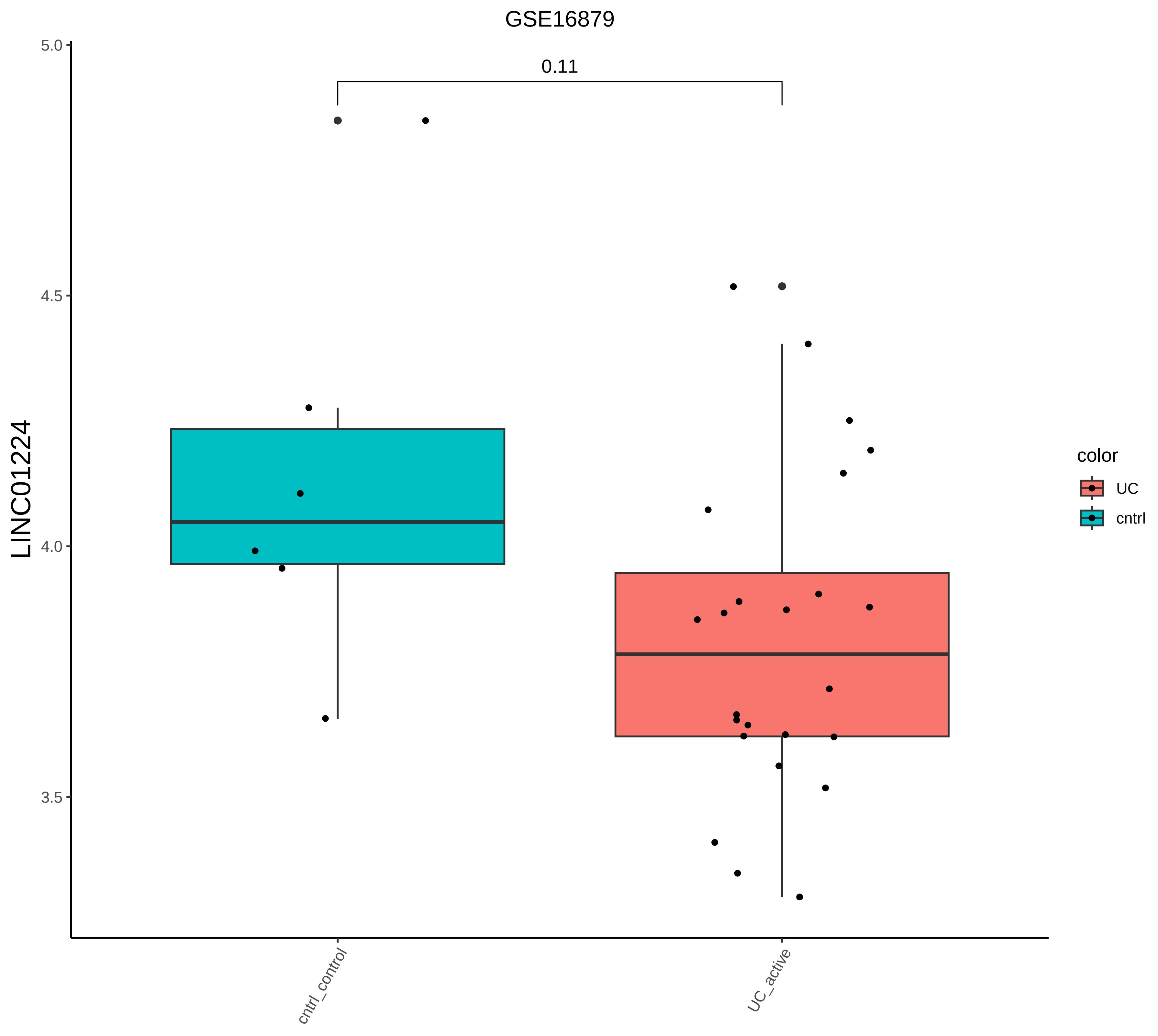
GSE16879
0.11
5.0
4.5
4.0
3.5
LINC01224
cntrl_control
UC_active
color
UC
cntrl

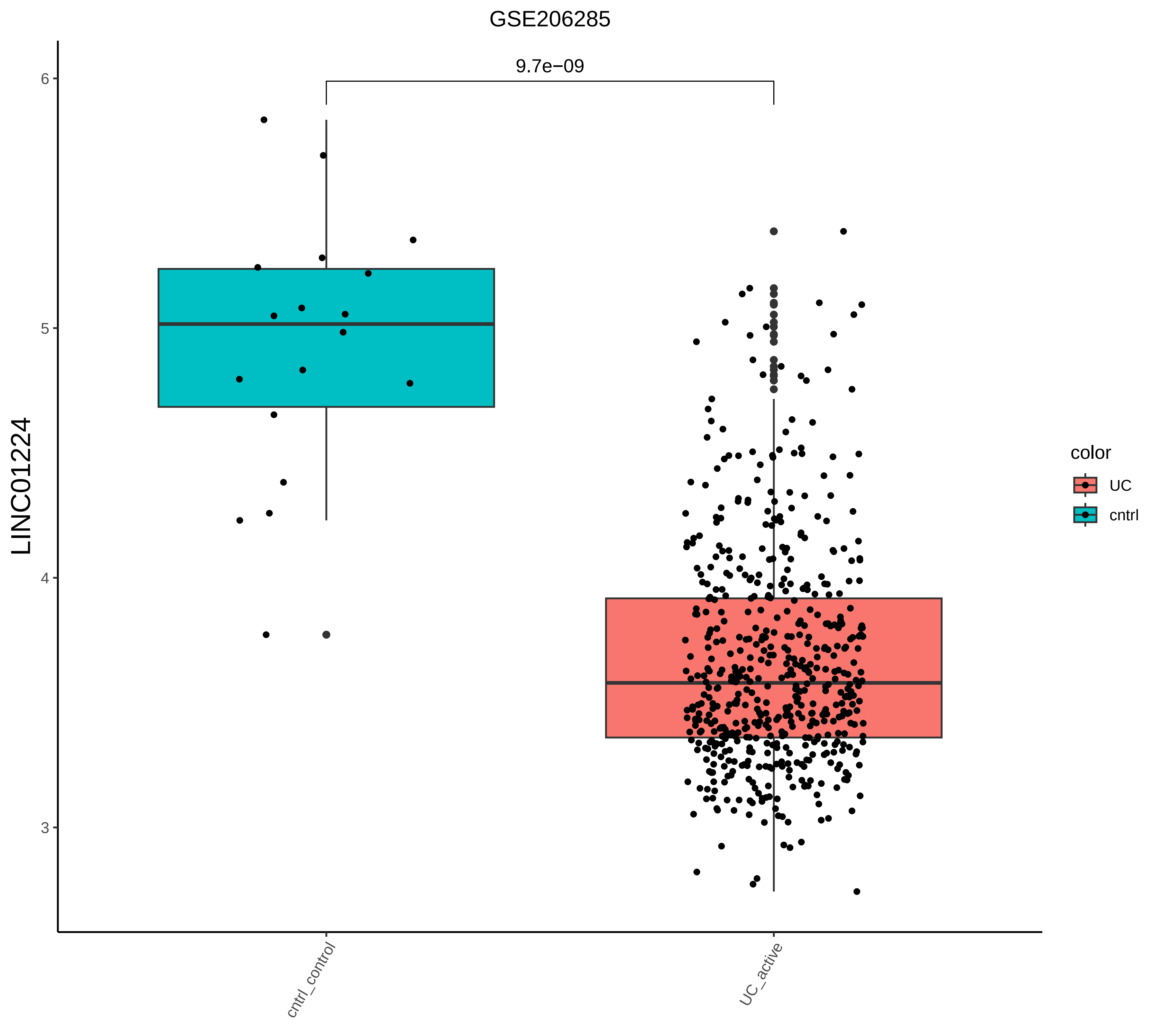

GSE206285
9.7e−09
LINC01224
6
5
4
3
cntrl_control
UC_active
color
UC
cntrl

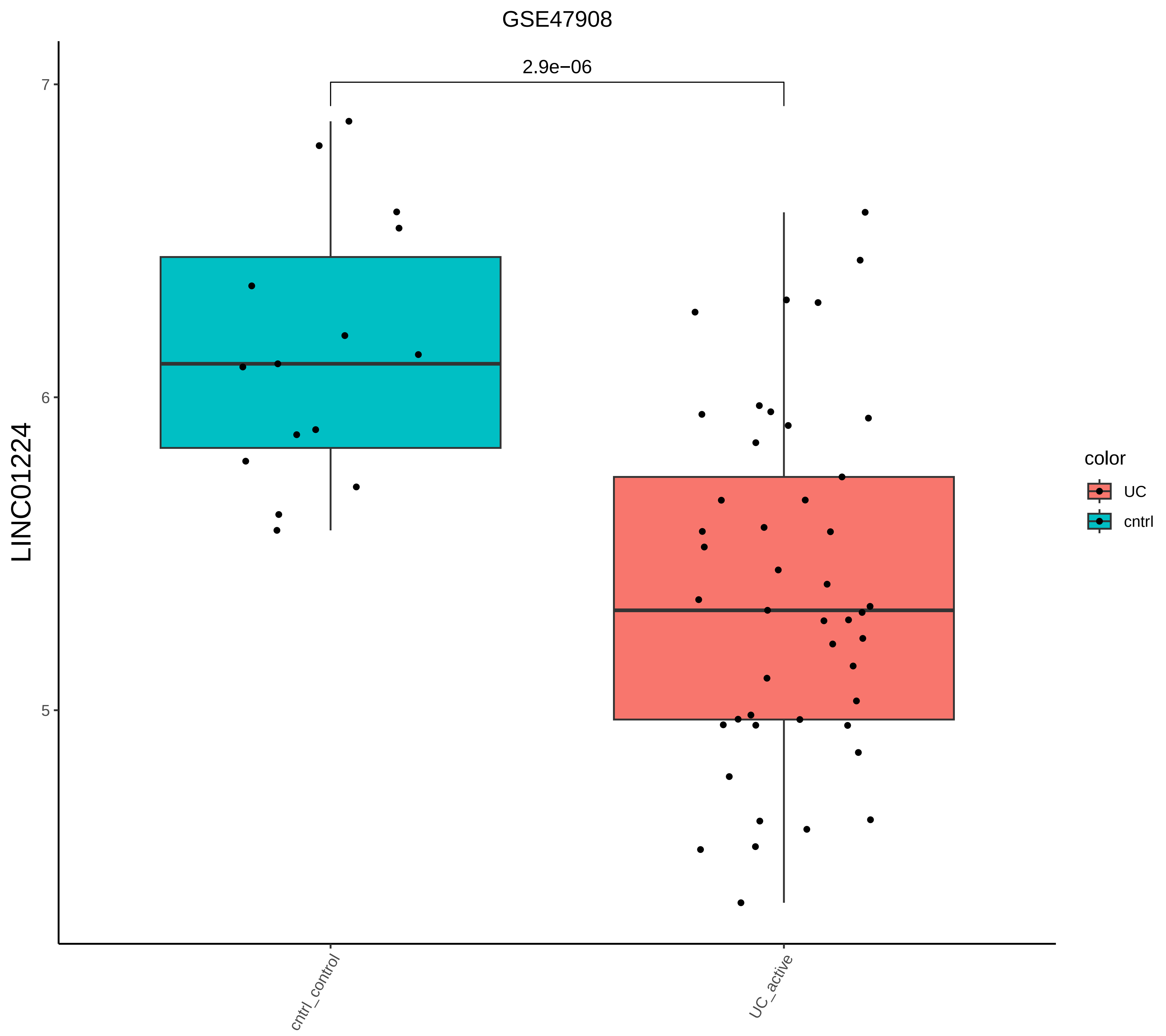
GSE47908
2.9e−06
7
6
5
LINC01224
cntrl_control
UC_active
color
UC
cntrl

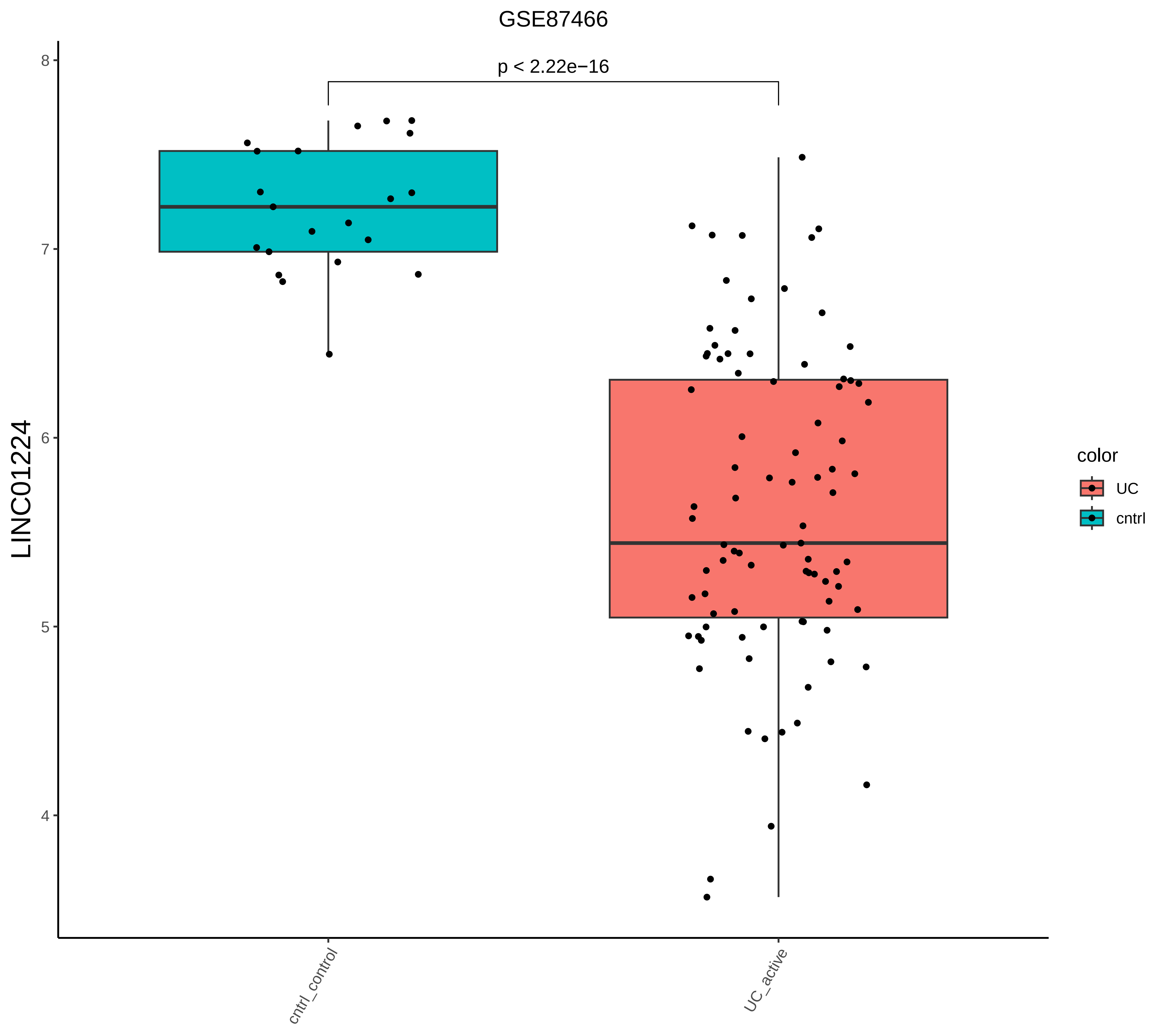

GSE87466
p < 2.22e−16
LINC01224
8
7
6
5
4
cntrl_control
UC_active
color
UC
cntrl

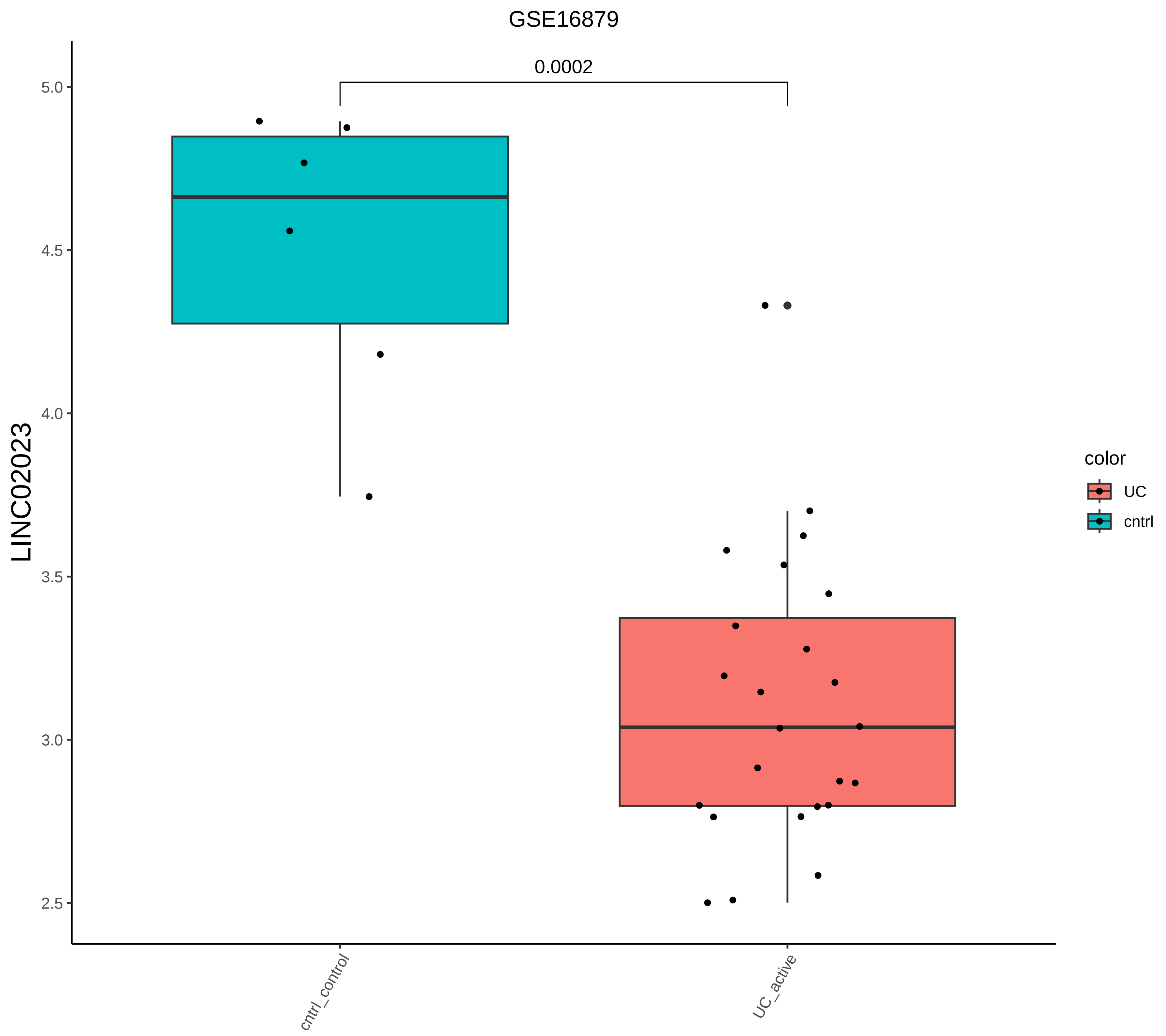
GSE16879
0.0002
LINC02023
5.0
4.5
4.0
3.5
3.0
2.5
cntrl_control
UC_active
color
UC
cntrl

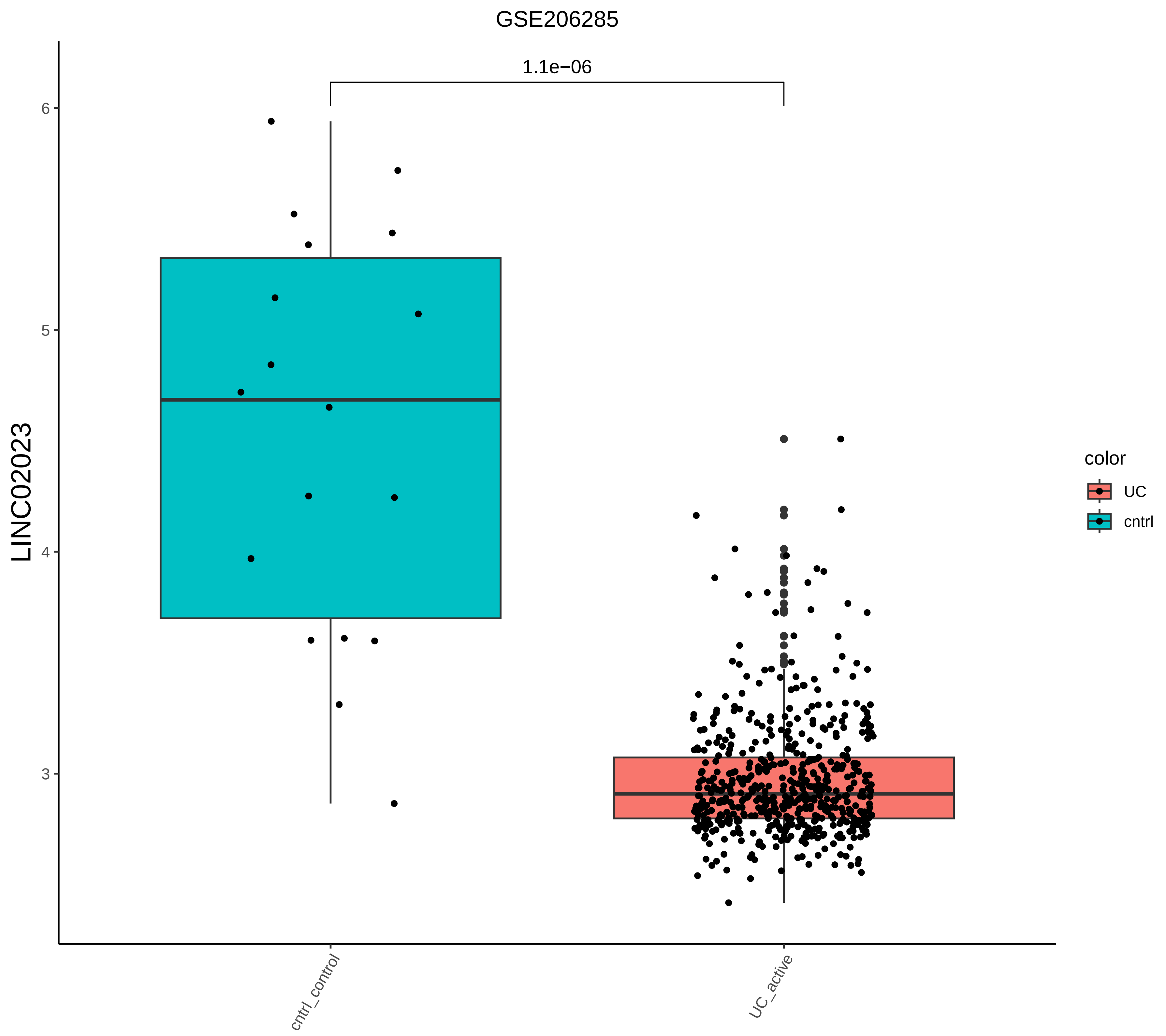

GSE206285
1.1e−06
6
5
4
3
LINC02023
cntrl_control
UC_active
color
UC
cntrl

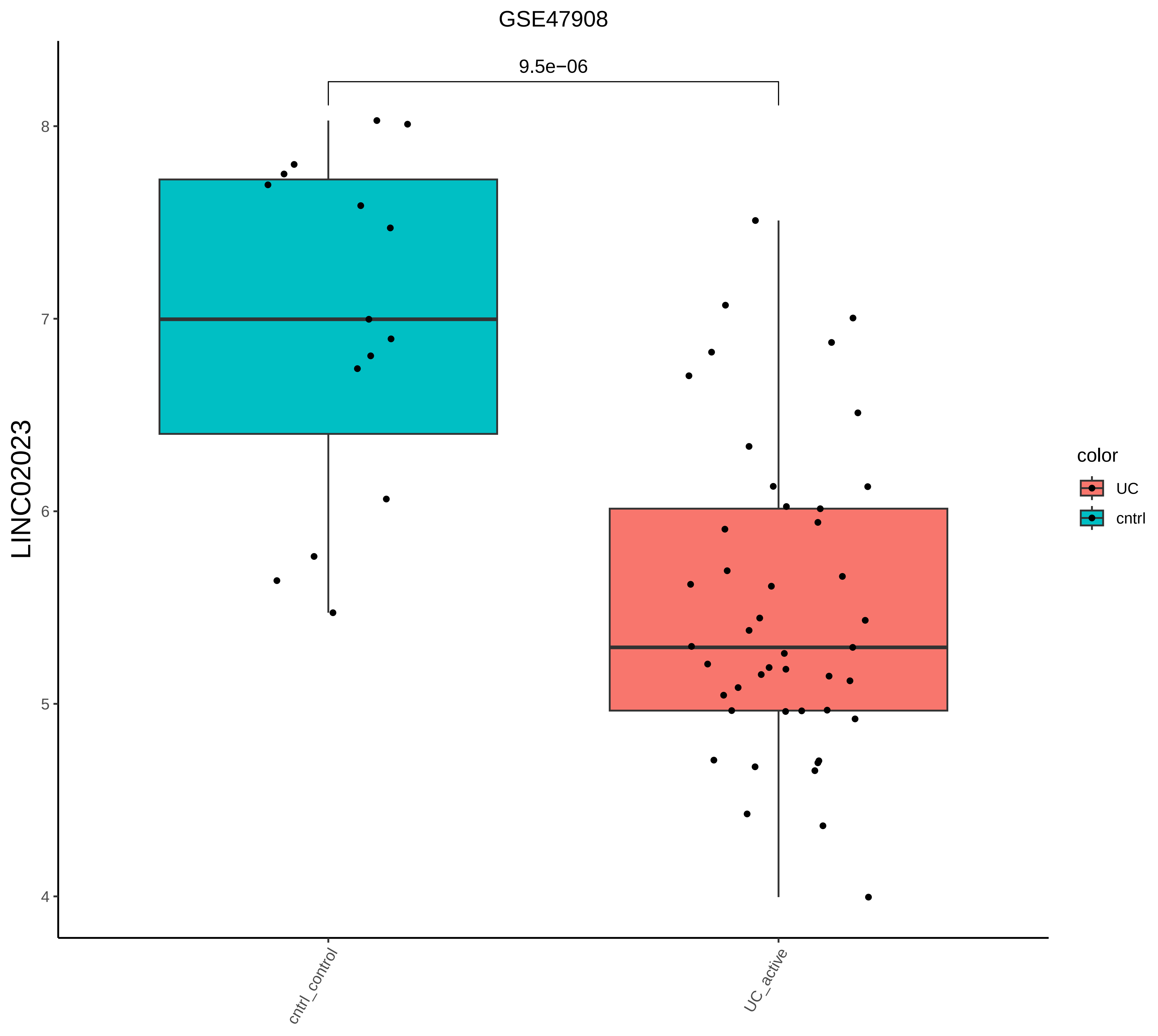
GSE47908
9.5e−06
LINC02023
8
7
6
5
4
cntrl_control
UC_active
color
UC
cntrl

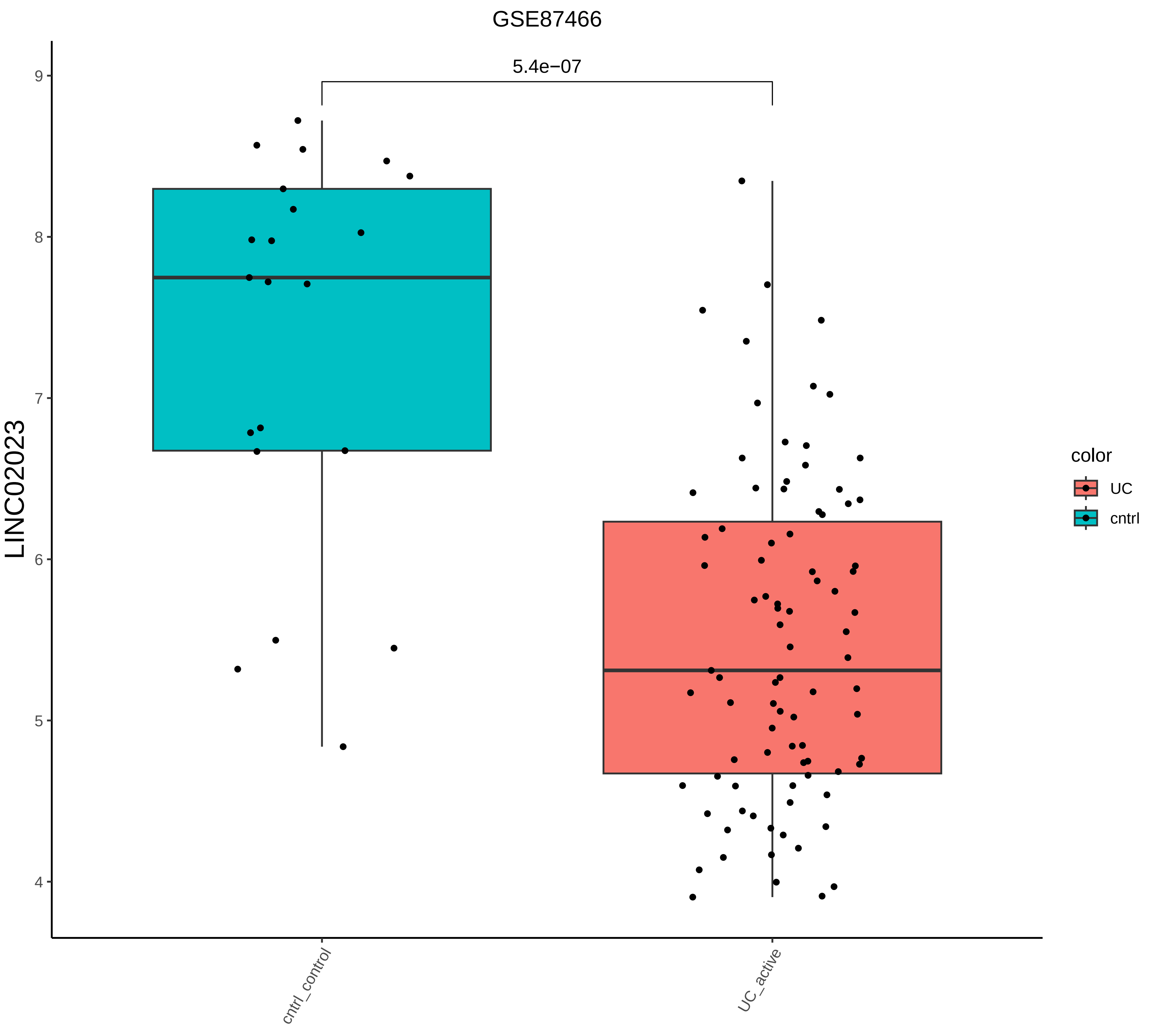
GSE87466
5.4e−07
LINC02023
9
8
7
6
5
4
cntrl_control
UC_active
color
UC
cntrl

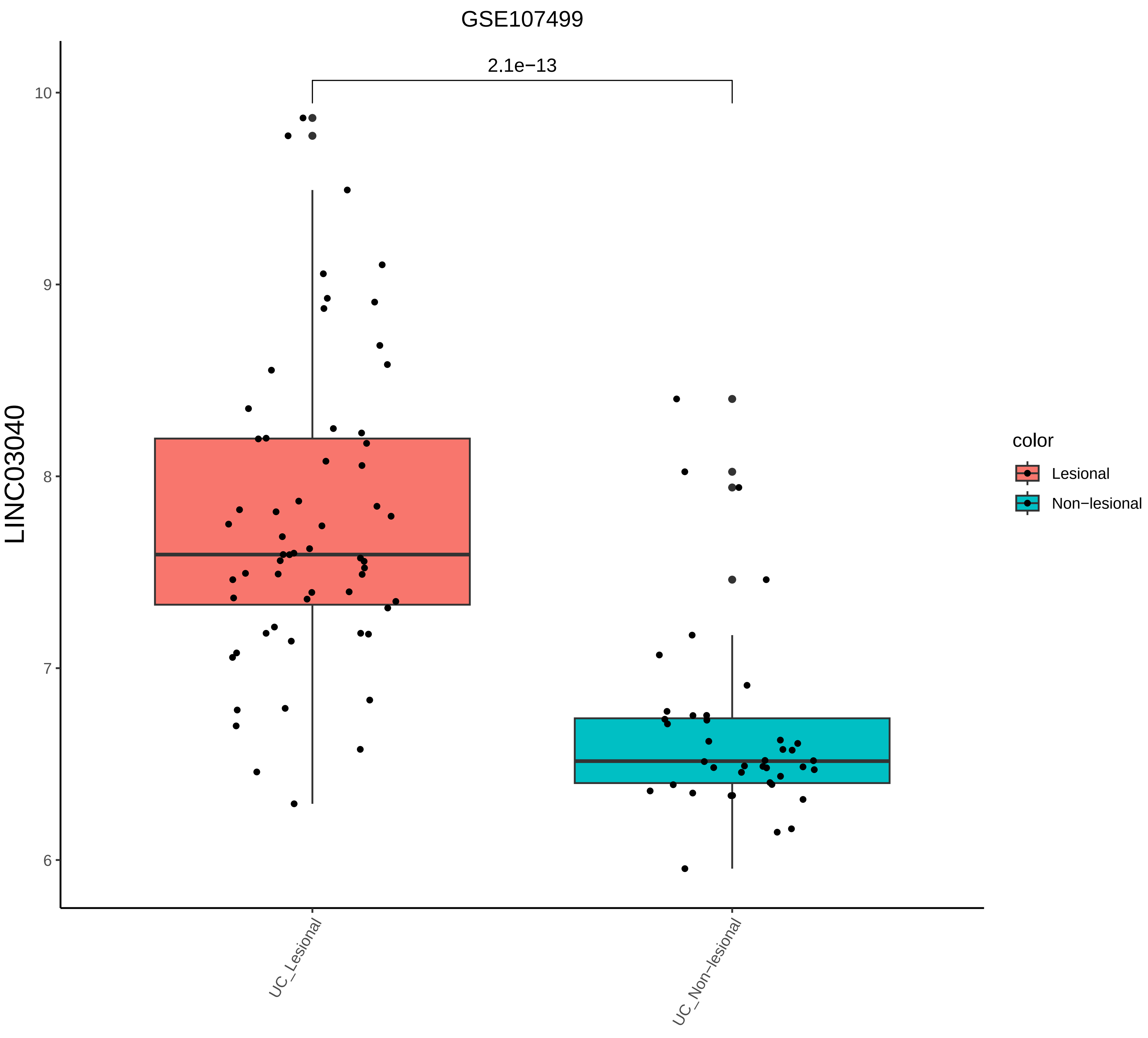

GSE107499
2.1e−13
LINC03040
10
9
8
7
6
UC_Lesional
UC_Non−lesional
color
Lesional
Non−lesional

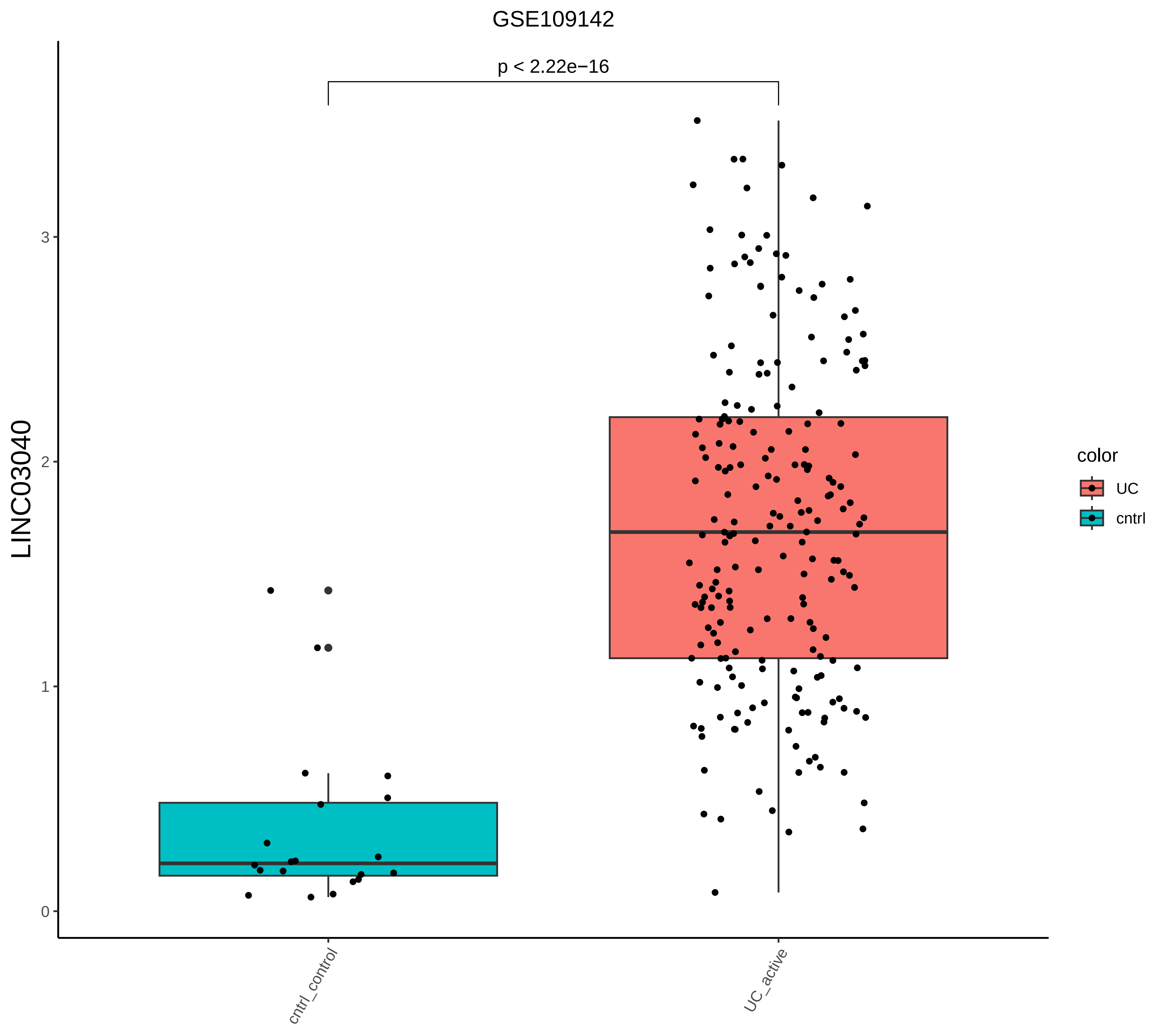

GSE109142
p < 2.22e−16
LINC03040
0
1
2
3
cntrl_control
UC_active
color
UC
cntrl

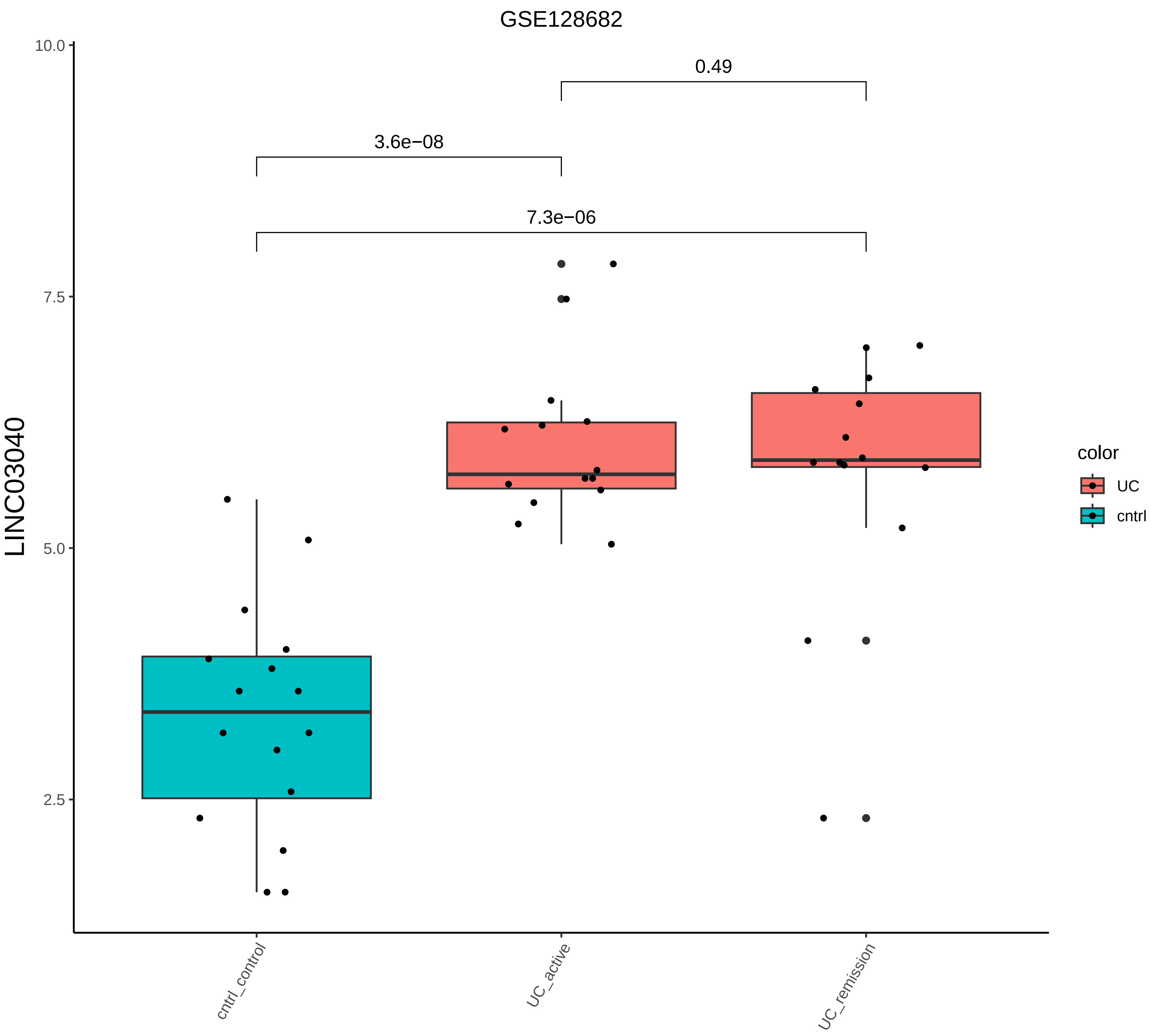

GSE128682
0.49
3.6e−08
7.3e−06
10.0
7.5
5.0
2.5
LINC03040
cntrl_control
UC_active
UC_remission
color
UC
cntrl

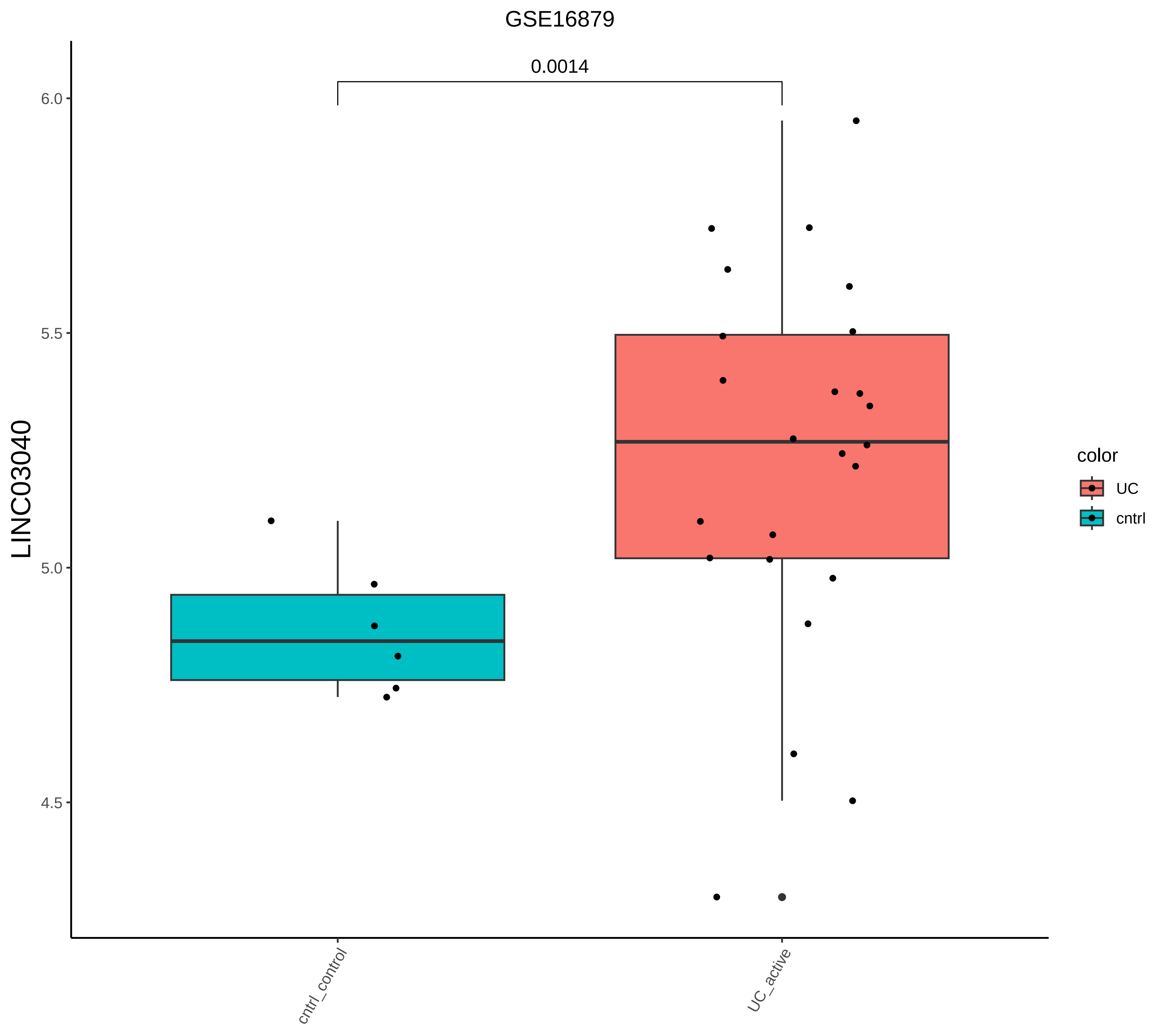
GSE16879
0.0014
LINC03040
6.0
5.5
5.0
4.5
cntrl_control
UC_active
color
UC
cntrl

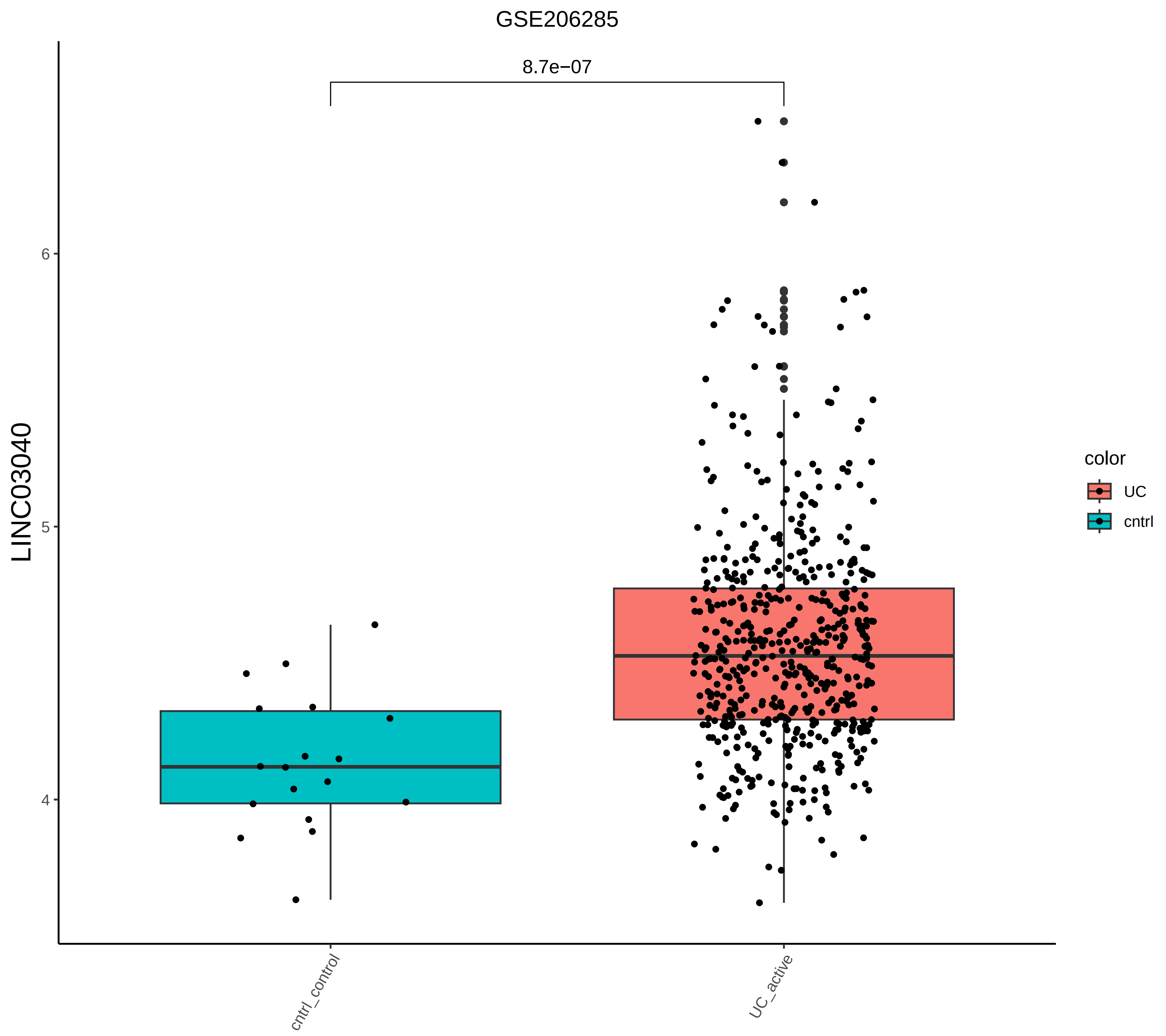
GSE206285
8.7e−07
LINC03040
6
5
4
cntrl_control
UC_active
color
UC
cntrl

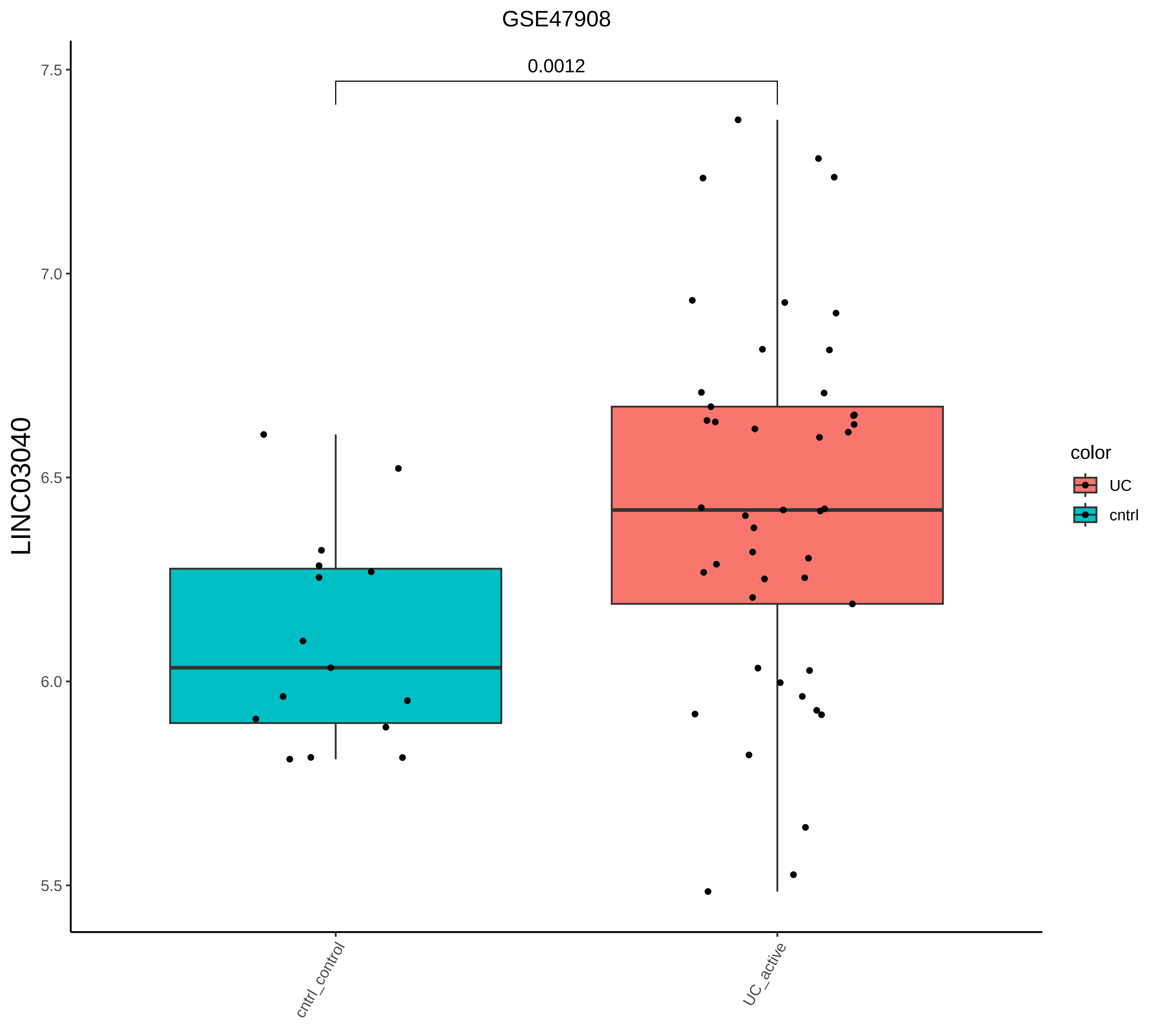

GSE47908
0.0012
7.5
7.0
6.5
6.0
5.5
LINC03040
cntrl_control
UC_active
color
UC
cntrl

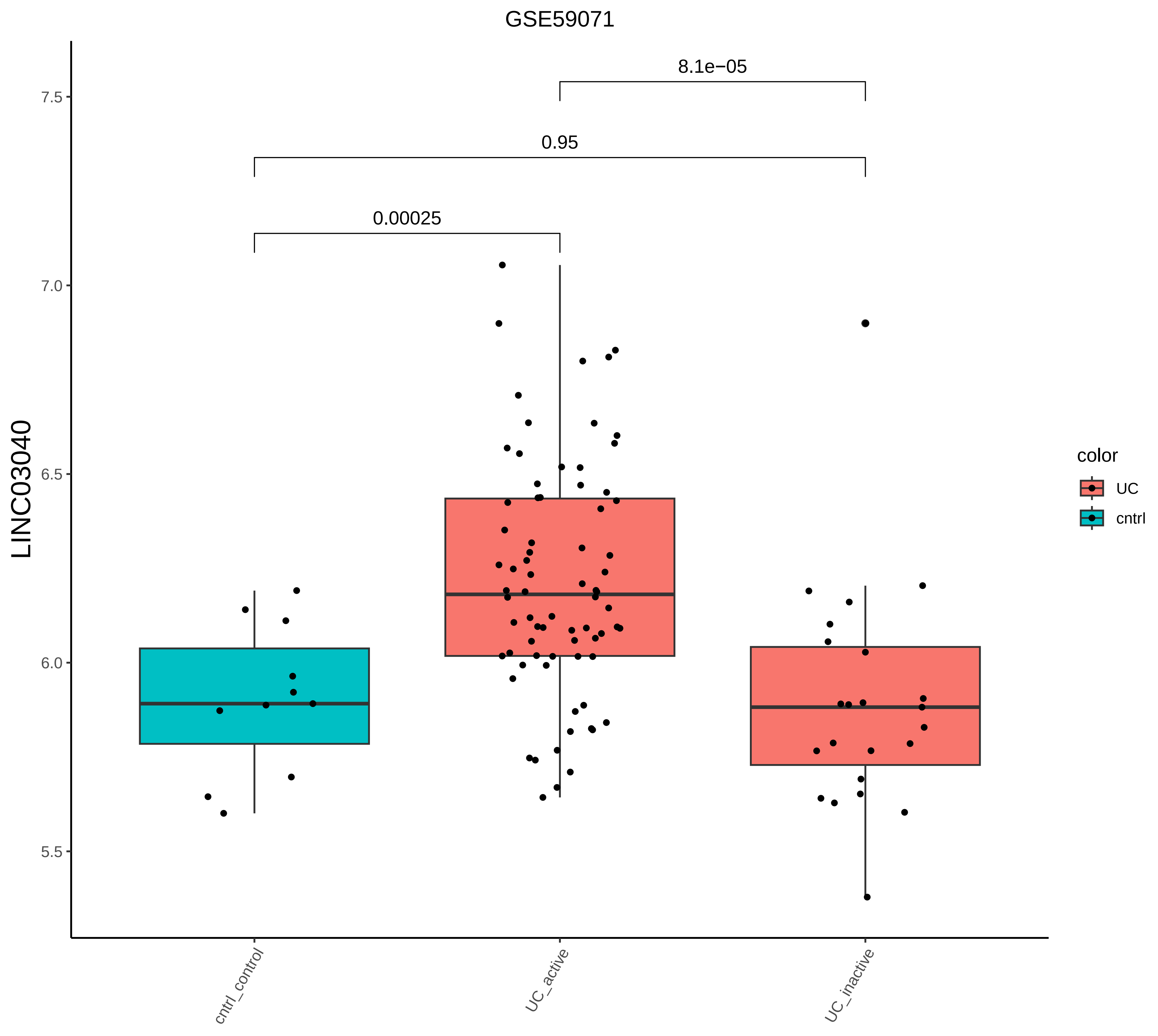
GSE59071
8.1e−05
0.95
0.00025
LINC03040
7.5
7.0
6.5
6.0
5.5
cntrl_control
UC_active
UC_inactive
color
UC
cntrl

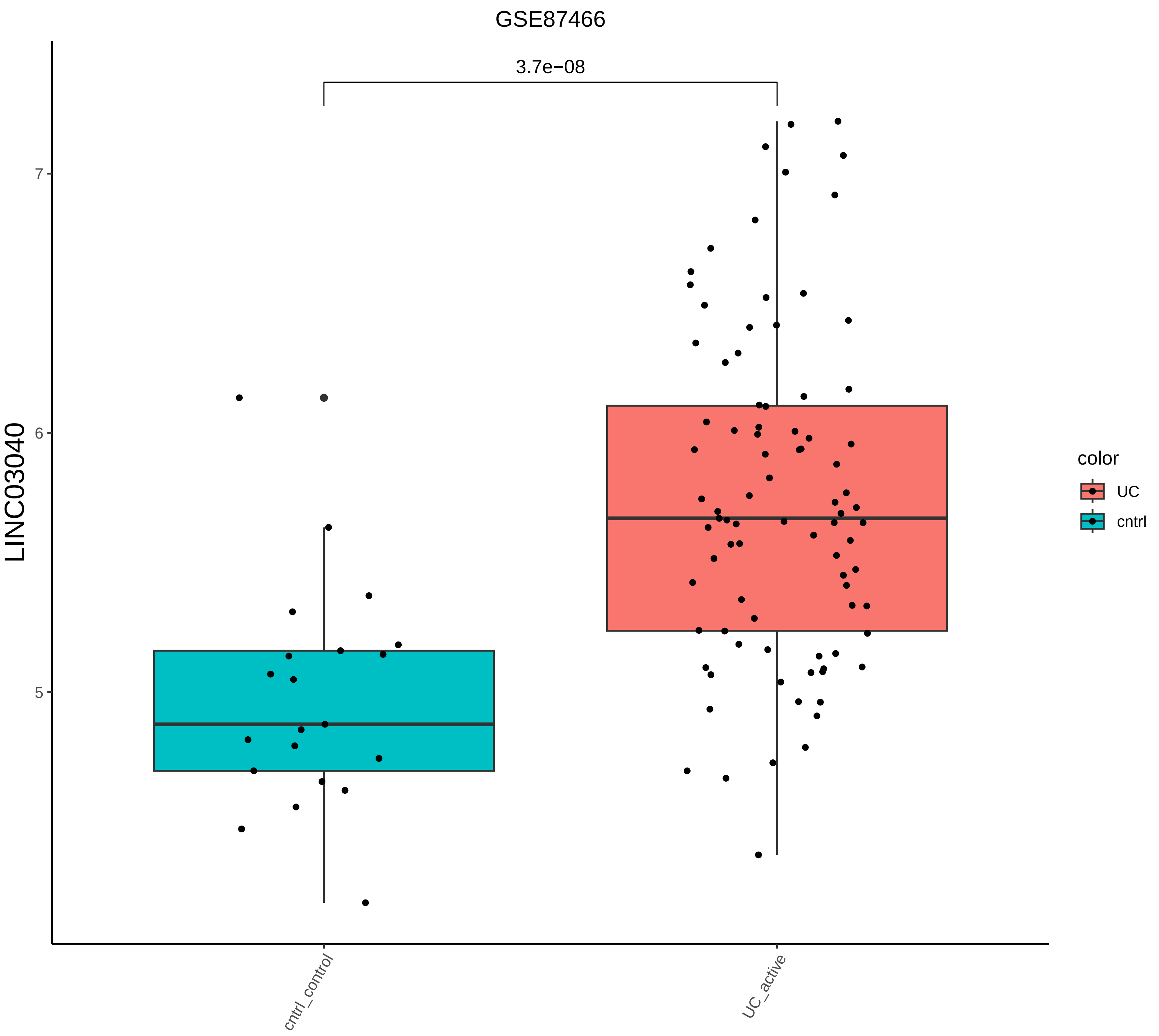

GSE87466
3.7e−08
LINC03040
7
6
5
cntrl_control
UC_active
color
UC
cntrl

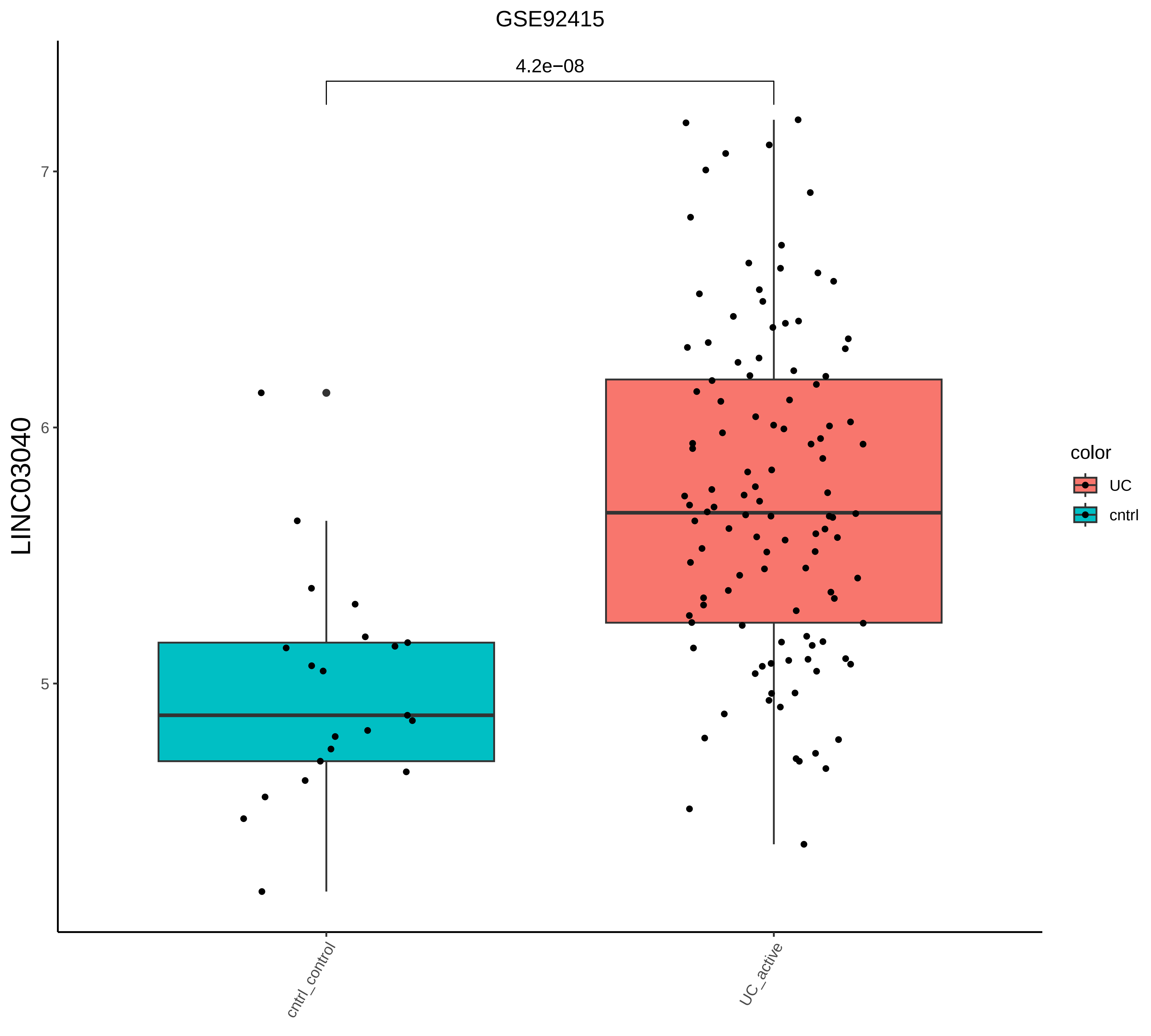

GSE92415
4.2e−08
LINC03040
7
6
5
cntrl_control
UC_active
color
UC
cntrl

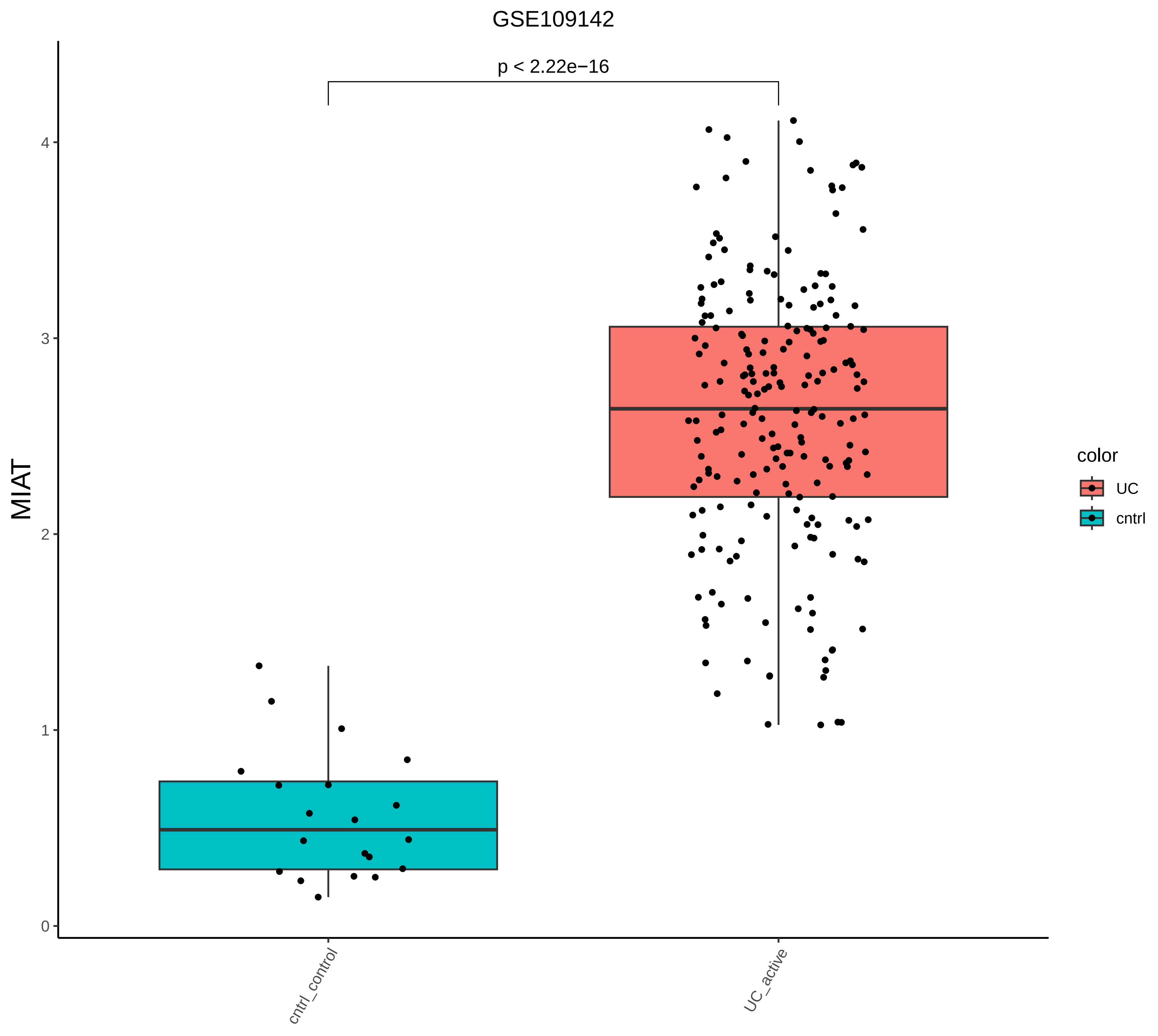
GSE109142
p < 2.22e−16
MIAT
4
3
2
1
0
cntrl_control
UC_active
color
UC
cntrl

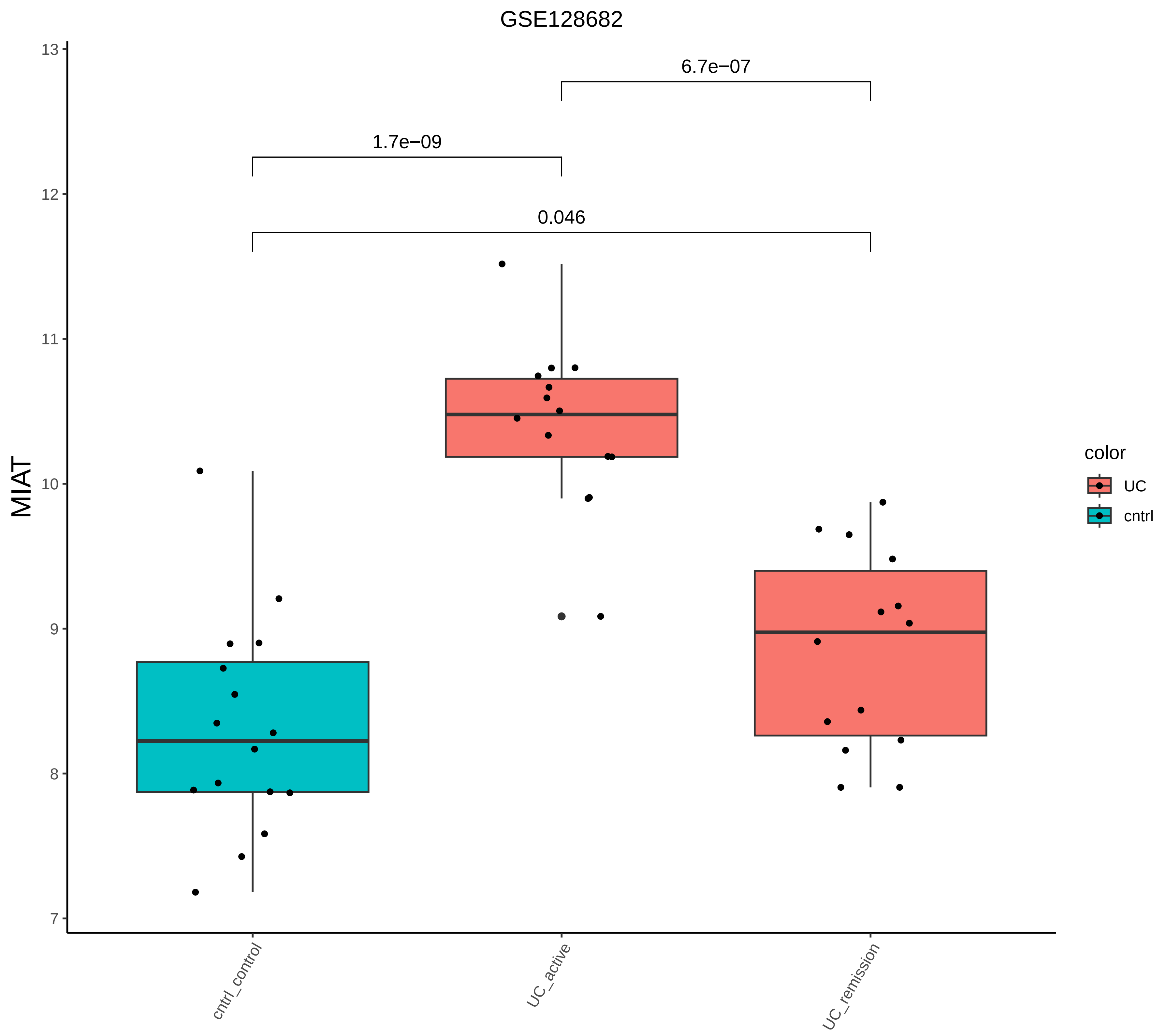
GSE128682
MIAT
6.7e−07
1.7e−09
0.046
13
12
11
10
9
8
7
cntrl_control
UC_active
UC_remission
color
UC
cntrl

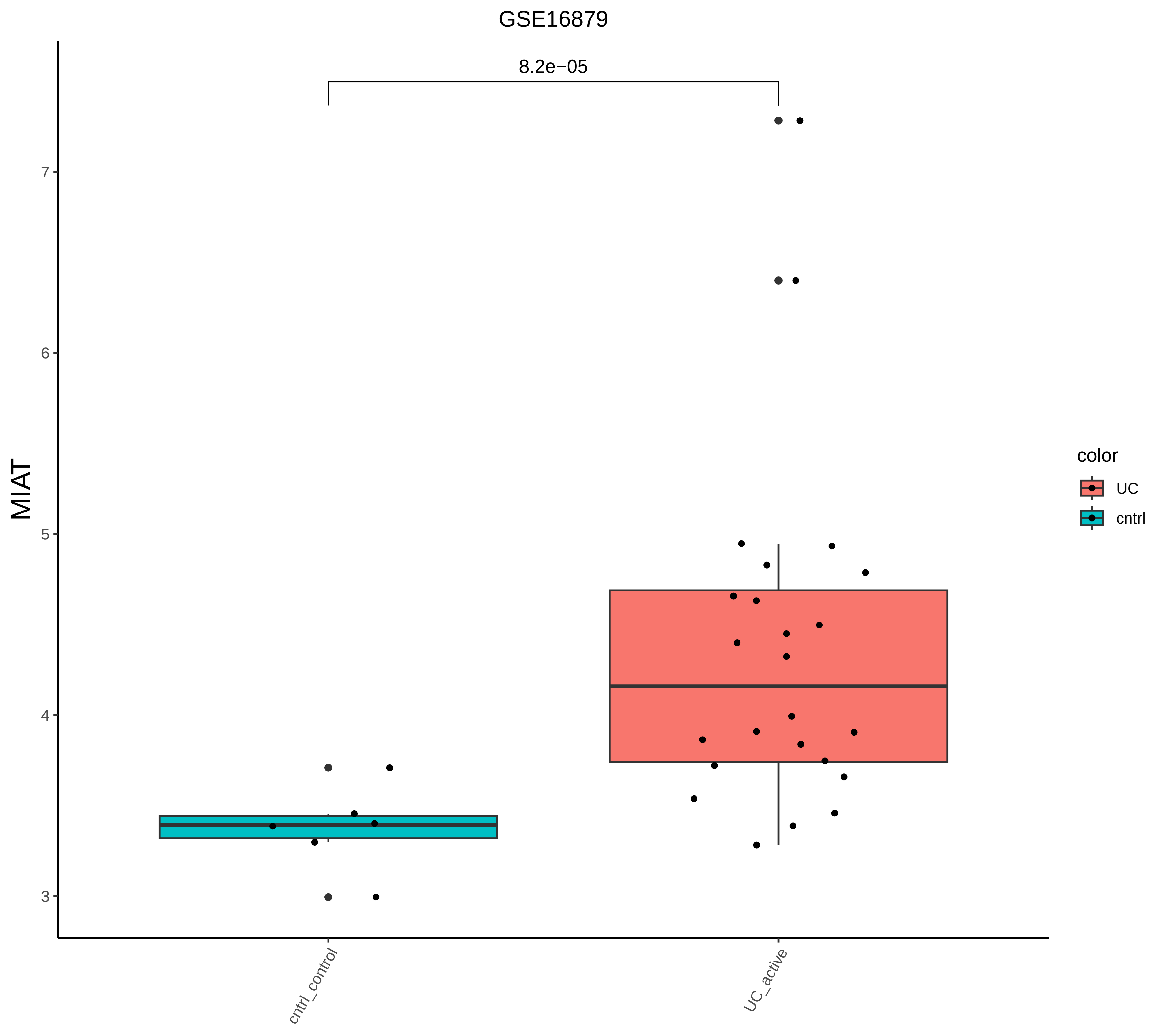

GSE16879
8.2e−05
MIAT
7
6
5
4
3
cntrl_control
UC_active
color
UC
cntrl

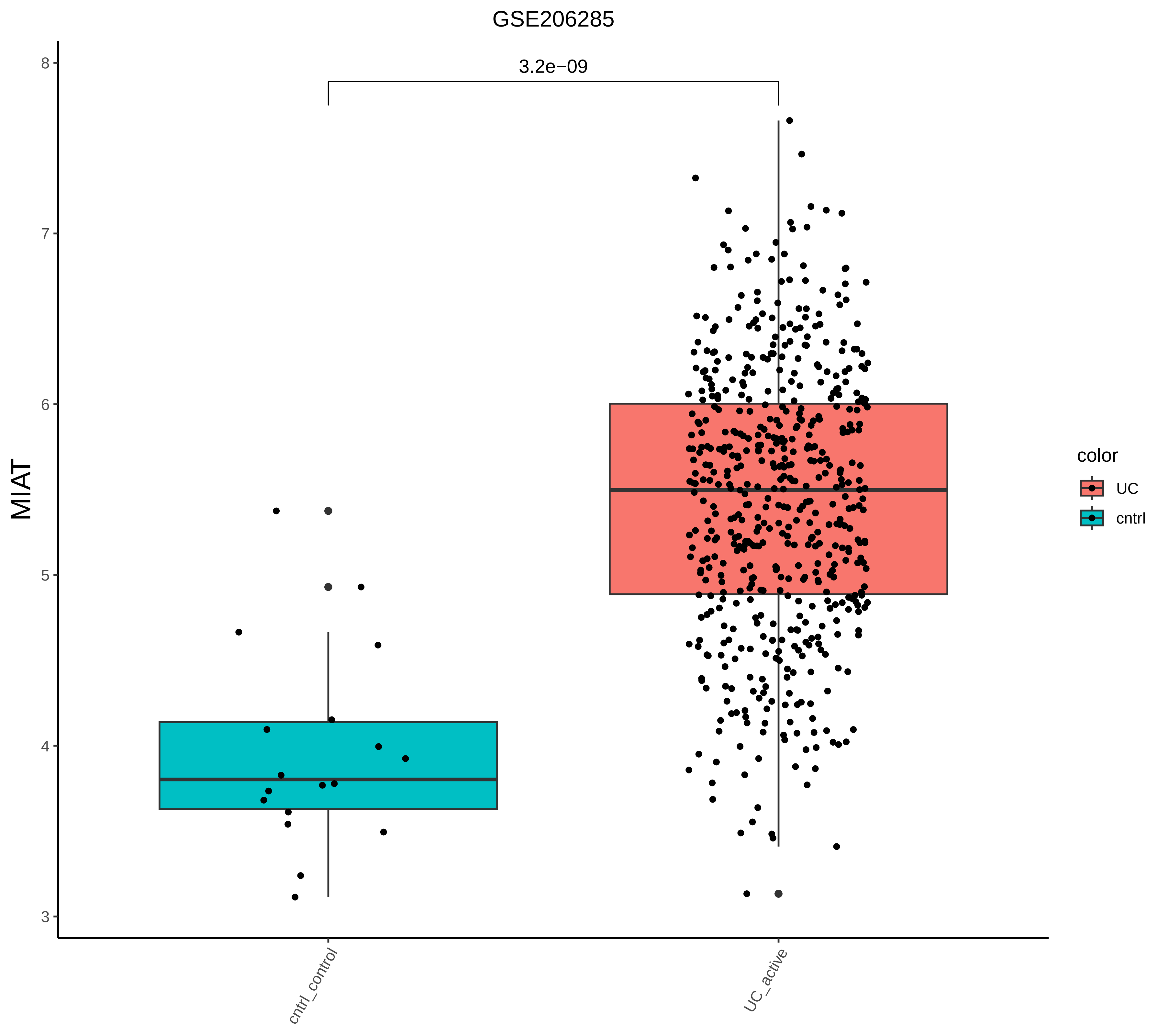

GSE206285
3.2e−09
MIAT
8
7
6
5
4
3
cntrl_control
UC_active
color
UC
cntrl

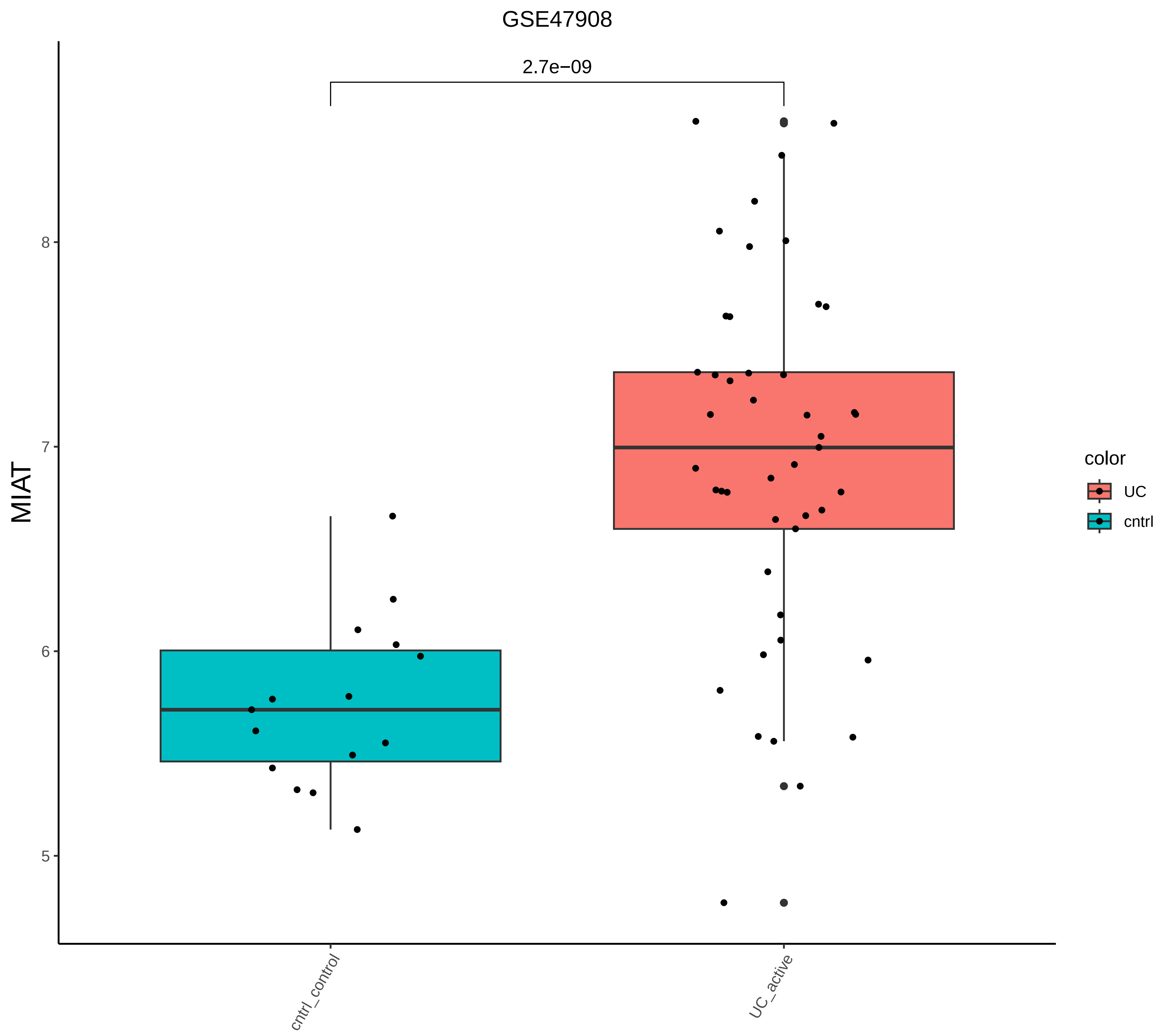
GSE47908
2.7e−09
MIAT
8
7
6
5
cntrl_control
UC_active
color
UC
cntrl

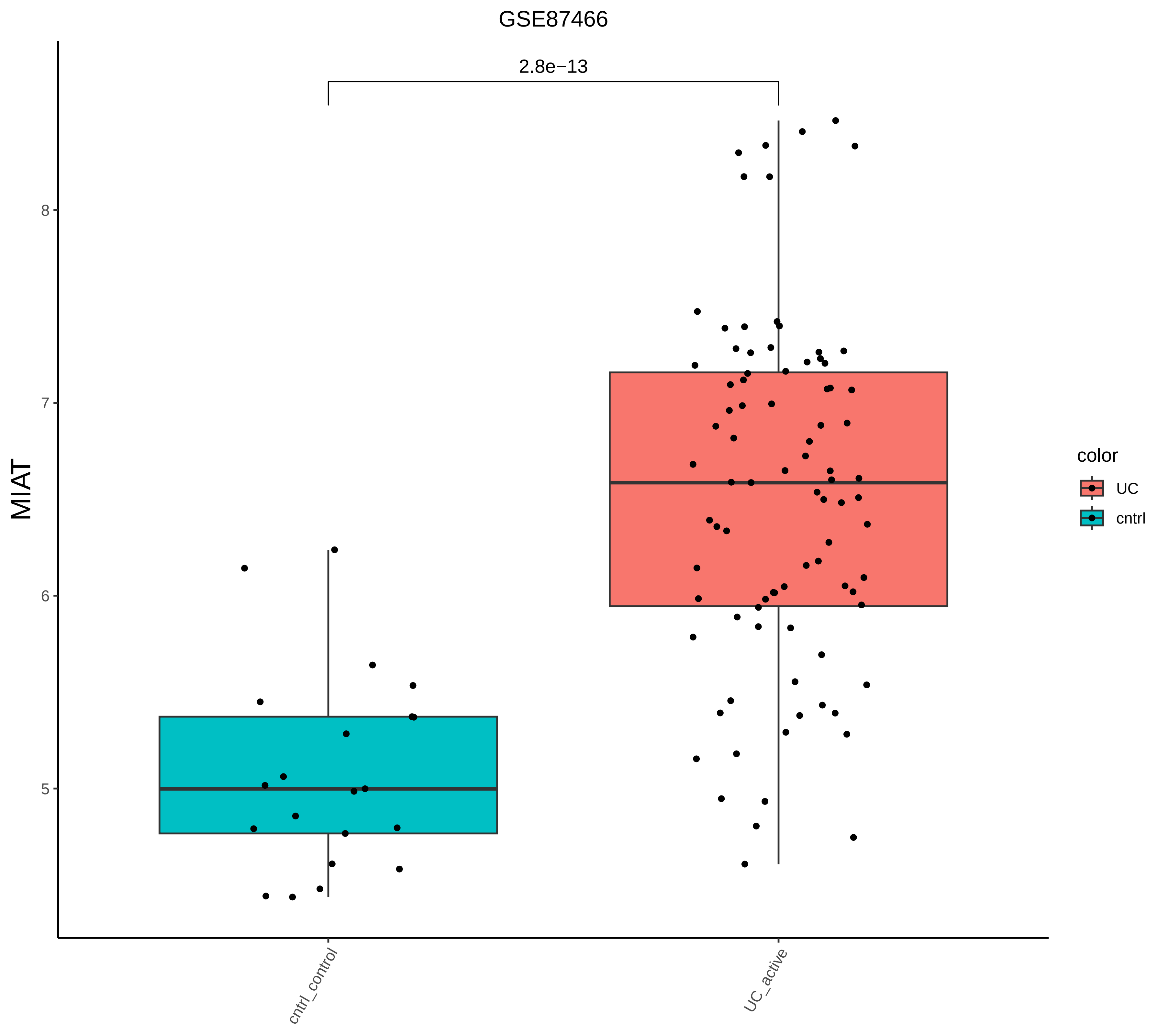
GSE87466
2.8e−13
MIAT
8
7
6
5
cntrl_control
UC_active
color
UC
cntrl

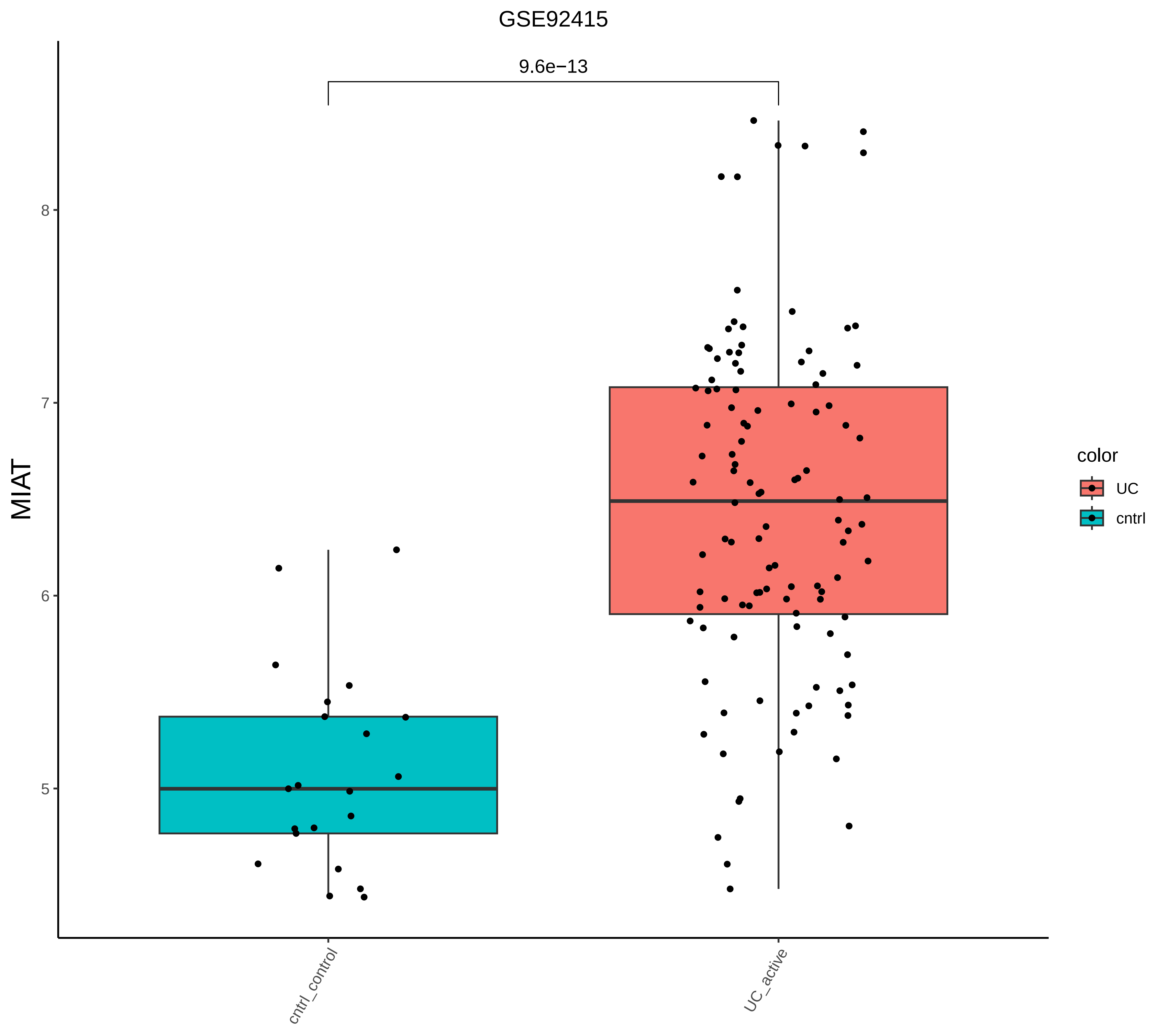
GSE92415
9.6e−13
MIAT
8
7
6
5
cntrl_control
UC_active
color
UC
cntrl

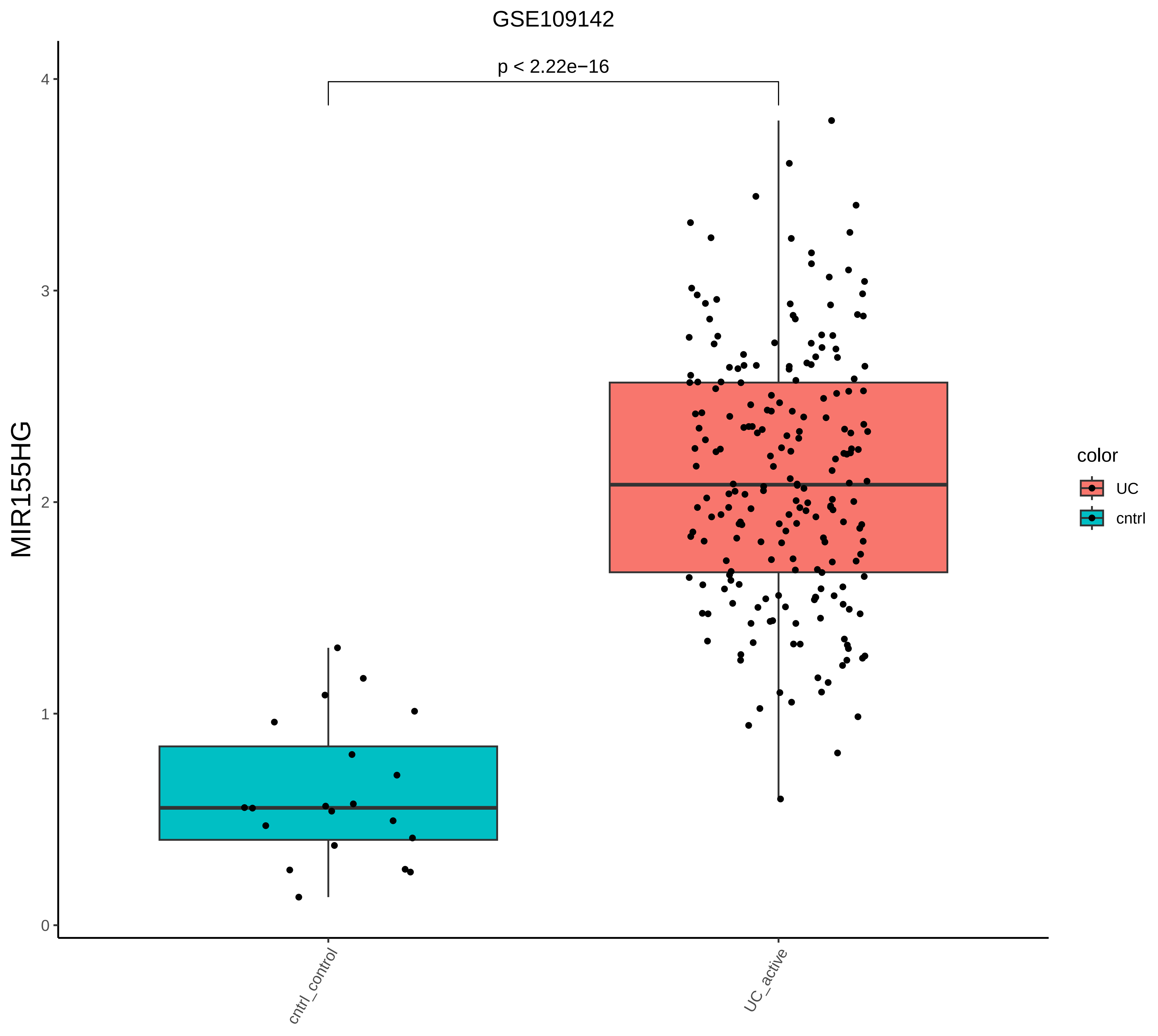
GSE109142
p < 2.22e−16
MIR155HG
0
1
2
3
4
cntrl_control
UC_active
color
UC
cntrl

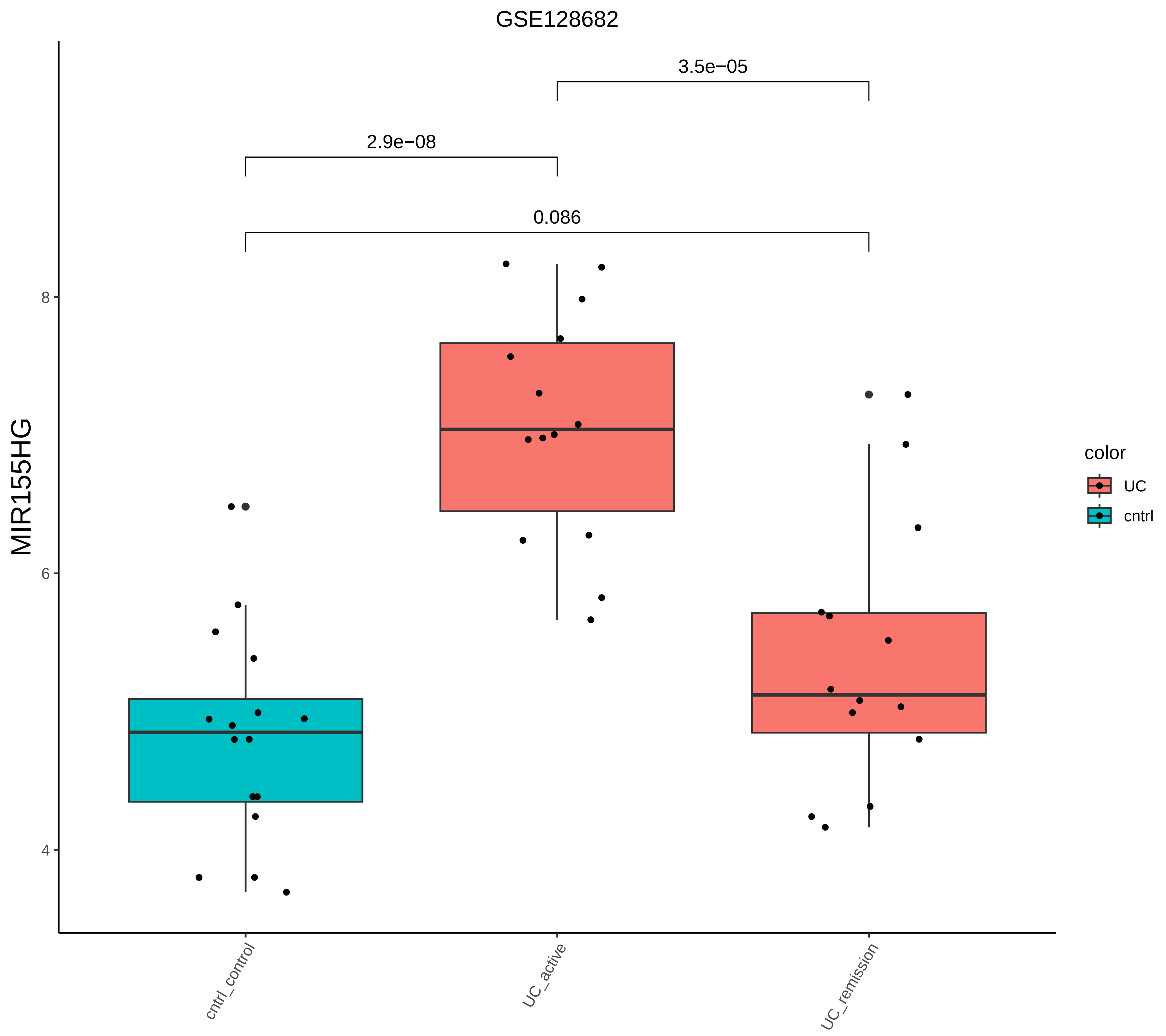
GSE128682
3.5e−05
2.9e−08
0.086
MIR155HG
8
6
4
color
UC
cntrl
cntrl_control
UC_active
UC_remission

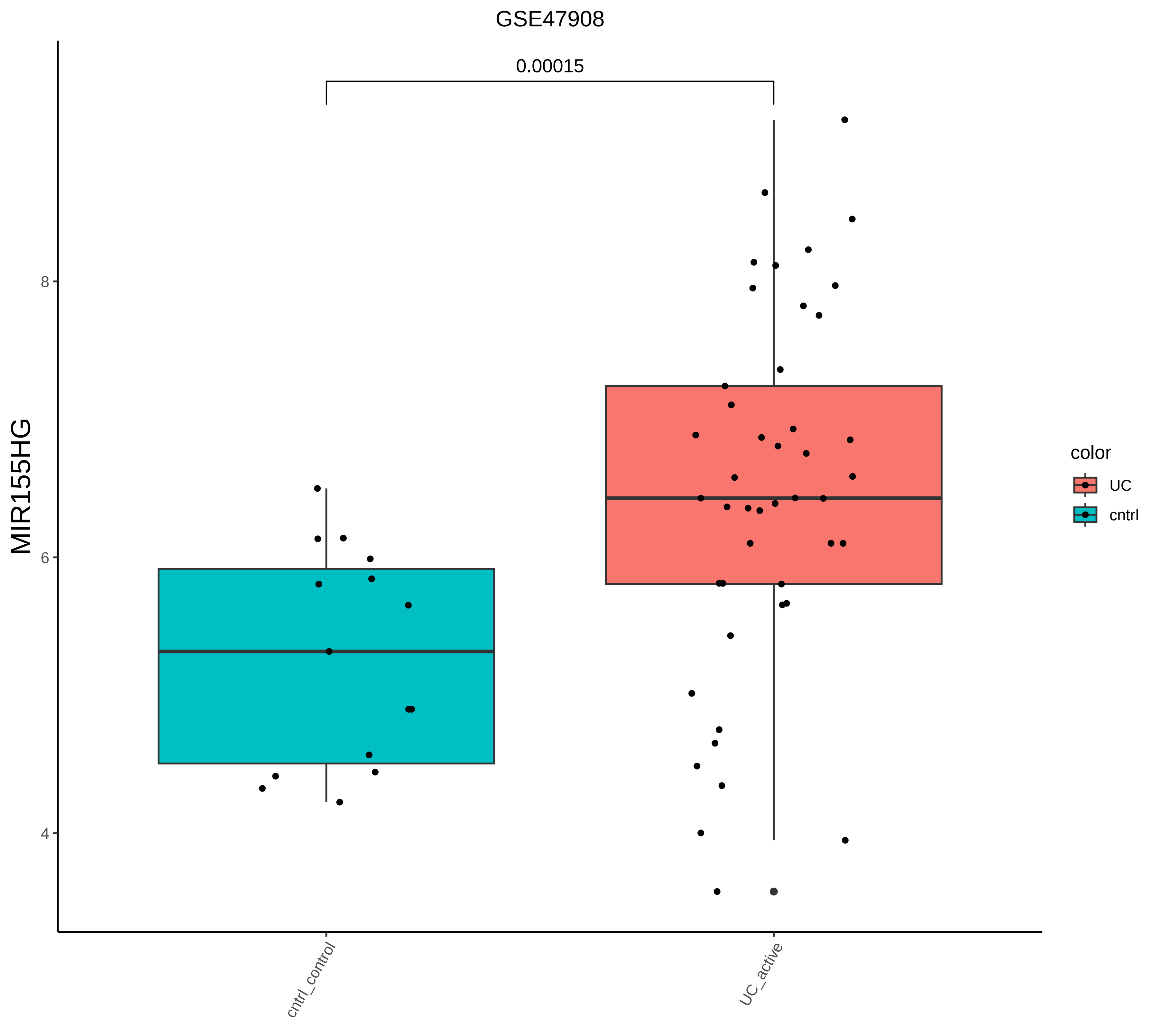

GSE47908
0.00015
MIR155HG
8
6
4
cntrl_control
UC_active
color
UC
cntrl

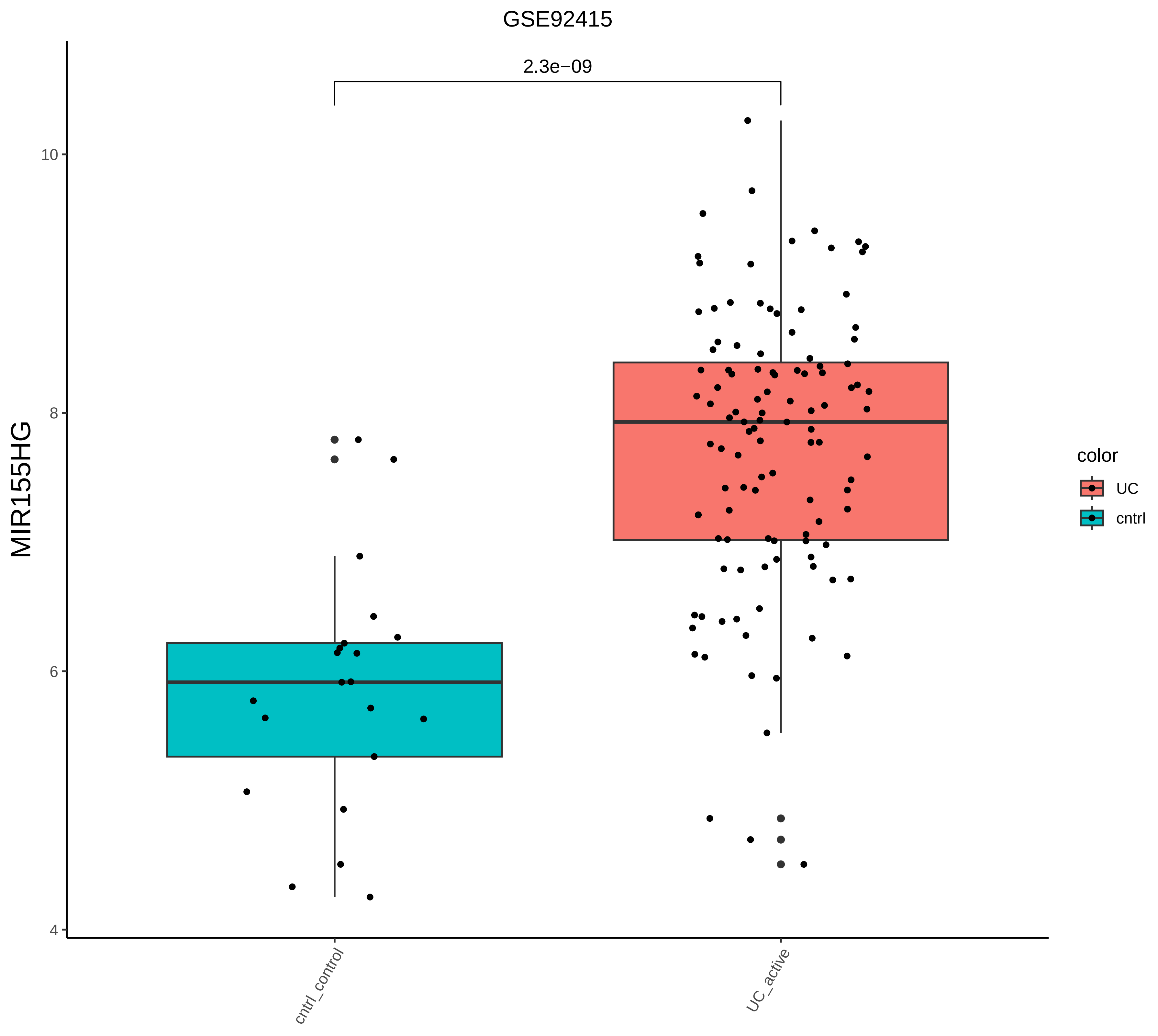
GSE92415
2.3e−09
MIR155HG
10
8
6
4
cntrl_control
UC_active
color
UC
cntrl

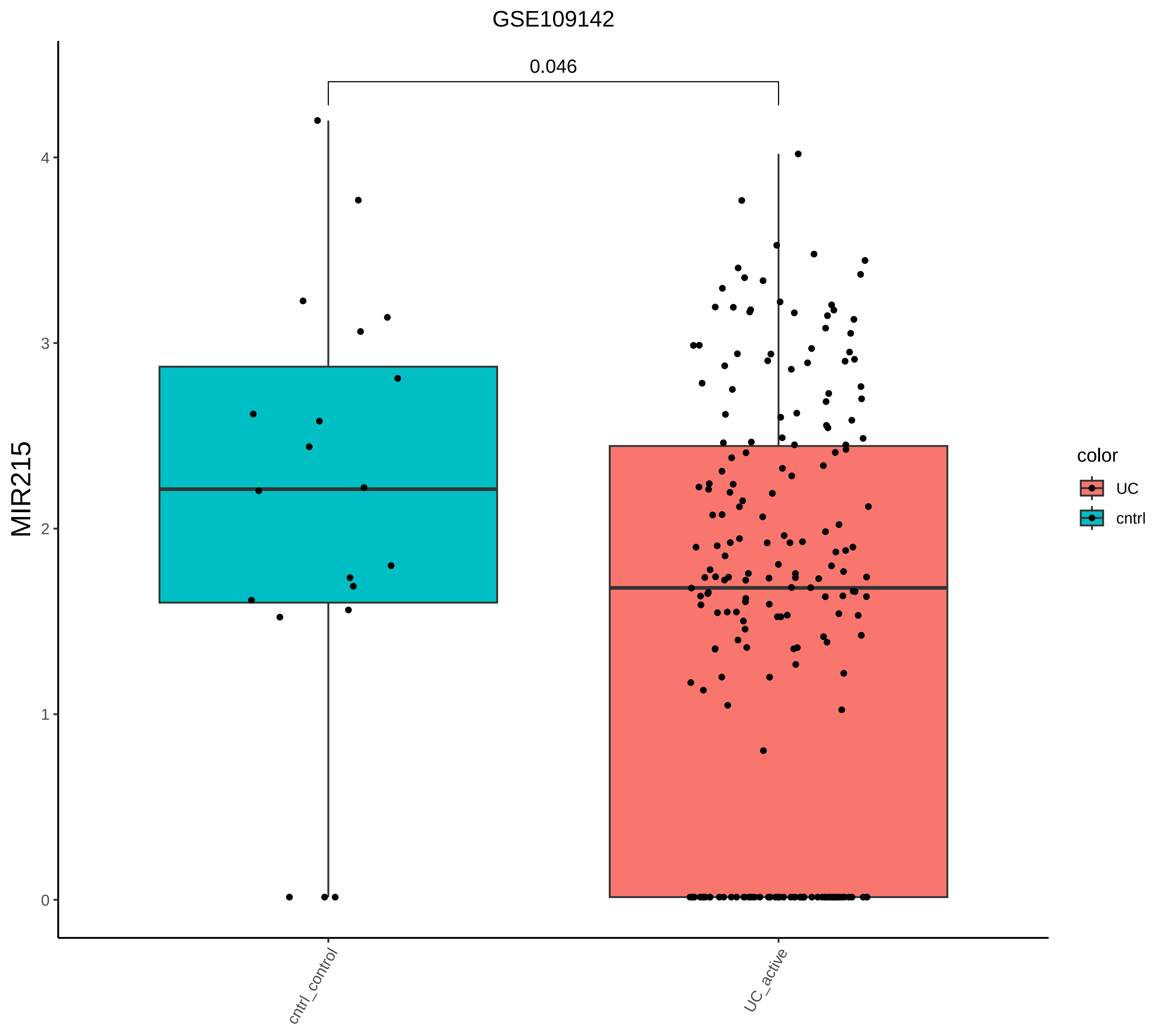

GSE109142
0.046
MIR215
0
1
2
3
4
cntrl_control
UC_active
color
UC
cntrl

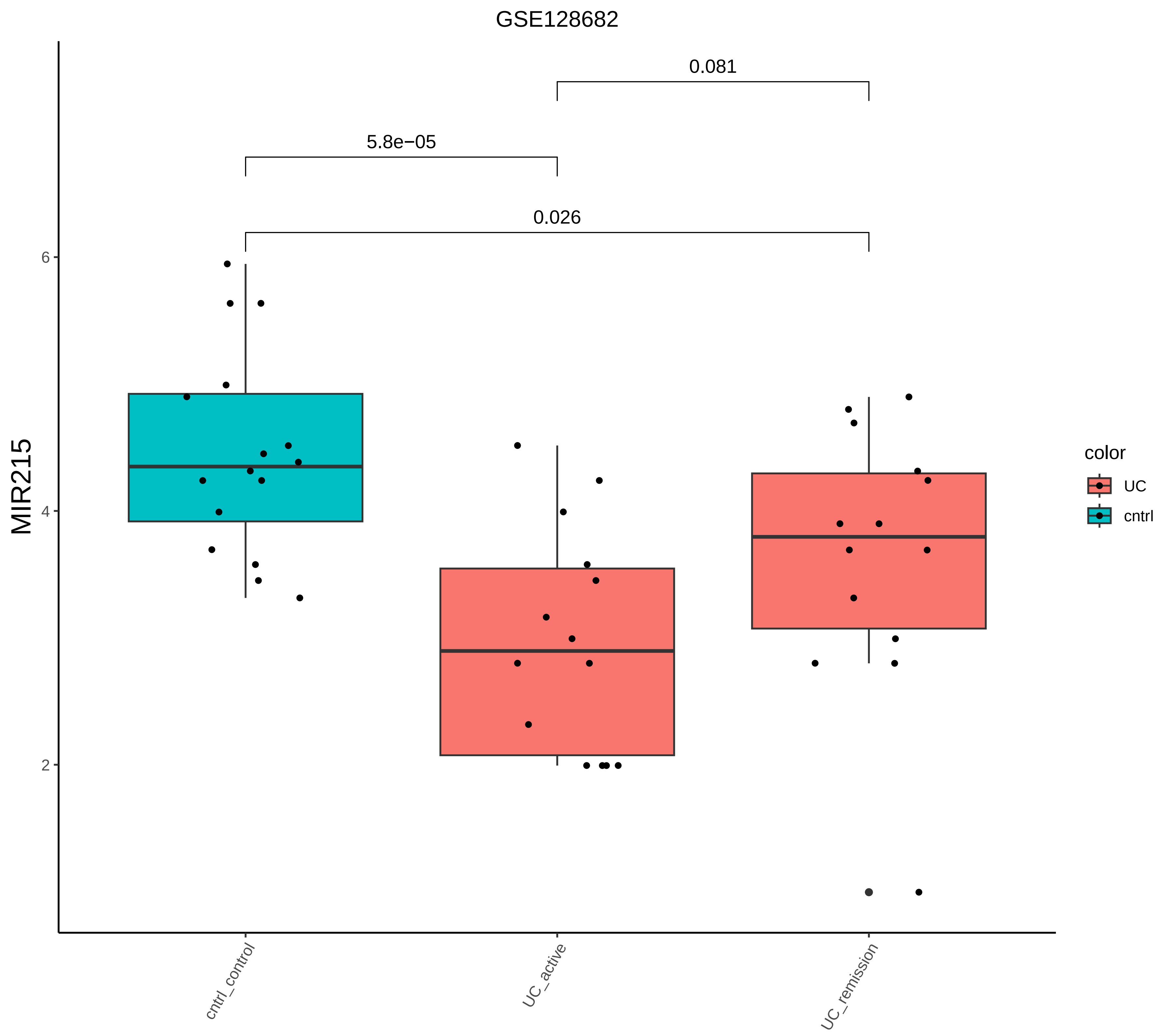

GSE128682
0.081
5.8e−05
0.026
MIR215
6
4
2
cntrl_control
UC_active
UC_remission
color
UC
cntrl

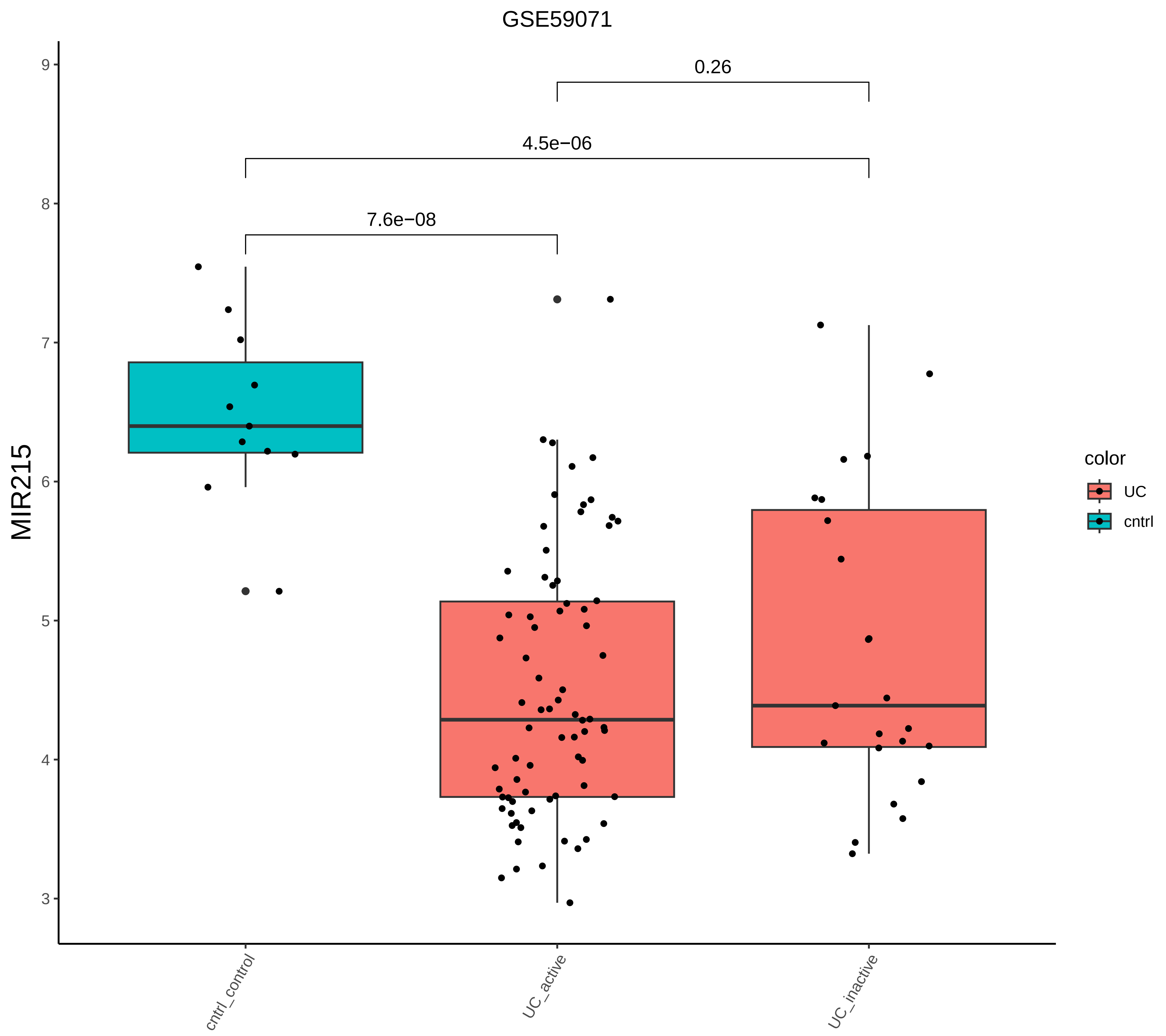

GSE59071
0.26
4.5e−06
7.6e−08
MIR215
9
8
7
6
5
4
3
cntrl_control
UC_active
UC_inactive
color
UC
cntrl

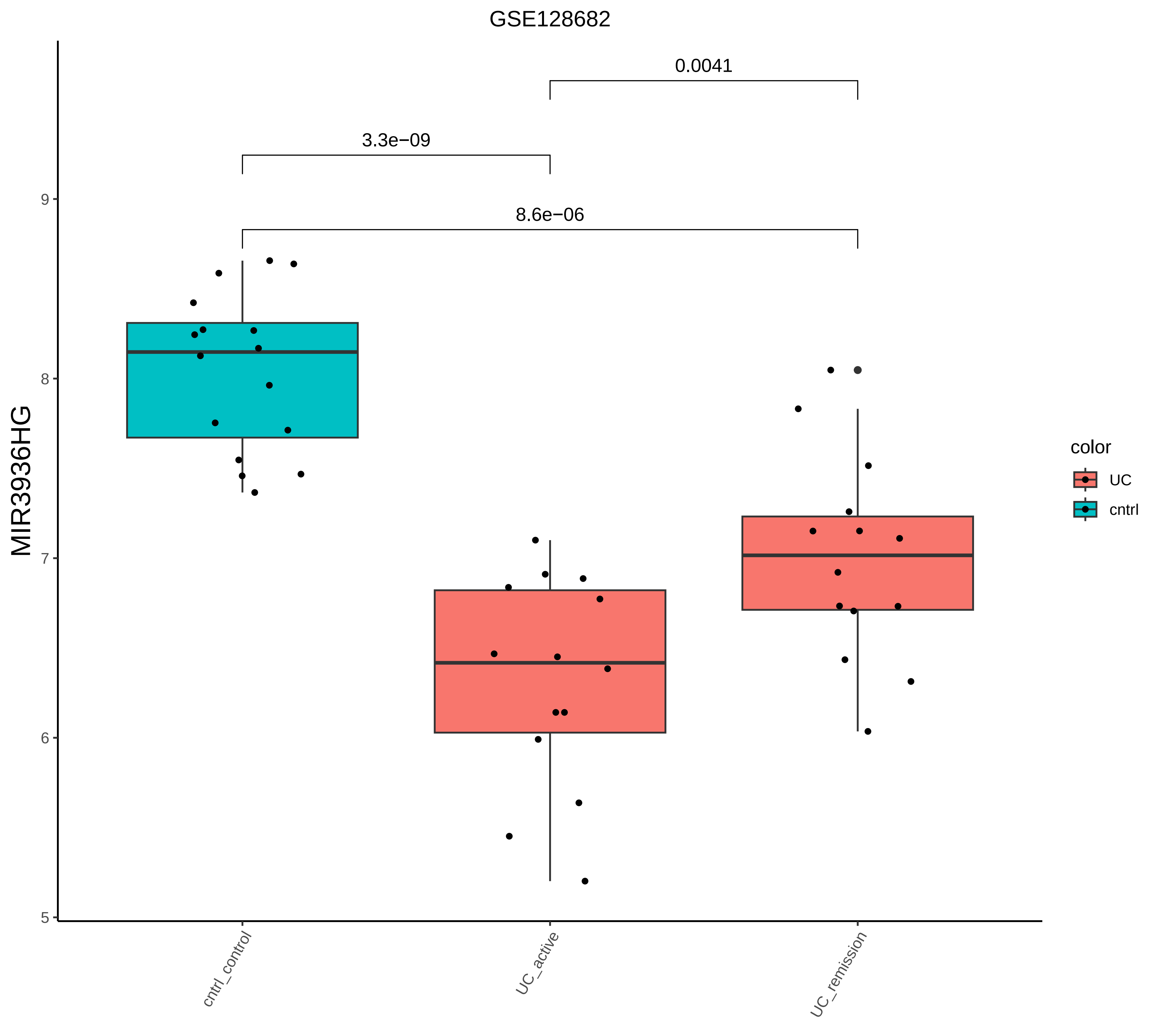

GSE128682
0.0041
3.3e−09
8.6e−06
MIR3936HG
9
8
7
6
5
cntrl_control
UC_active
UC_remission
color
UC
cntrl

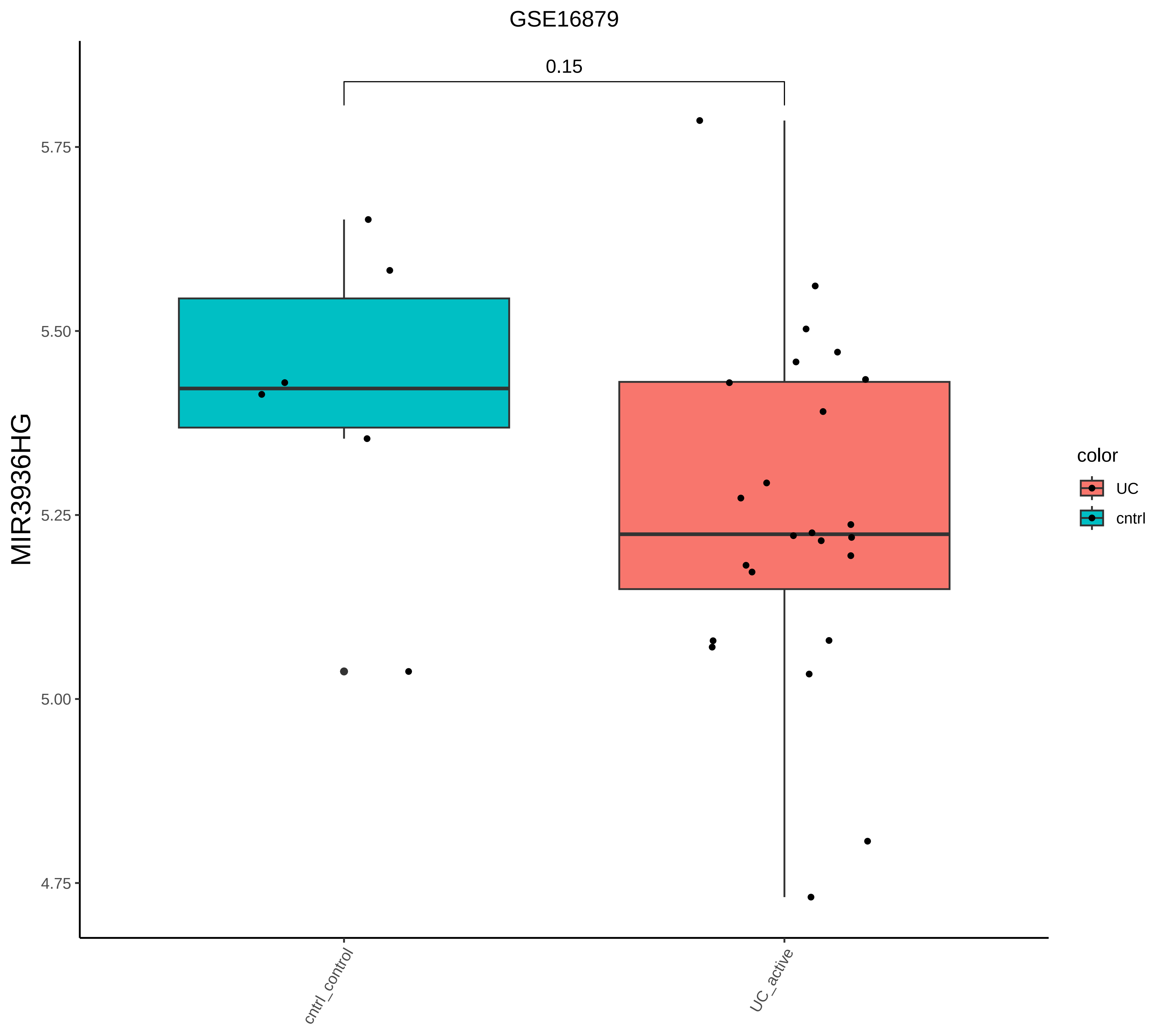

GSE16879
0.15
MIR3936HG
5.75
5.50
5.25
5.00
4.75
cntrl_control
UC_active
color
UC
cntrl

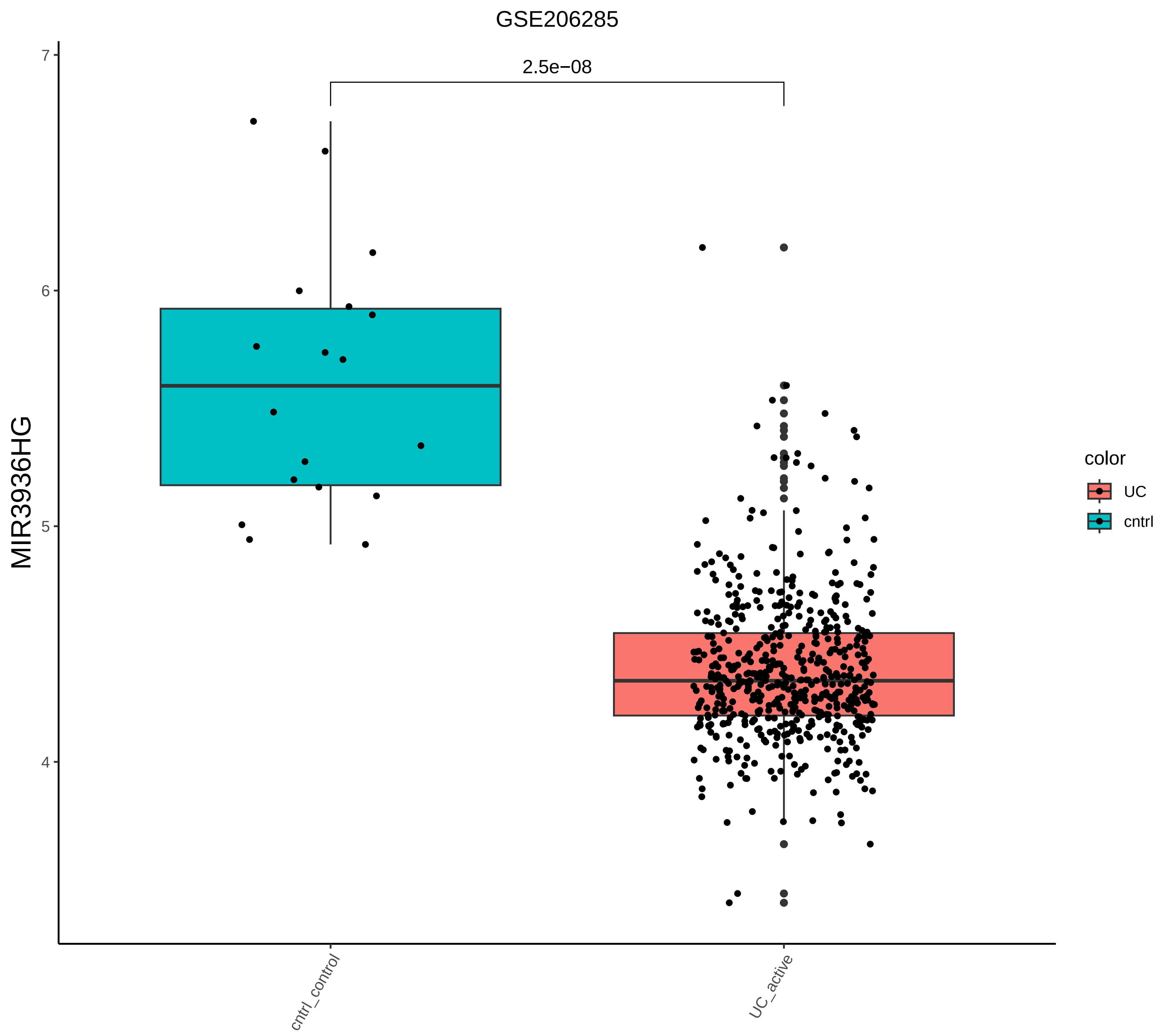

GSE206285
2.5e−08
MIR3936HG
7
6
5
4
cntrl_control
UC_active
color
UC
cntrl

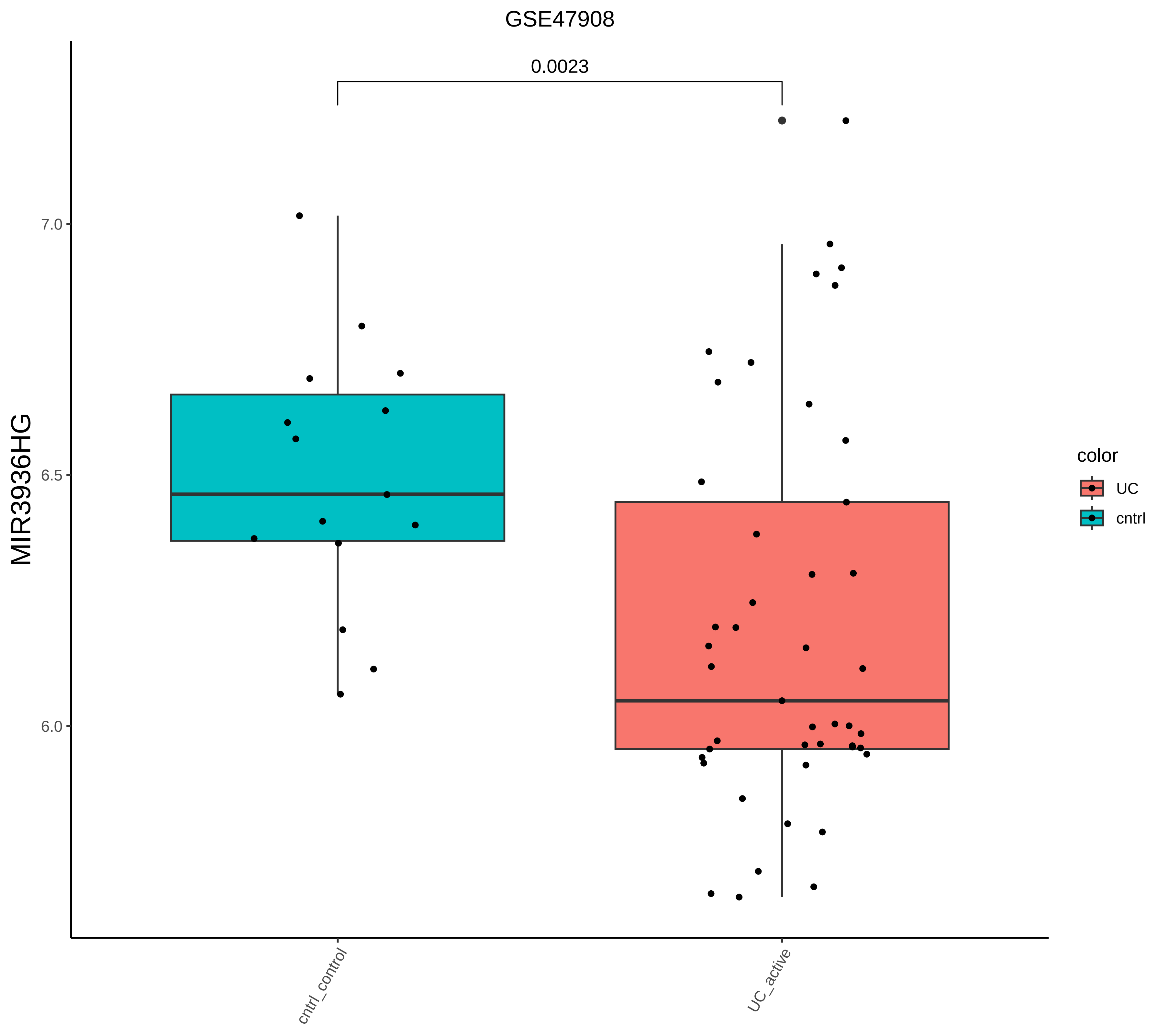

GSE47908
0.0023
MIR3936HG
7.0
6.5
6.0
cntrl_control
UC_active
color
UC
cntrl

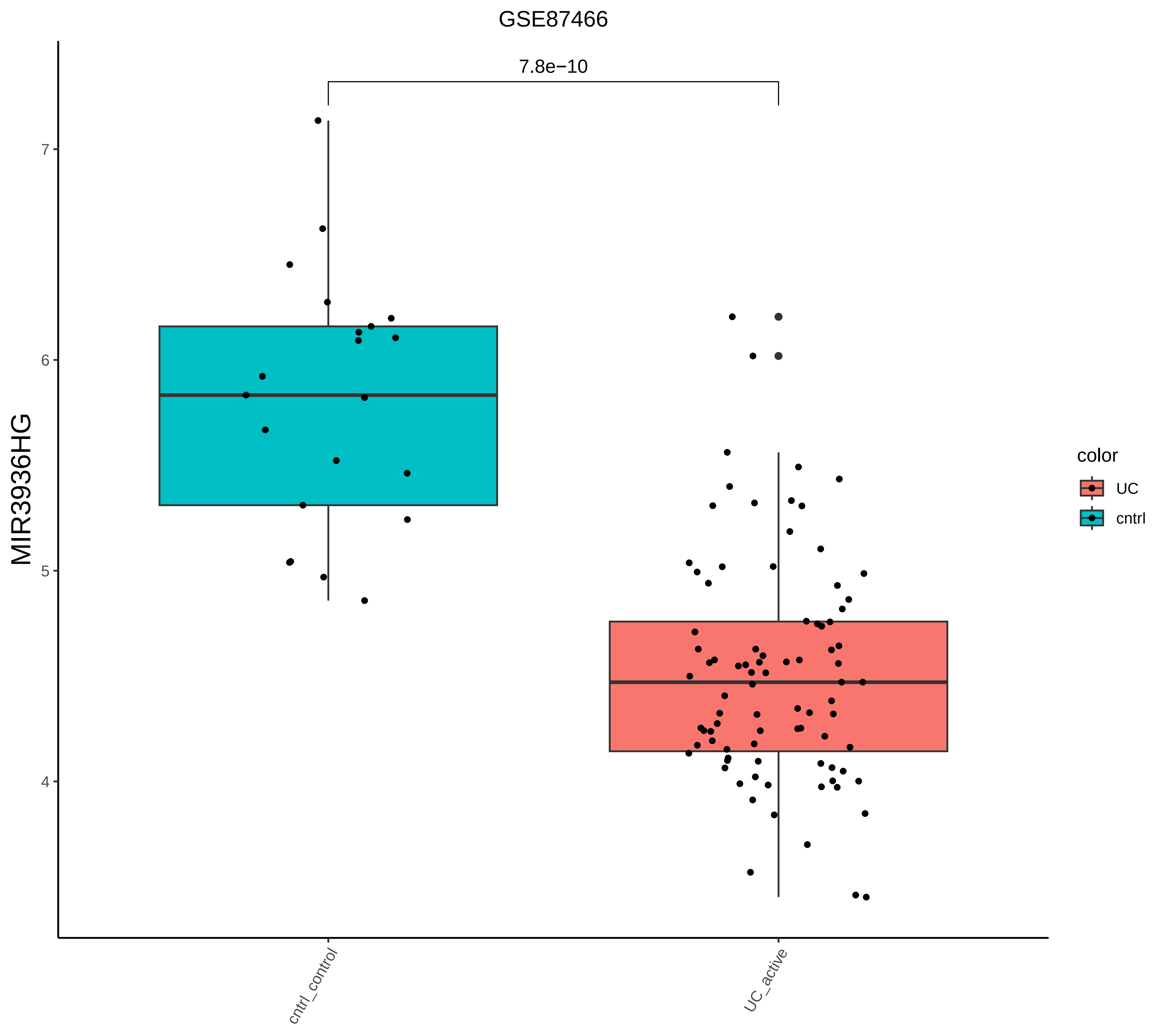

GSE87466
7.8e−10
MIR3936HG
7
6
5
4
cntrl_control
UC_active
color
UC
cntrl

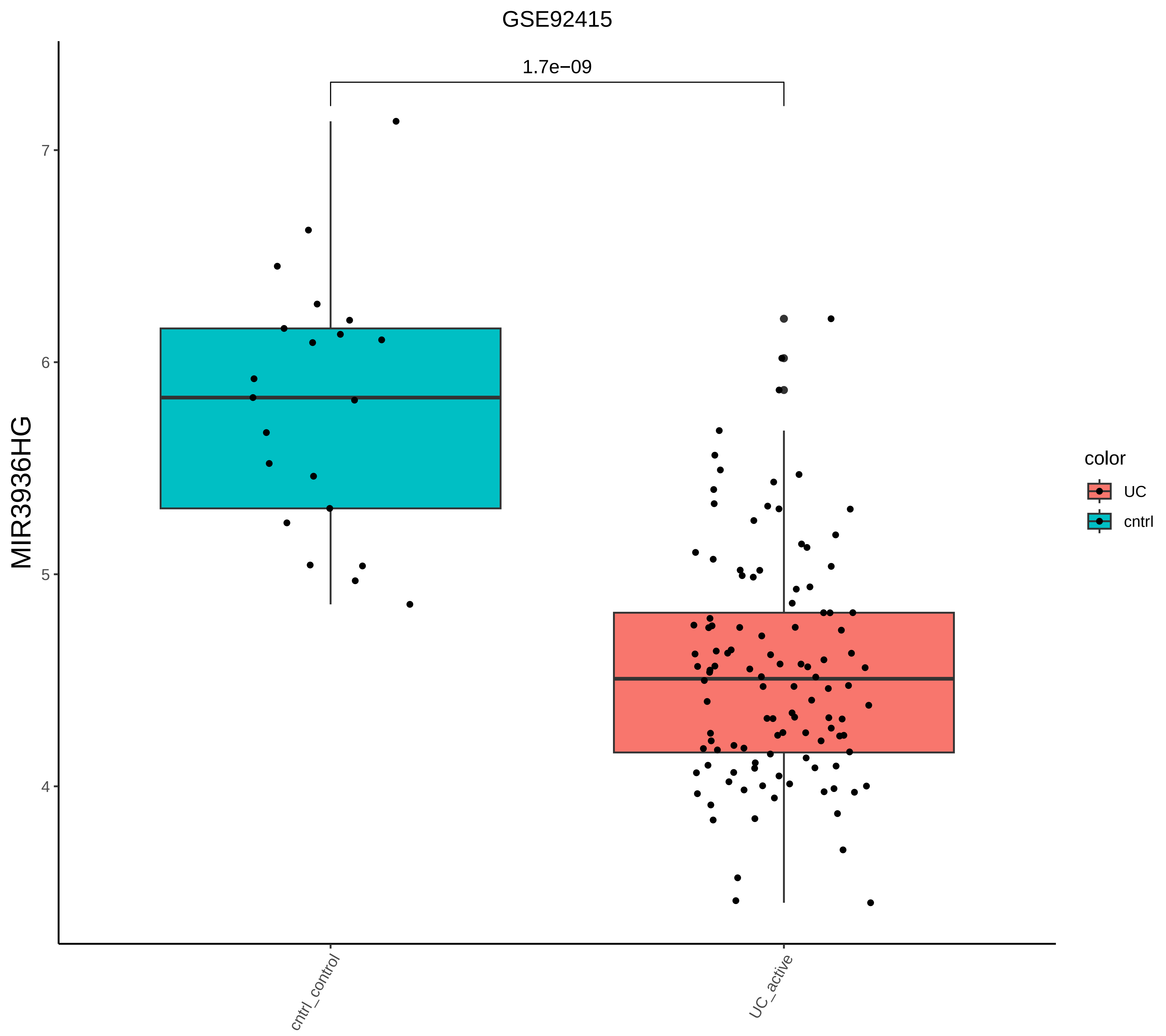

GSE92415
1.7e−09
MIR3936HG
7
6
5
4
cntrl_control
UC_active
color
UC
cntrl

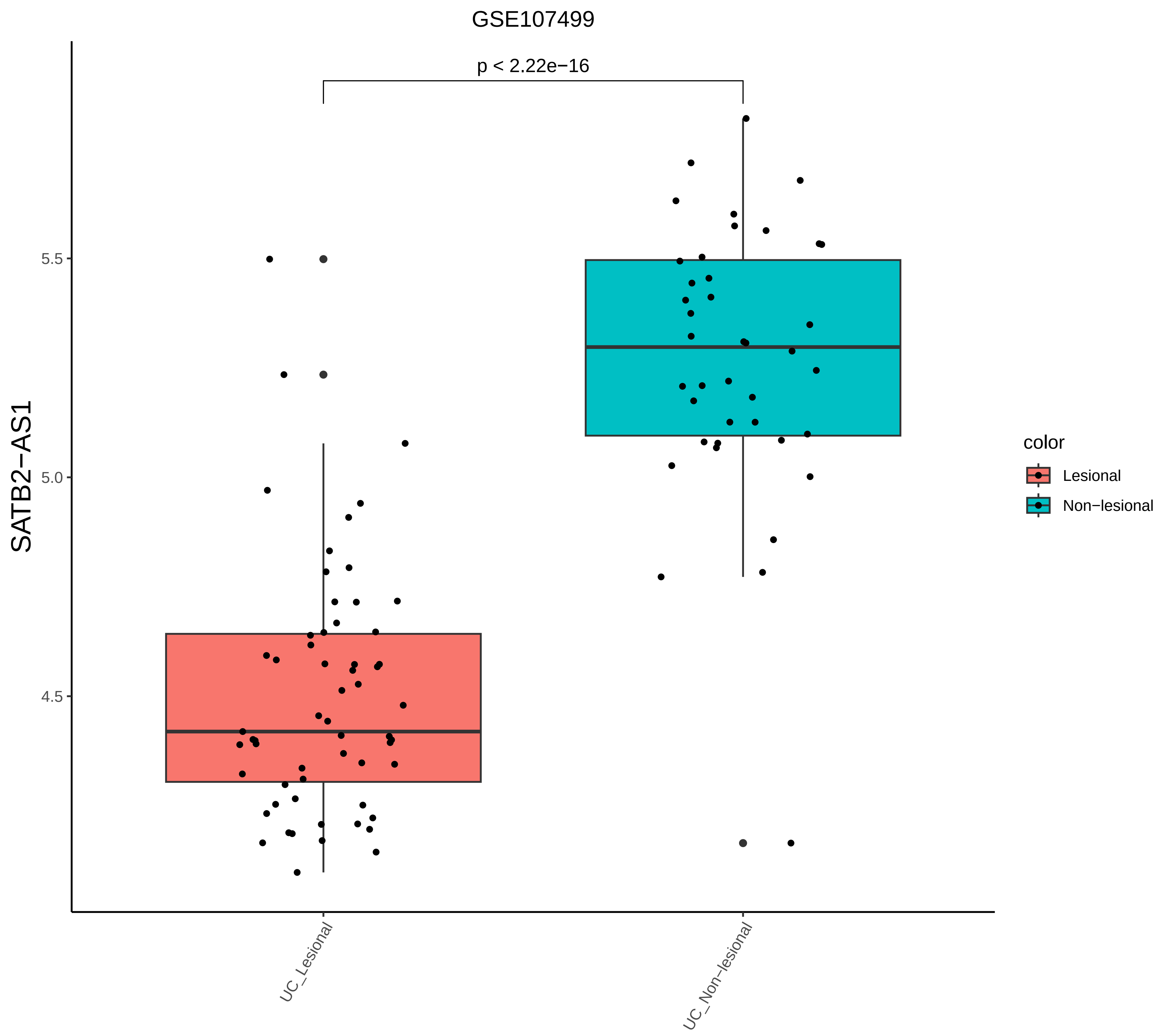
GSE107499
p < 2.22e−16
SATB2−AS1
5.5
5.0
4.5
UC_Lesional
UC_Non−lesional
color
Lesional
Non−lesional

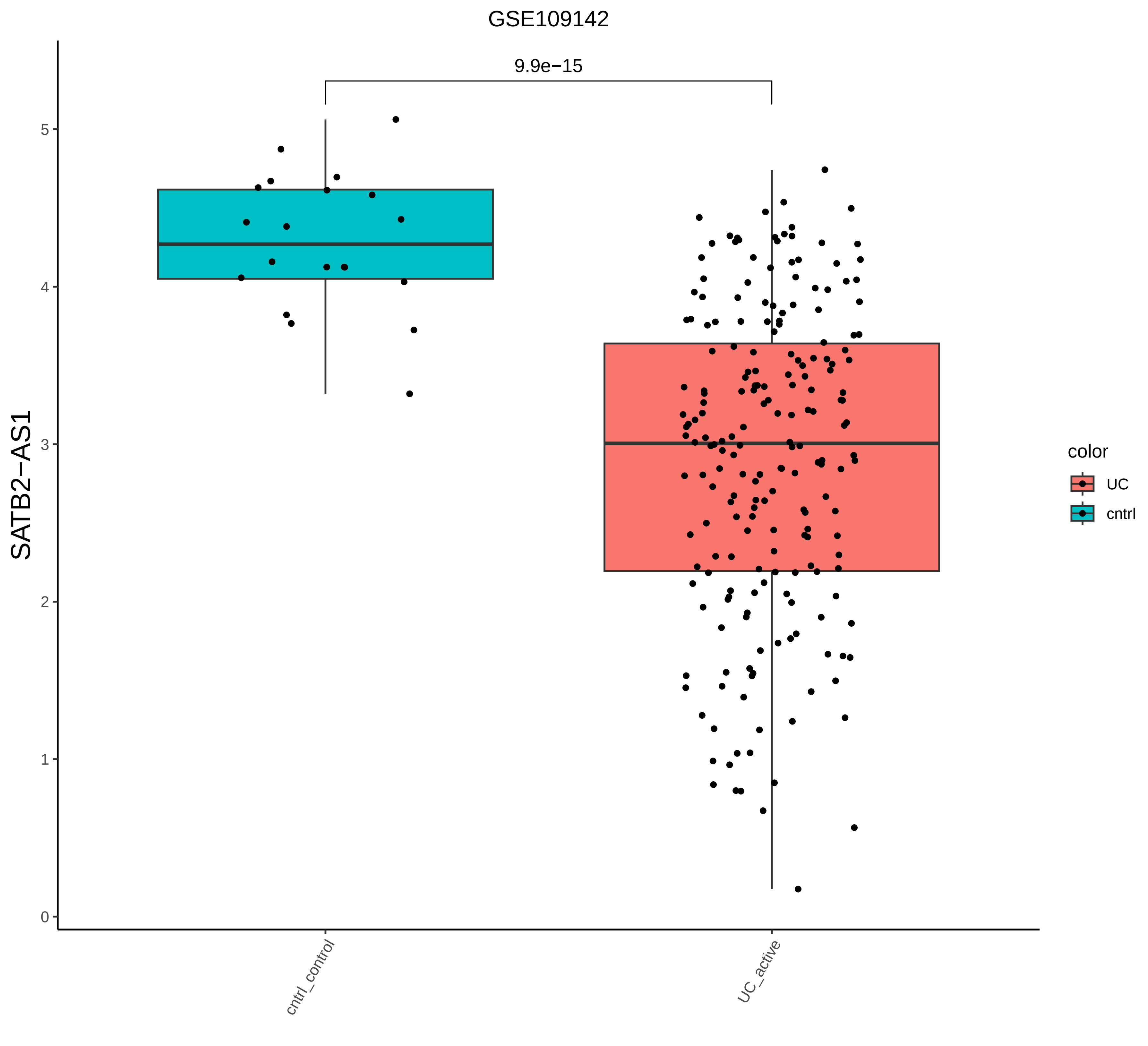
GSE109142
9.9e−15
SATB2−AS1
0
1
2
3
4
5
cntrl_control
UC_active
color
UC
cntrl

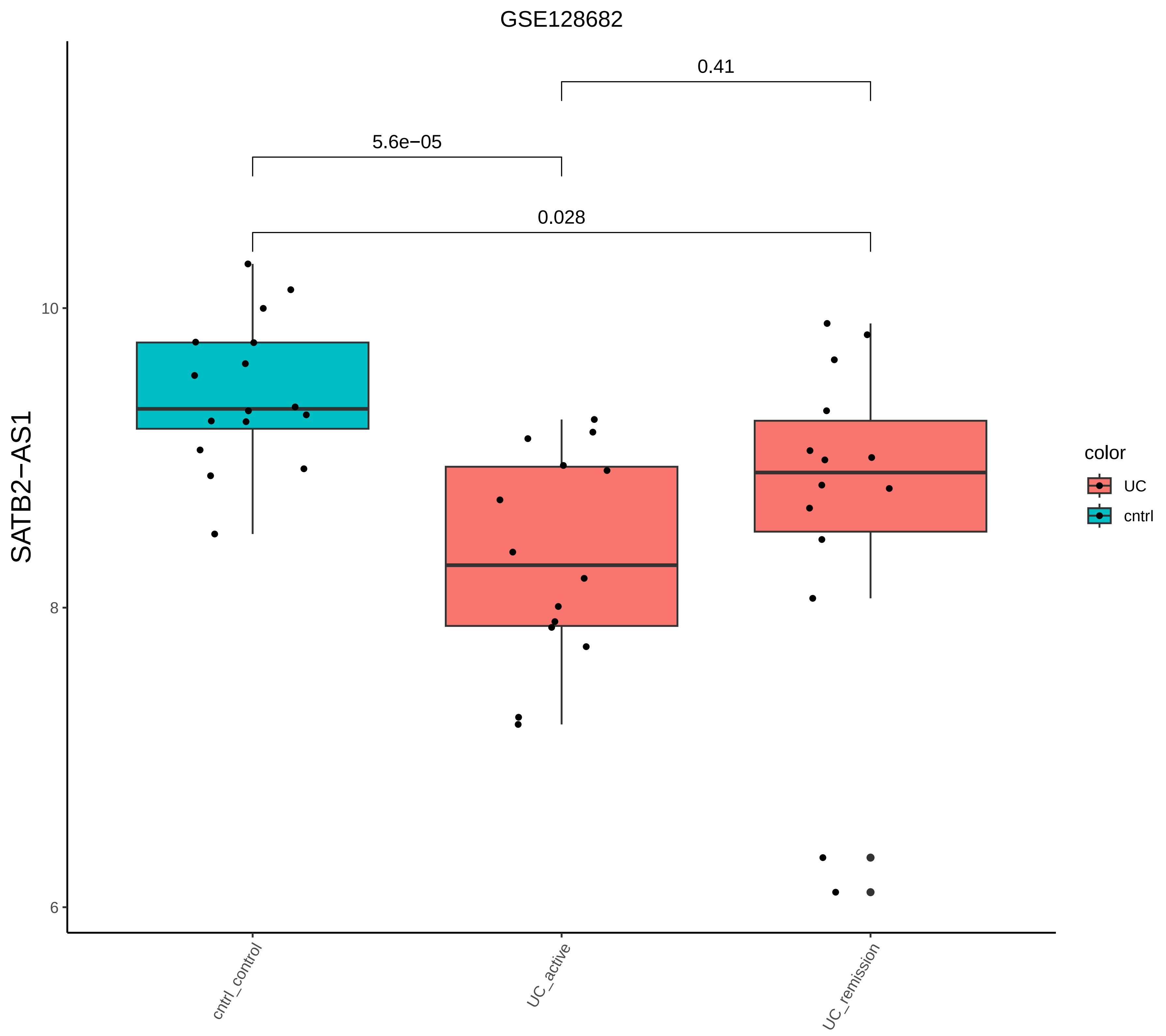
GSE128682
0.41
5.6e−05
0.028
SATB2−AS1
10
8
6
cntrl_control
UC_active
UC_remission
color
UC
cntrl

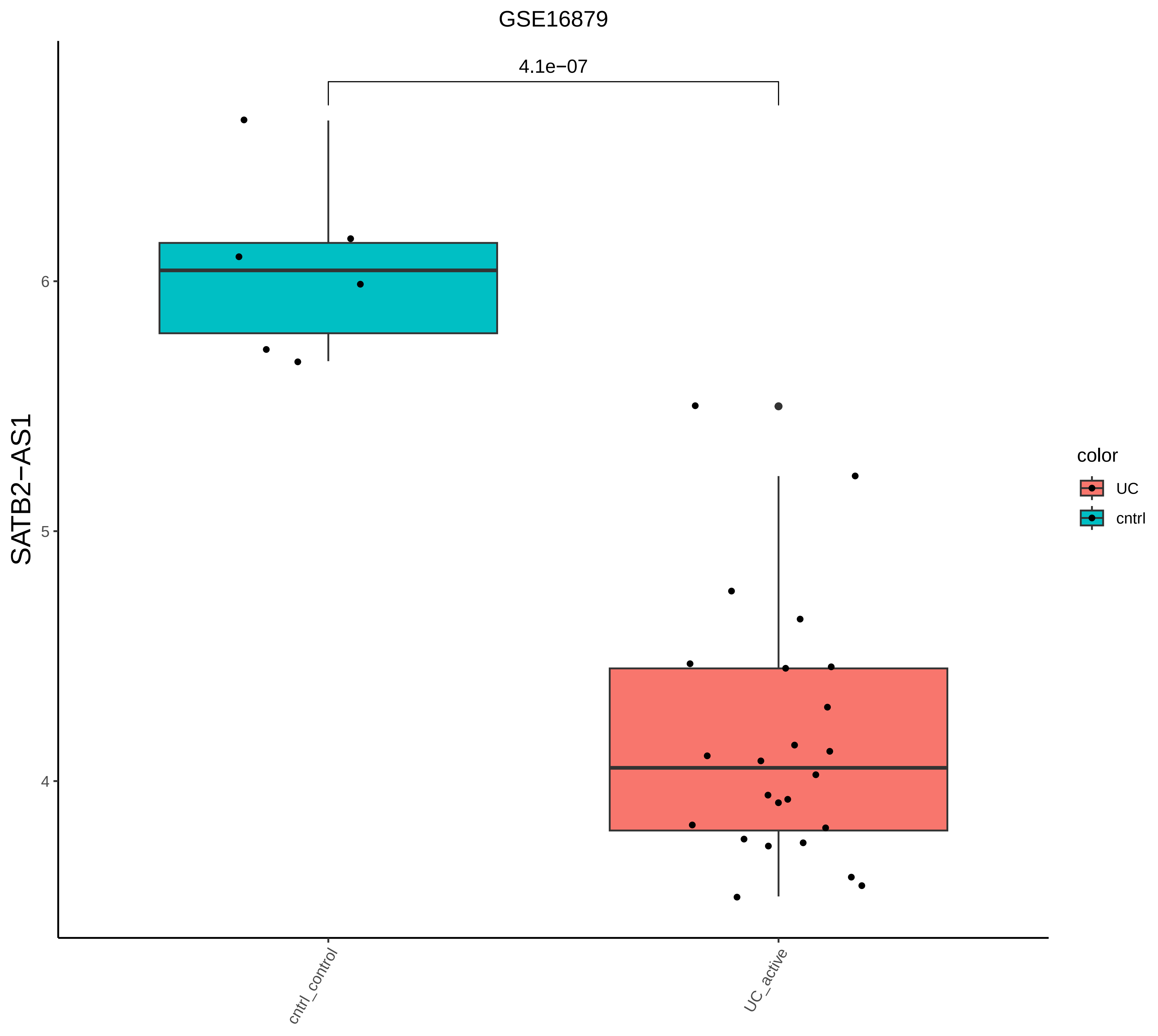
GSE16879
4.1e−07
SATB2−AS1
6
5
4
cntrl_control
UC_active
color
UC
cntrl

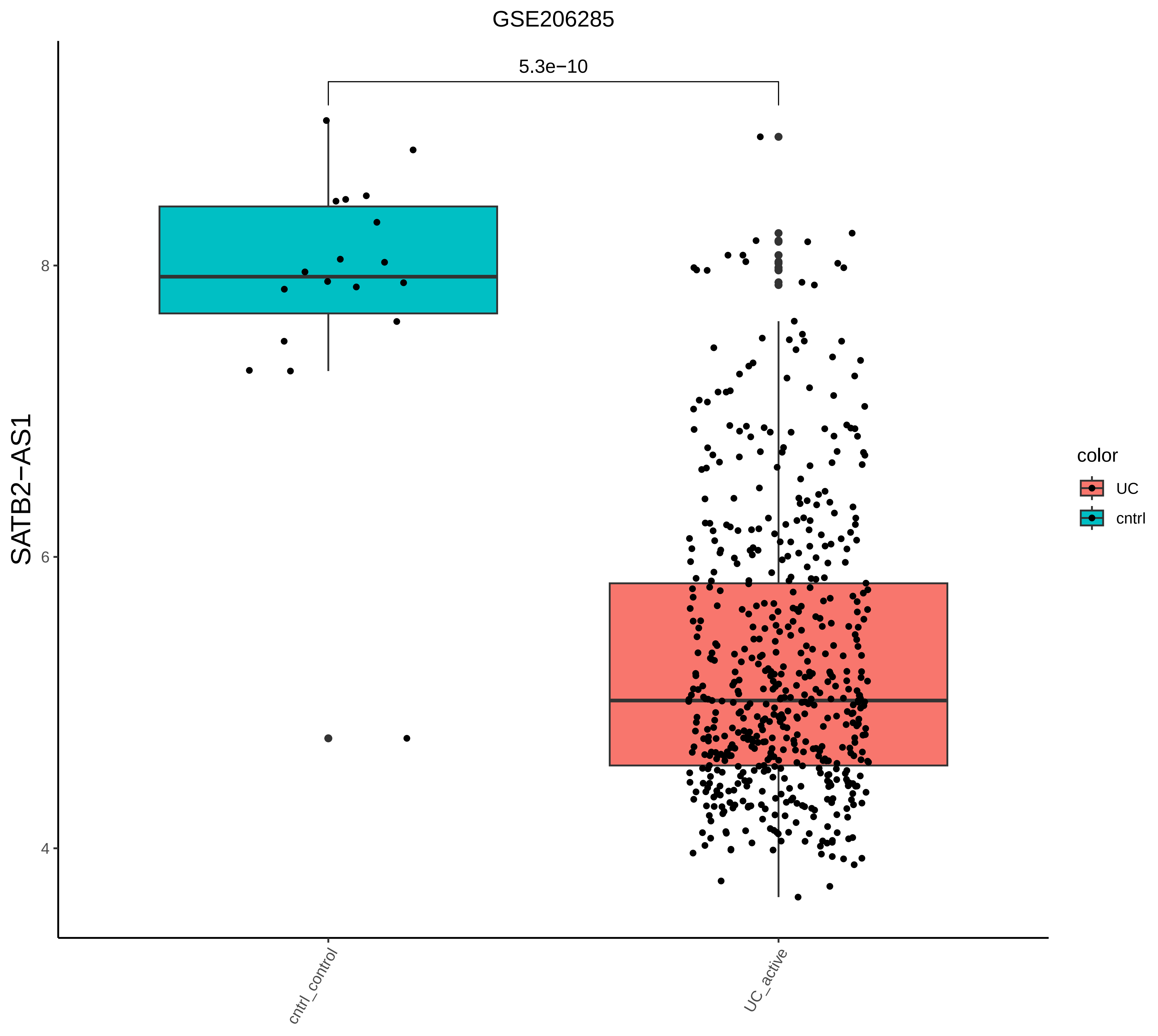
GSE206285
5.3e−10
SATB2−AS1
8
6
4
cntrl_control
UC_active
color
UC
cntrl

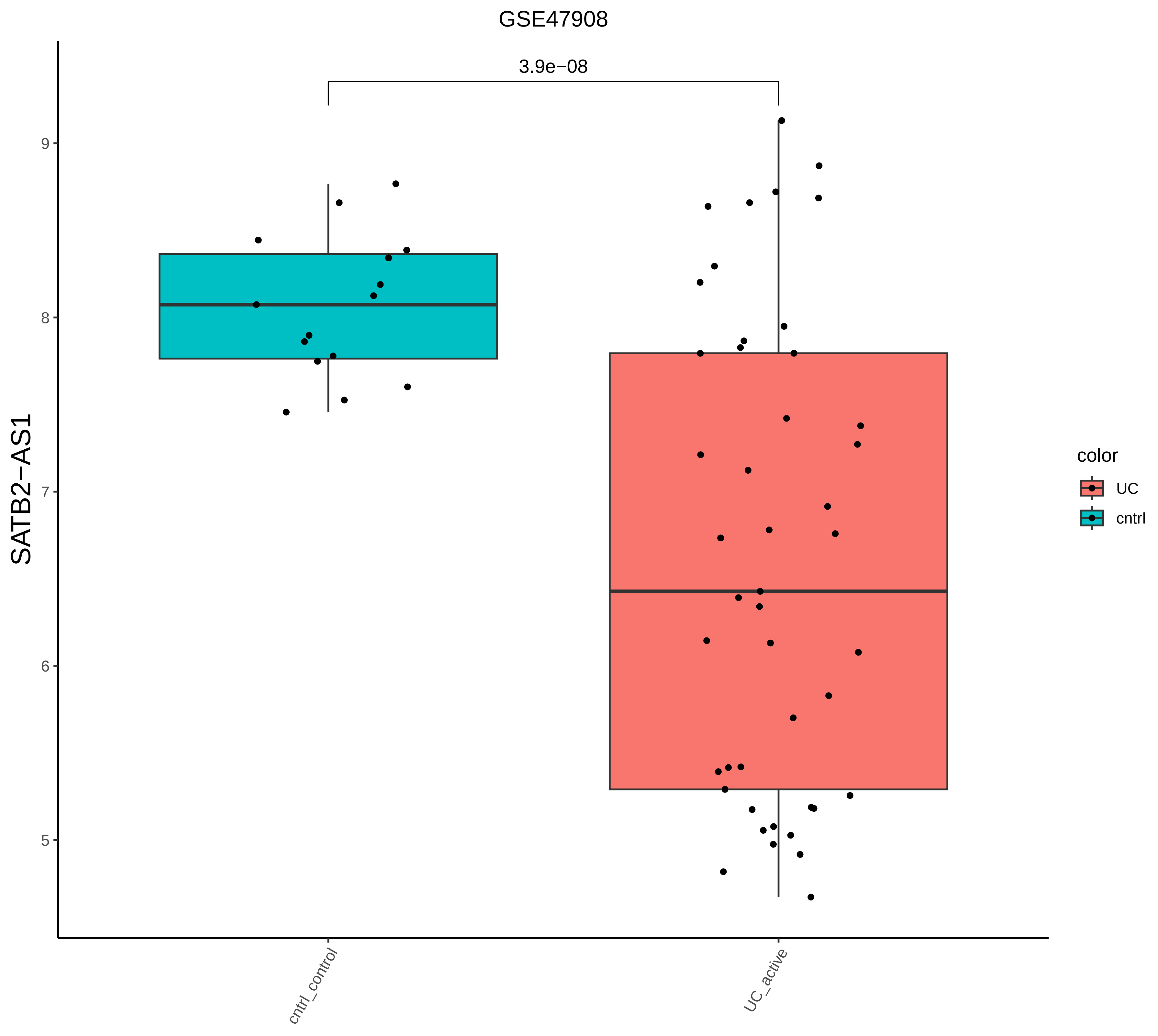
GSE47908
3.9e−08
SATB2−AS1
9
8
7
6
5
cntrl_control
UC_active
color
UC
cntrl

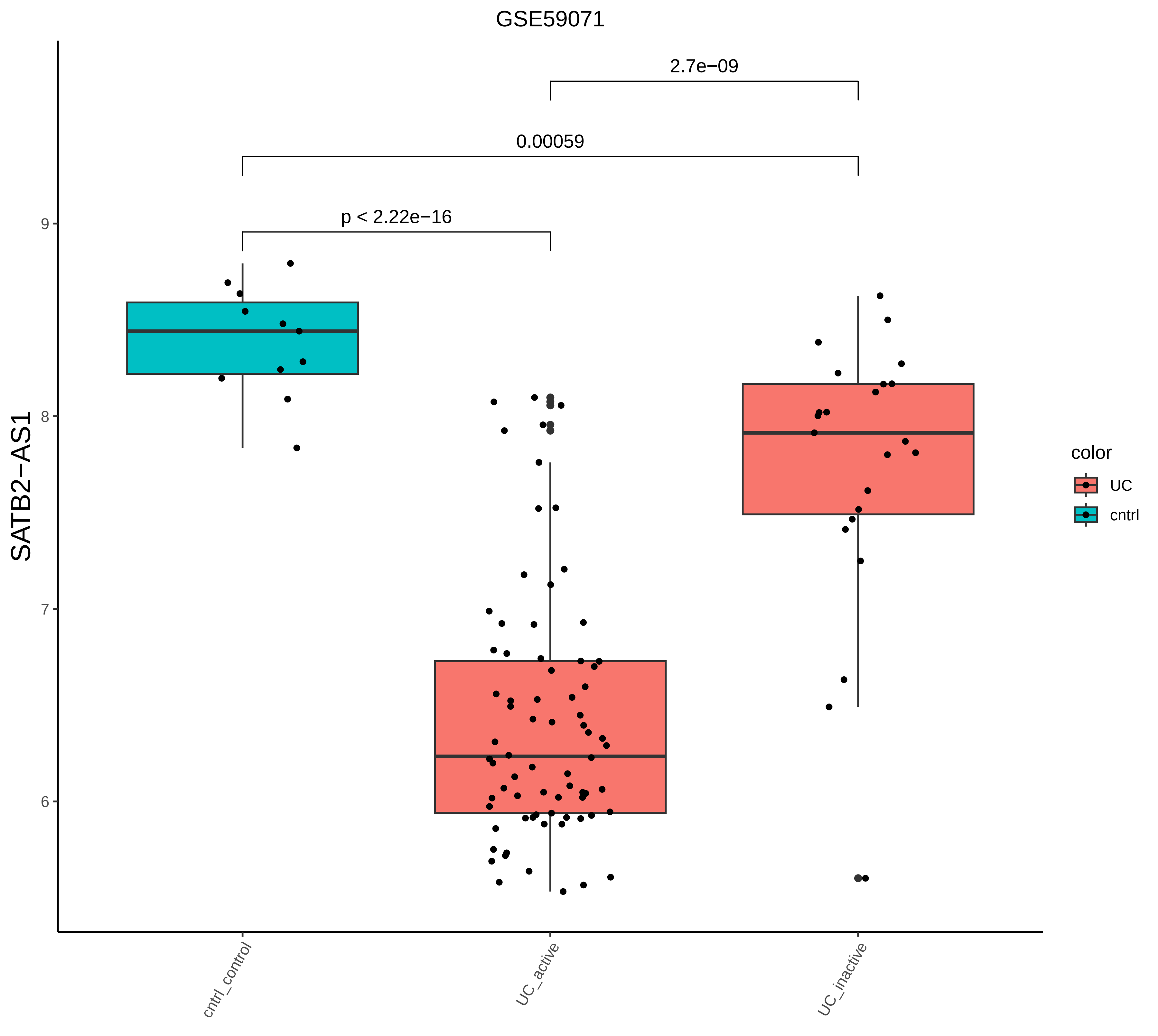
GSE59071
2.7e−09
0.00059
p < 2.22e−16
SATB2−AS1
9
8
7
6
cntrl_control
UC_active
UC_inactive
color
UC
cntrl

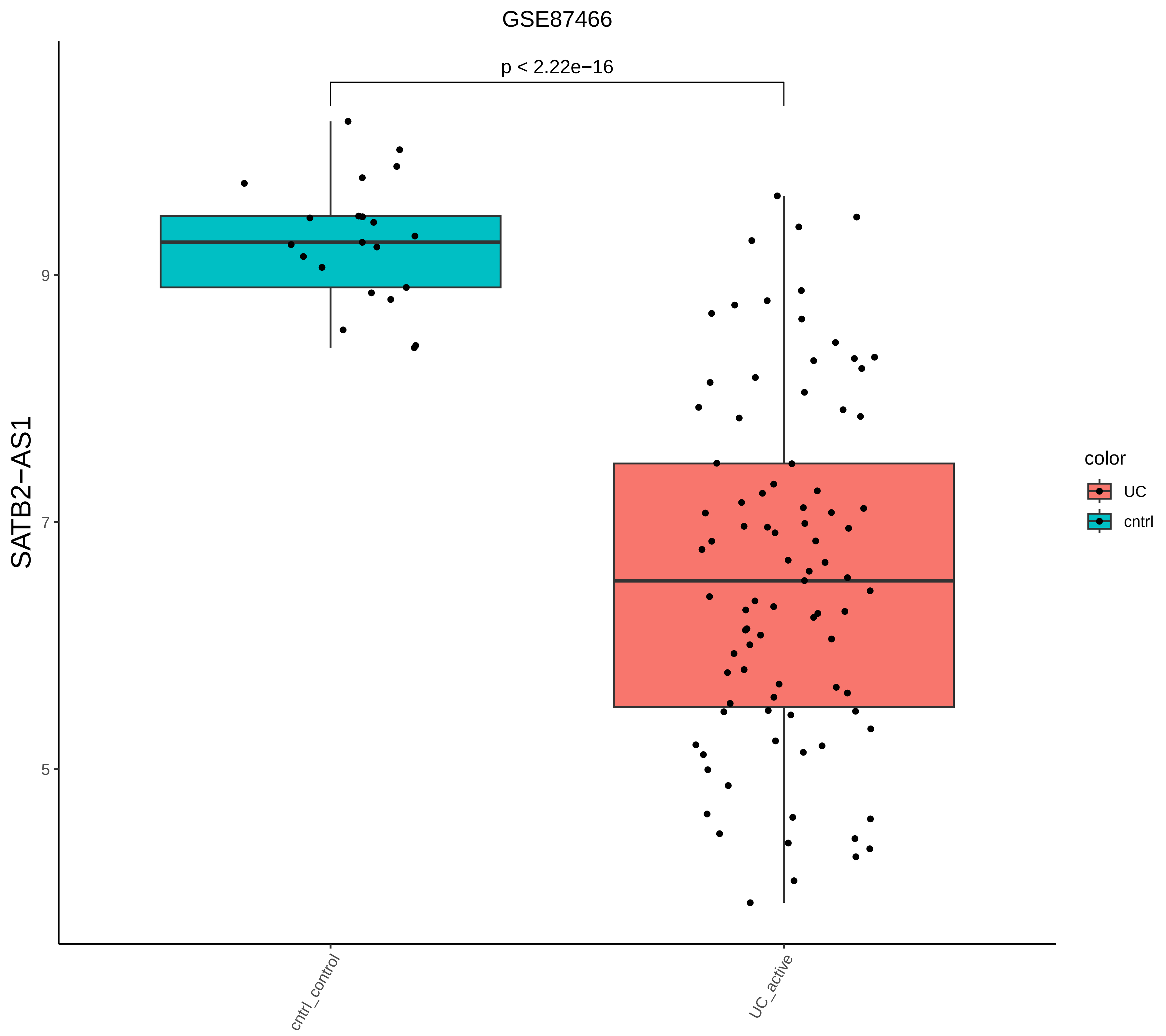
GSE87466
p < 2.22e−16
SATB2−AS1
9
7
5
cntrl_control
UC_active
color
UC
cntrl

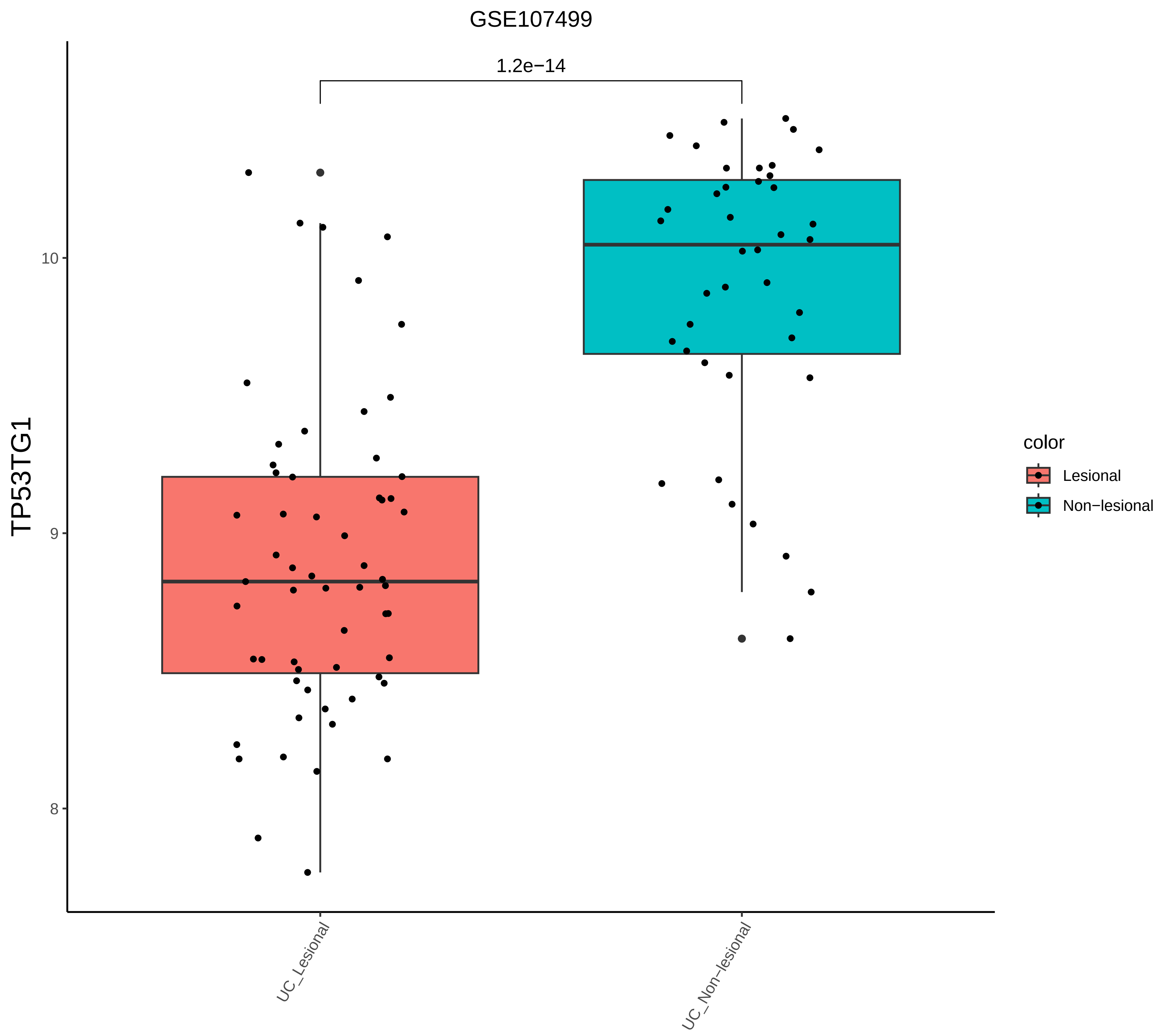

GSE107499
1.2e−14
TP53TG1
10
9
8
UC_Lesional
UC_Non−lesional
color
Lesional
Non−lesional

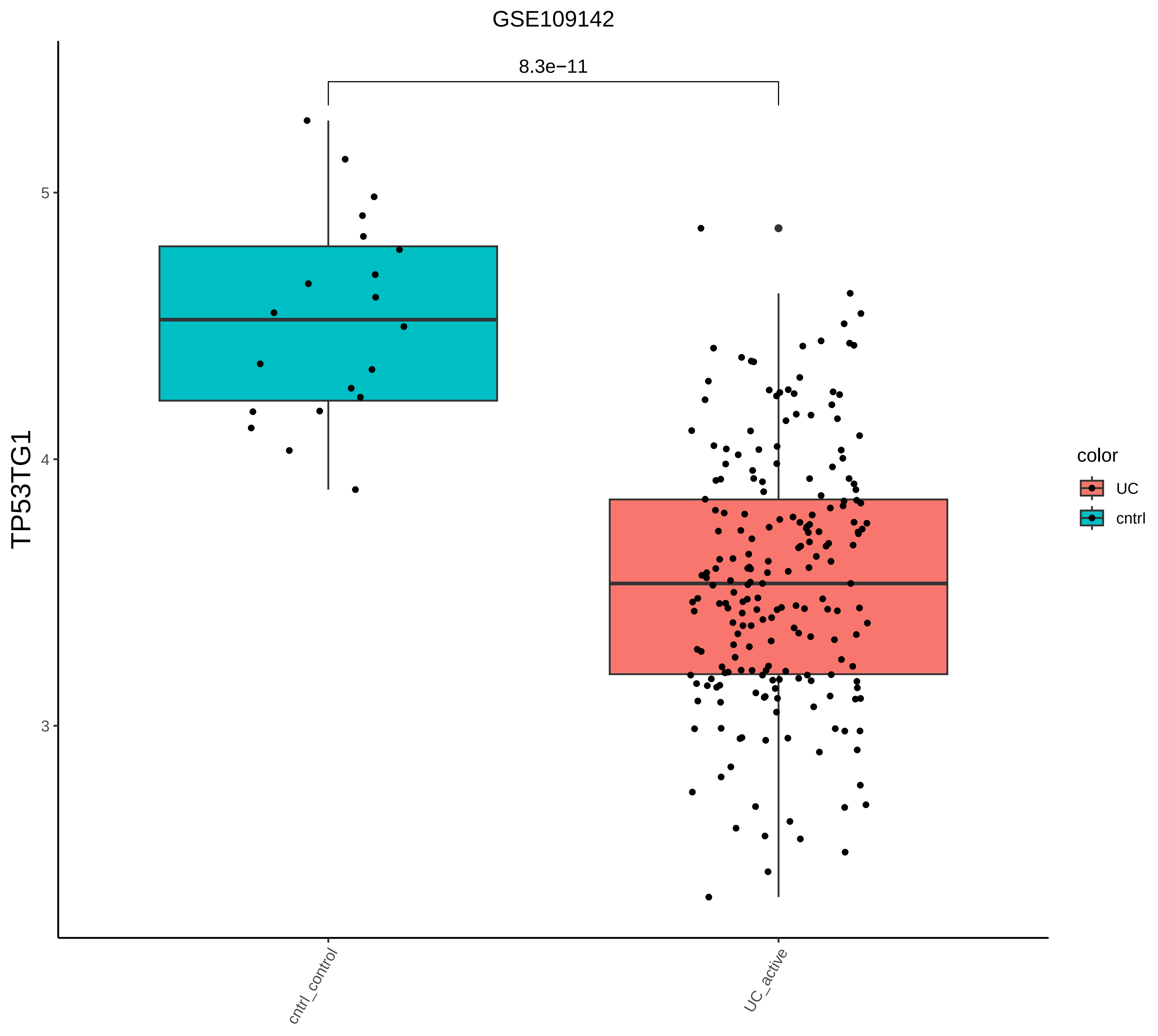

GSE109142
8.3e−11
TP53TG1
5
4
3
cntrl_control
UC_active
color
UC
cntrl

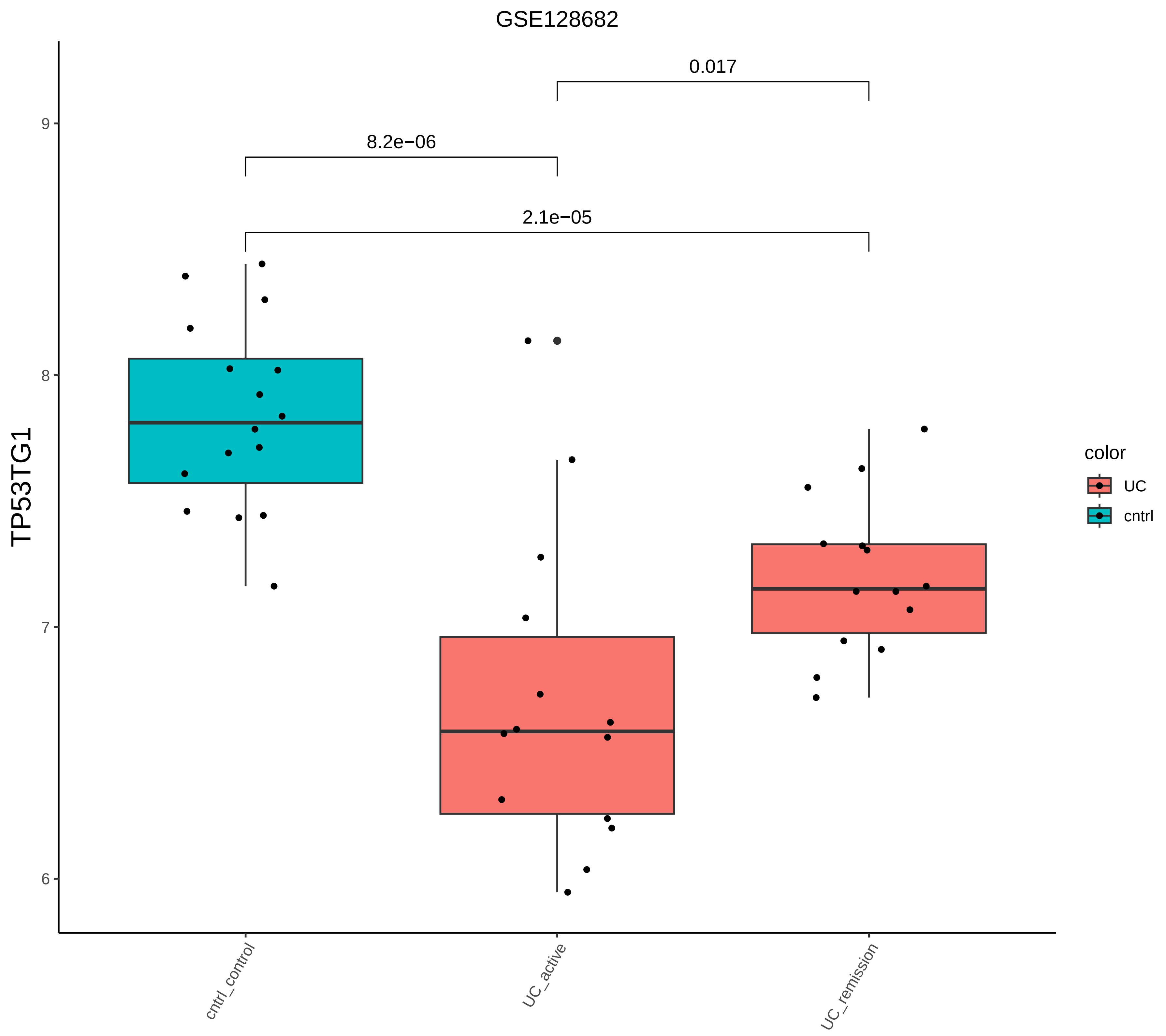
GSE128682
0.017
8.2e−06
2.1e−05
TP53TG1
9
8
7
6
cntrl_control
UC_active
UC_remission
color
UC
cntrl

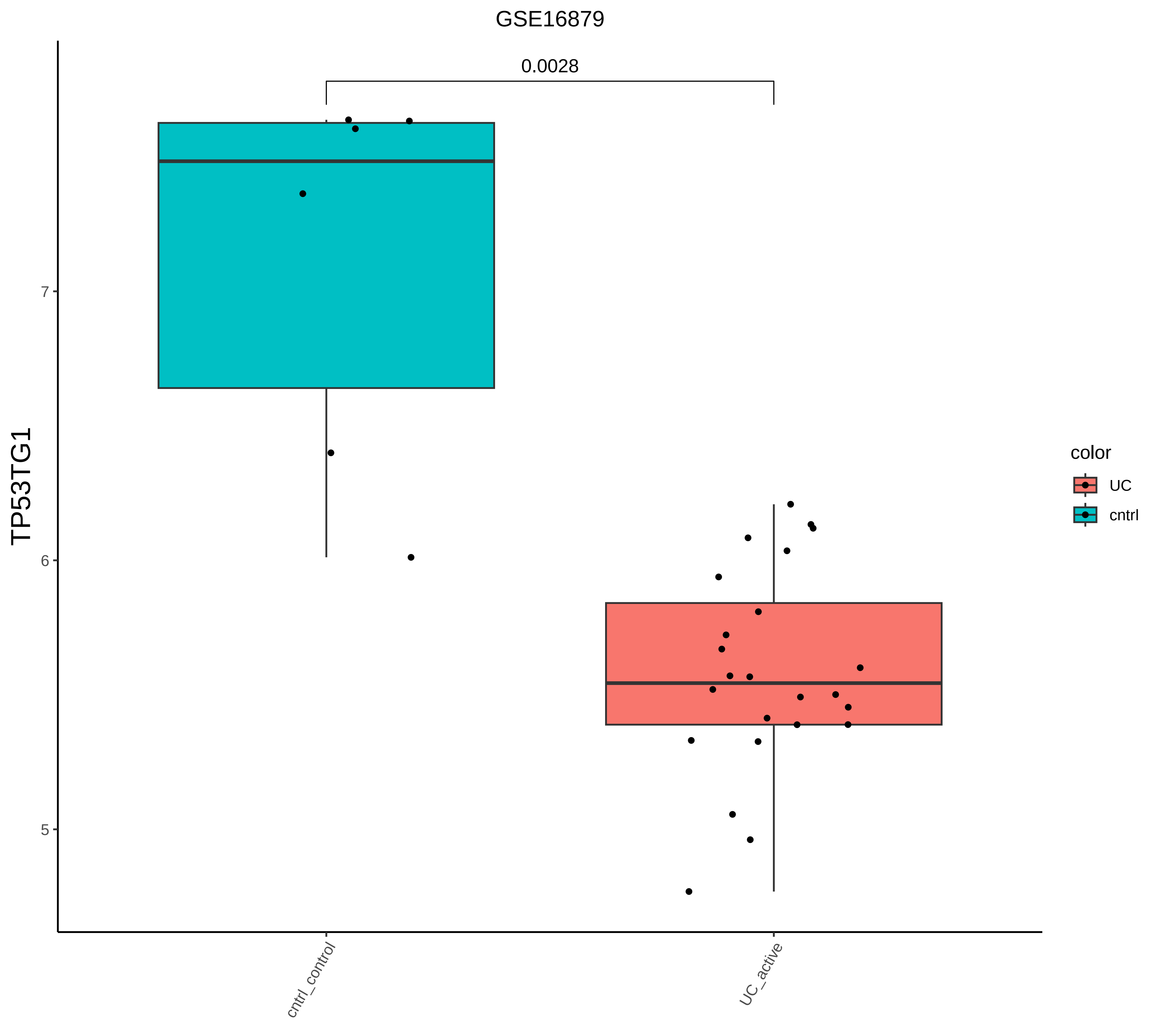

GSE16879
0.0028
TP53TG1
7
6
5
cntrl_control
UC_active
color
UC
cntrl

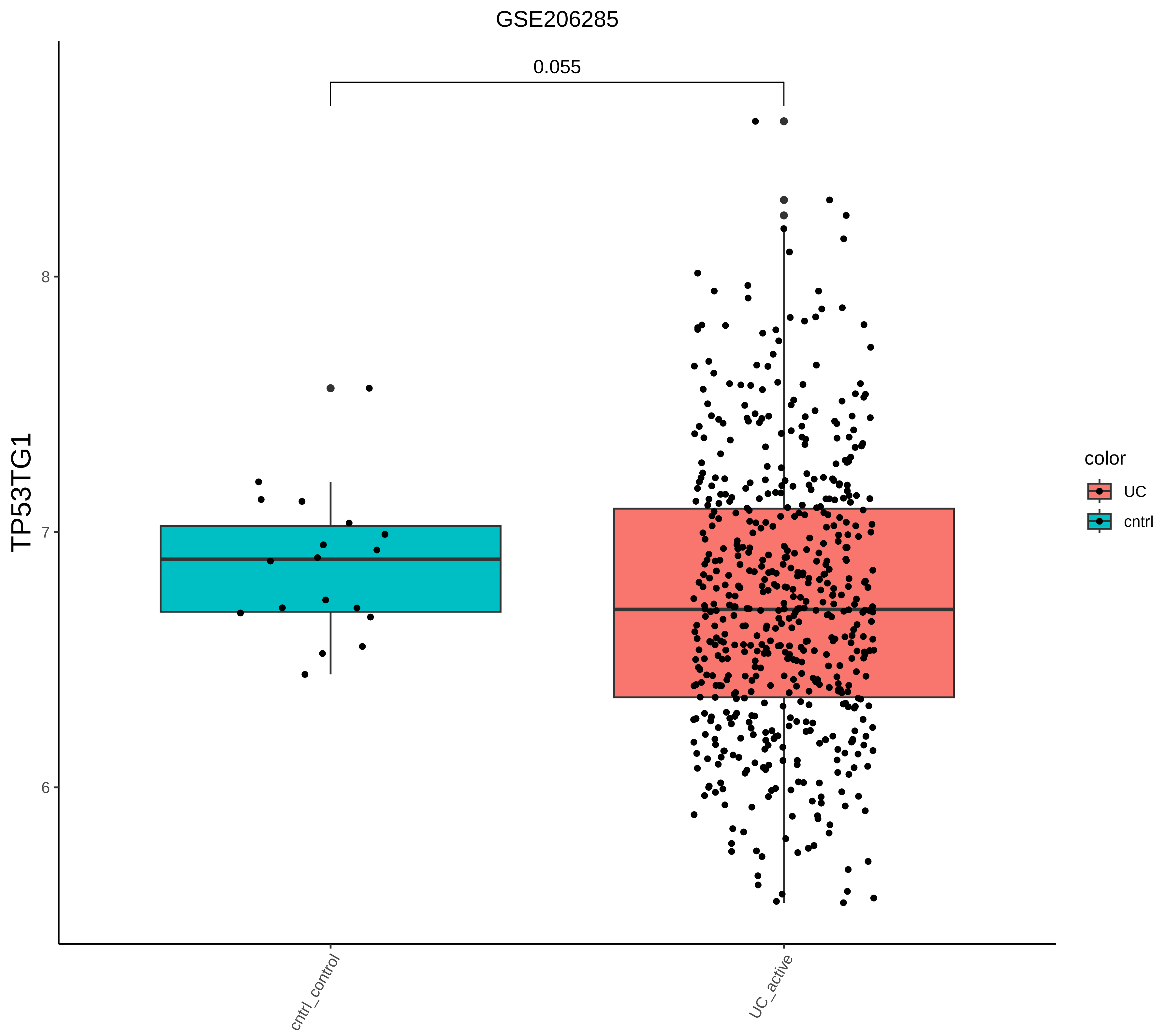
GSE206285
0.055
TP53TG1
8
7
6
cntrl_control
UC_active
color
UC
cntrl

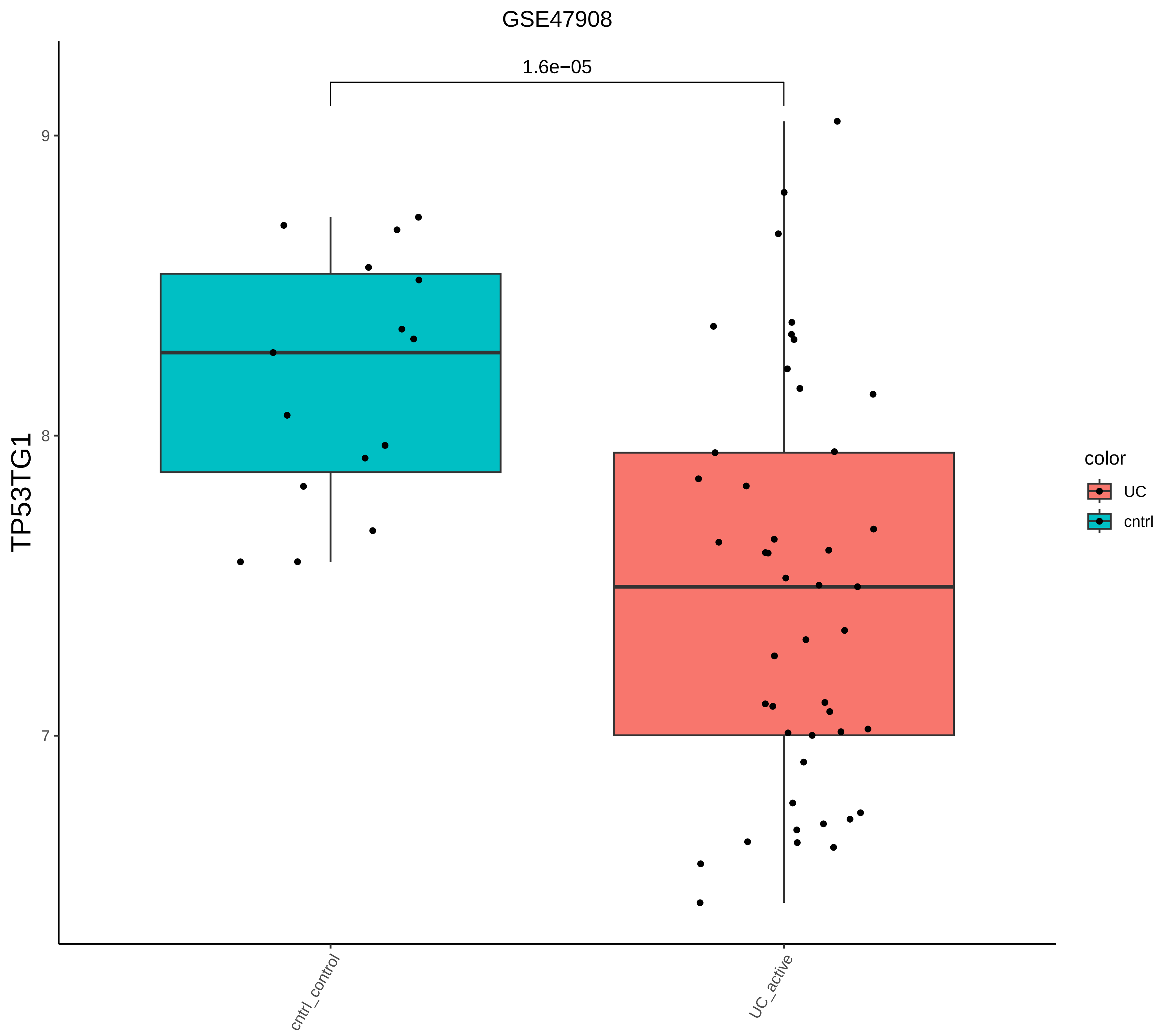
GSE47908
1.6e−05
TP53TG1
9
8
7
cntrl_control
UC_active
color
UC
cntrl

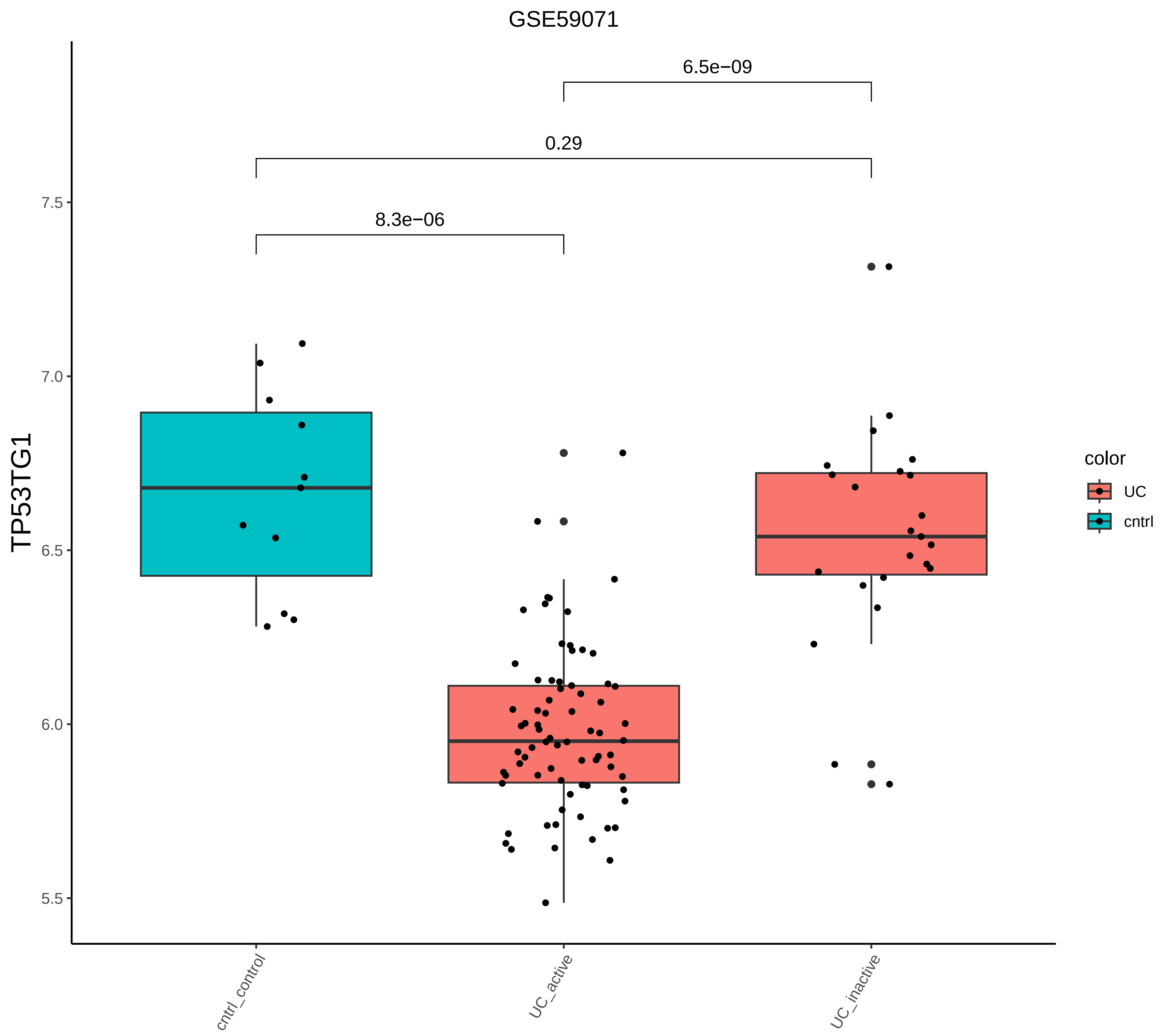
GSE59071
6.5e−09
0.29
8.3e−06
TP53TG1
7.5
7.0
6.5
6.0
5.5
cntrl_control
UC_active
UC_inactive
color
UC
cntrl

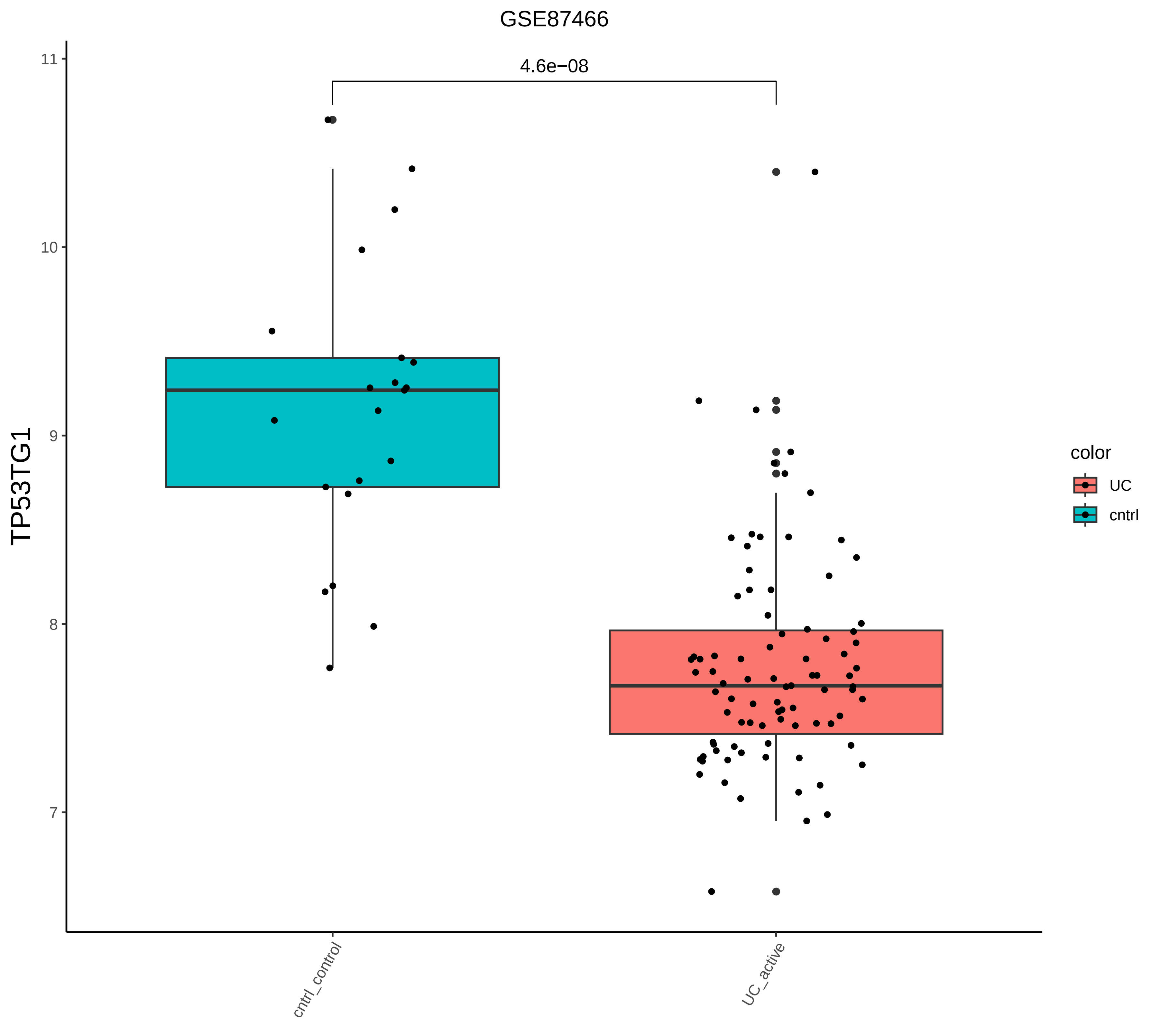

GSE87466
4.6e−08
TP53TG1
11
10
9
8
7
cntrl_control
UC_active
color
UC
cntrl

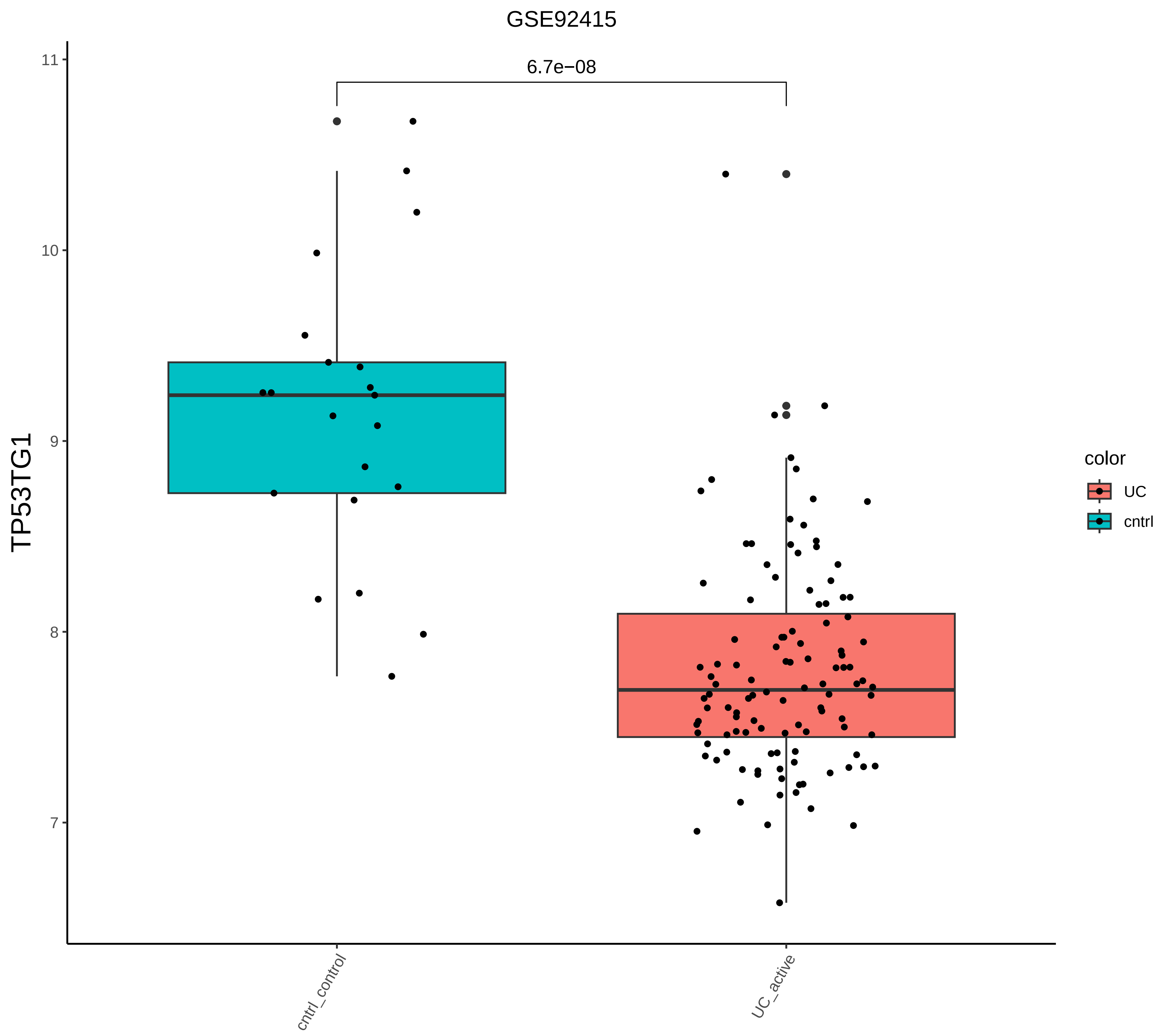

GSE92415
6.7e−08
TP53TG1
11
10
9
8
7
cntrl_control
UC_active
color
UC
cntrl

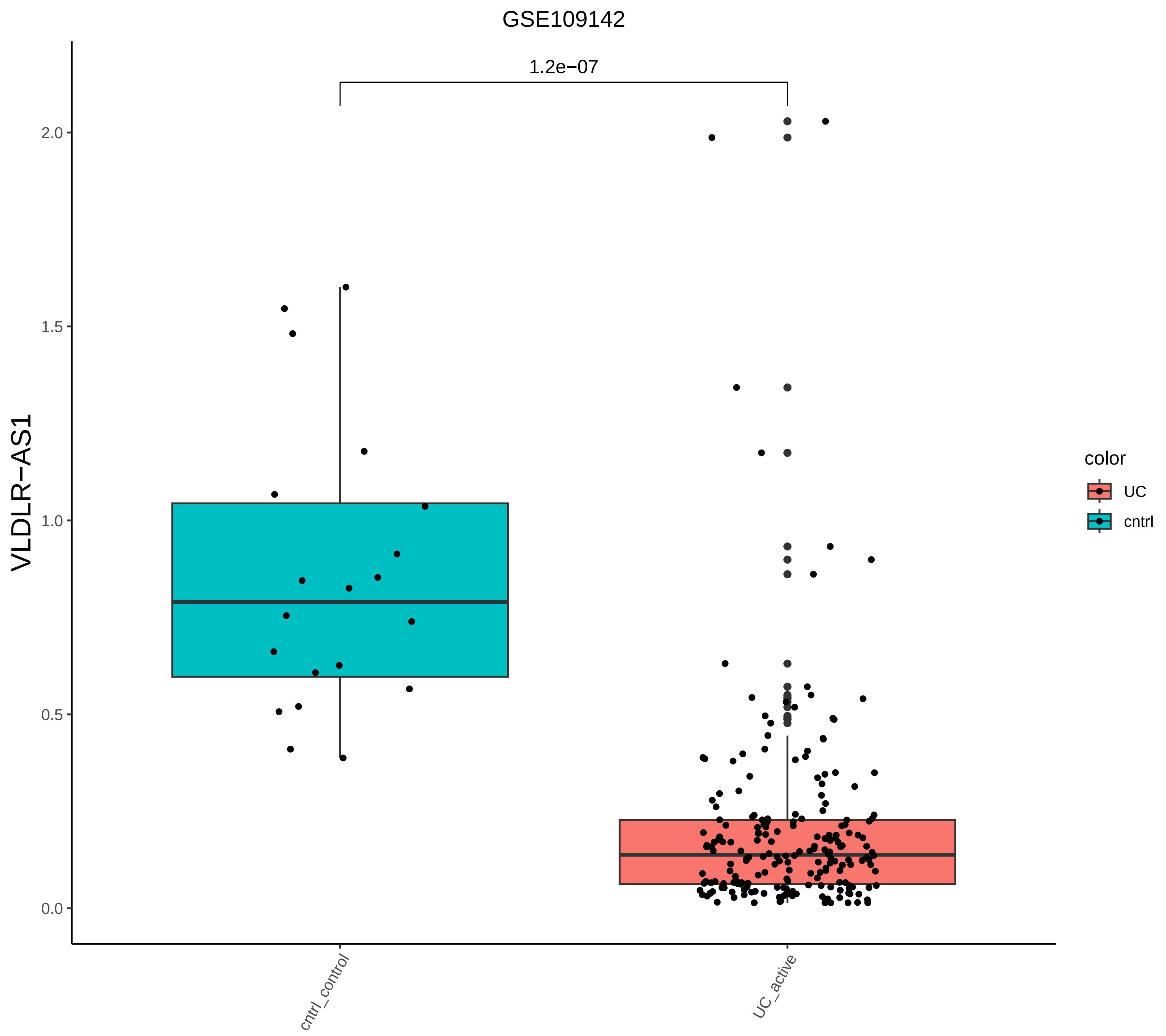
GSE109142
1.2e−07
VLDLR−AS1
2.0
1.5
1.0
0.5
0.0
cntrl_control
UC_active
color
UC
cntrl

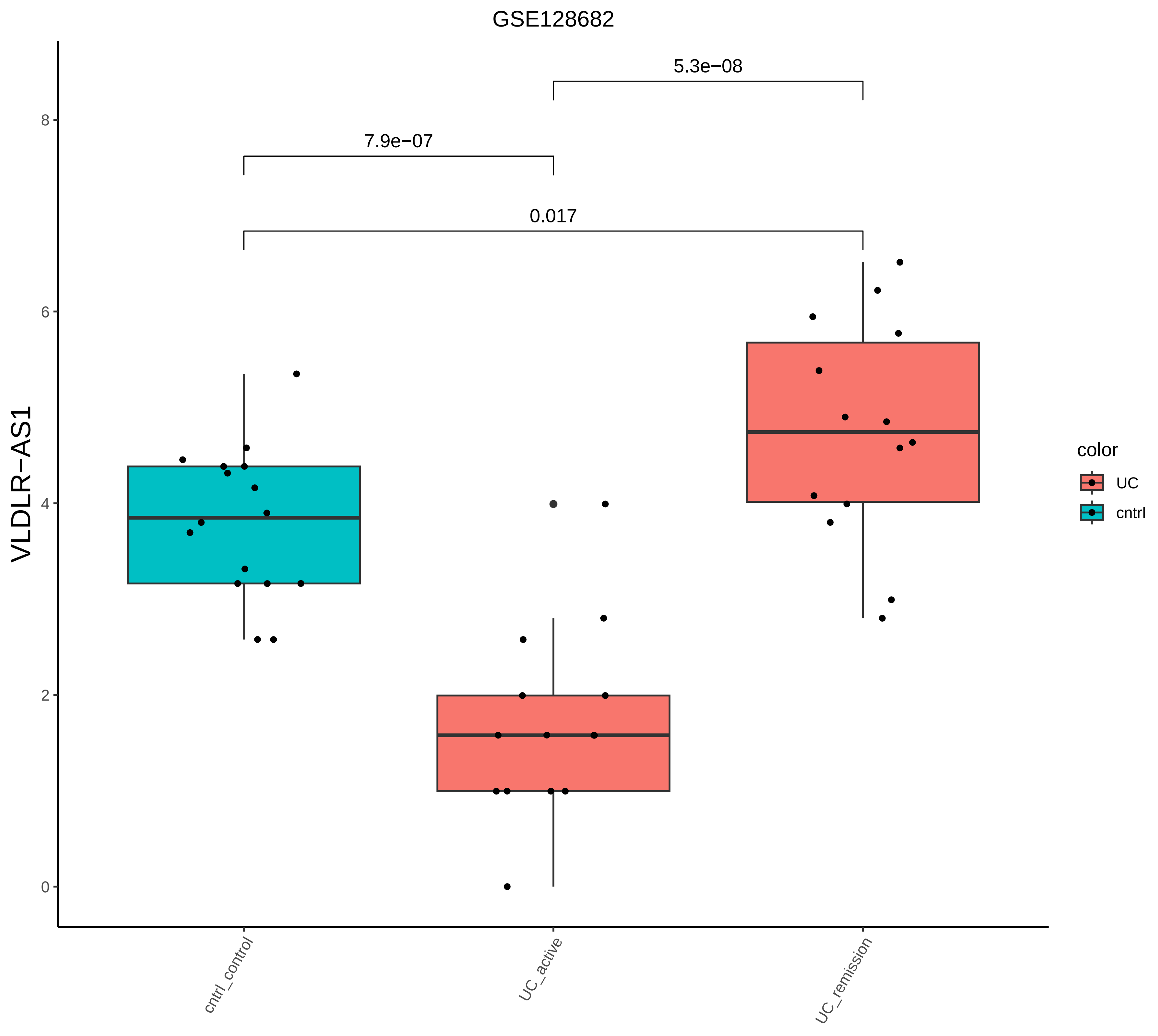
GSE128682
5.3e−08
7.9e−07
0.017
VLDLR−AS1
8
6
4
2
0
cntrl_control
UC_active
UC_remission
color
UC
cntrl

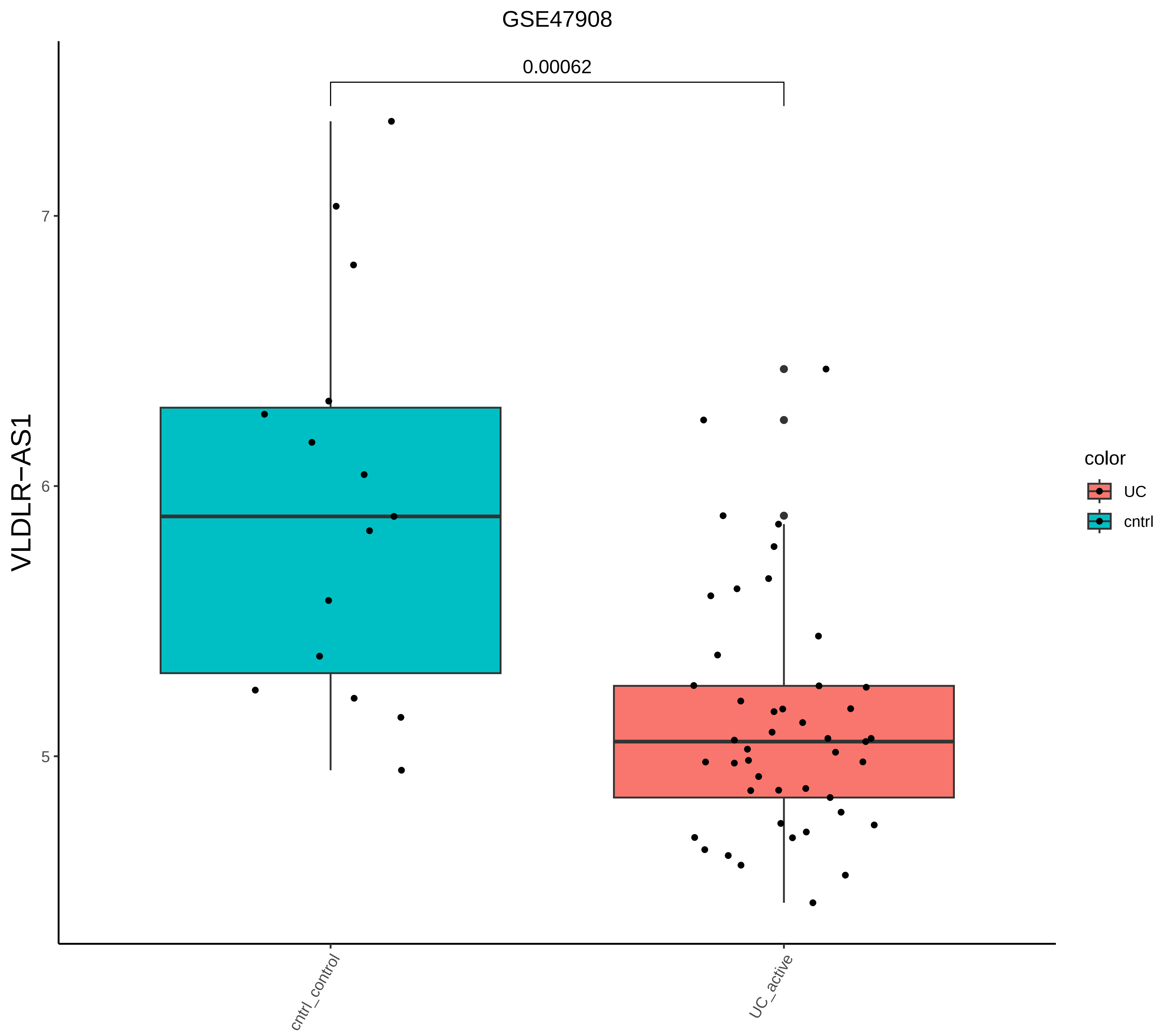
GSE47908
0.00062
VLDLR−AS1
7
6
5
cntrl_control
UC_active
color
UC
cntrl

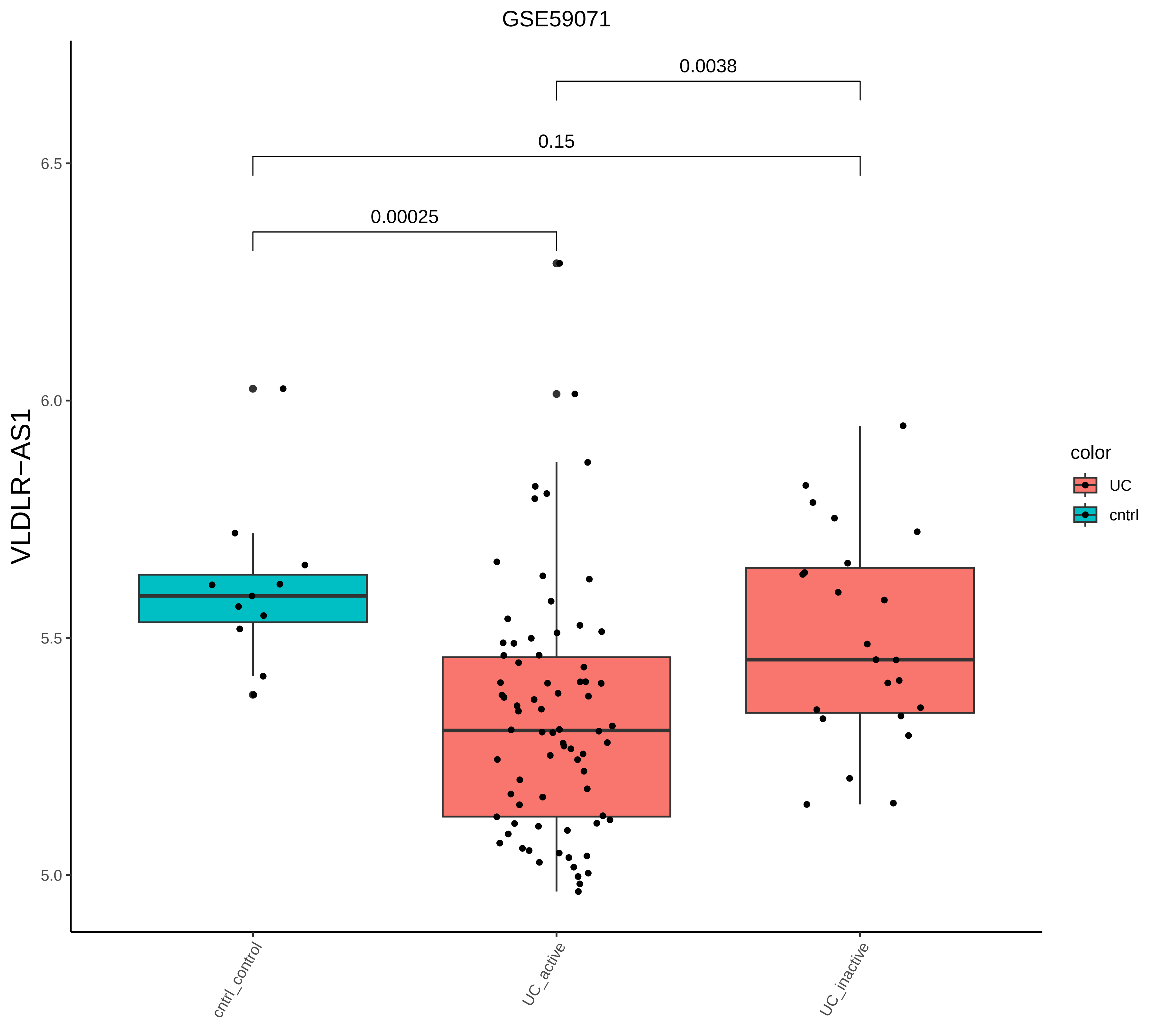

GSE59071
0.0038
0.15
0.00025
6.5
6.0
5.5
5.0
VLDLR−AS1
cntrl_control
UC_active
UC_inactive
color
UC
cntrl

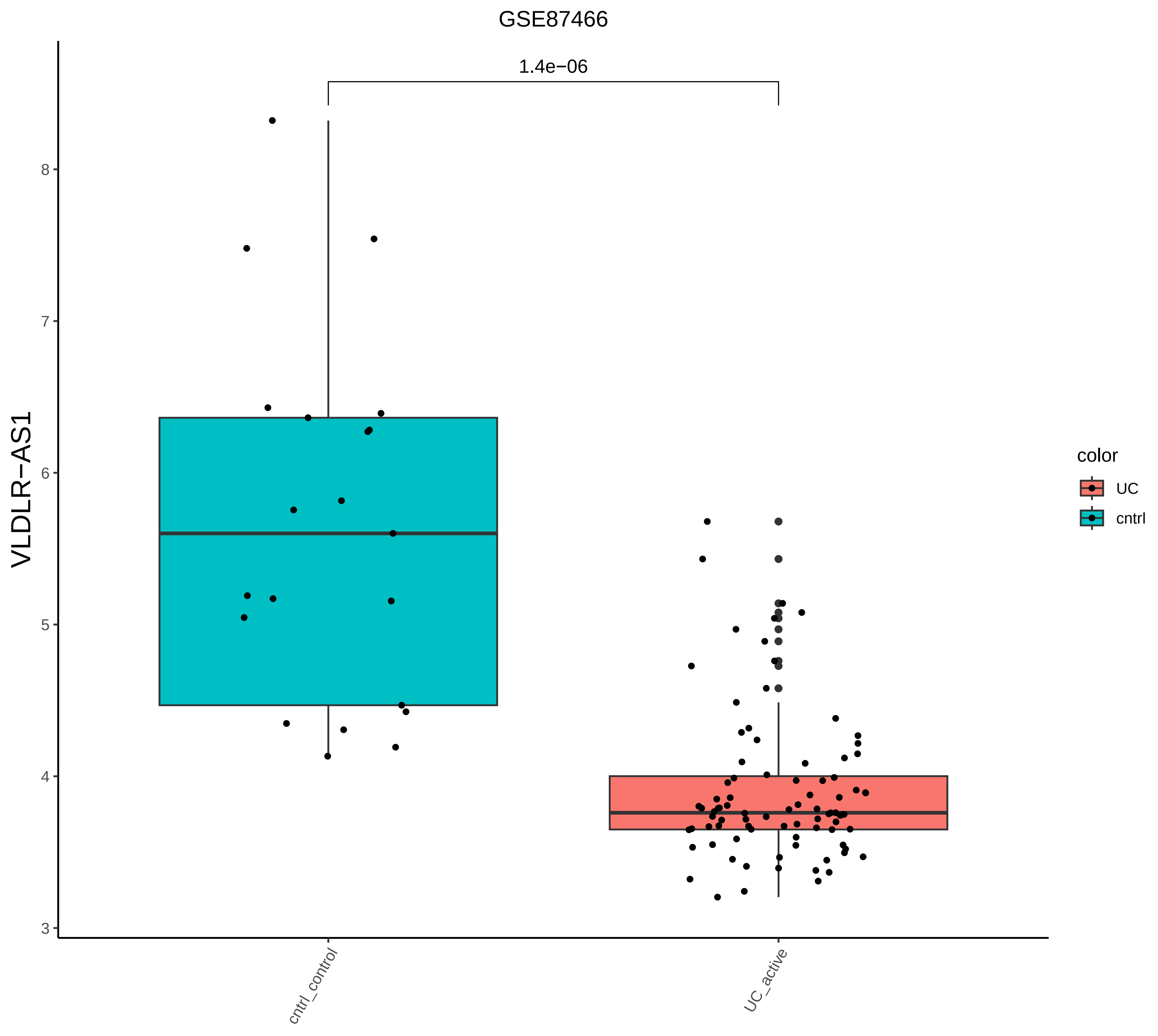
GSE87466
1.4e−06
VLDLR−AS1
8
7
6
5
4
3
cntrl_control
UC_active
color
UC
cntrl
