# Supplementary material for: Challenges in Defining a Reference Set of Differentially Expressed lncRNAs in Ulcerative Colitis by Meta-Analysis
Source: Curr Issues Mol Biol. 2024 Apr 5;46(4):3164–74. doi: 10.3390/cimb46040198 (PMC11049510; doi:10.3390/cimb46040198)
Supplement: Supplementary file 1 [file cimb-46-00198-s001.zip › Supplementary files/Figure S2.pdf]

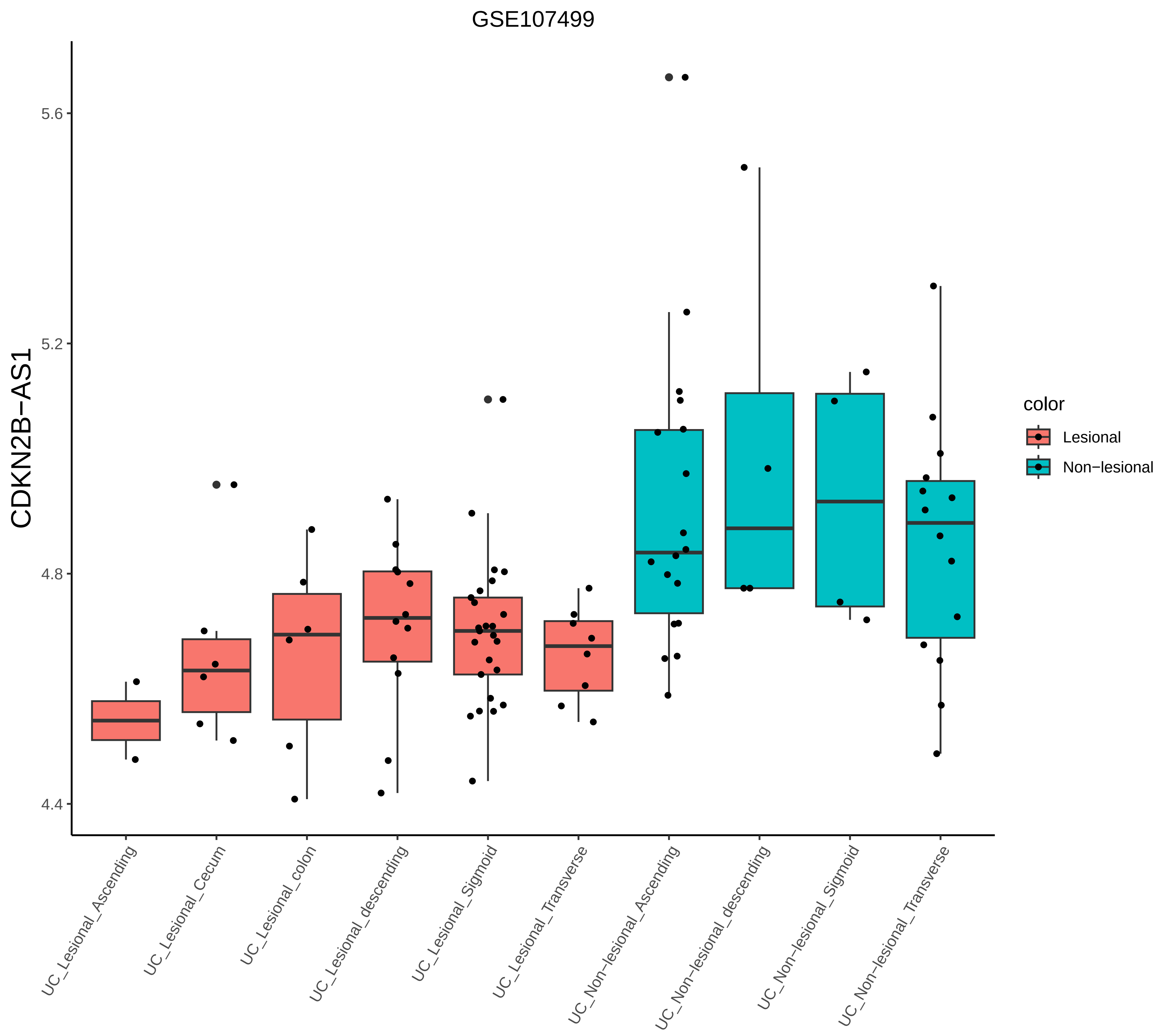
GSE107499
CDKN2B−AS1
5.6
5.2
4.8
4.4
UC_Lesional_Ascending
UC_Lesional_Cecum
UC_Lesional_colon
UC_Lesional_descending
UC_Lesional_Sigmoid
UC_Lesional_Transverse
UC_Non−lesional_Ascending
UC_Non−lesional_descending
UC_Non−lesional_Sigmoid
UC_Non−lesional_Transverse
color
Lesional
Non−lesional

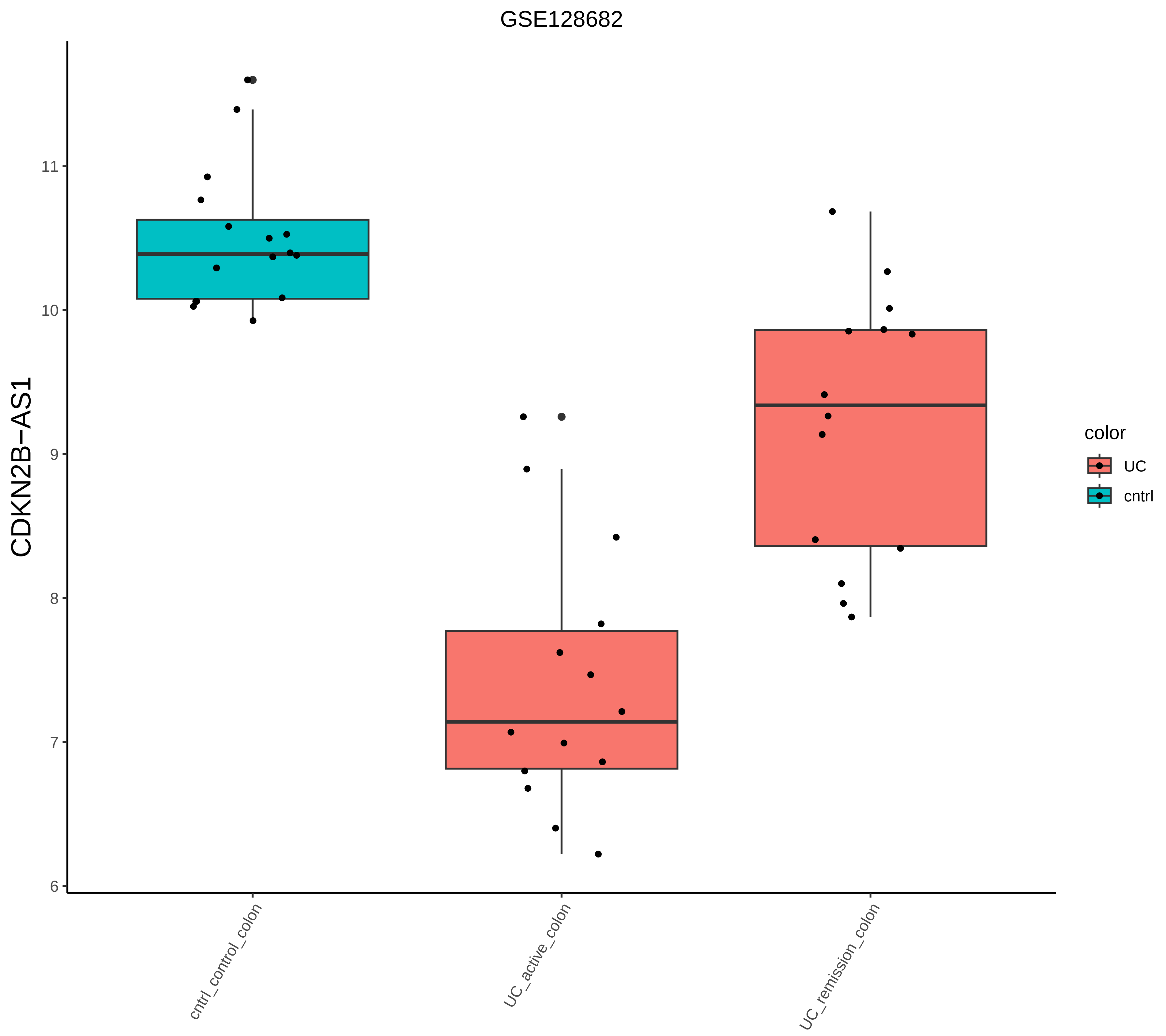

GSE128682
CDKN2B−AS1
11
10
9
8
7
6
cntrl_control_colon
UC_active_colon
UC_remission_colon
color
UC
cntrl

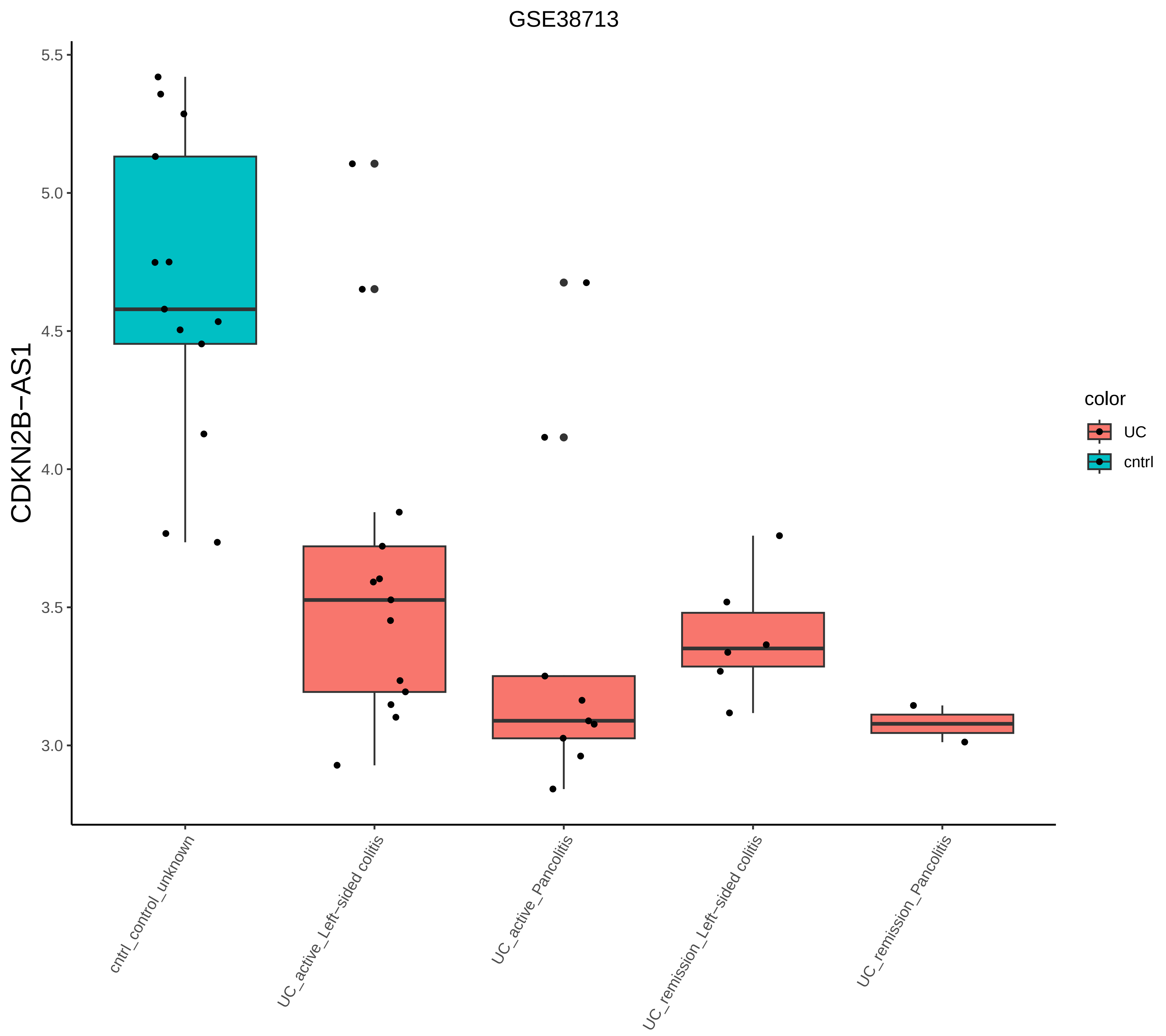

GSE38713
CDKN2B−AS1
5.5
5.0
4.5
4.0
3.5
3.0
cntrl_control_unknown
UC_active_Left−sided colitis
UC_active_Pancolitis
UC_remission_Left−sided colitis
UC_remission_Pancolitis
color
UC
cntrl

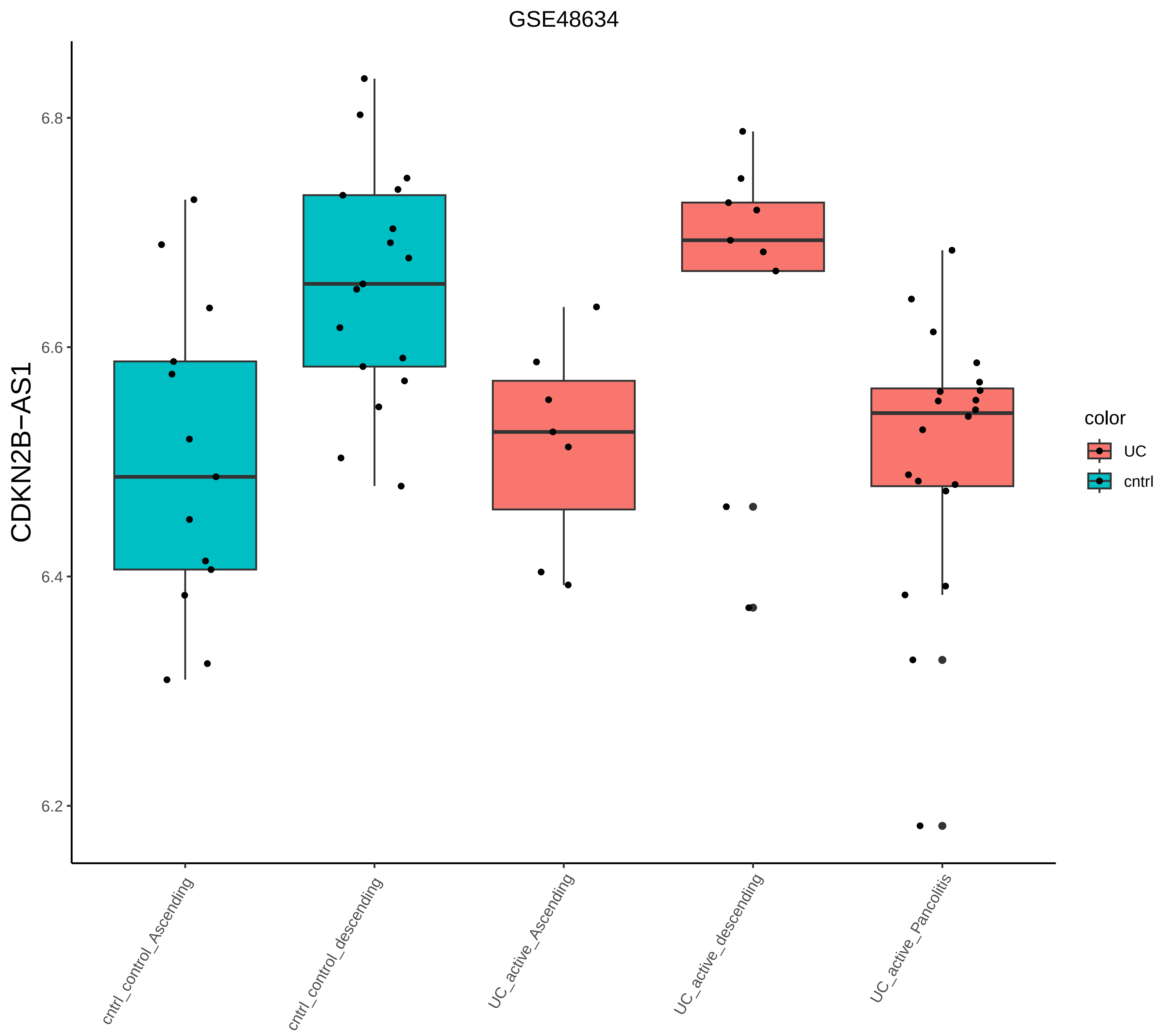

GSE48634
CDKN2B−AS1
6.8
6.6
6.4
6.2
cntrl_control_Ascending
cntrl_control_descending
UC_active_Ascending
UC_active_descending
UC_active_Pancolitis
color
UC
cntrl

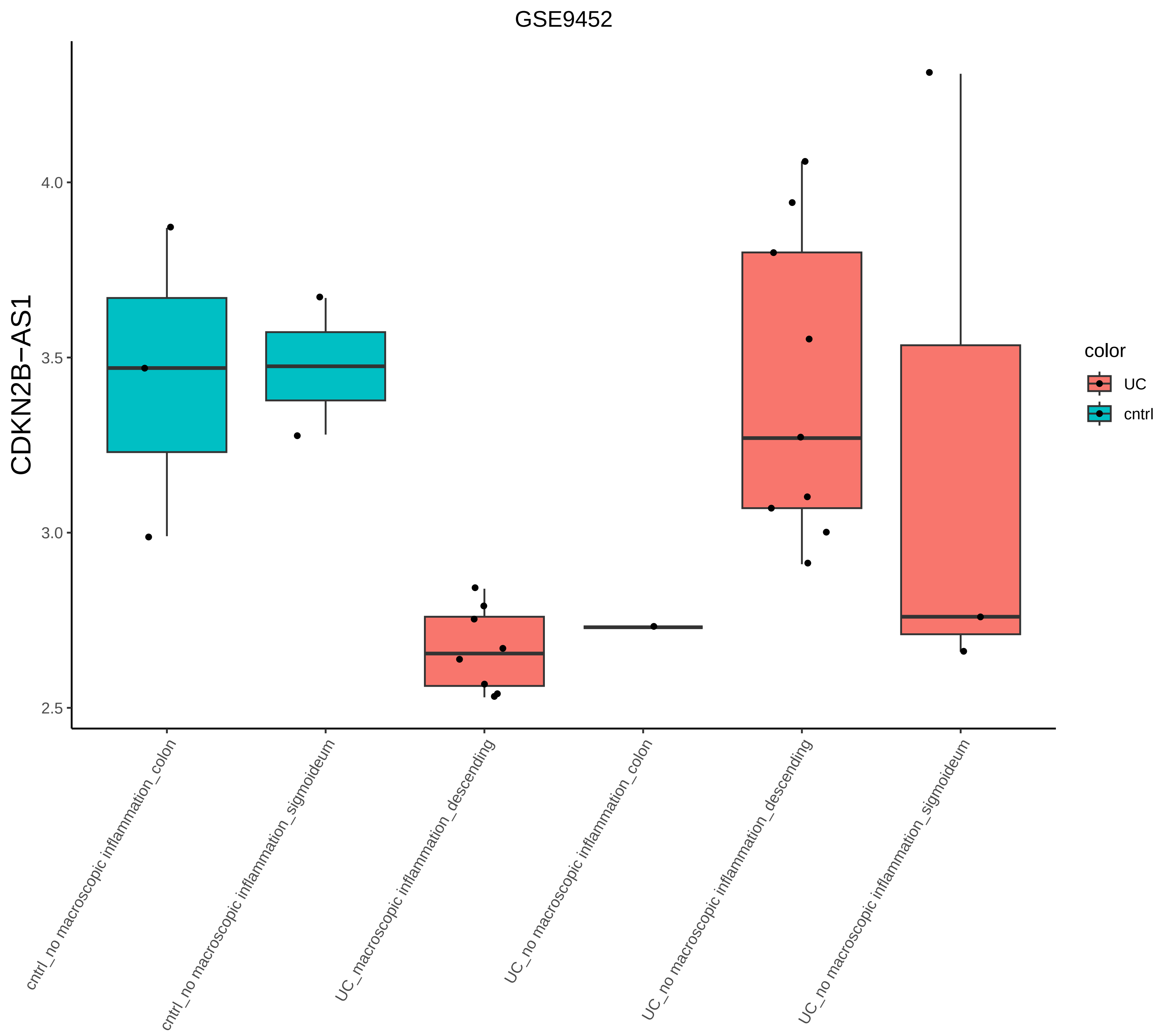

GSE9452
CDKN2B−AS1
4.0
3.5
3.0
2.5
cntrl_no macroscopic inflammation_colon
cntrl_no macroscopic inflammation_sigmoideum
UC_macroscopic inflammation_descending
UC_no macroscopic inflammation_colon
UC_no macroscopic inflammation_descending
UC_no macroscopic inflammation_sigmoideum
color
UC
cntrl

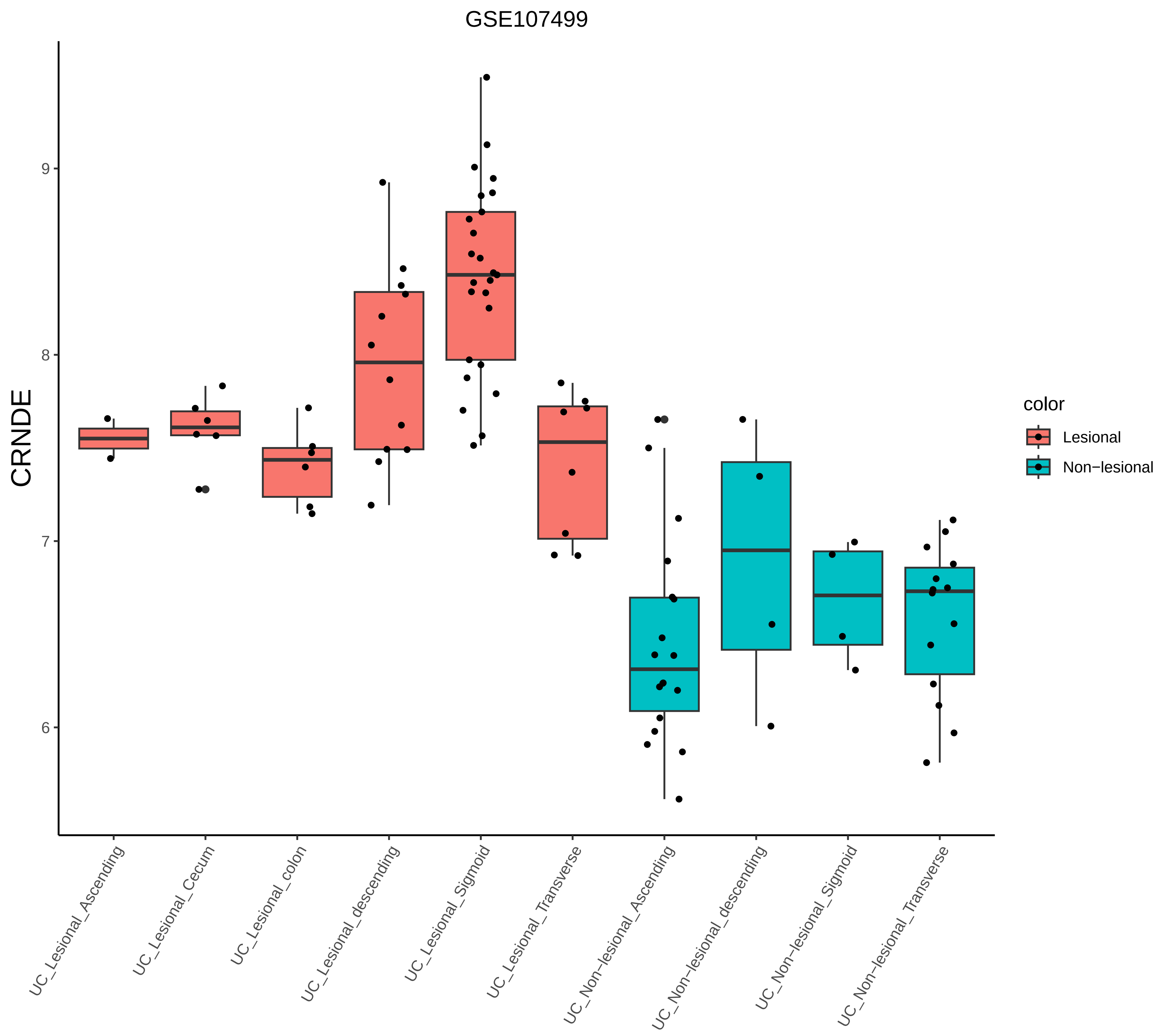
GSE107499
CRNDE
9
8
7
6
color
Lesional
Non−lesional
UC_Lesional_Ascending
UC_Lesional_Cecum
UC_Lesional_colon
UC_Lesional_descending
UC_Lesional_Sigmoid
UC_Lesional_Transverse
UC_Non−lesional_Ascending
UC_Non−lesional_descending
UC_Non−lesional_Sigmoid
UC_Non−lesional_Transverse

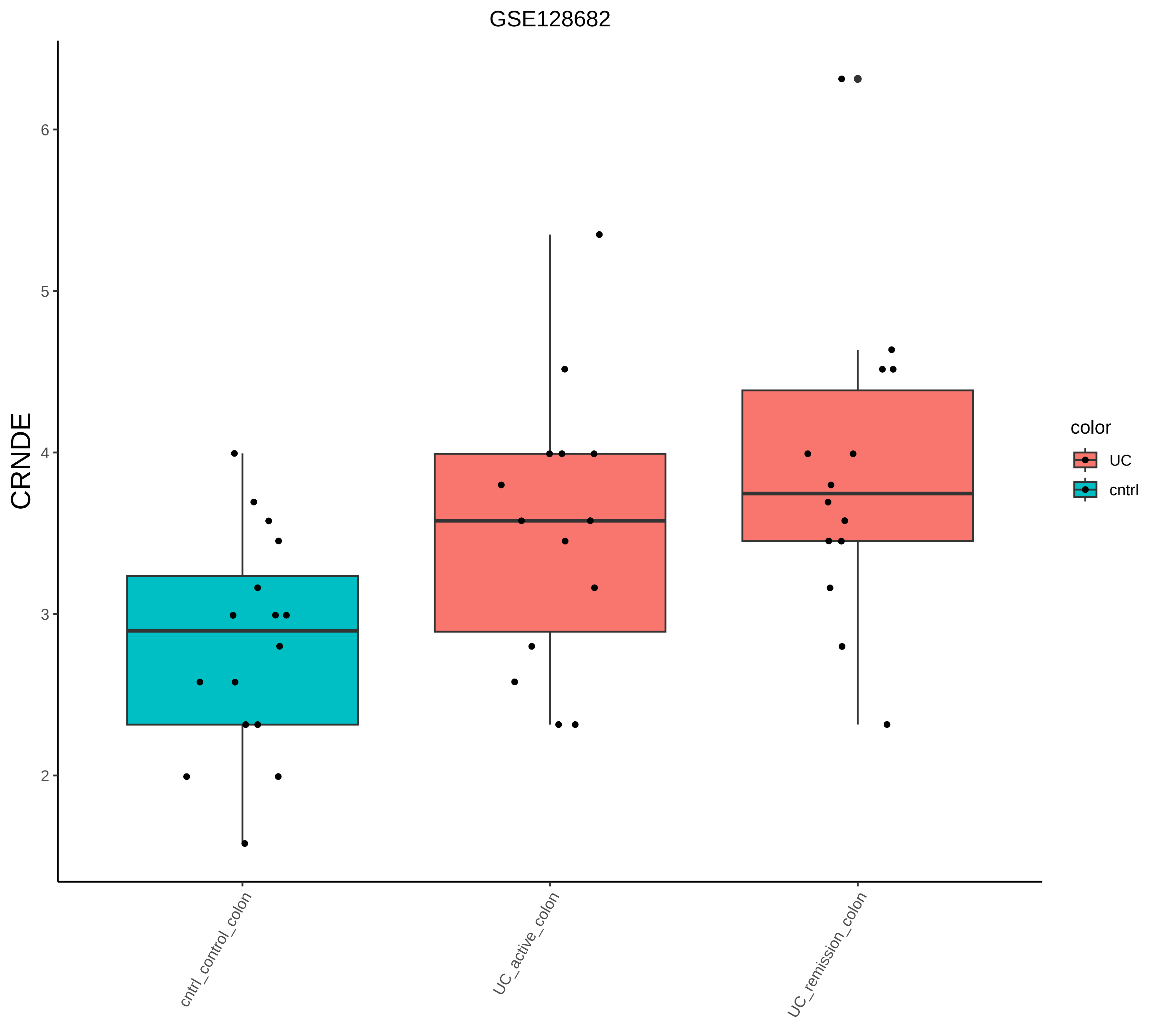
GSE128682
CRNDE
6
5
4
3
2
cntrl_control_colon
UC_active_colon
UC_remission_colon
color
UC
cntrl

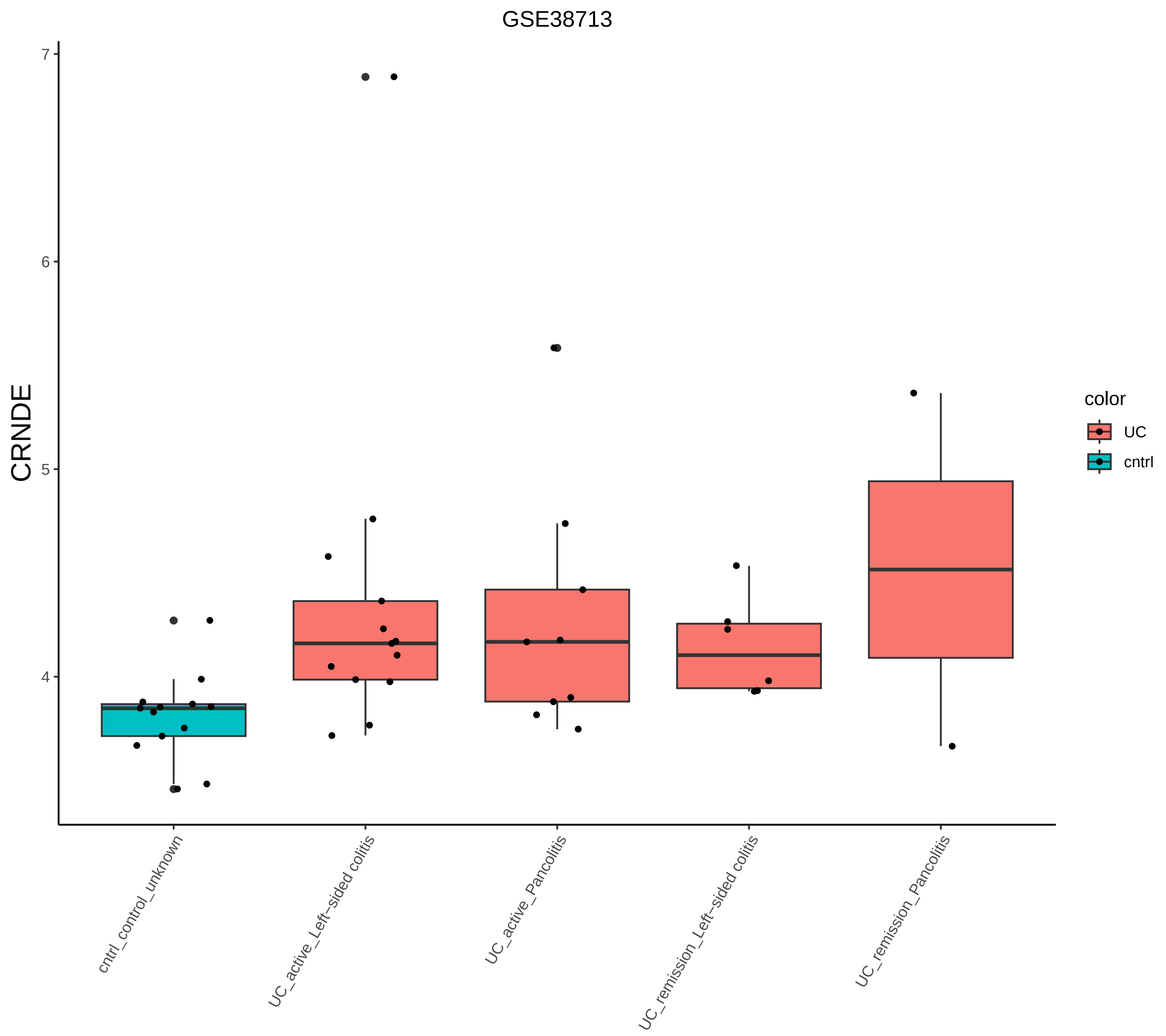

GSE38713
CRNDE
7
6
5
4
cntrl_control_unknown
UC_active_Left−sided colitis
UC_active_Pancolitis
UC_remission_Left−sided colitis
UC_remission_Pancolitis
color
UC
cntrl

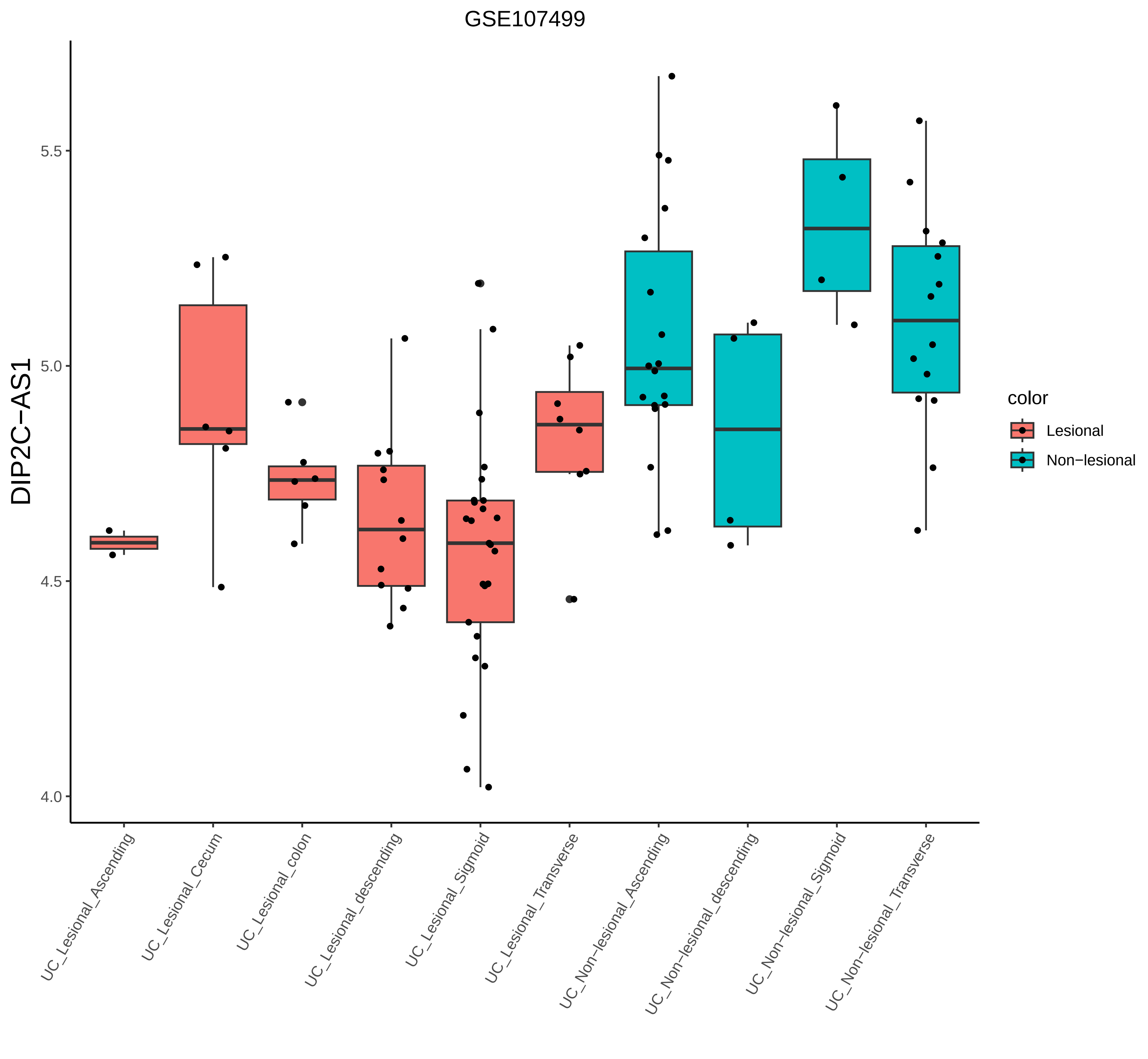

GSE107499
DIP2C−AS1
5.5
5.0
4.5
4.0
UC_Lesional_Ascending
UC_Lesional_Cecum
UC_Lesional_colon
UC_Lesional_descending
UC_Lesional_Sigmoid
UC_Lesional_Transverse
UC_Non−lesional_Ascending
UC_Non−lesional_descending
UC_Non−lesional_Sigmoid
UC_Non−lesional_Transverse
color
Lesional
Non−lesional

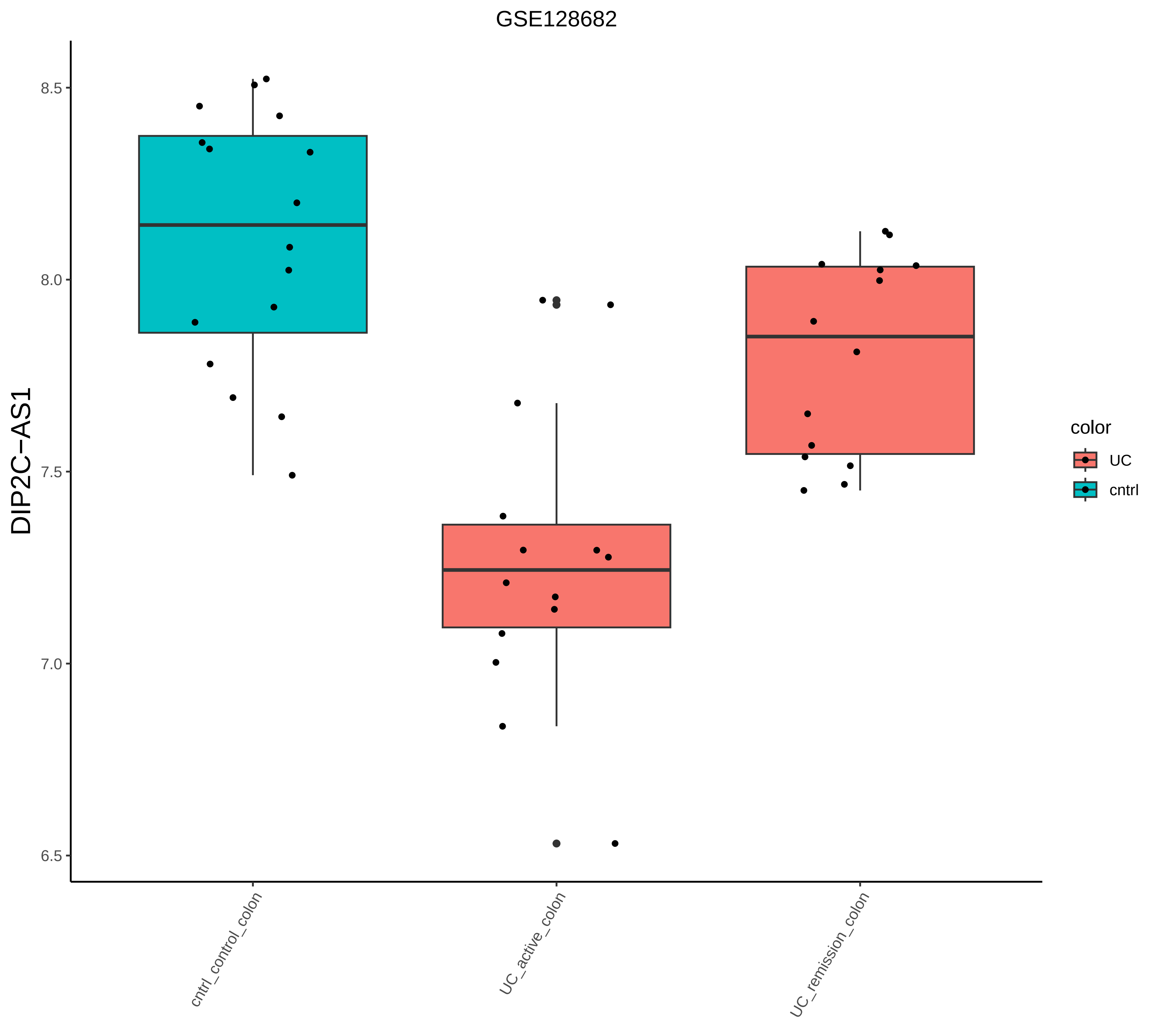

GSE128682
DIP2C−AS1
8.5
8.0
7.5
7.0
6.5
cntrl_control_colon
UC_active_colon
UC_remission_colon
color
UC
cntrl

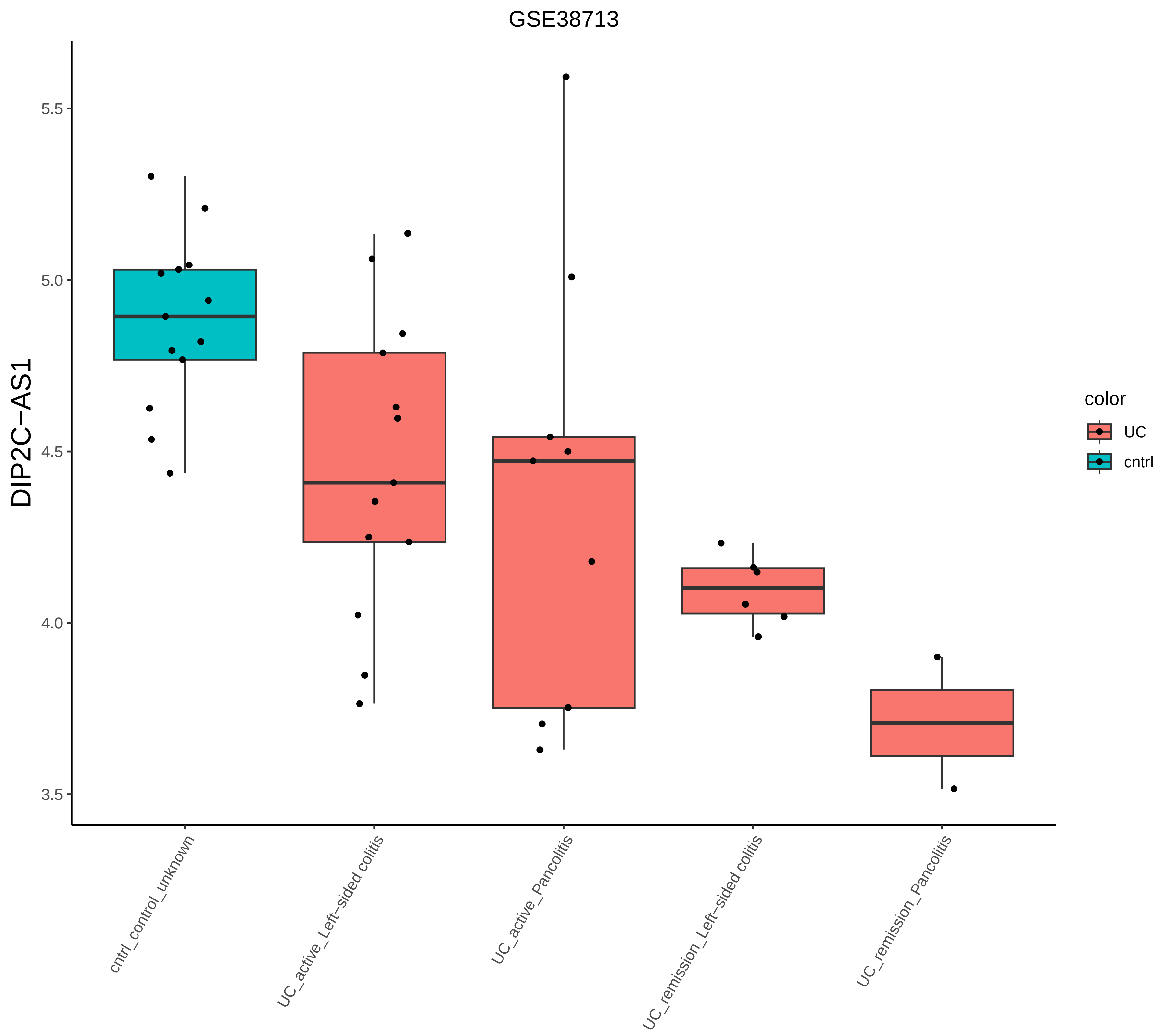

GSE38713
DIP2C−AS1
5.5
5.0
4.5
4.0
3.5
cntrl_control_unknown
UC_active_Left−sided colitis
UC_active_Pancolitis
UC_remission_Left−sided colitis
UC_remission_Pancolitis
color
UC
cntrl

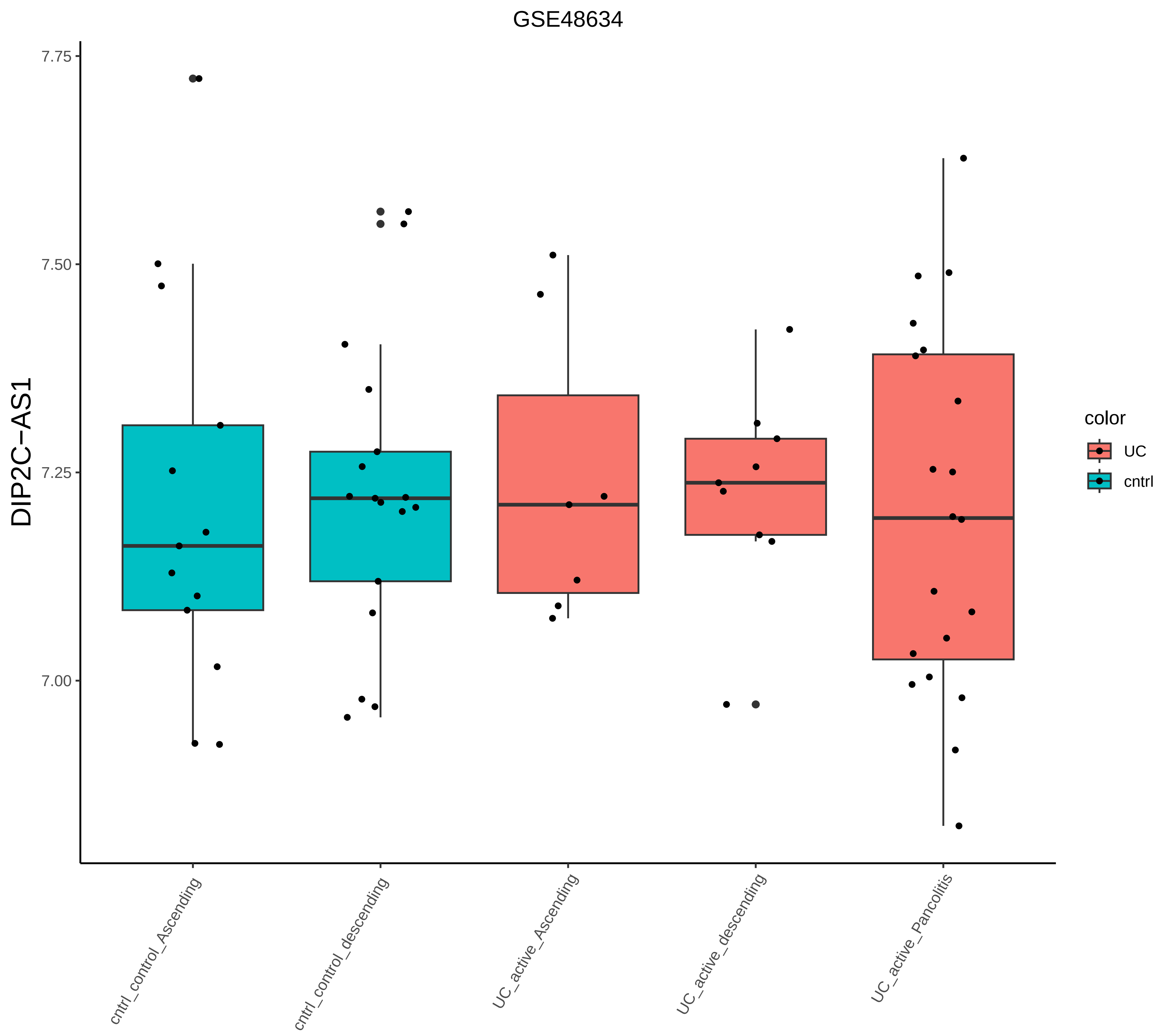

GSE48634
DIP2C−AS1
7.75
7.50
7.25
7.00
cntrl_control_Ascending
cntrl_control_descending
UC_active_Ascending
UC_active_descending
UC_active_Pancolitis
color
UC
cntrl

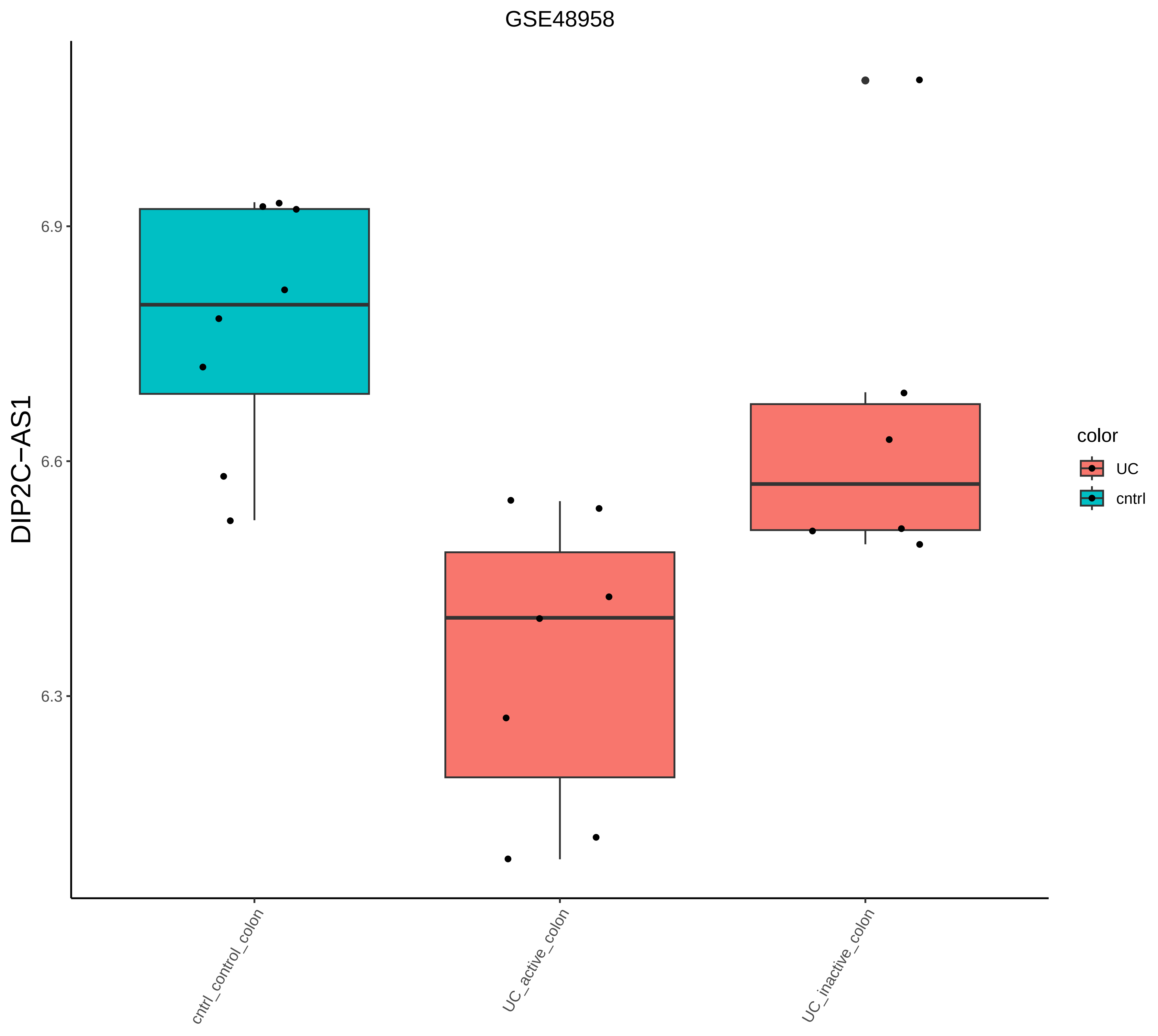

GSE48958
DIP2C−AS1
6.9
6.6
6.3
cntrl_control_colon
UC_active_colon
UC_inactive_colon
color
UC
cntrl

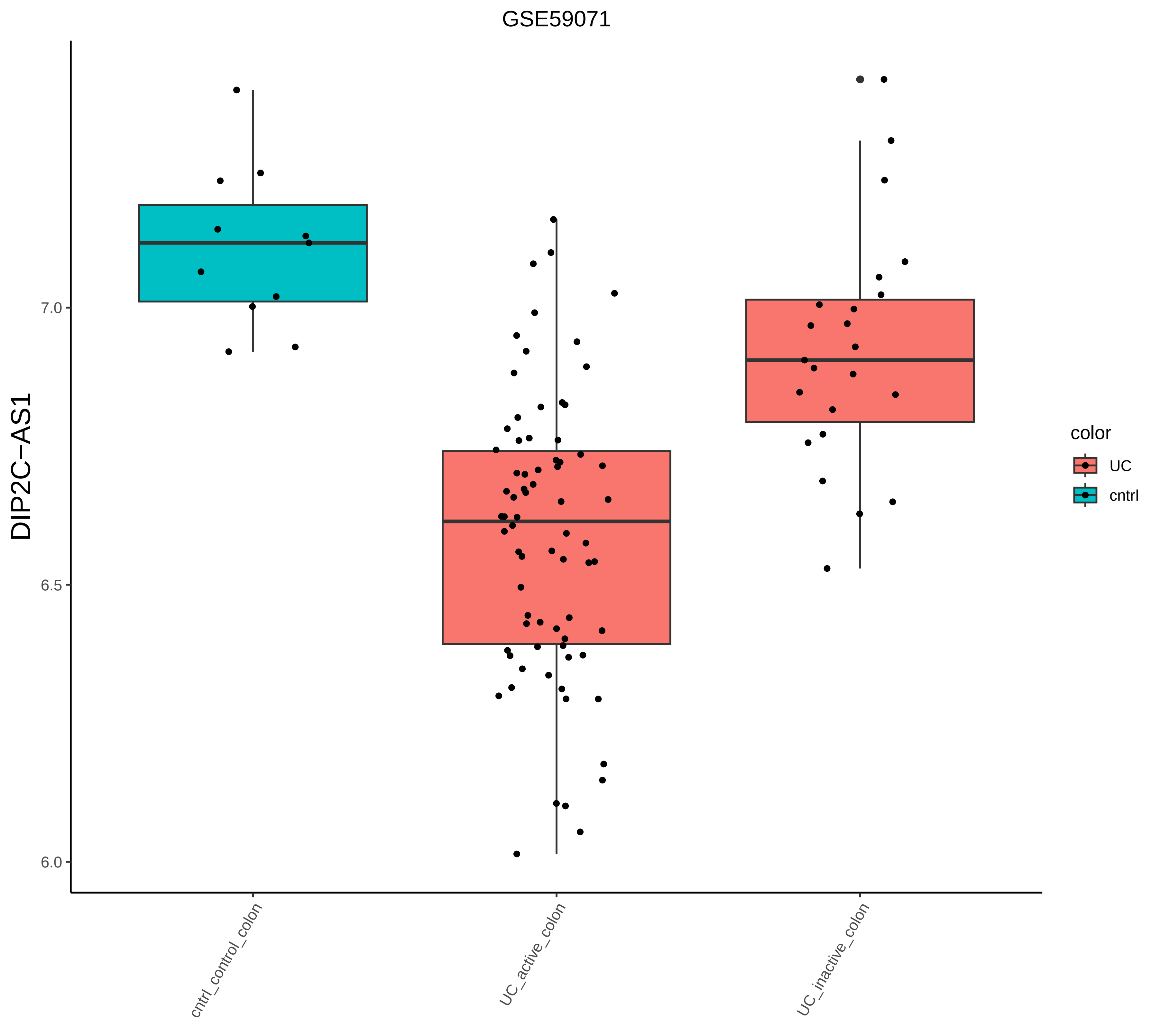

GSE59071
DIP2C−AS1
7.0
6.5
6.0
cntrl_control_colon
UC_active_colon
UC_inactive_colon
color
UC
cntrl

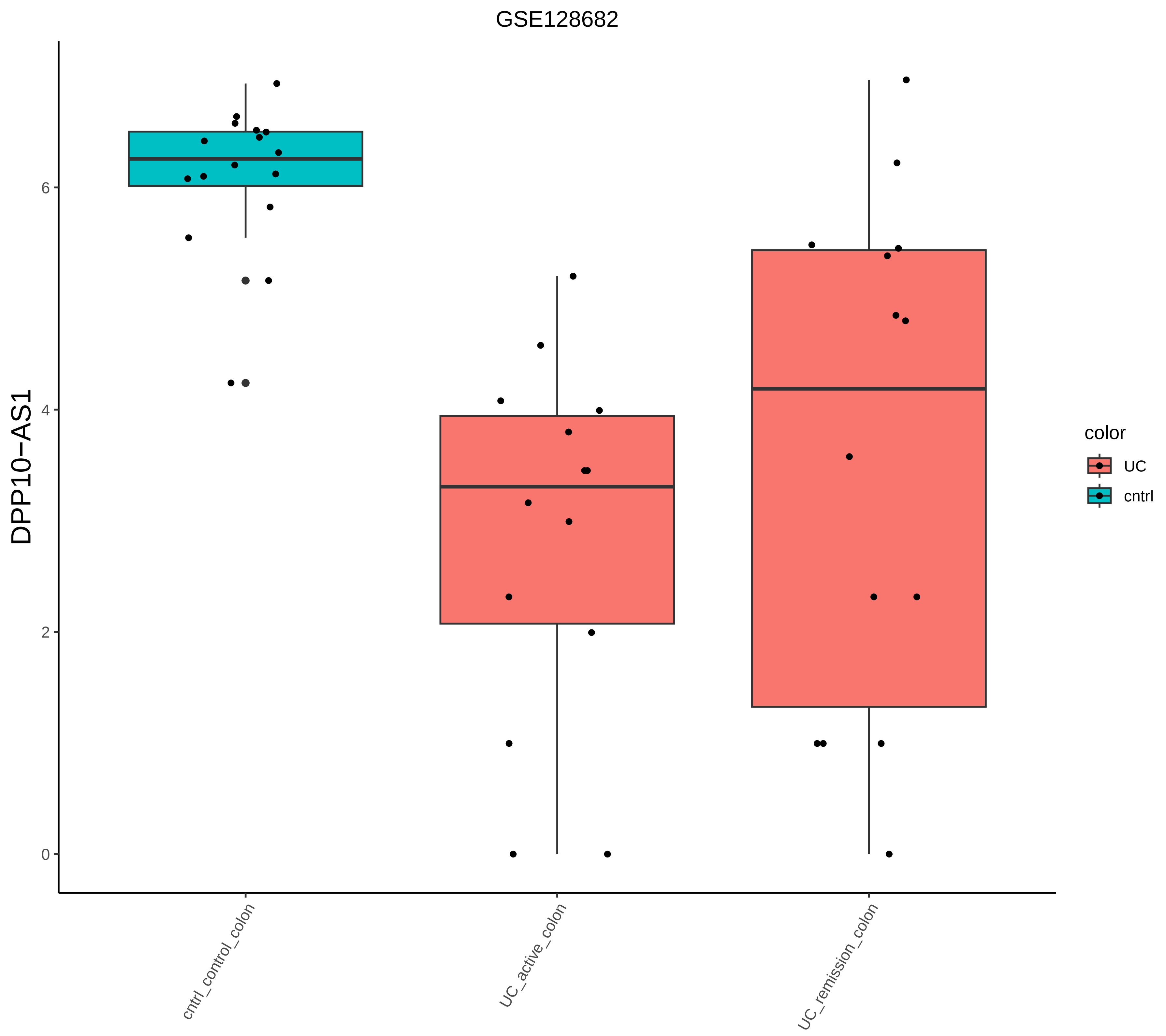

GSE128682
DPP10−AS1
0
2
4
6
cntrl_control_colon
UC_active_colon
UC_remission_colon
color
UC
cntrl

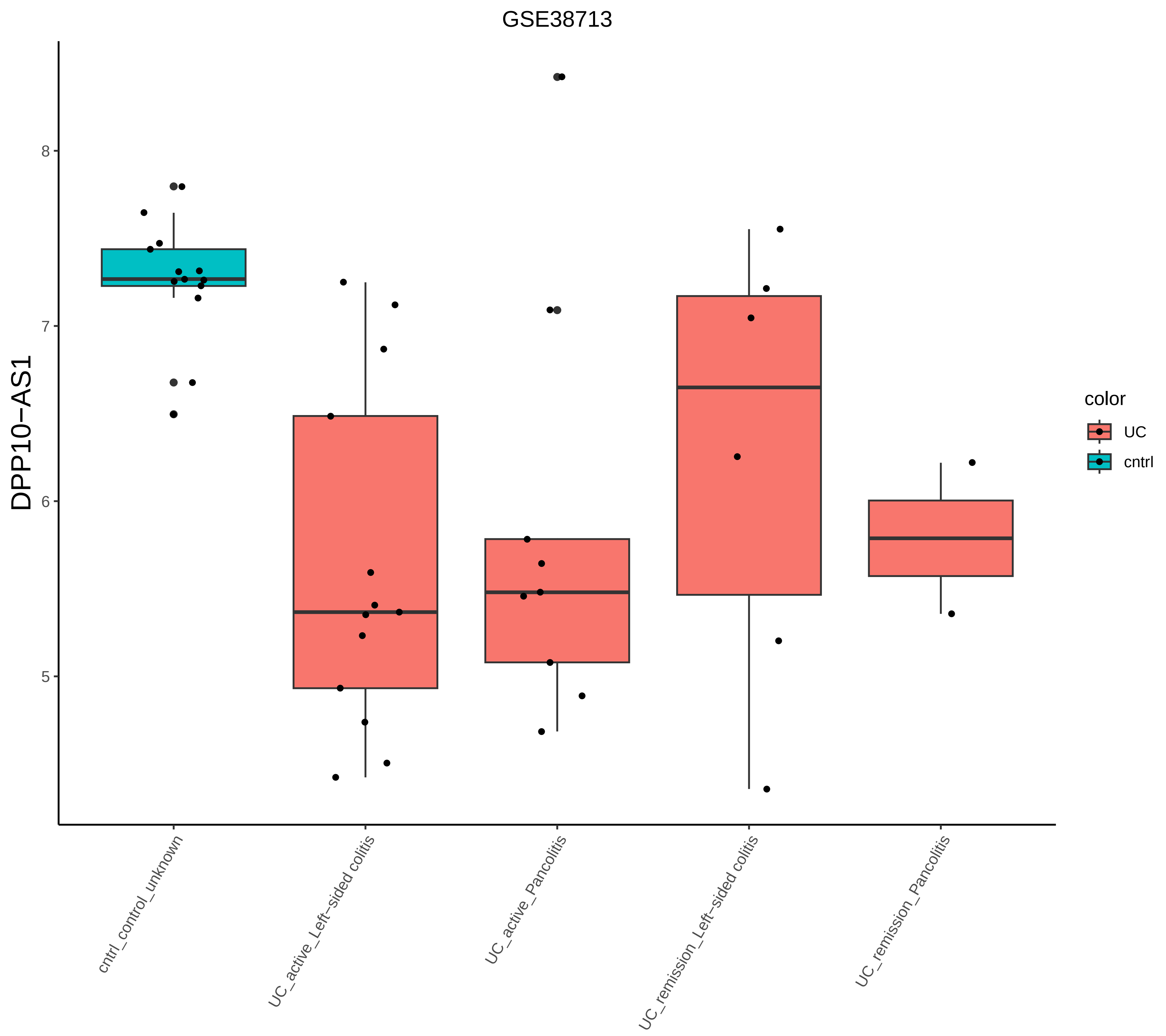

GSE38713
DPP10−AS1
8
7
6
5
cntrl_control_unknown
UC_active_Left−sided colitis
UC_active_Pancolitis
UC_remission_Left−sided colitis
UC_remission_Pancolitis
color
UC
cntrl

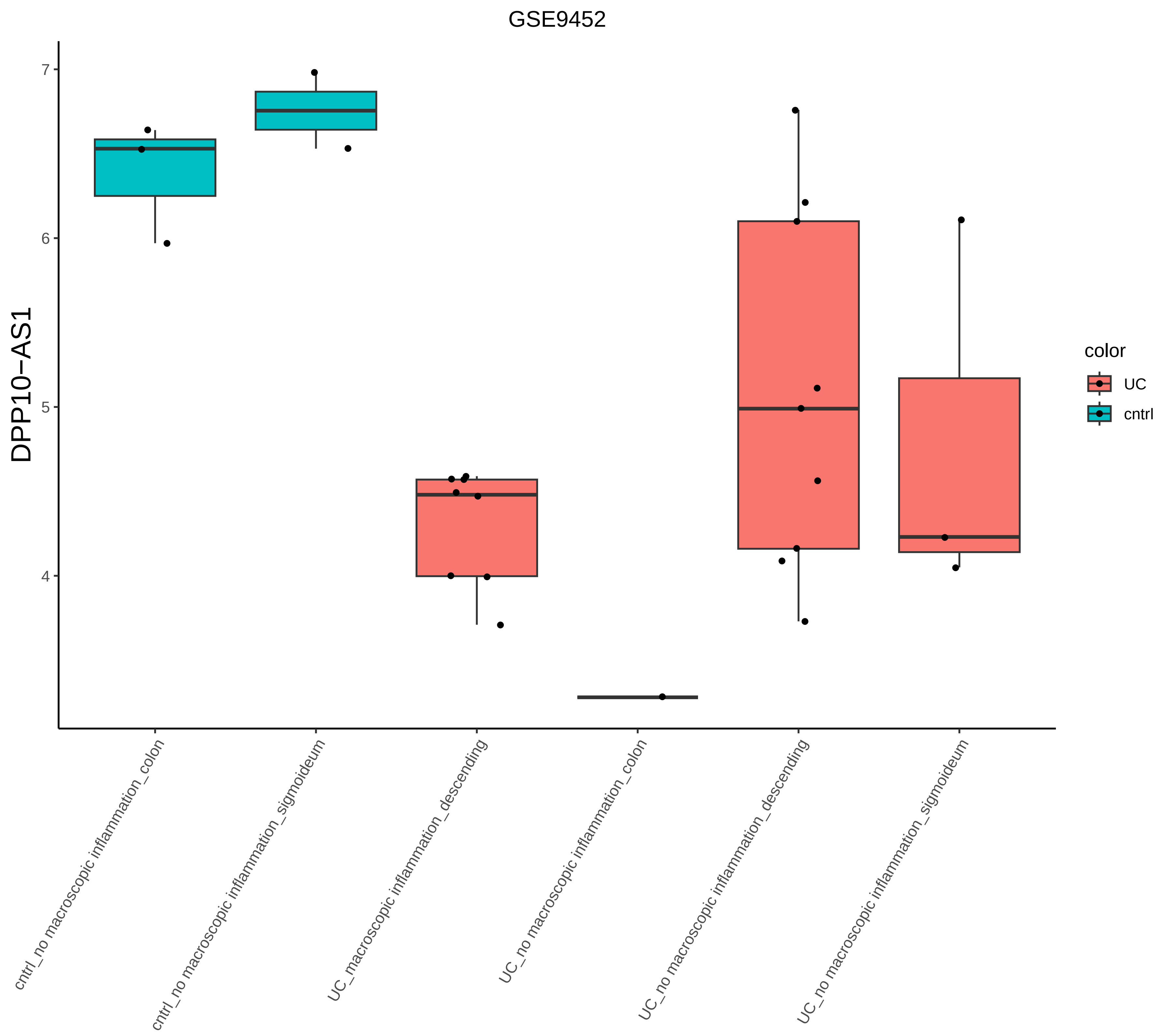
GSE9452
DPP10−AS1
7
6
5
4
color
UC
cntrl
cntrl_no macroscopic inflammation_colon
cntrl_no macroscopic inflammation_sigmoideum
UC_macroscopic inflammation_descending
UC_no macroscopic inflammation_colon
UC_no macroscopic inflammation_descending
UC_no macroscopic inflammation_sigmoideum

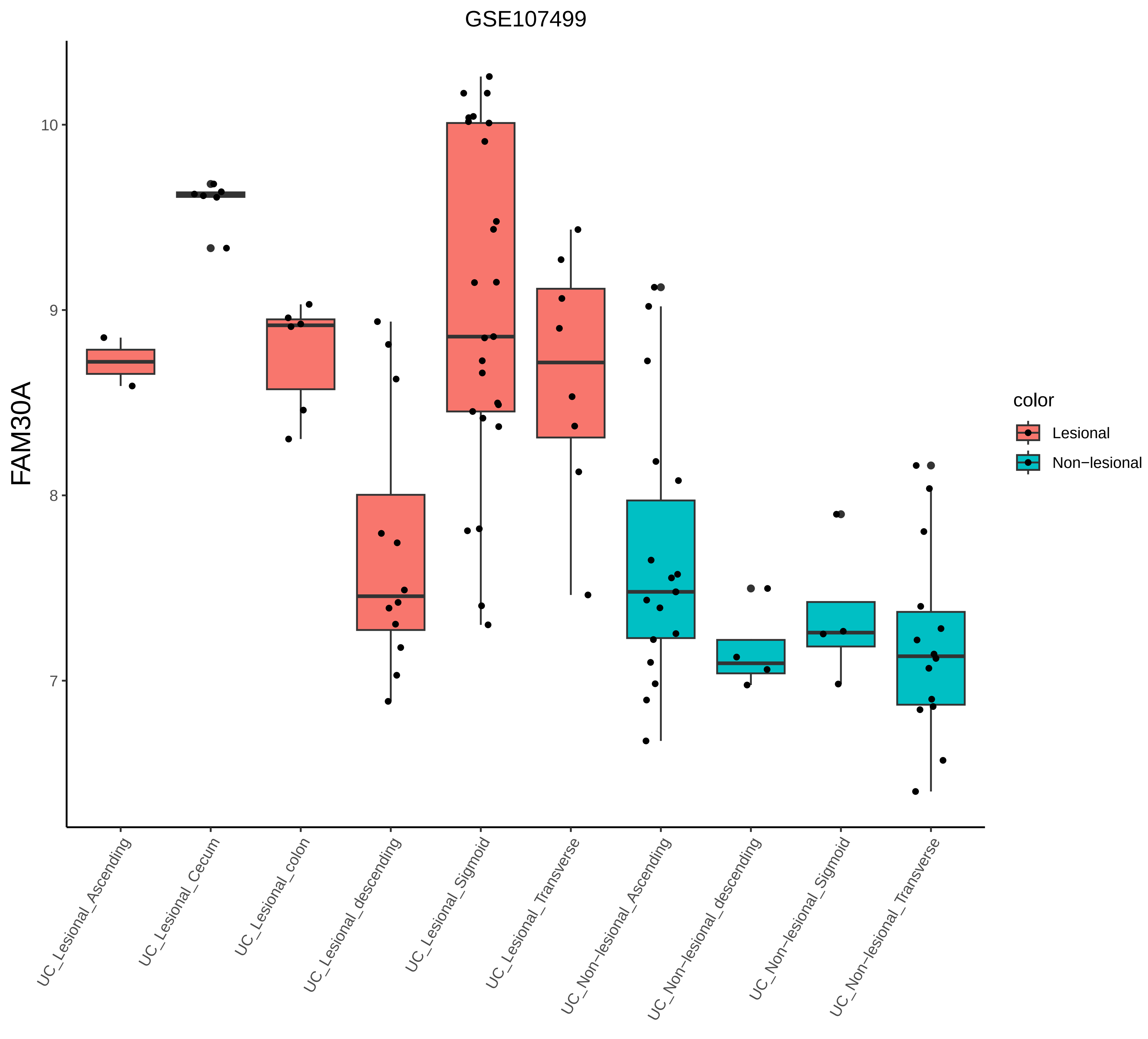

GSE107499
FAM30A
10
9
8
7
color
Lesional
Non−lesional
UC_Lesional_Ascending
UC_Lesional_Cecum
UC_Lesional_colon
UC_Lesional_descending
UC_Lesional_Sigmoid
UC_Lesional_Transverse
UC_Non−lesional_Ascending
UC_Non−lesional_descending
UC_Non−lesional_Sigmoid
UC_Non−lesional_Transverse

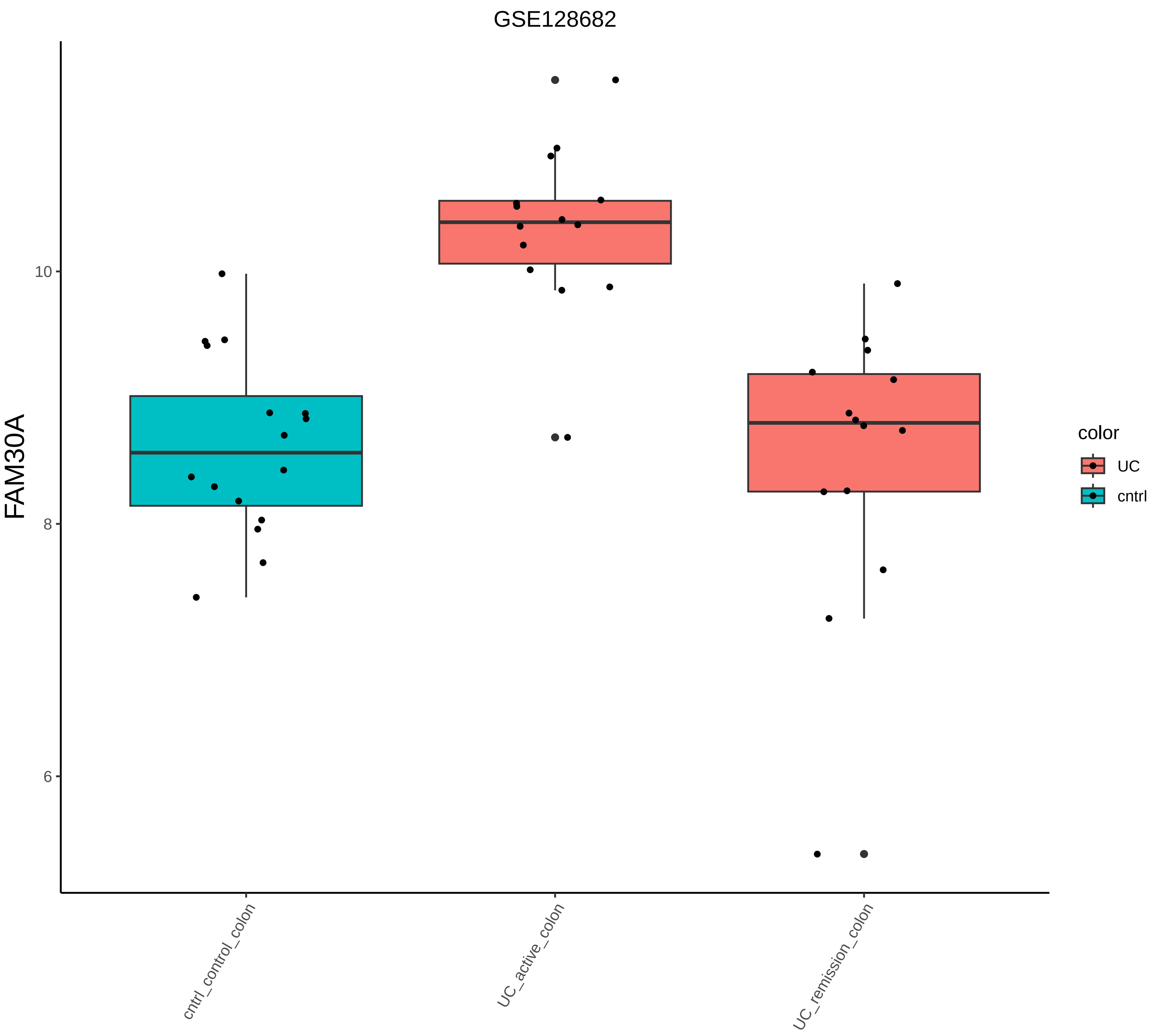

GSE128682
FAM30A
10
8
6
cntrl_control_colon
UC_active_colon
UC_remission_colon
color
UC
cntrl

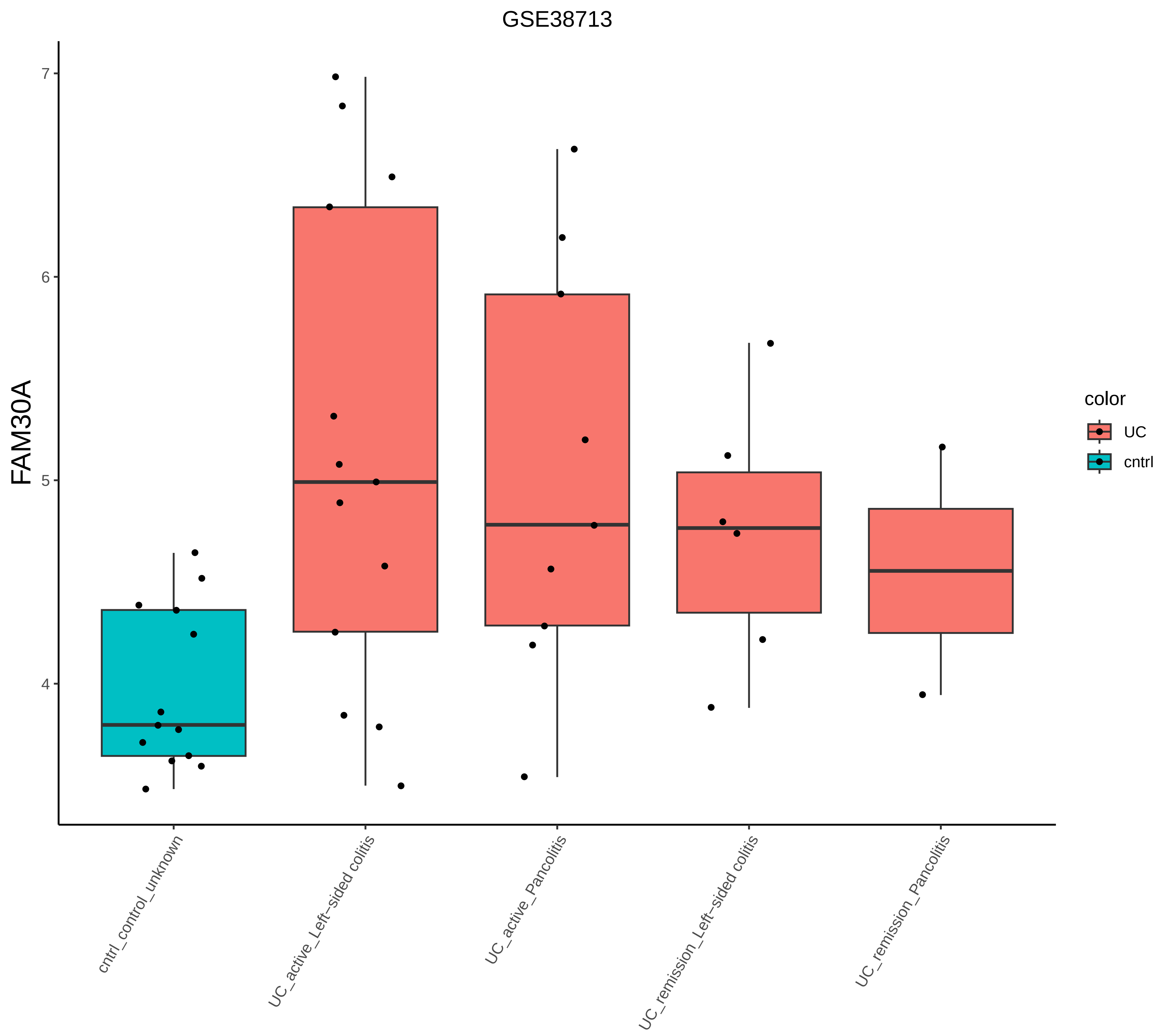
GSE38713
FAM30A
7
6
5
4
cntrl_control_unknown
UC_active_Left−sided colitis
UC_active_Pancolitis
UC_remission_Left−sided colitis
UC_remission_Pancolitis
color
UC
cntrl

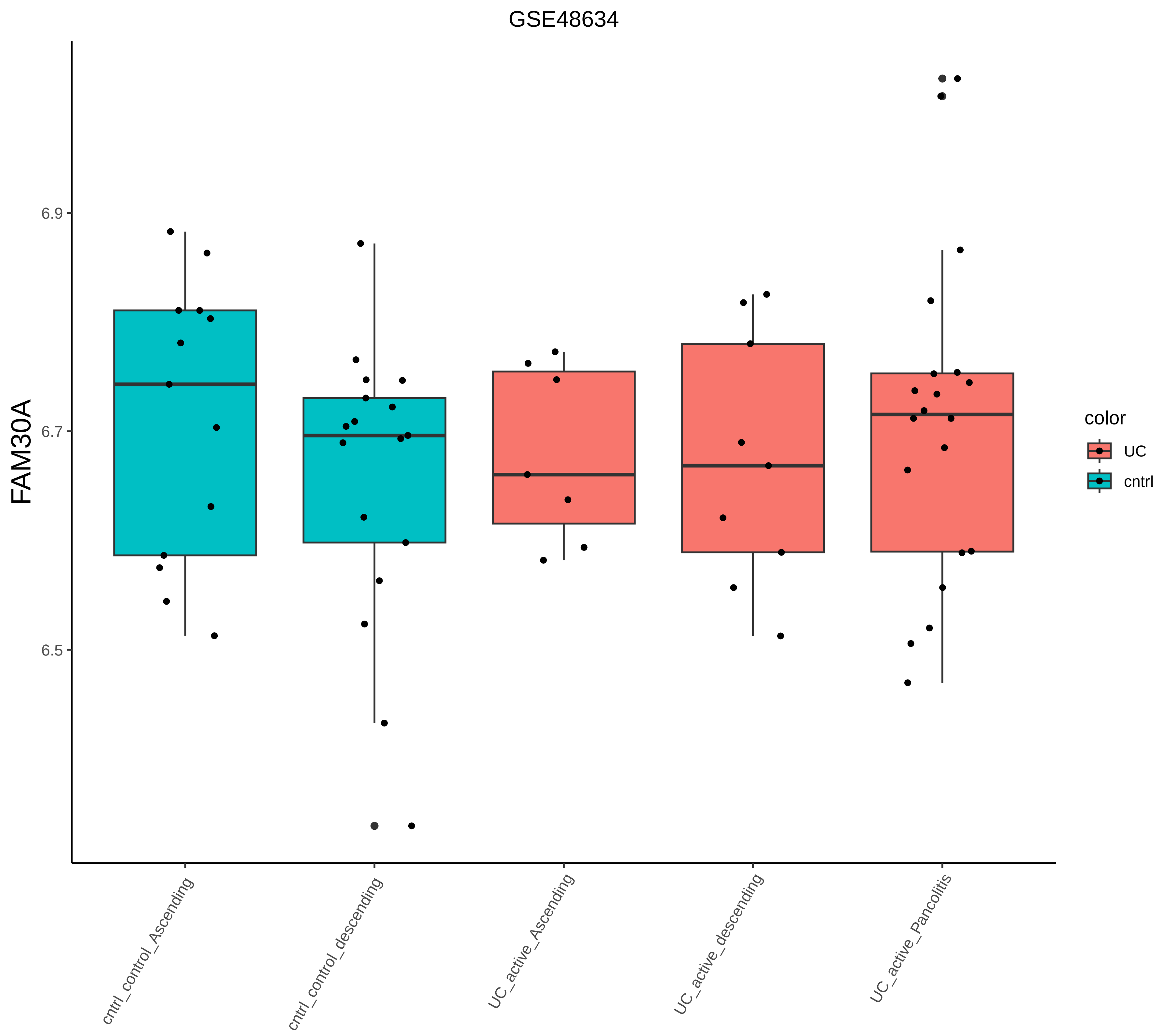

GSE48634
FAM30A
6.9
6.7
6.5
color
UC
cntrl
cntrl_control_Ascending
cntrl_control_descending
UC_active_Ascending
UC_active_descending
UC_active_Pancolitis

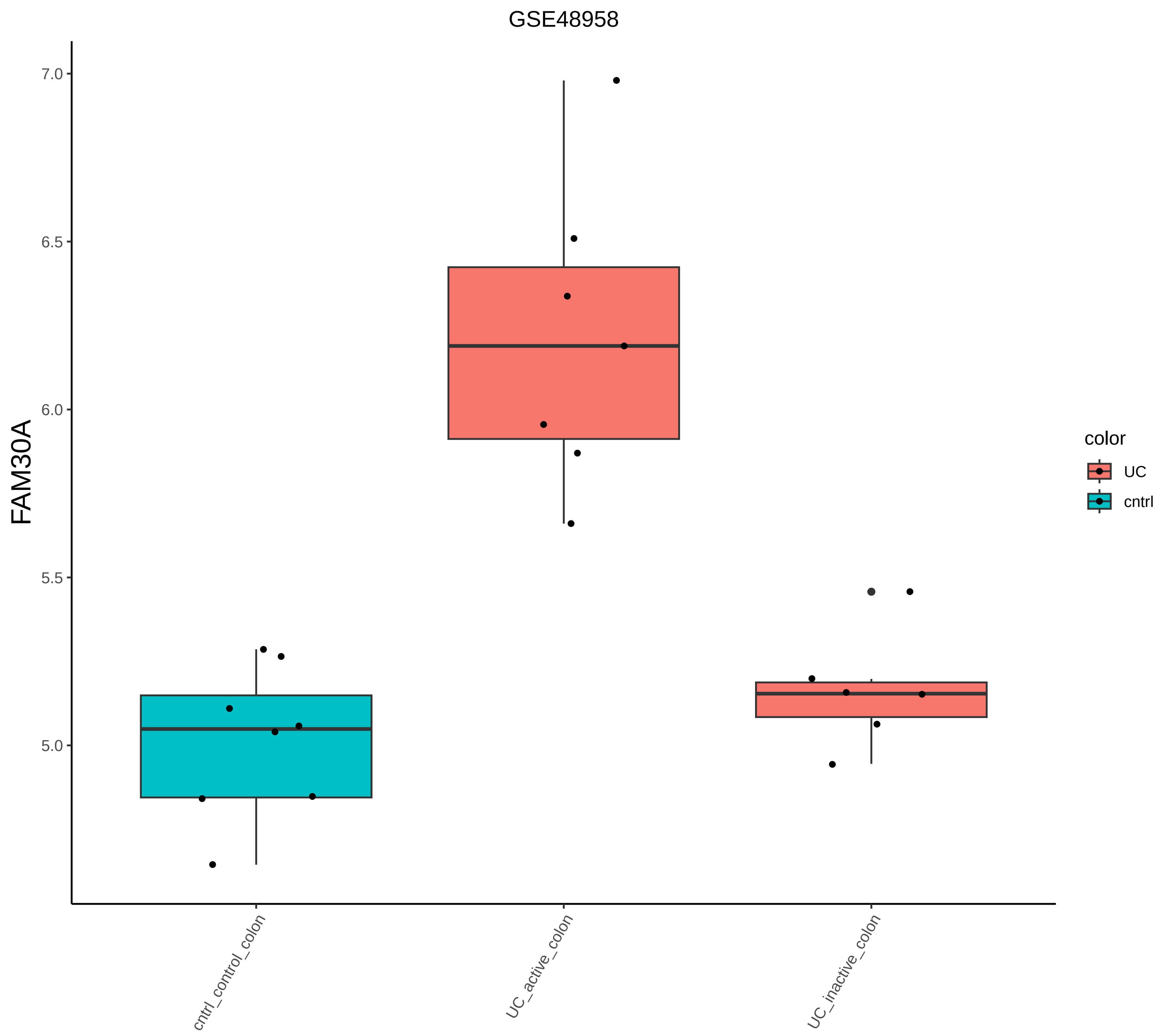

GSE48958
FAM30A
7.0
6.5
6.0
5.5
5.0
cntrl_control_colon
UC_active_colon
UC_inactive_colon
color
UC
cntrl

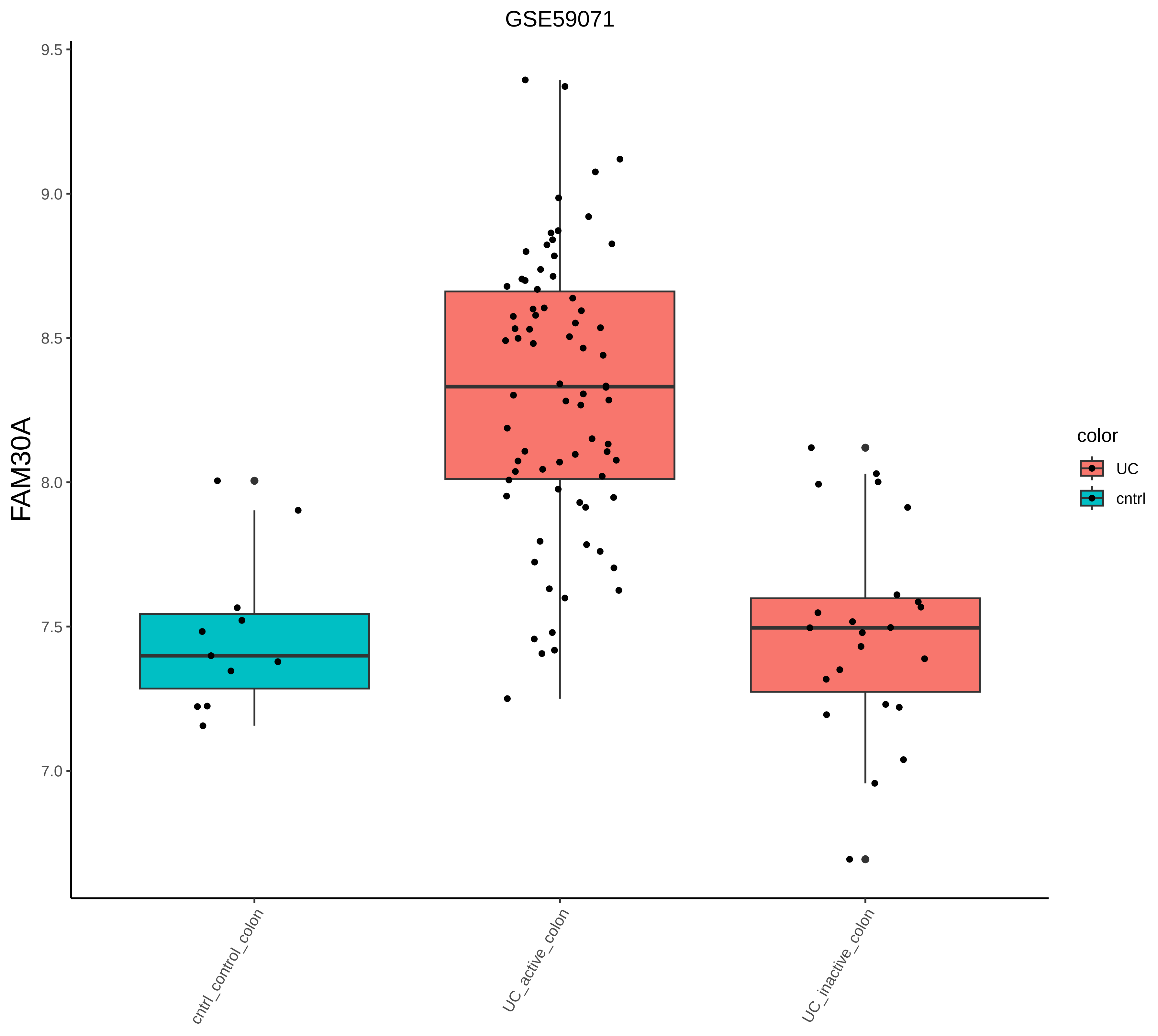
GSE59071
FAM30A
9.5
9.0
8.5
8.0
7.5
7.0
cntrl_control_colon
UC_active_colon
UC_inactive_colon
color
UC
cntrl

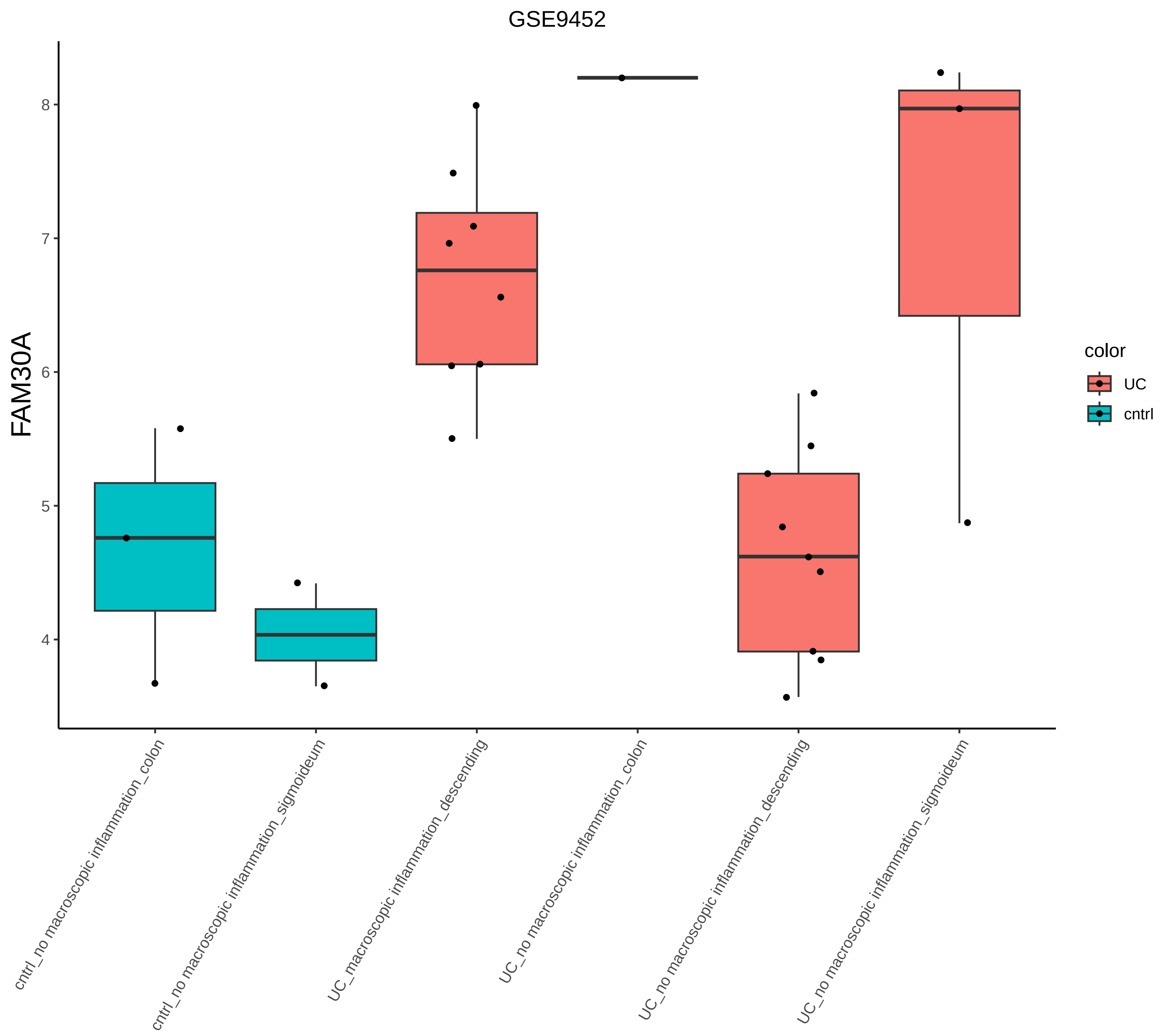

GSE9452
FAM30A
8
7
6
5
4
color
UC
cntrl
cntrl_no macroscopic inflammation_colon
cntrl_no macroscopic inflammation_sigmoideum
UC_macroscopic inflammation_descending
UC_no macroscopic inflammation_colon
UC_no macroscopic inflammation_descending
UC_no macroscopic inflammation_sigmoideum

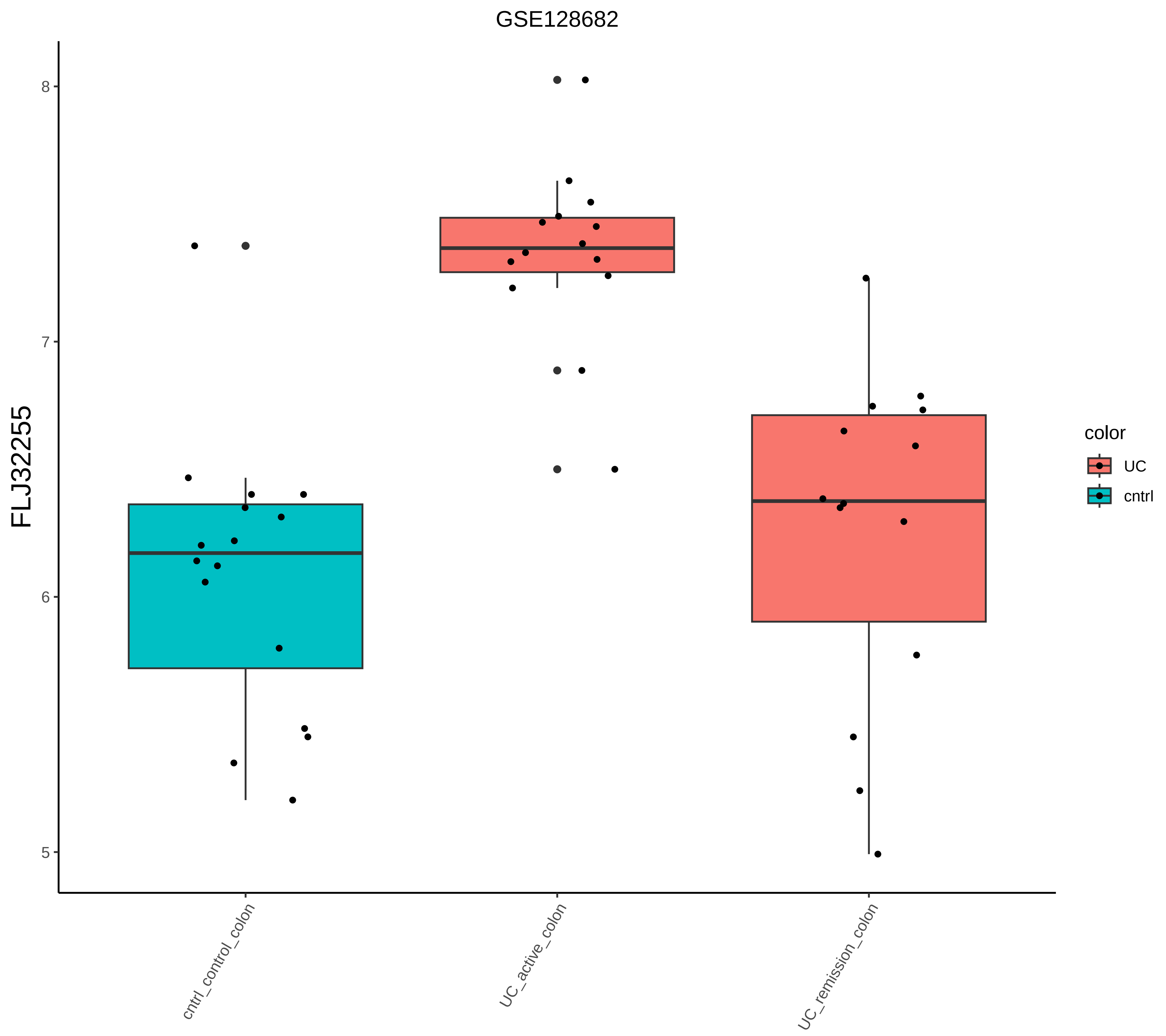

GSE128682
FLJ32255
8
7
6
5
cntrl_control_colon
UC_active_colon
UC_remission_colon
color
UC
cntrl

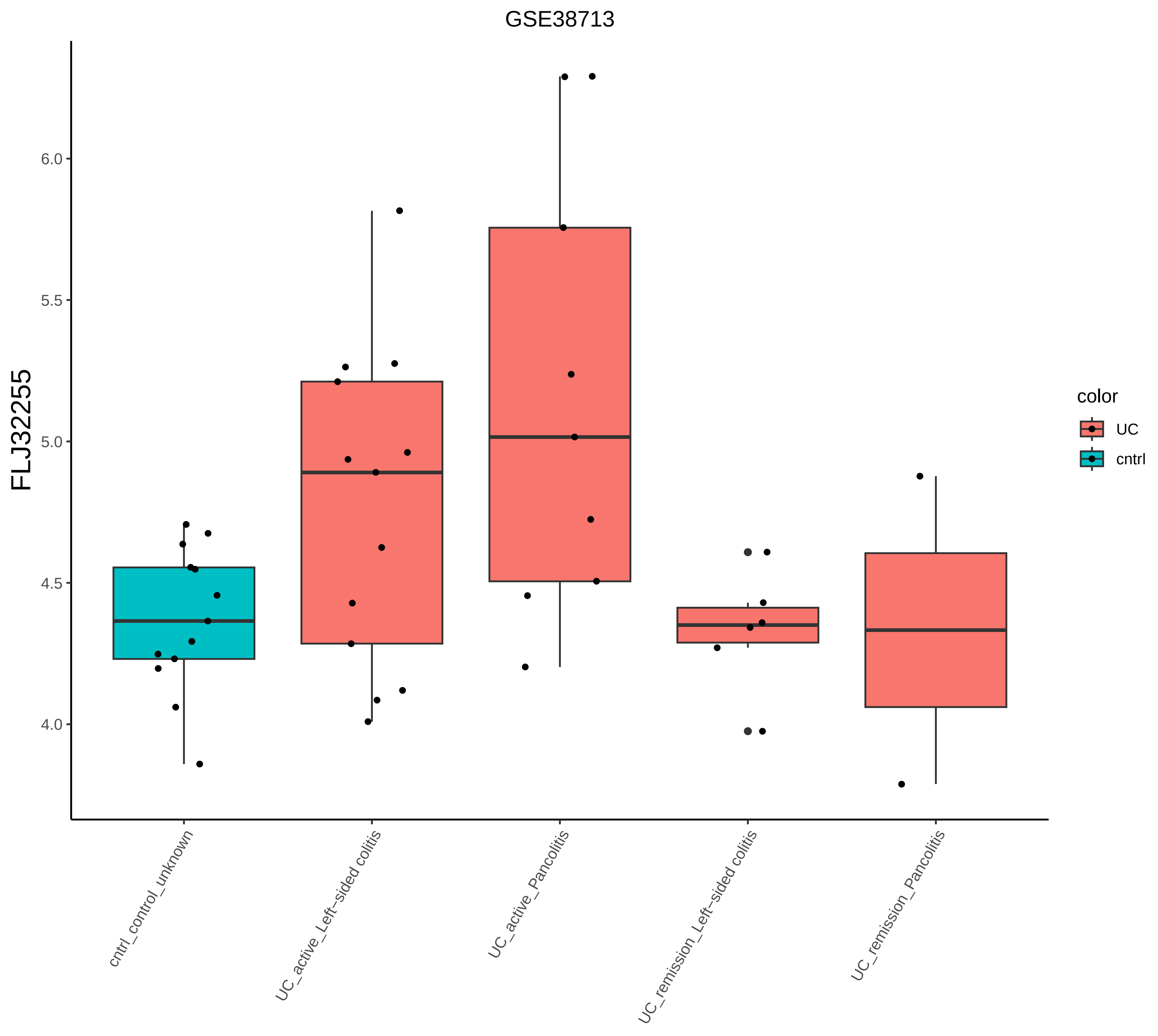

GSE38713
FLJ32255
6.0
5.5
5.0
4.5
4.0
cntrl_control_unknown
UC_active_Left−sided colitis
UC_active_Pancolitis
UC_remission_Left−sided colitis
UC_remission_Pancolitis
color
UC
cntrl

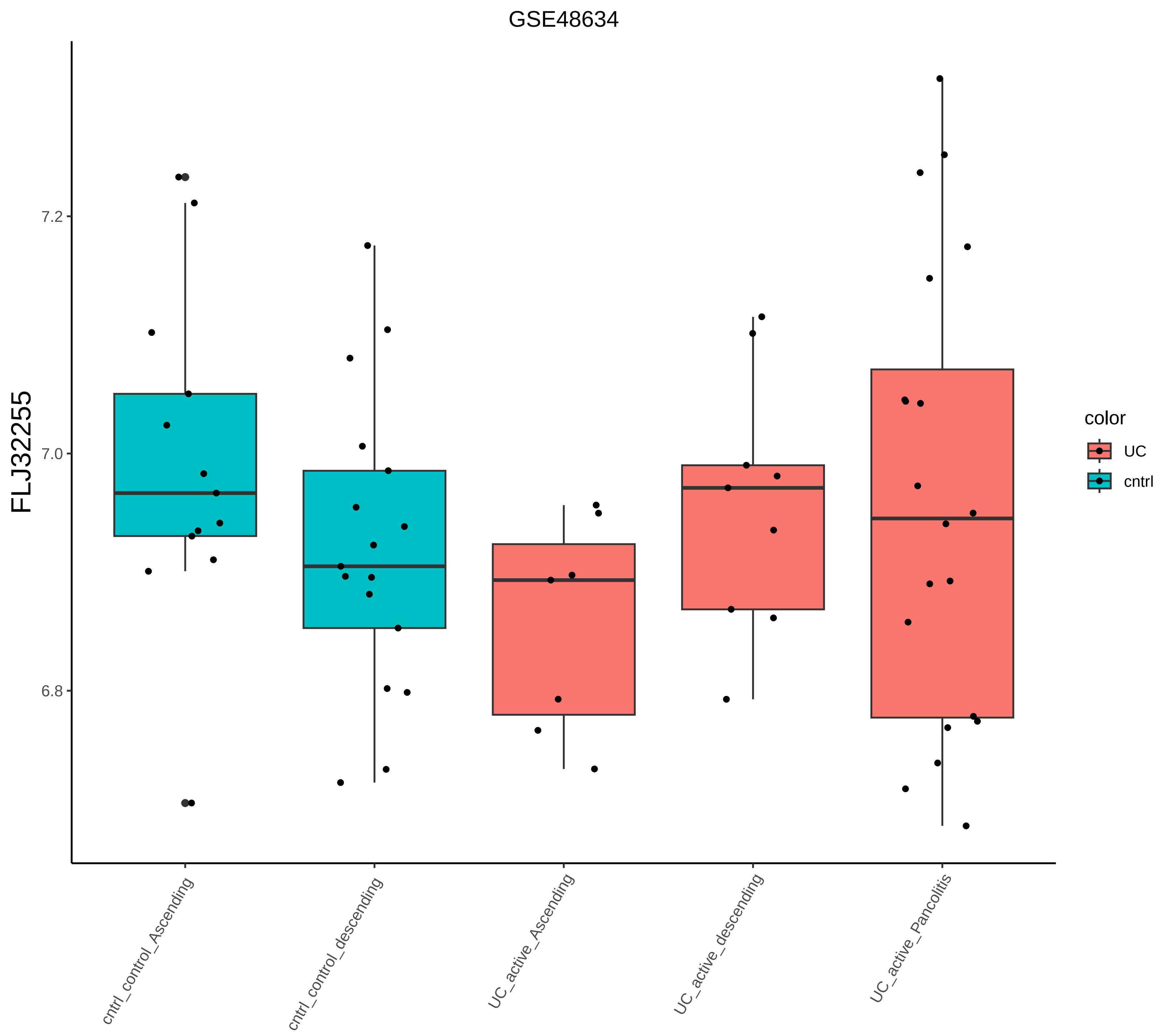

GSE48634
FLJ32255
7.2
7.0
6.8
cntrl_control_Ascending
cntrl_control_descending
UC_active_Ascending
UC_active_descending
UC_active_Pancolitis
color
UC
cntrl

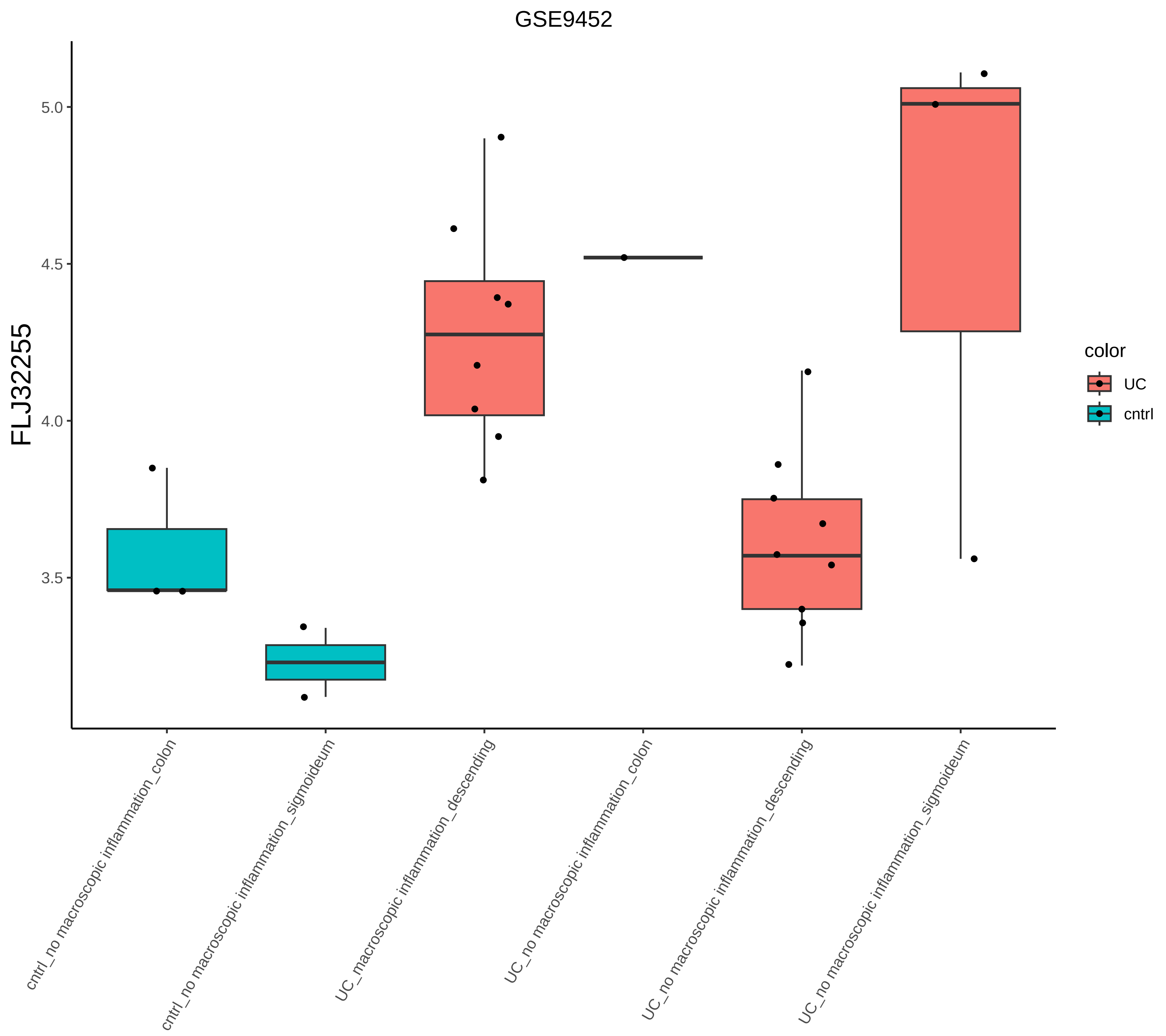

GSE9452
FLJ32255
5.0
4.5
4.0
3.5
color
UC
cntrl
cntrl_no macroscopic inflammation_colon
cntrl_no macroscopic inflammation_sigmoideum
UC_macroscopic inflammation_descending
UC_no macroscopic inflammation_colon
UC_no macroscopic inflammation_descending
UC_no macroscopic inflammation_sigmoideum

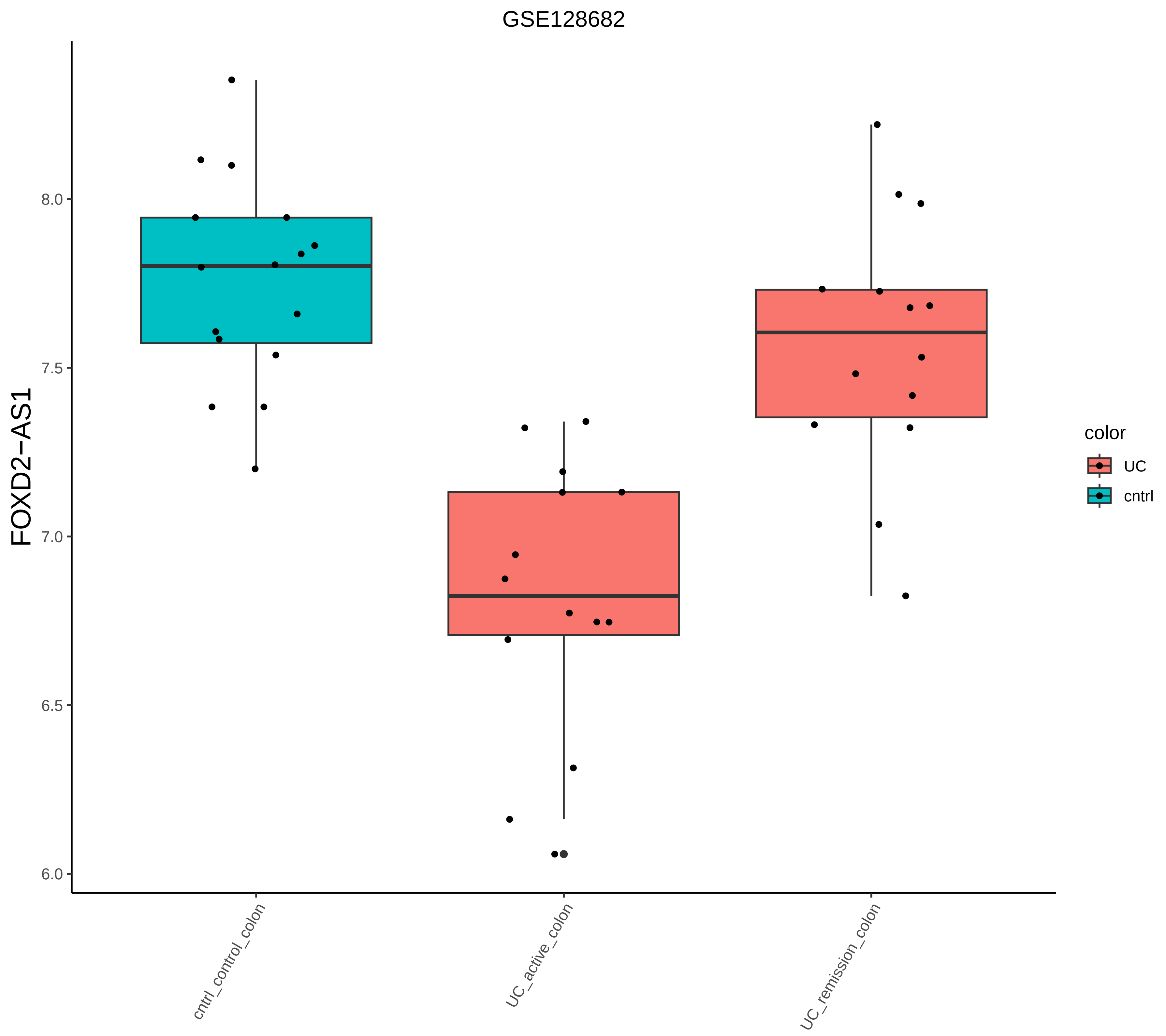
GSE128682
FOXD2−AS1
8.0
7.5
7.0
6.5
6.0
cntrl_control_colon
UC_active_colon
UC_remission_colon
color
UC
cntrl

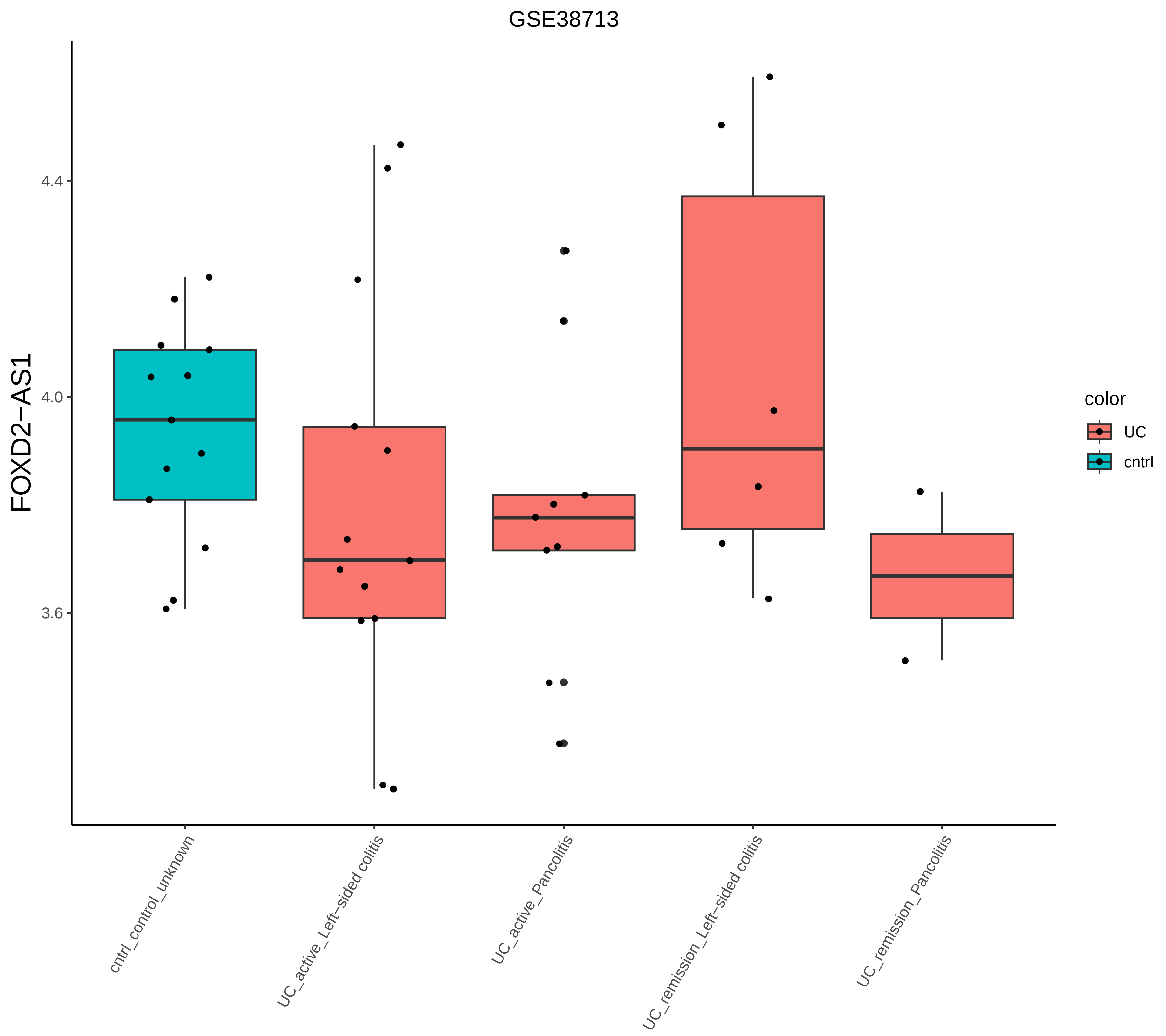

GSE38713
FOXD2−AS1
4.4
4.0
3.6
cntrl_control_unknown
UC_active_Left−sided colitis
UC_active_Pancolitis
UC_remission_Left−sided colitis
UC_remission_Pancolitis
color
UC
cntrl

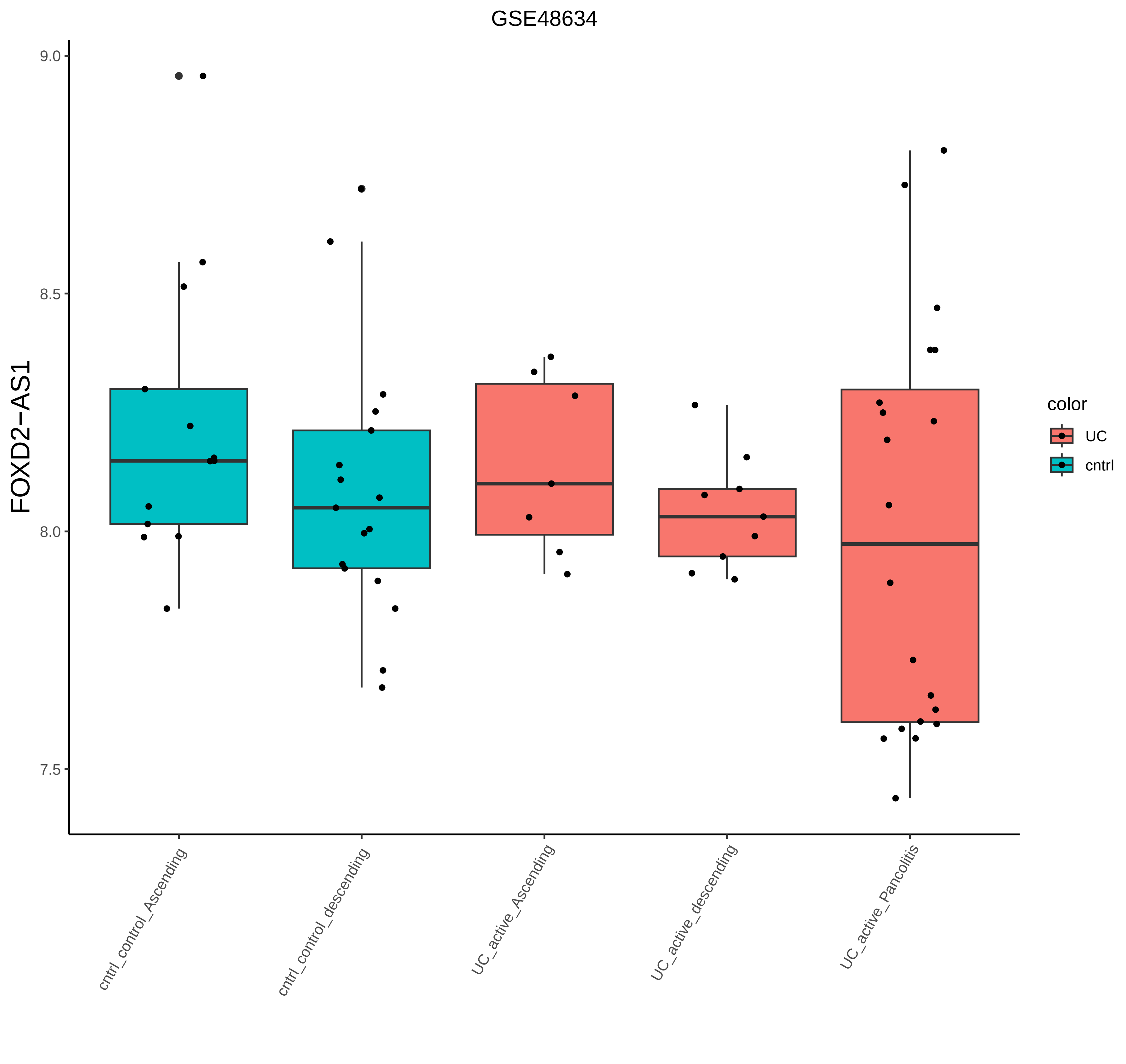

GSE48634
FOXD2−AS1
9.0
8.5
8.0
7.5
cntrl_control_Ascending
cntrl_control_descending
UC_active_Ascending
UC_active_descending
UC_active_Pancolitis
color
UC
cntrl

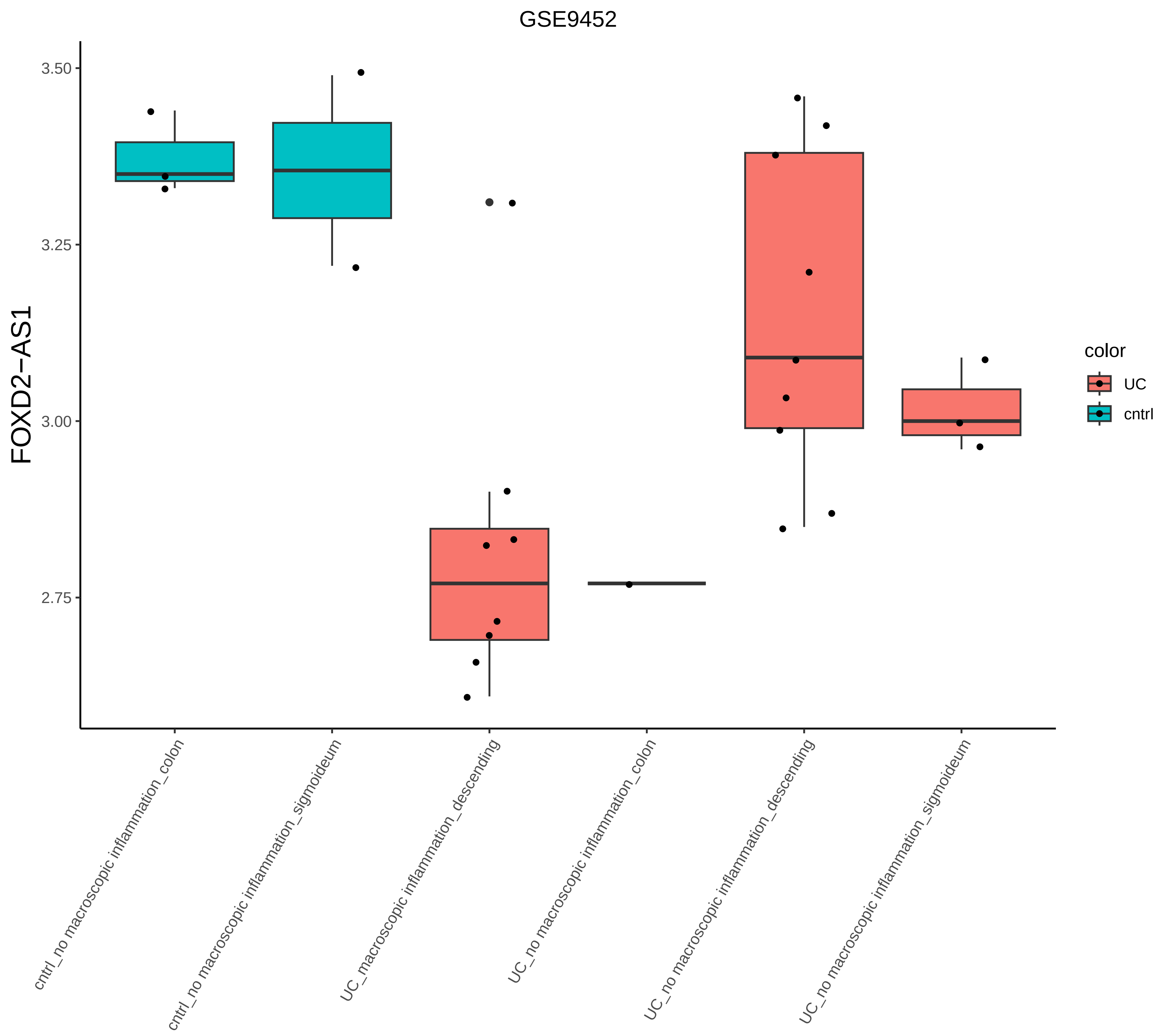

GSE9452
FOXD2−AS1
3.50
3.25
3.00
2.75
color
UC
cntrl
cntrl_no macroscopic inflammation_colon
cntrl_no macroscopic inflammation_sigmoideum
UC_macroscopic inflammation_descending
UC_no macroscopic inflammation_colon
UC_no macroscopic inflammation_descending
UC_no macroscopic inflammation_sigmoideum

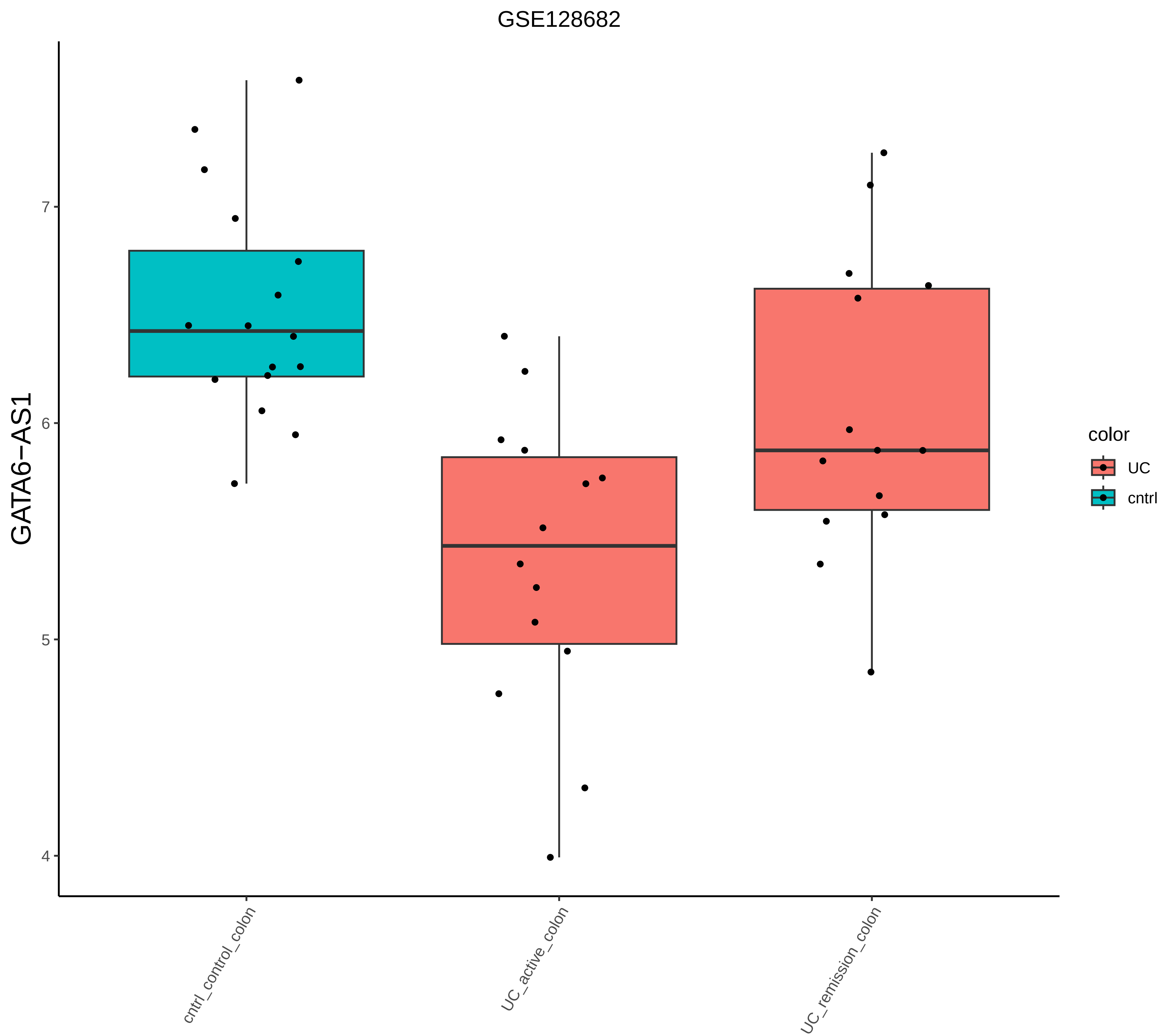
GSE128682
GATA6−AS1
7
6
5
4
cntrl_control_colon
UC_active_colon
UC_remission_colon
color
UC
cntrl

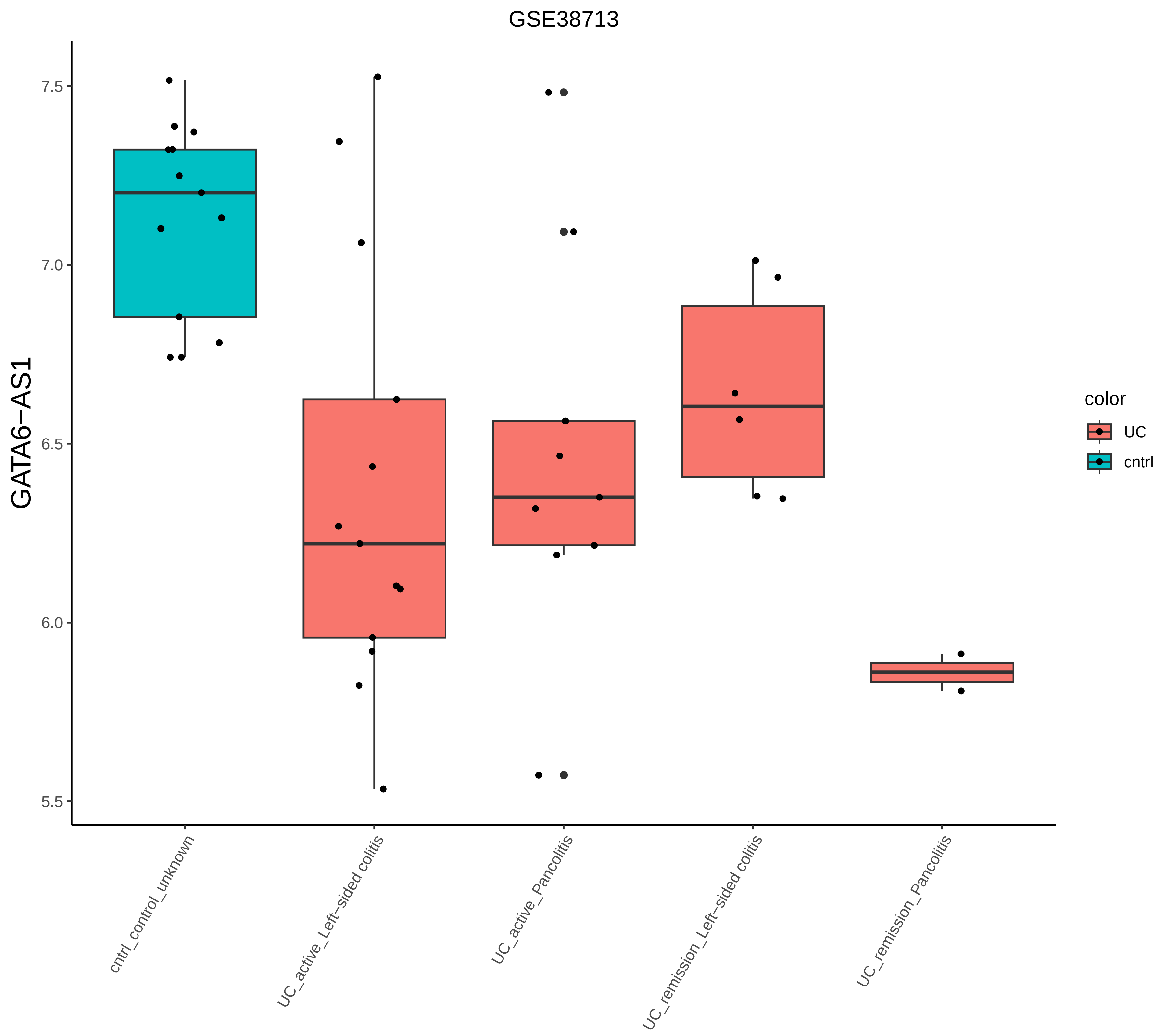

GSE38713
GATA6−AS1
7.5
7.0
6.5
6.0
5.5
cntrl_control_unknown
UC_active_Left−sided colitis
UC_active_Pancolitis
UC_remission_Left−sided colitis
UC_remission_Pancolitis
color
UC
cntrl

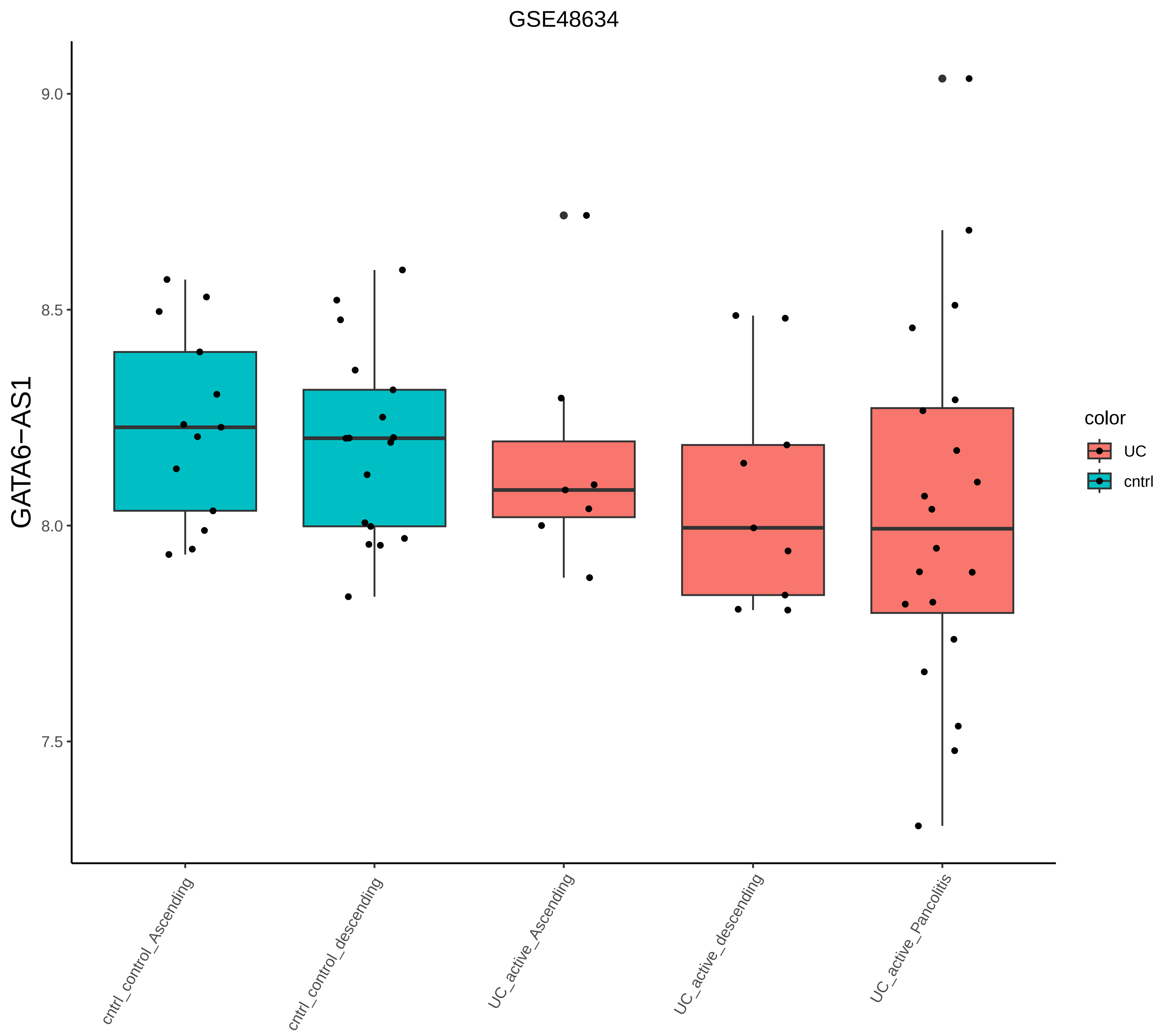

GSE48634
GATA6−AS1
9.0
8.5
8.0
7.5
cntrl_control_Ascending
cntrl_control_descending
UC_active_Ascending
UC_active_descending
UC_active_Pancolitis
color
UC
cntrl

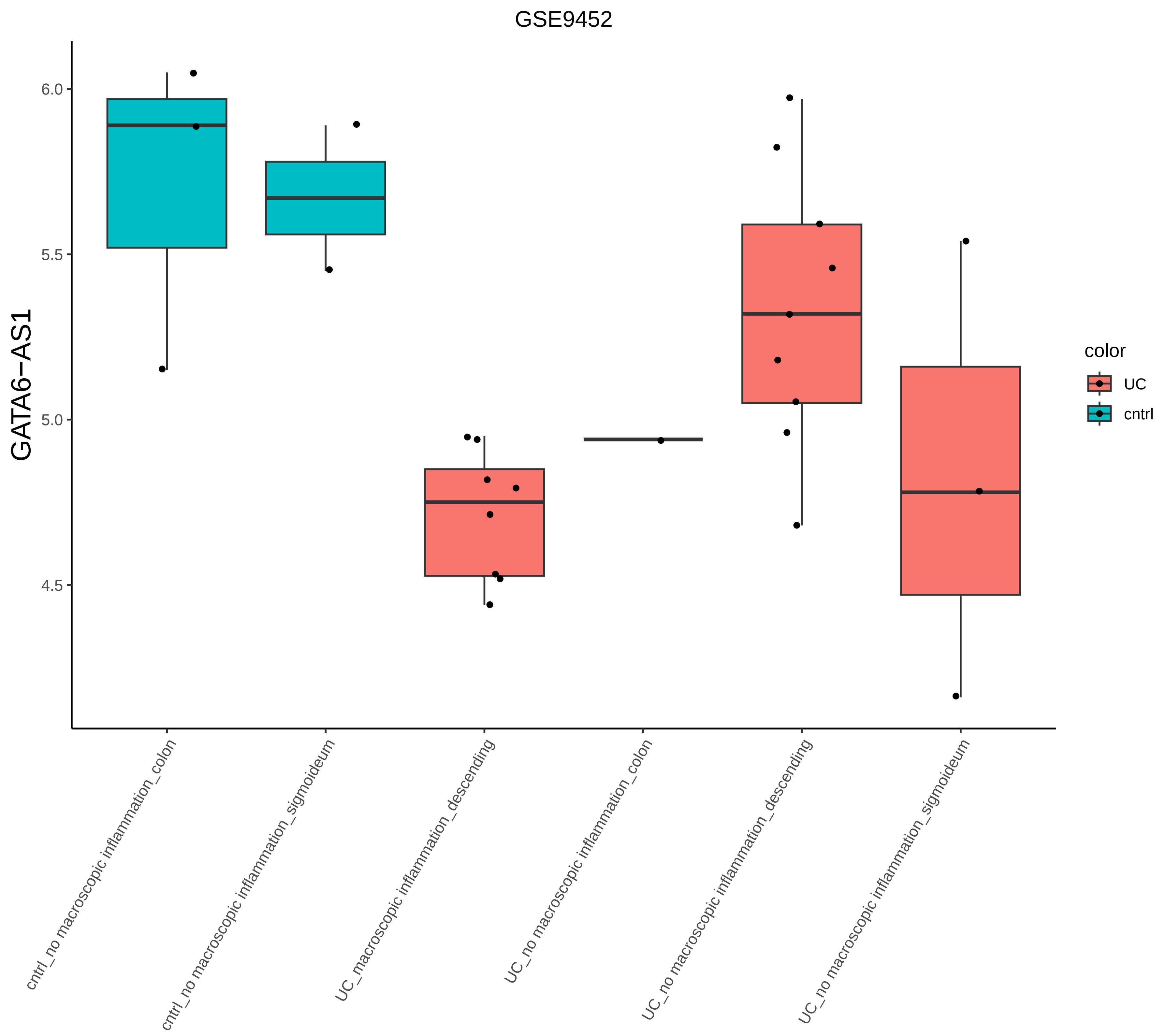

GSE9452
GATA6−AS1
6.0
5.5
5.0
4.5
color
UC
cntrl
cntrl_no macroscopic inflammation_colon
cntrl_no macroscopic inflammation_sigmoideum
UC_macroscopic inflammation_descending
UC_no macroscopic inflammation_colon
UC_no macroscopic inflammation_descending
UC_no macroscopic inflammation_sigmoideum

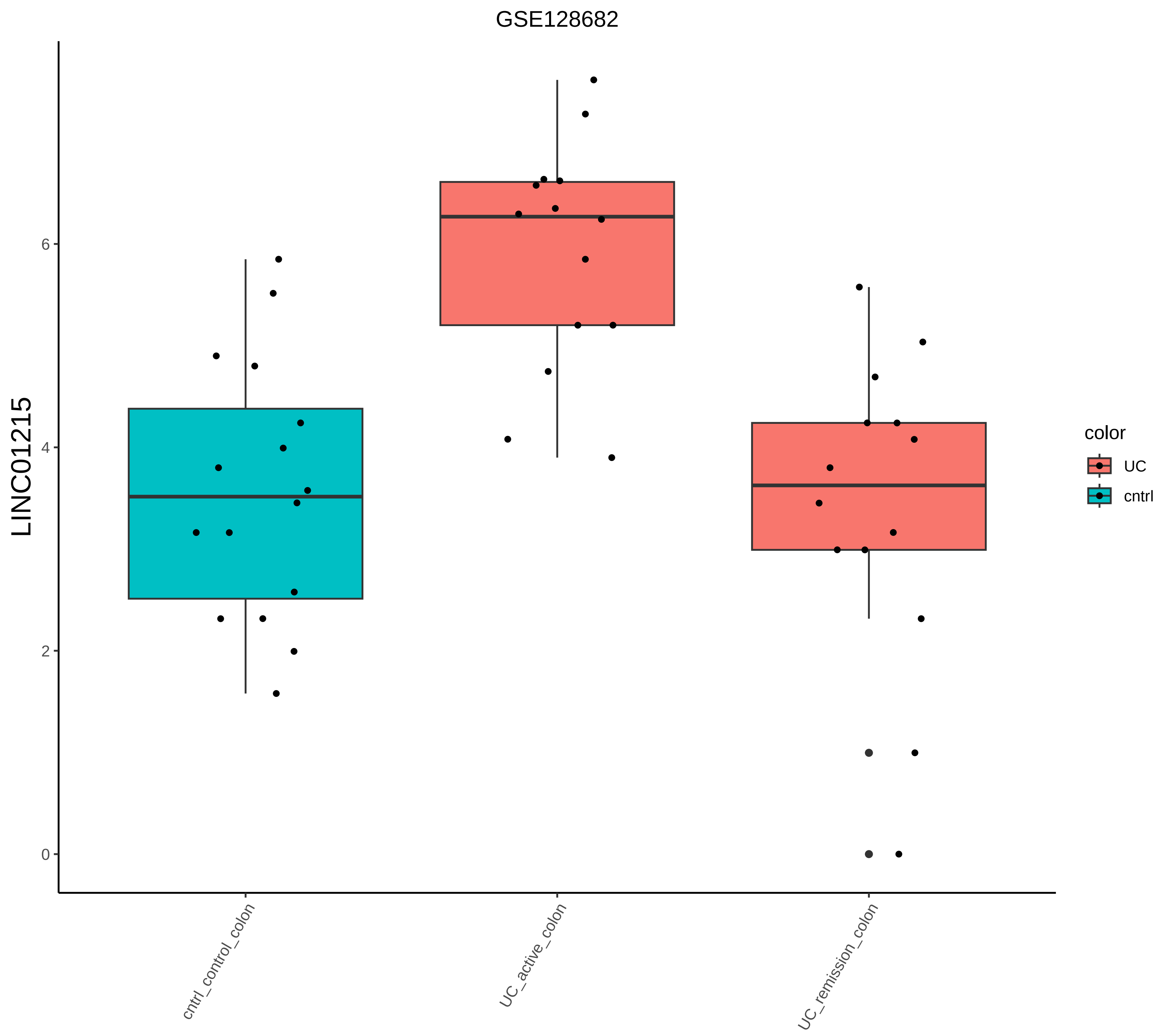

GSE128682
LINC01215
0
2
4
6
cntrl_control_colon
UC_active_colon
UC_remission_colon
color
UC
cntrl

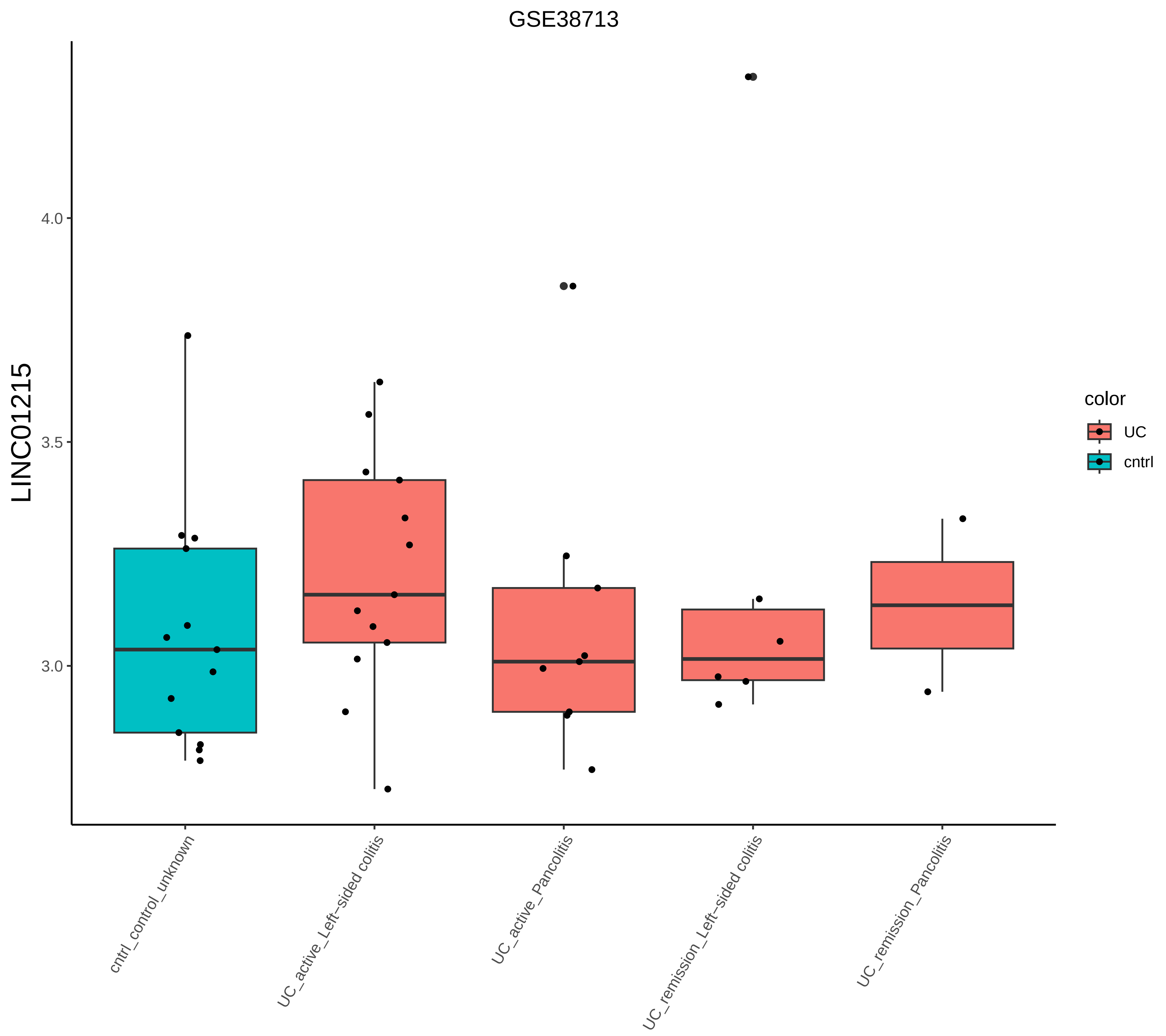
GSE38713
LINC01215
4.0
3.5
3.0
color
UC
cntrl
cntrl_control_unknown
UC_active_Left−sided colitis
UC_active_Pancolitis
UC_remission_Left−sided colitis
UC_remission_Pancolitis

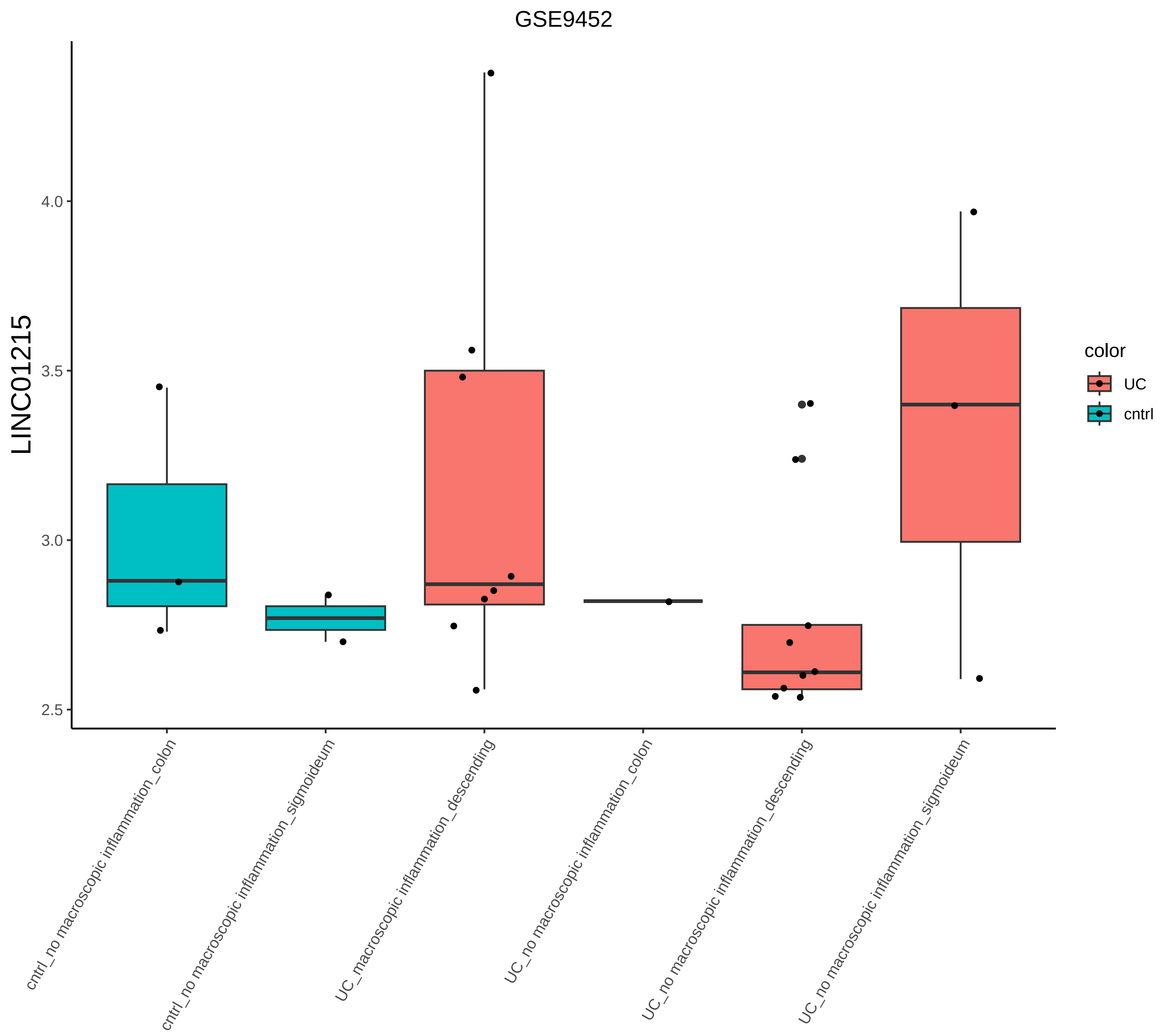

GSE9452
LINC01215
4.0
3.5
3.0
2.5
cntrl_no macroscopic inflammation_colon
cntrl_no macroscopic inflammation_sigmoideum
UC_macroscopic inflammation_descending
UC_no macroscopic inflammation_colon
UC_no macroscopic inflammation_descending
UC_no macroscopic inflammation_sigmoideum
color
UC
cntrl

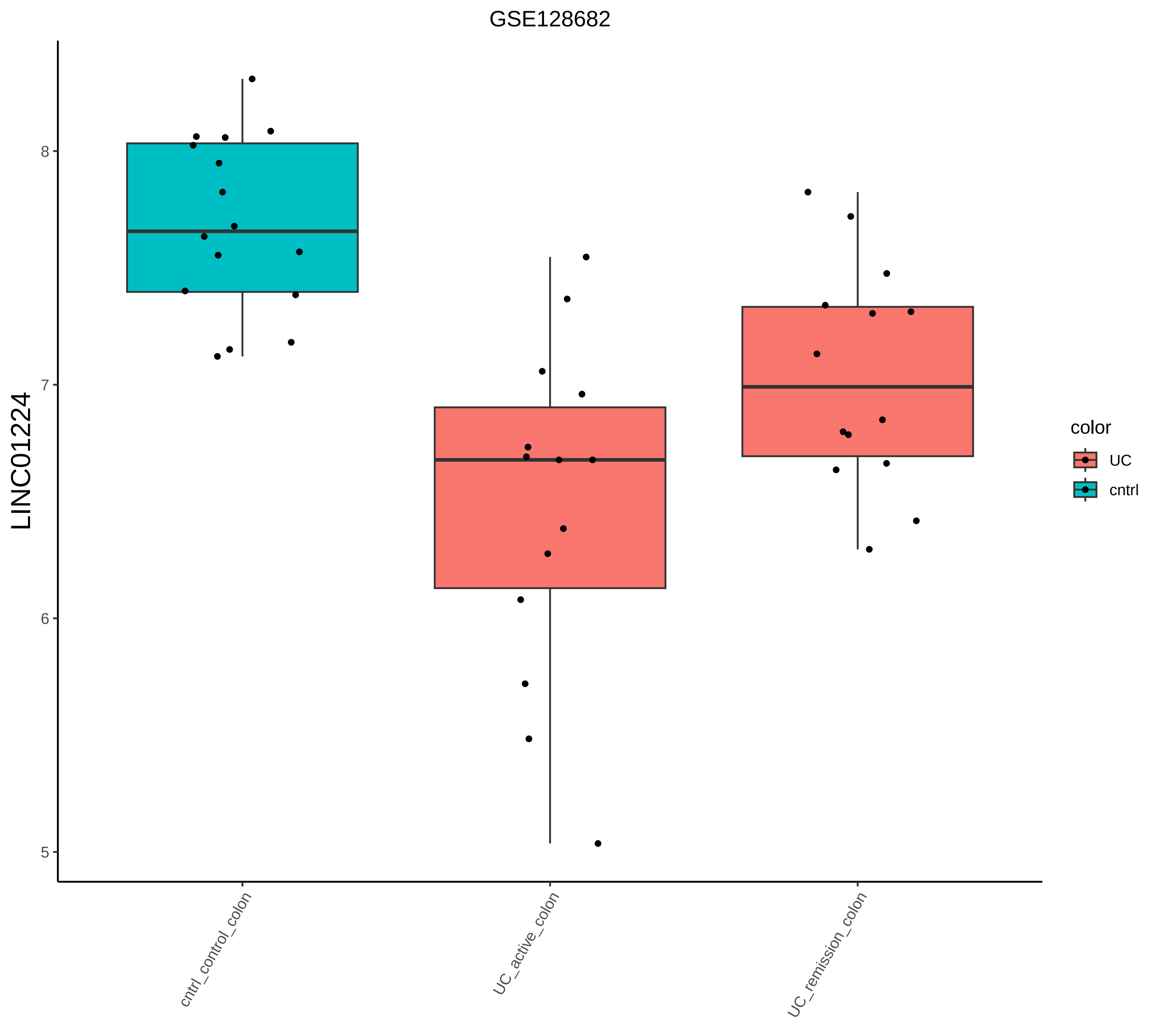

GSE128682
LINC01224
8
7
6
5
cntrl_control_colon
UC_active_colon
UC_remission_colon
color
UC
cntrl

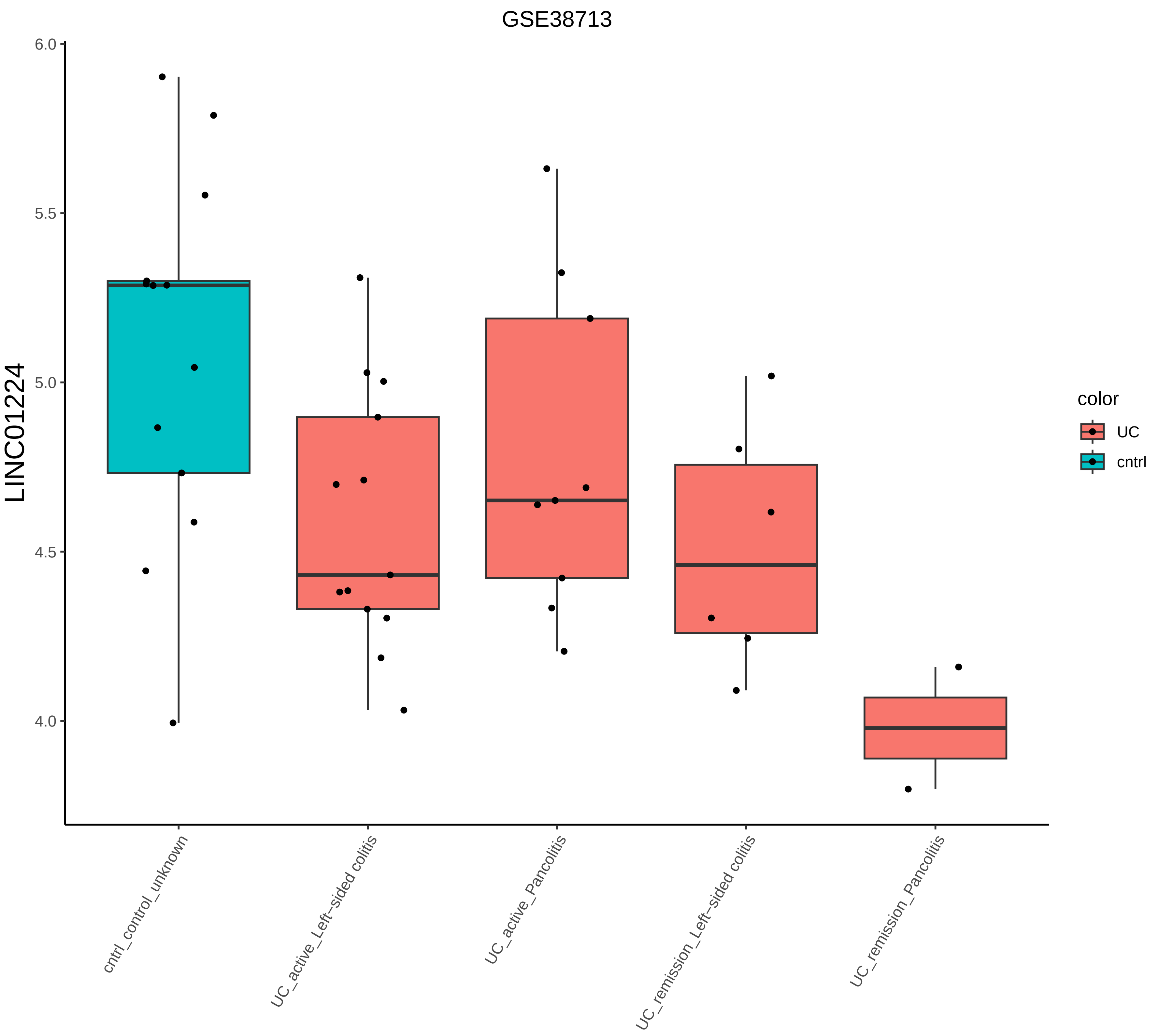
GSE38713
LINC01224
6.0
5.5
5.0
4.5
4.0
color
UC
cntrl
cntrl_control_unknown
UC_active_Left−sided colitis
UC_active_Pancolitis
UC_remission_Left−sided colitis
UC_remission_Pancolitis

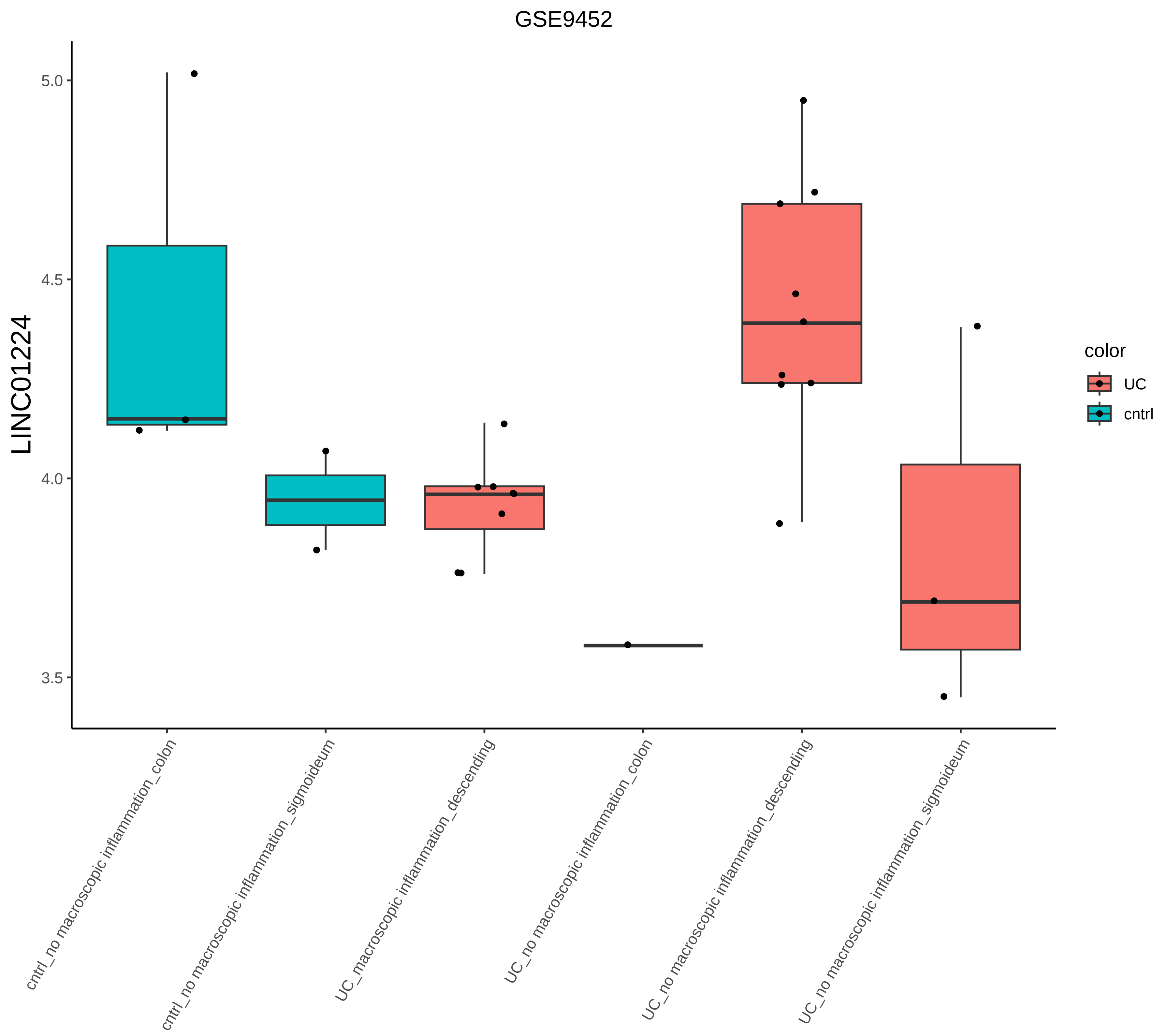
GSE9452
LINC01224
5.0
4.5
4.0
3.5
color
UC
cntrl
cntrl_no macroscopic inflammation_colon
cntrl_no macroscopic inflammation_sigmoideum
UC_macroscopic inflammation_descending
UC_no macroscopic inflammation_colon
UC_no macroscopic inflammation_descending
UC_no macroscopic inflammation_sigmoideum

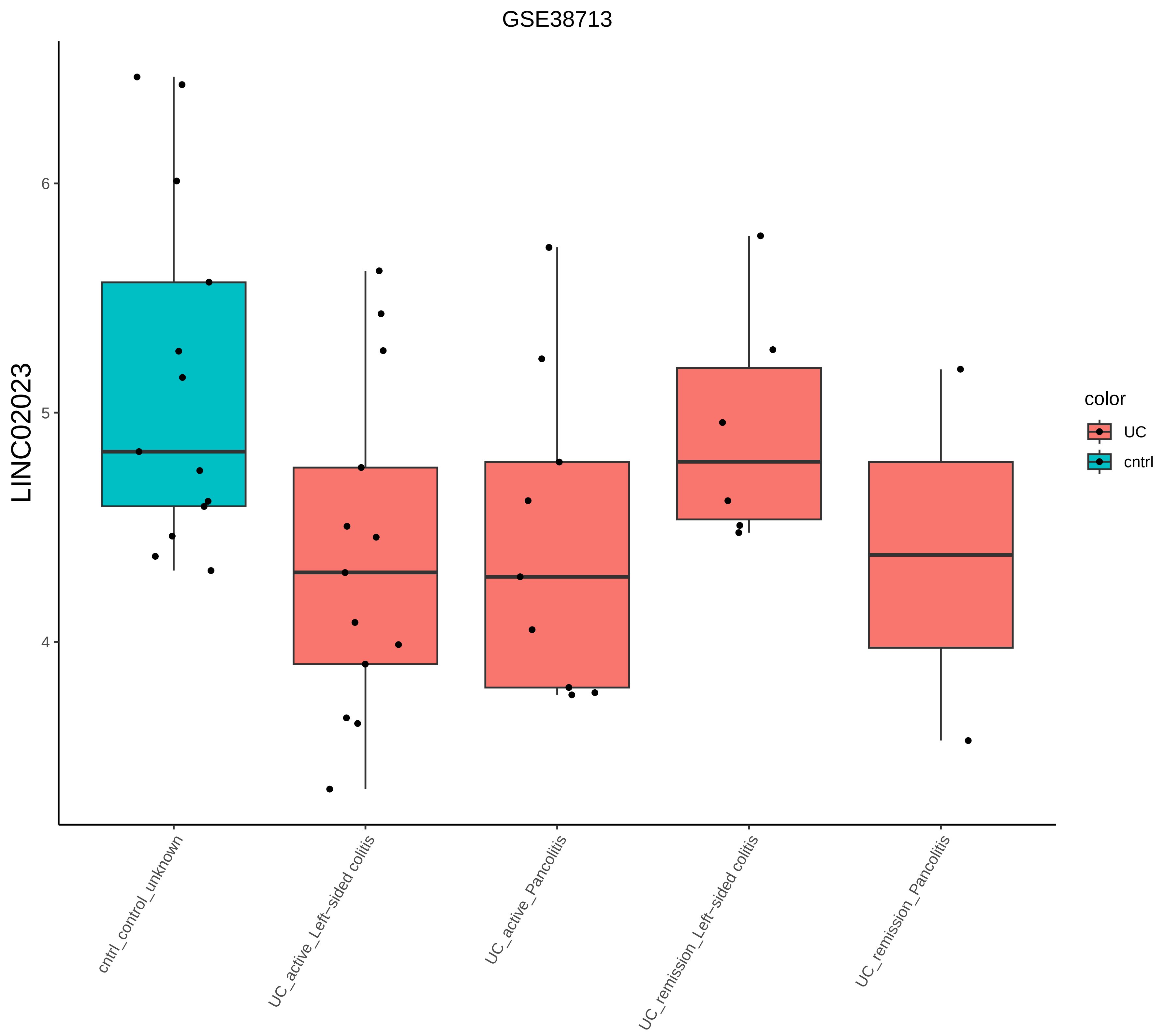
GSE38713
LINC02023
6
5
4
color
UC
cntrl
cntrl_control_unknown
UC_active_Left–sided colitis
UC_active_Pancolitis
UC_remission_Left–sided colitis
UC_remission_Pancolitis

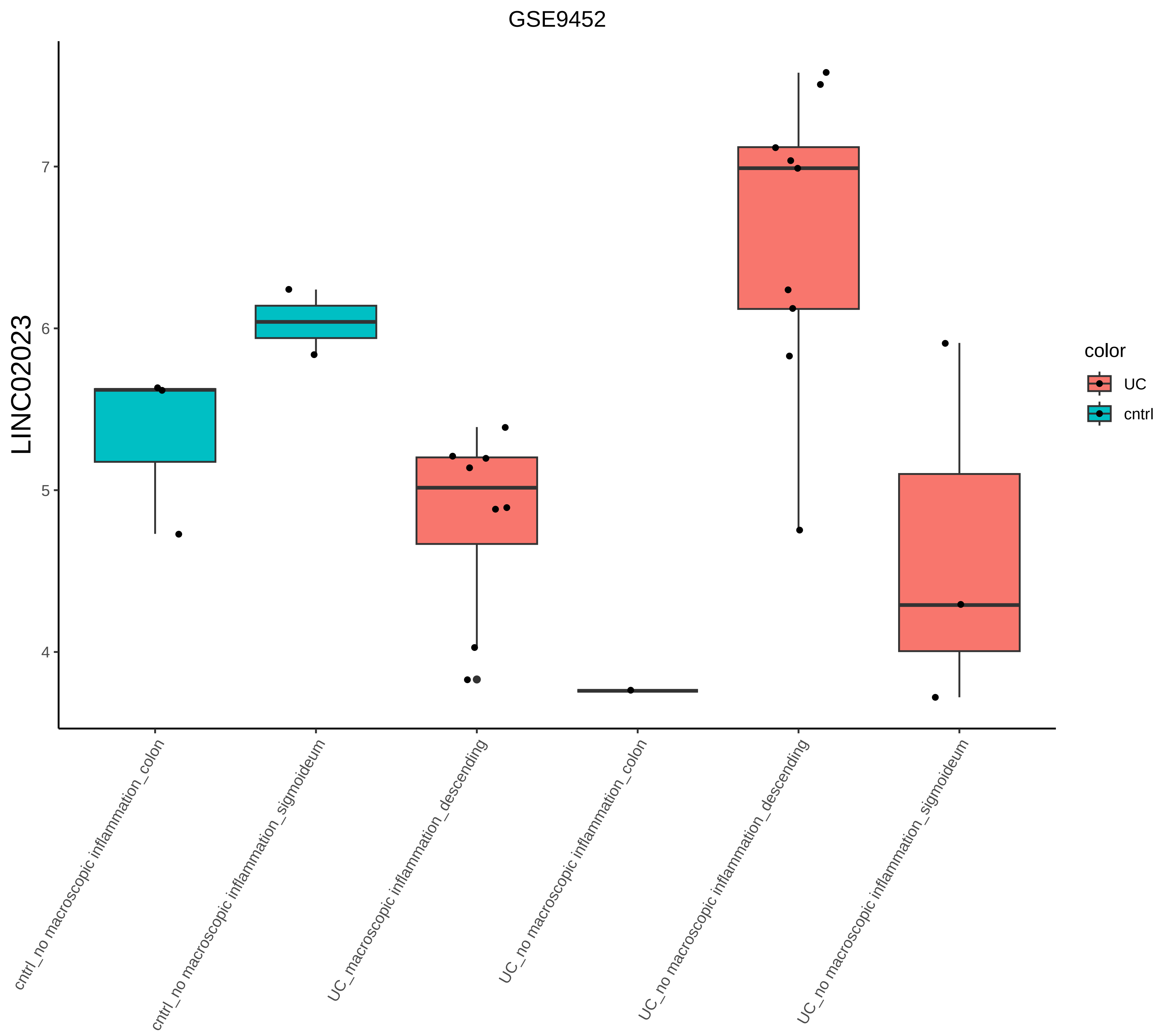
GSE9452
LINC02023
7
6
5
4
cntrl_no macroscopic inflammation_colon
cntrl_no macroscopic inflammation_sigmoideum
UC_macroscopic inflammation_descending
UC_no macroscopic inflammation_colon
UC_no macroscopic inflammation_descending
UC_no macroscopic inflammation_sigmoideum
color
UC
cntrl

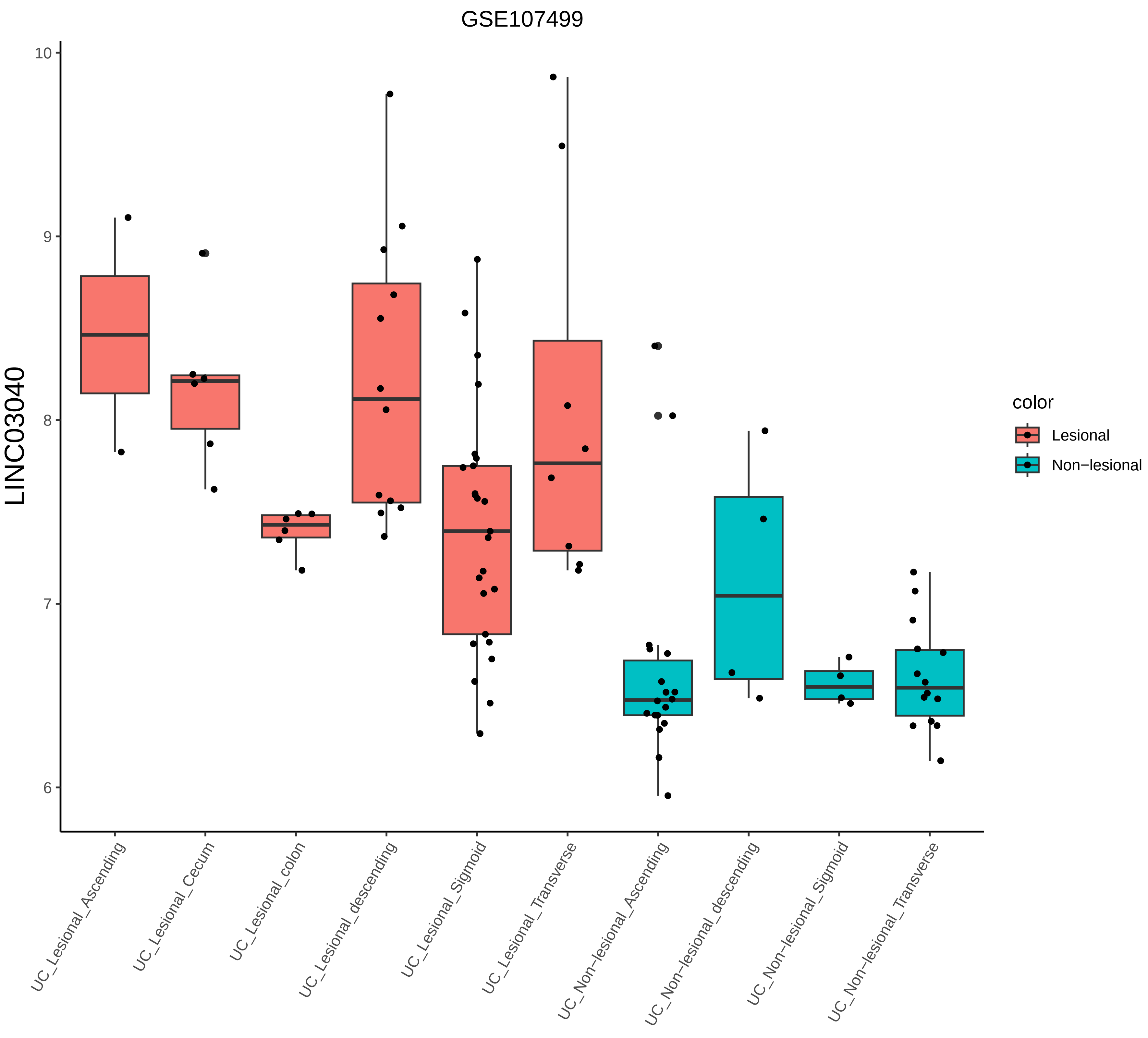
GSE107499
LINC03040
10
9
8
7
6
UC_Lesional_Ascending
UC_Lesional_Cecum
UC_Lesional_colon
UC_Lesional_descending
UC_Lesional_Sigmoid
UC_Lesional_Transverse
UC_Non−lesional_Ascending
UC_Non−lesional_descending
UC_Non−lesional_Sigmoid
UC_Non−lesional_Transverse
color
Lesional
Non−lesional

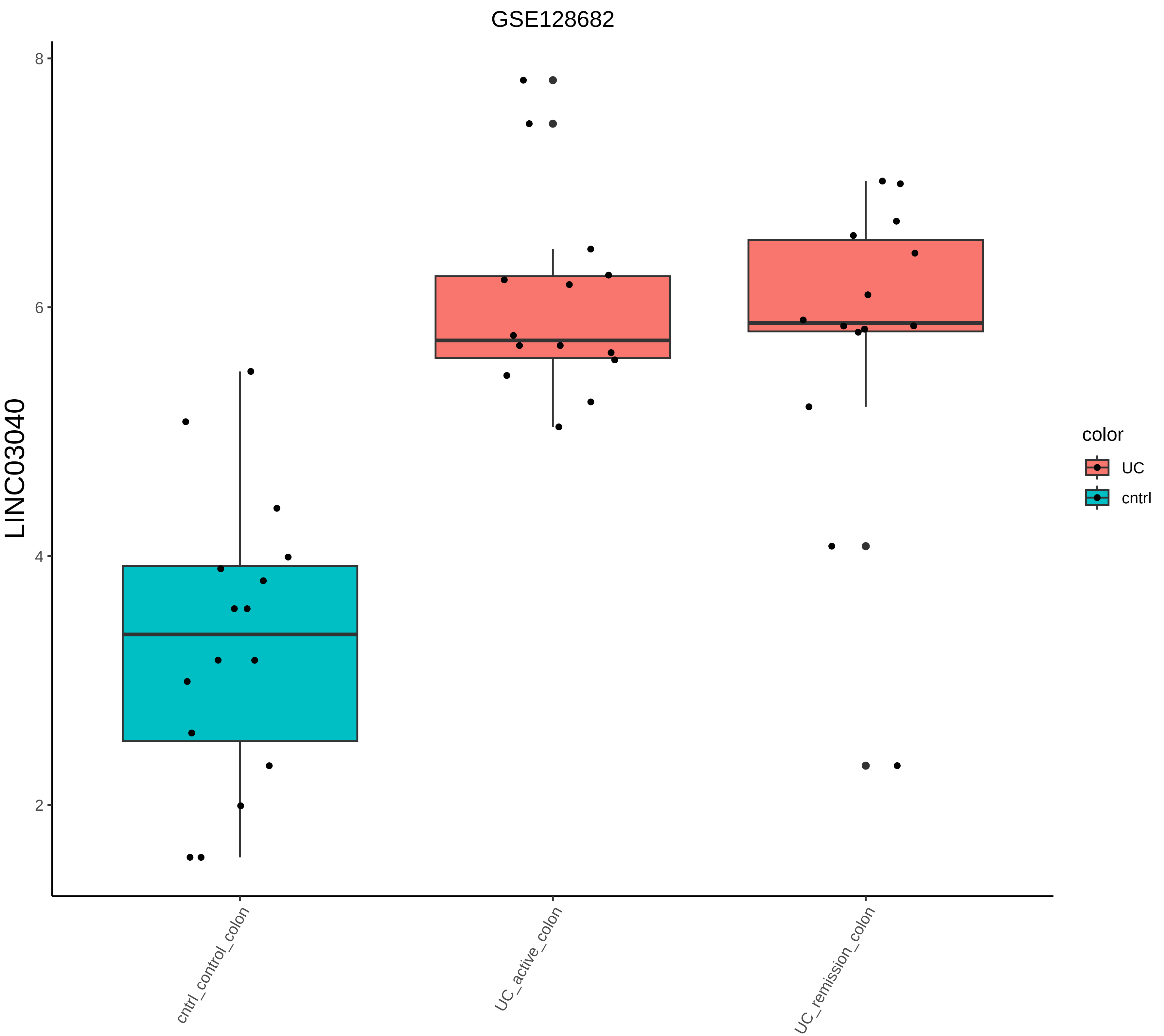
GSE128682
LINC03040
8
6
4
2
cntrl_control_colon
UC_active_colon
UC_remission_colon
color
UC
cntrl

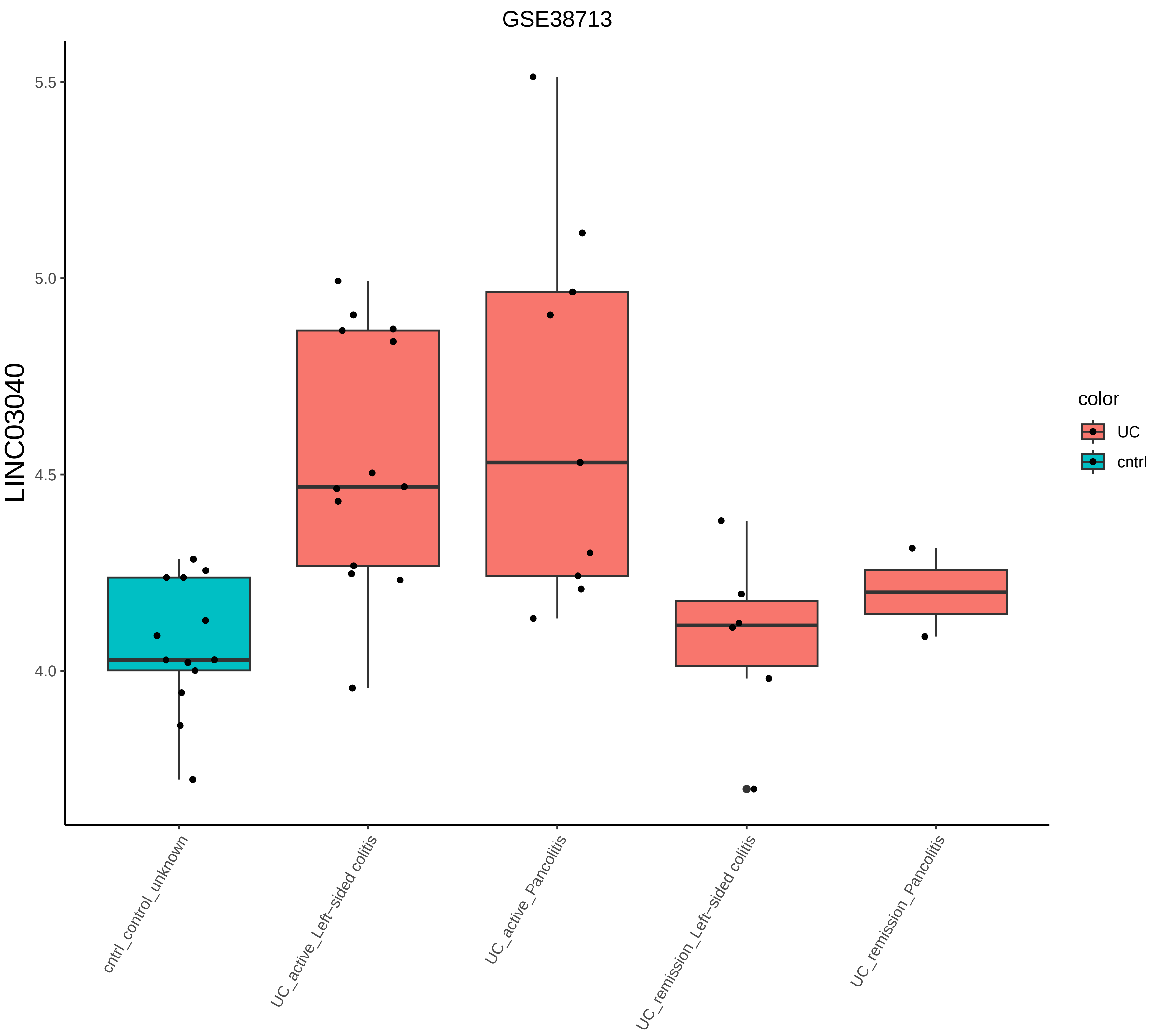

GSE38713
LINC03040
5.5
5.0
4.5
4.0
cntrl_control_unknown
UC_active_Left−sided colitis
UC_active_Pancolitis
UC_remission_Left−sided colitis
UC_remission_Pancolitis
color
UC
cntrl

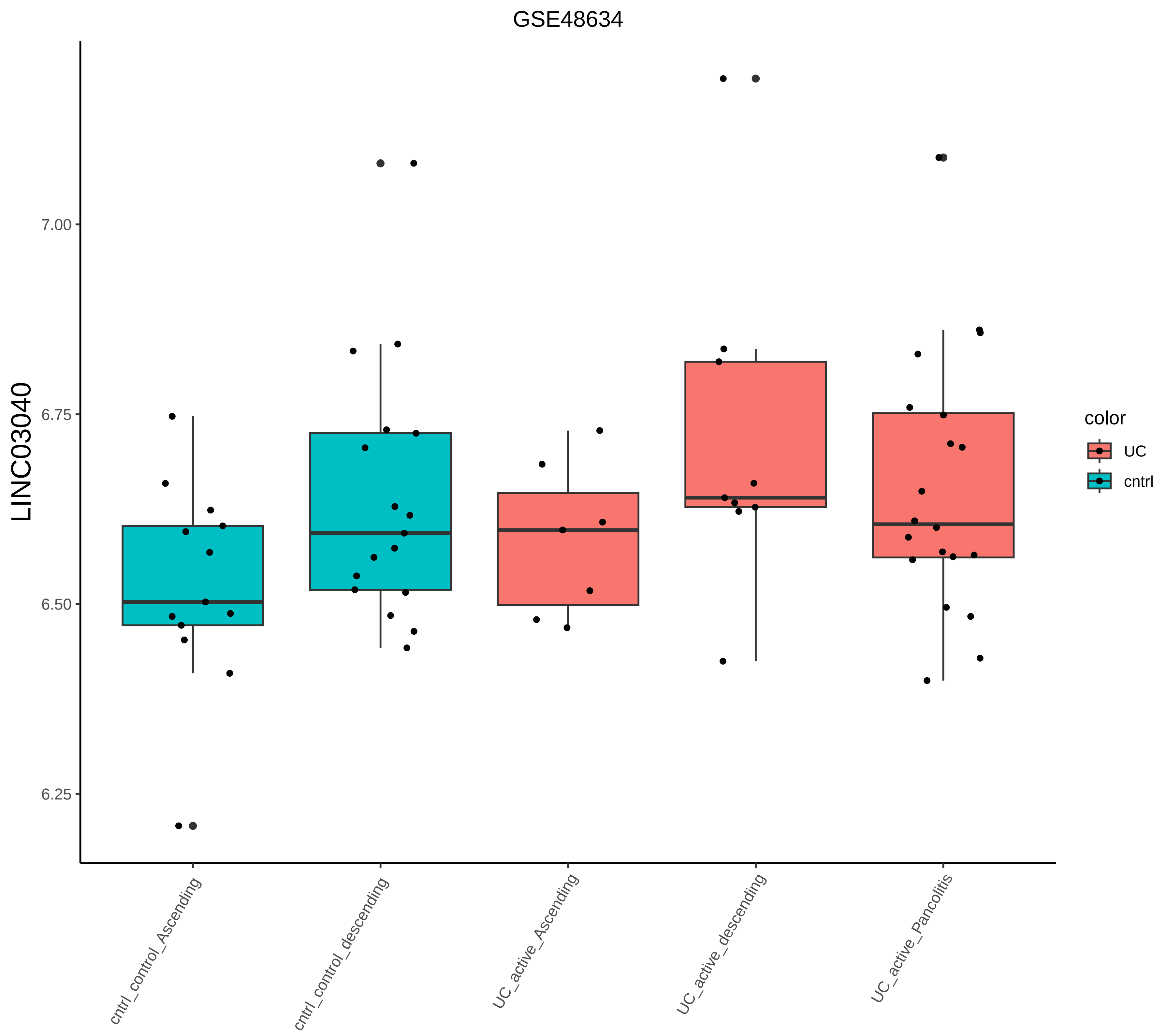

GSE48634
LINC03040
7.00
6.75
6.50
6.25
cntrl_control_Ascending
cntrl_control_descending
UC_active_Ascending
UC_active_descending
UC_active_Pancolitis
color
UC
cntrl

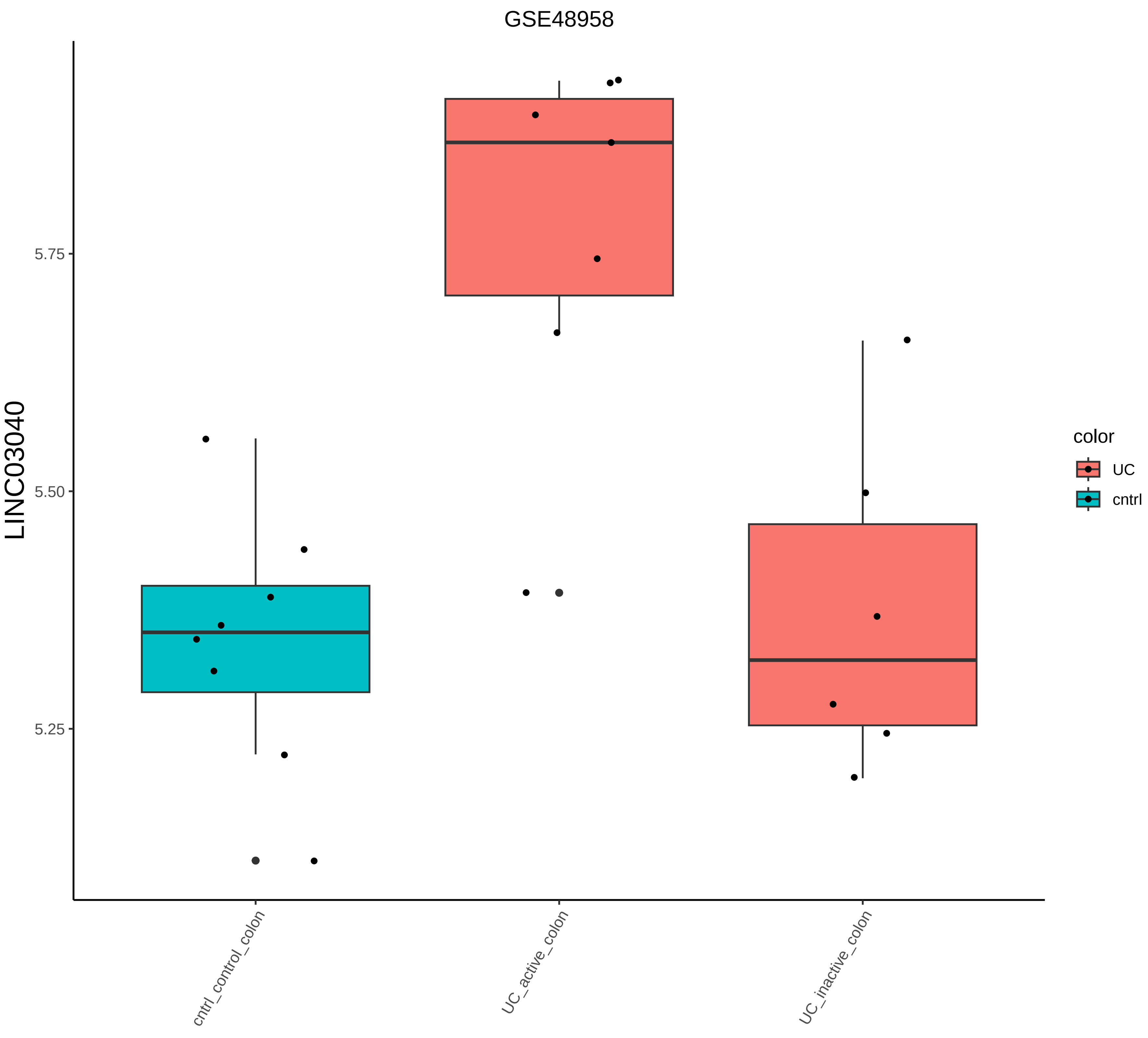
GSE48958
LINC03040
5.75
5.50
5.25
cntrl_control_colon
UC_active_colon
UC_inactive_colon
color
UC
cntrl

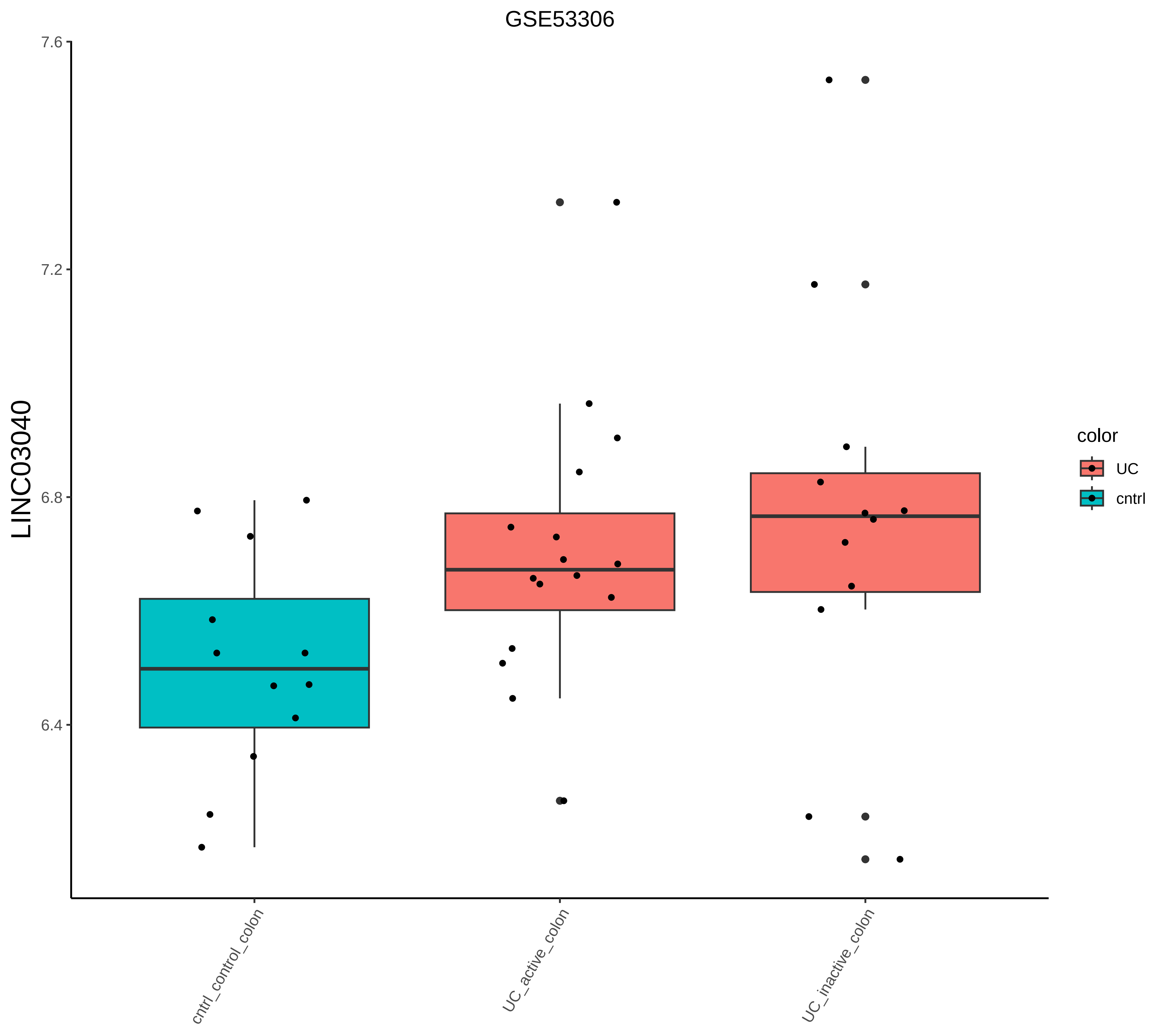
GSE53306
LINC03040
7.6
7.2
6.8
6.4
cntrl_control_colon
UC_active_colon
UC_inactive_colon
color
UC
cntrl

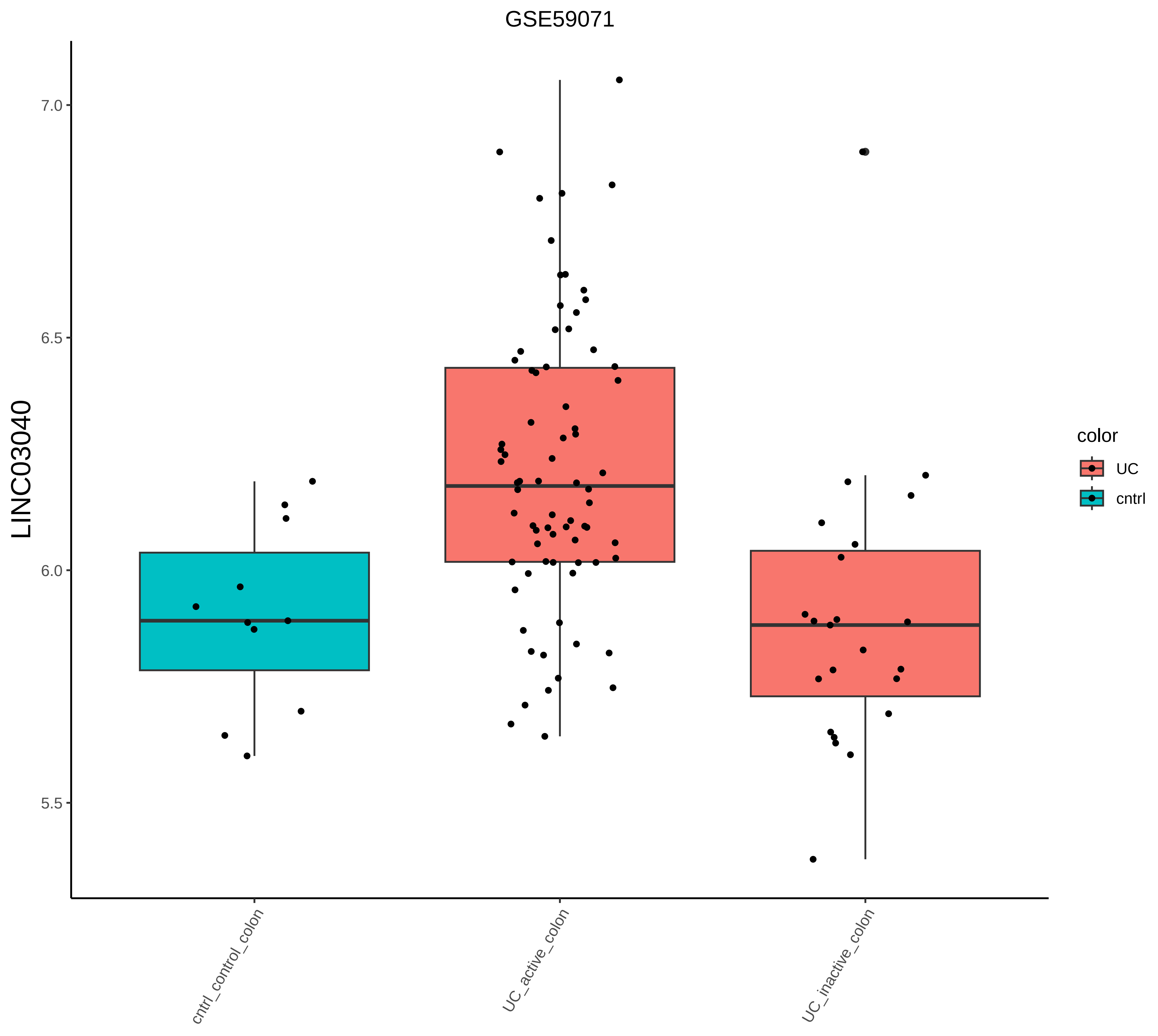
GSE59071
LINC03040
7.0
6.5
6.0
5.5
cntrl_control_colon
UC_active_colon
UC_inactive_colon
color
UC
cntrl

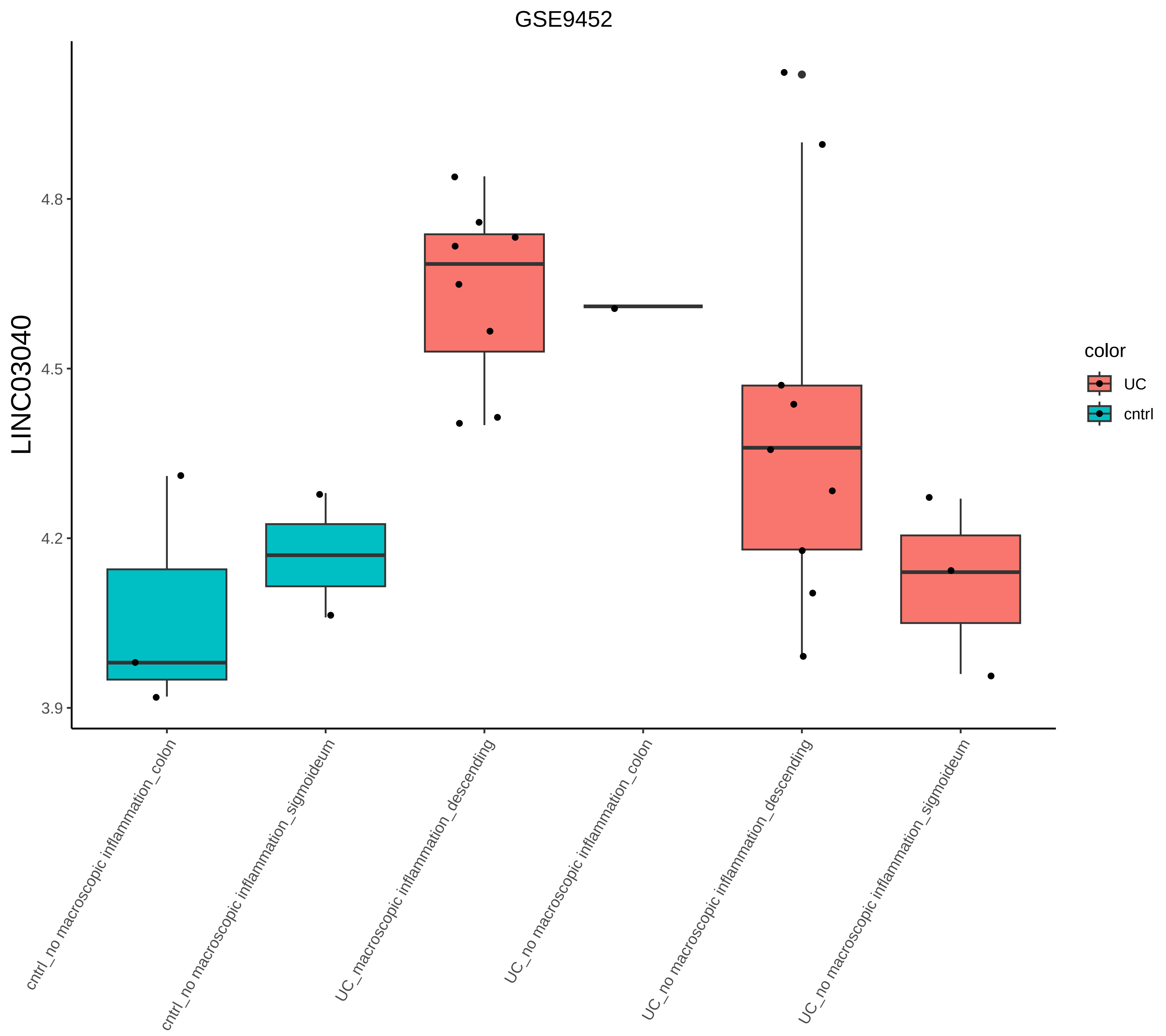

GSE9452
LINC03040
4.8
4.5
4.2
3.9
cntrl_no macroscopic inflammation_colon
cntrl_no macroscopic inflammation_sigmoideum
UC_macroscopic inflammation_descending
UC_no macroscopic inflammation_colon
UC_no macroscopic inflammation_descending
UC_no macroscopic inflammation_sigmoideum
color
UC
cntrl

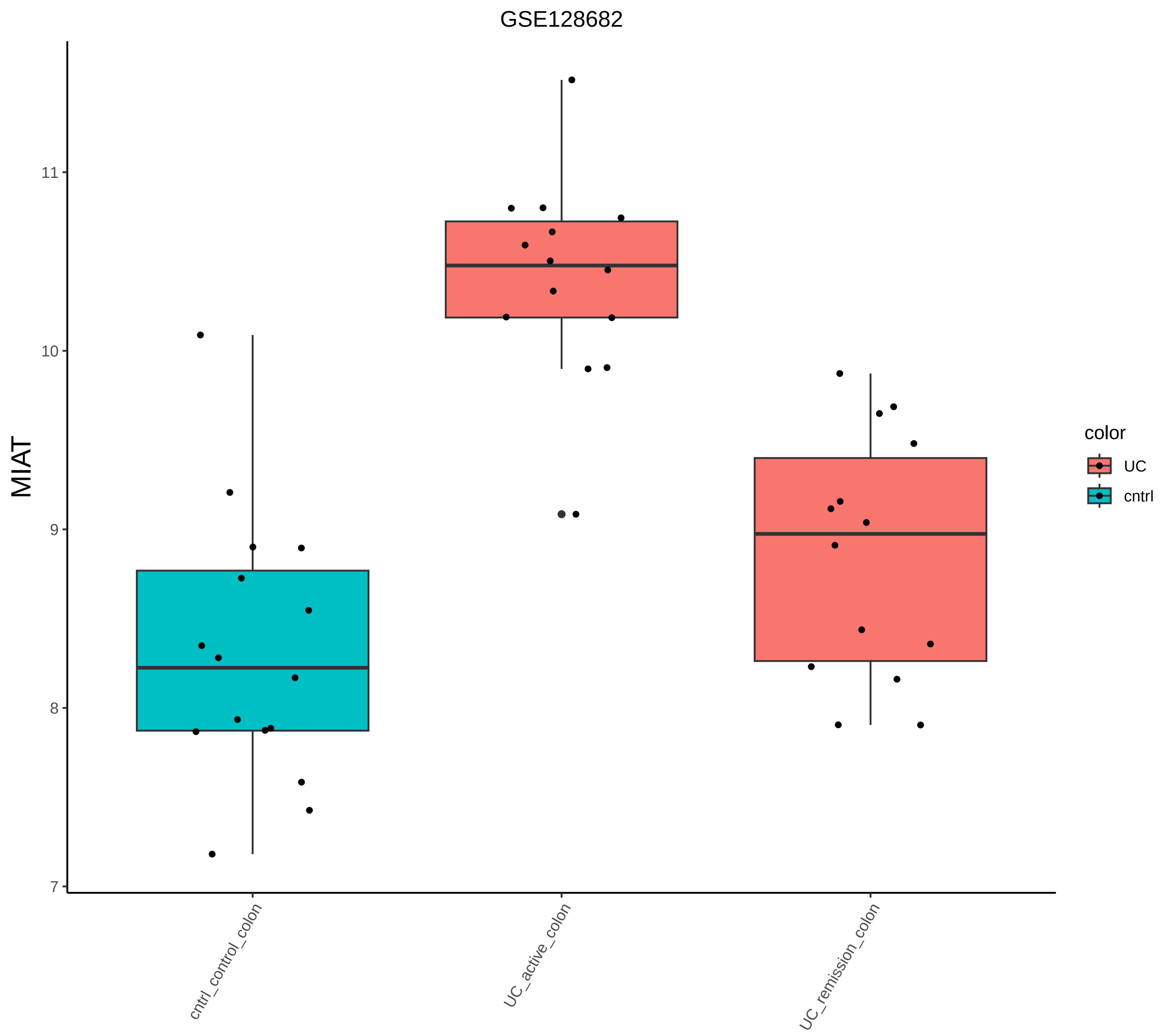

GSE128682
MIAT
11
10
9
8
7
cntrl_control_colon
UC_active_colon
UC_remission_colon
color
UC
cntrl

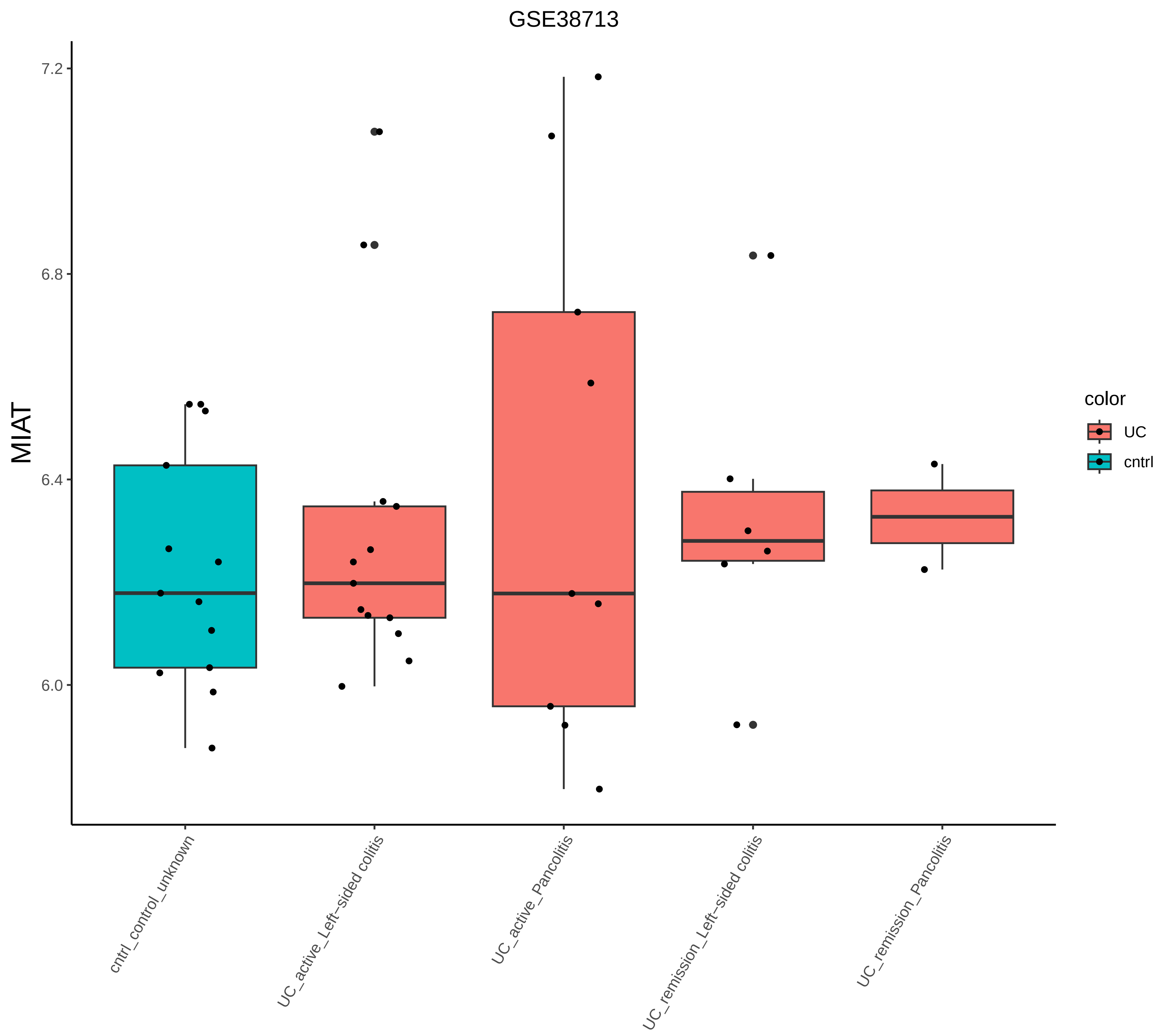
GSE38713
MIAT
7.2
6.8
6.4
6.0
cntrl_control_unknown
UC_active_Left−sided colitis
UC_active_Pancolitis
UC_remission_Left−sided colitis
UC_remission_Pancolitis
color
UC
cntrl

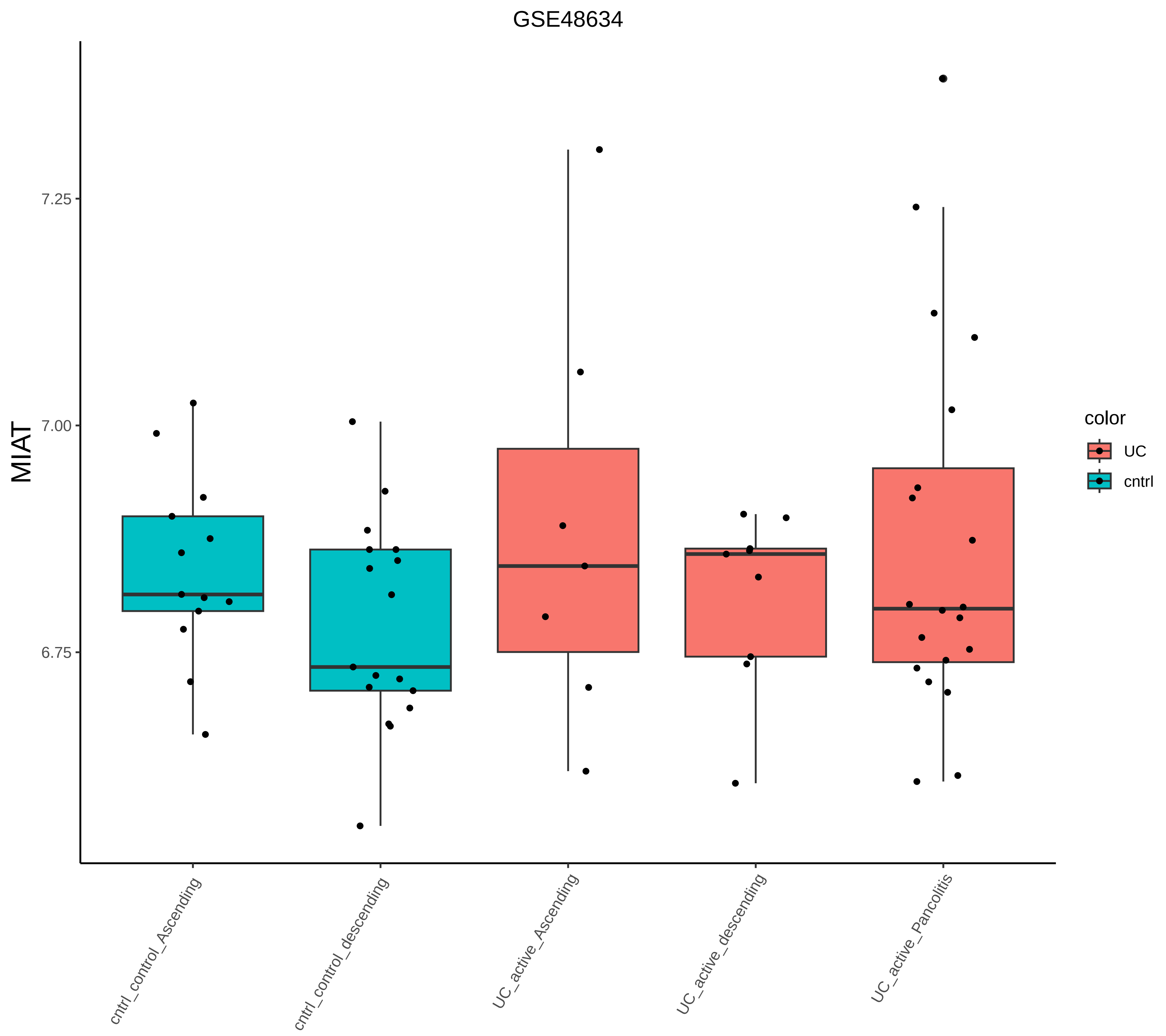

GSE48634
MIAT
7.25
7.00
6.75
cntrl_control_Ascending
cntrl_control_descending
UC_active_Ascending
UC_active_descending
UC_active_Pancolitis
color
UC
cntrl

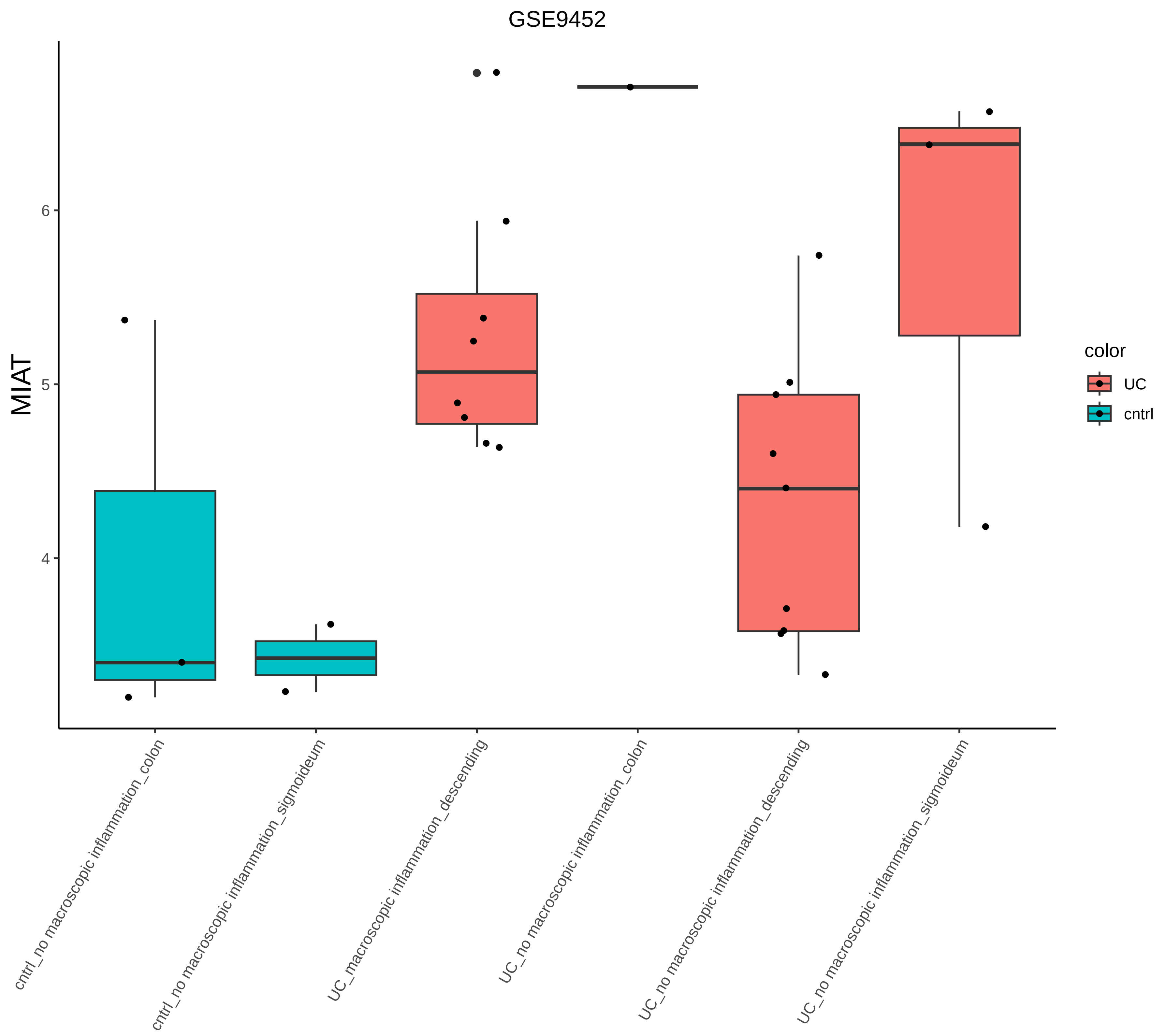
GSE9452
MIAT
6
5
4
color
UC
cntrl
cntrl_no macroscopic inflammation_colon
cntrl_no macroscopic inflammation_sigmoideum
UC_macroscopic inflammation_descending
UC_no macroscopic inflammation_colon
UC_no macroscopic inflammation_descending
UC_no macroscopic inflammation_sigmoideum

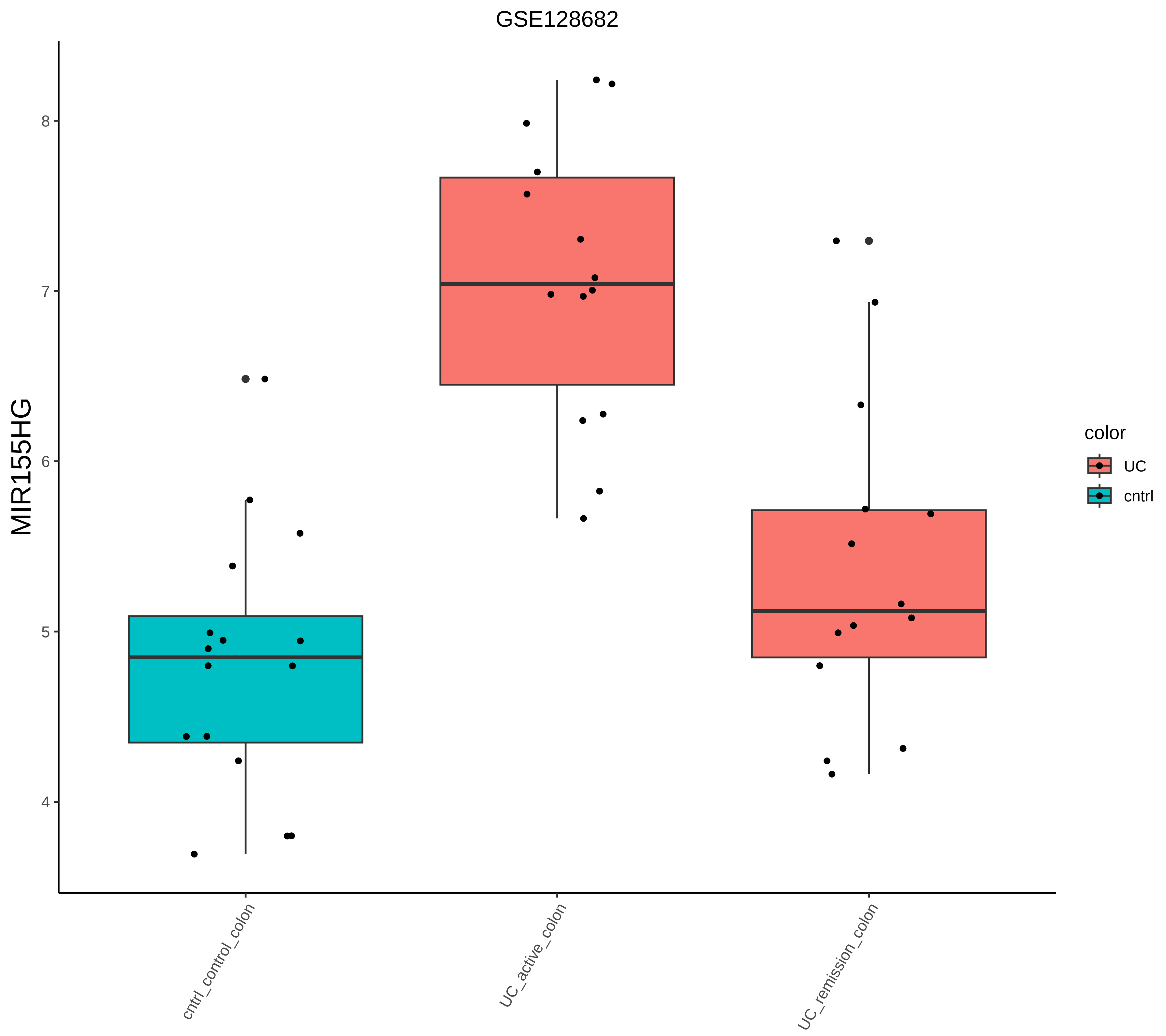
GSE128682
MIR155HG
8
7
6
5
4
cntrl_control_colon
UC_active_colon
UC_remission_colon
color
UC
cntrl

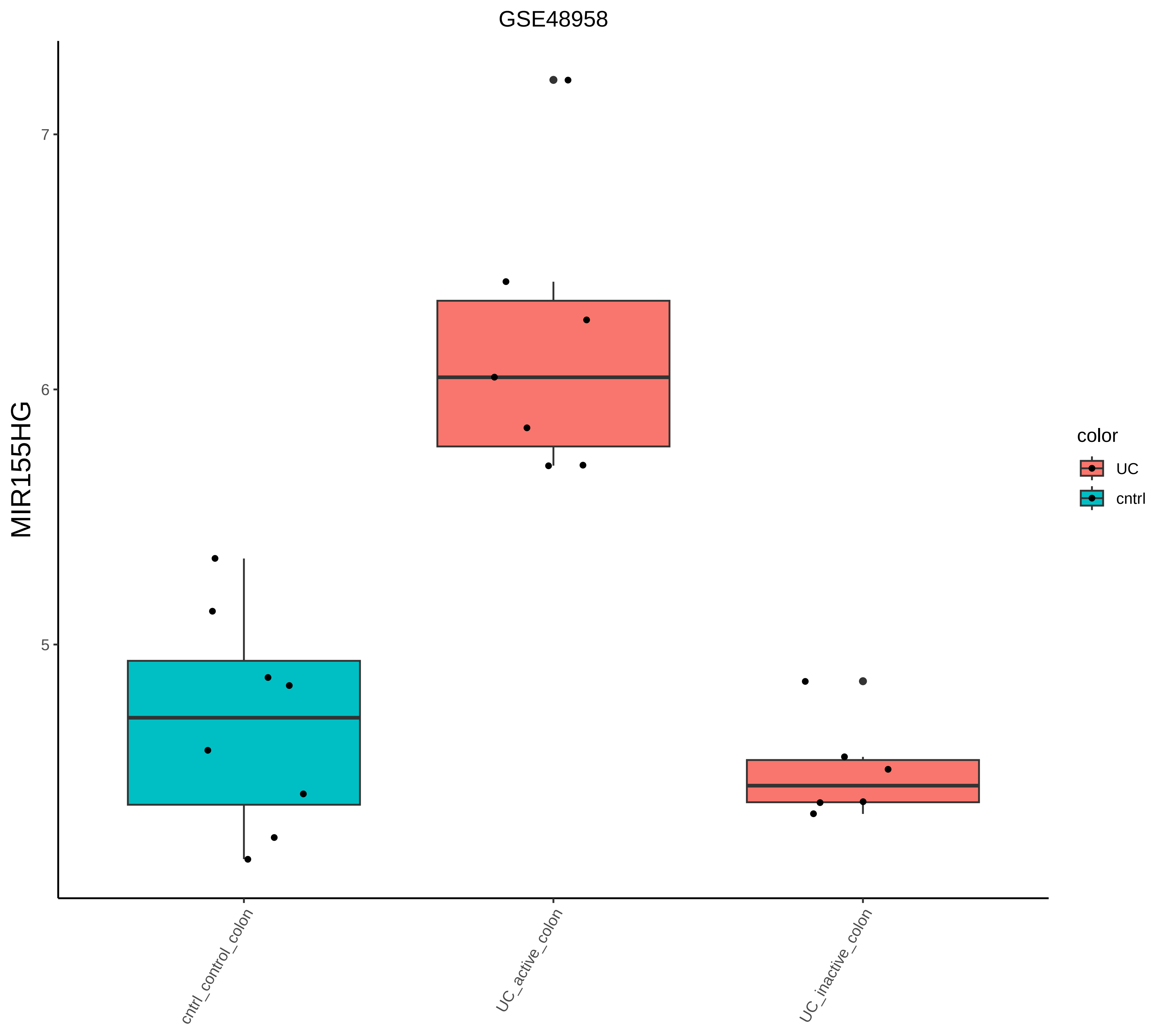

GSE48958
MIR155HG
7
6
5
cntrl_control_colon
UC_active_colon
UC_inactive_colon
color
UC
cntrl

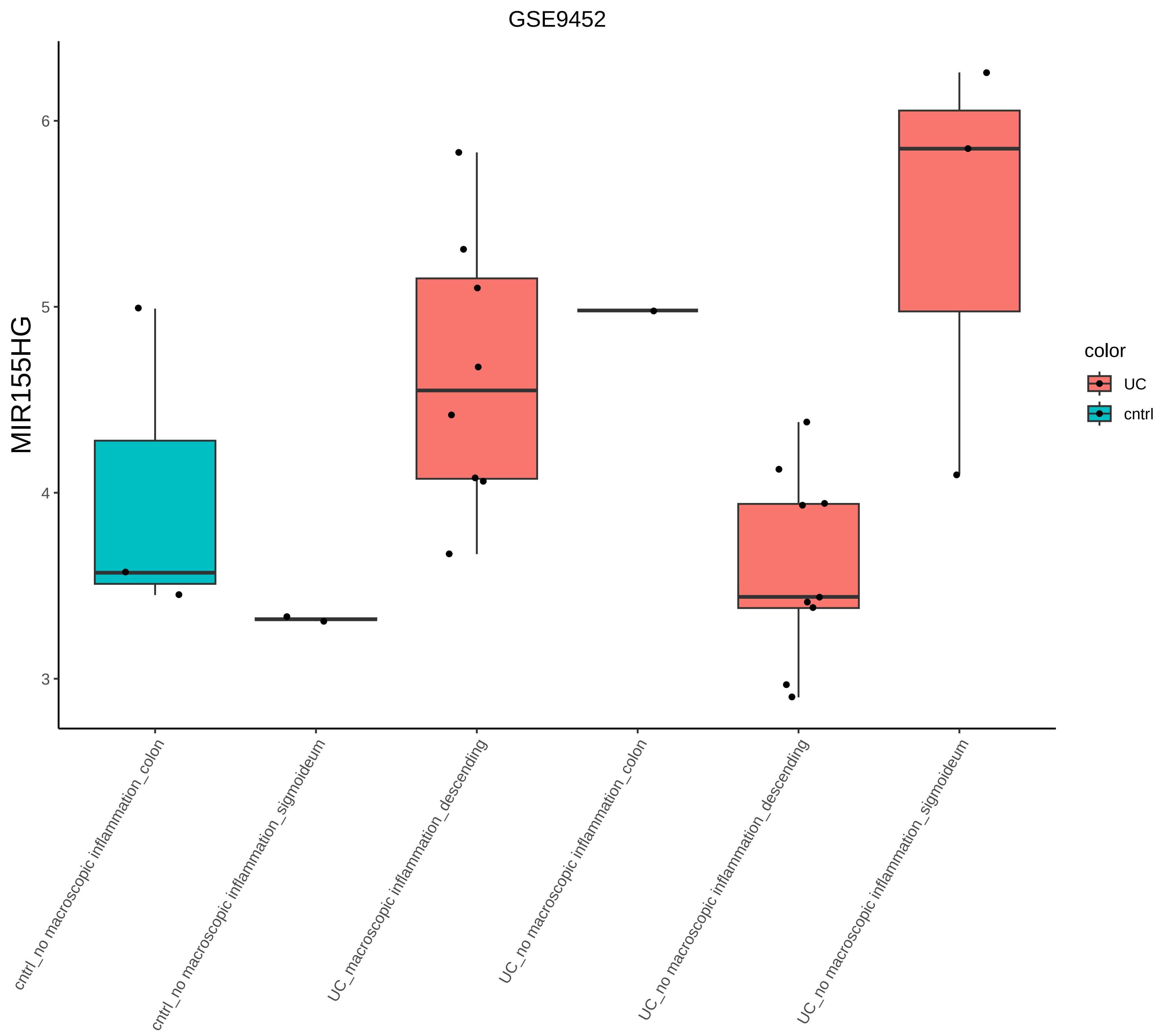
GSE9452
MIR155HG
6
5
4
3
color
UC
cntrl
cntrl_no macroscopic inflammation_colon
cntrl_no macroscopic inflammation_sigmoideum
UC_macroscopic inflammation_descending
UC_no macroscopic inflammation_colon
UC_no macroscopic inflammation_descending
UC_no macroscopic inflammation_sigmoideum

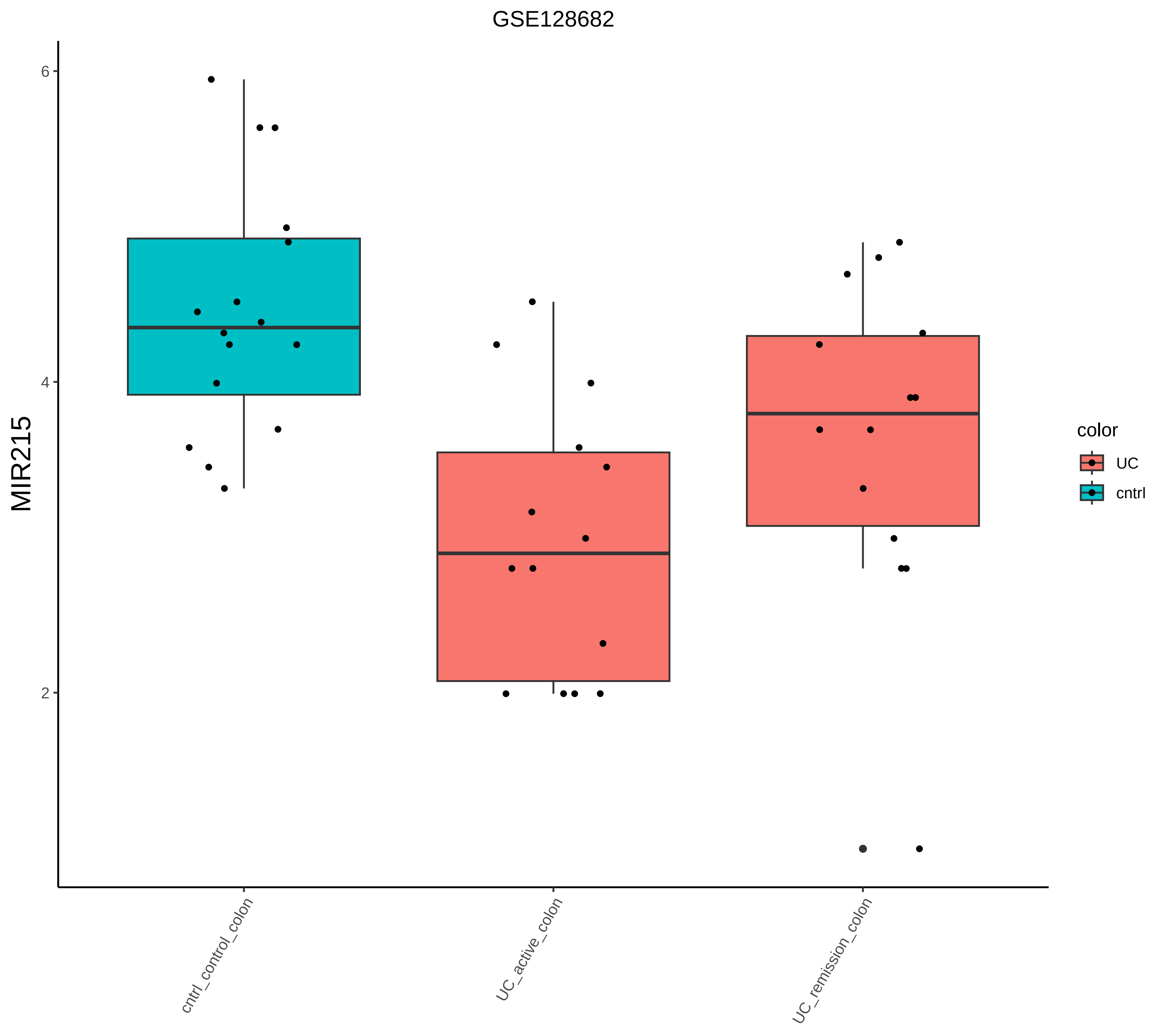

GSE128682
MIR215
6
4
2
cntrl_control_colon
UC_active_colon
UC_remission_colon
color
UC
cntrl

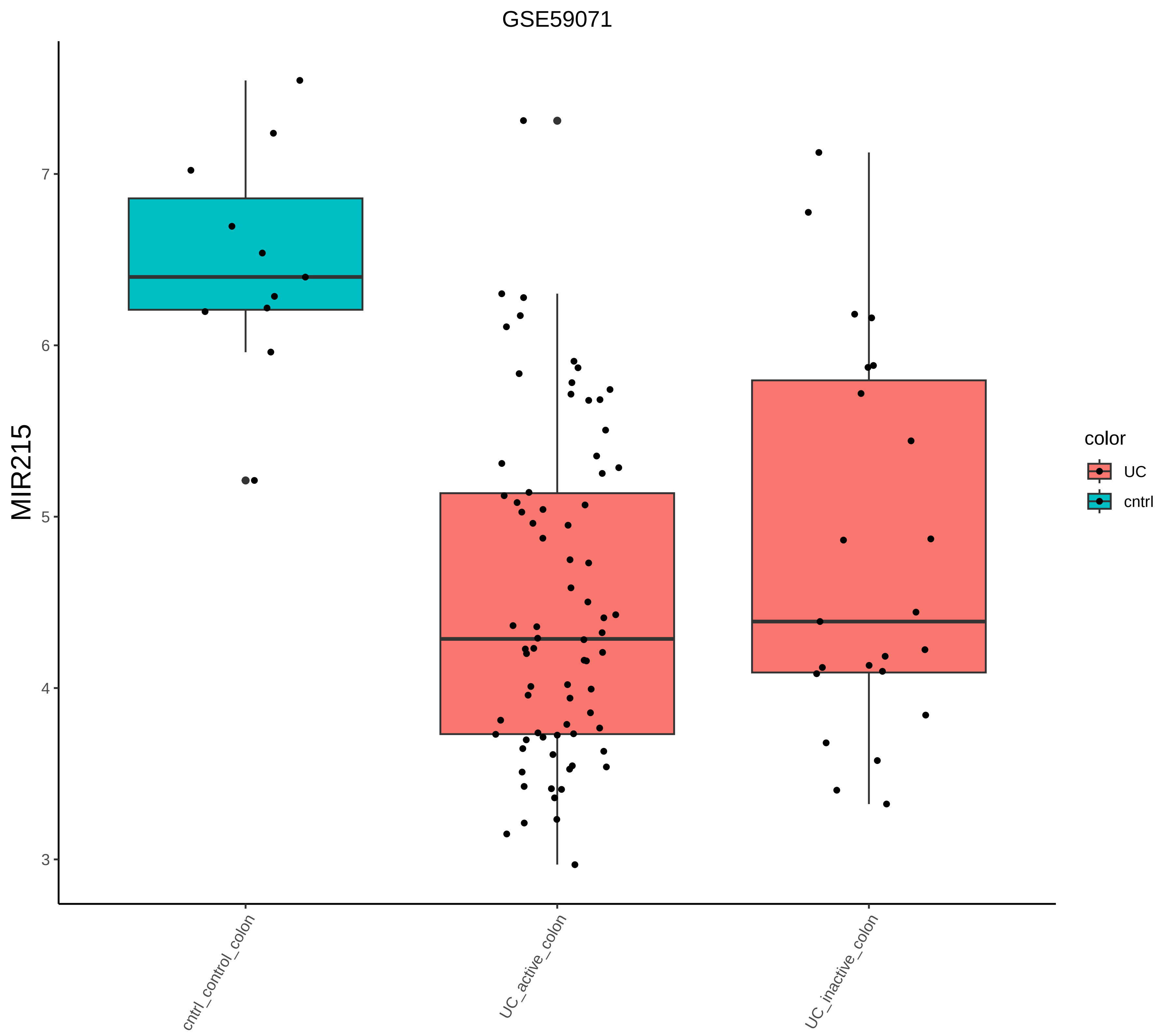

GSE59071
MIR215
7
6
5
4
3
cntrl_control_colon
UC_active_colon
UC_inactive_colon
color
UC
cntrl

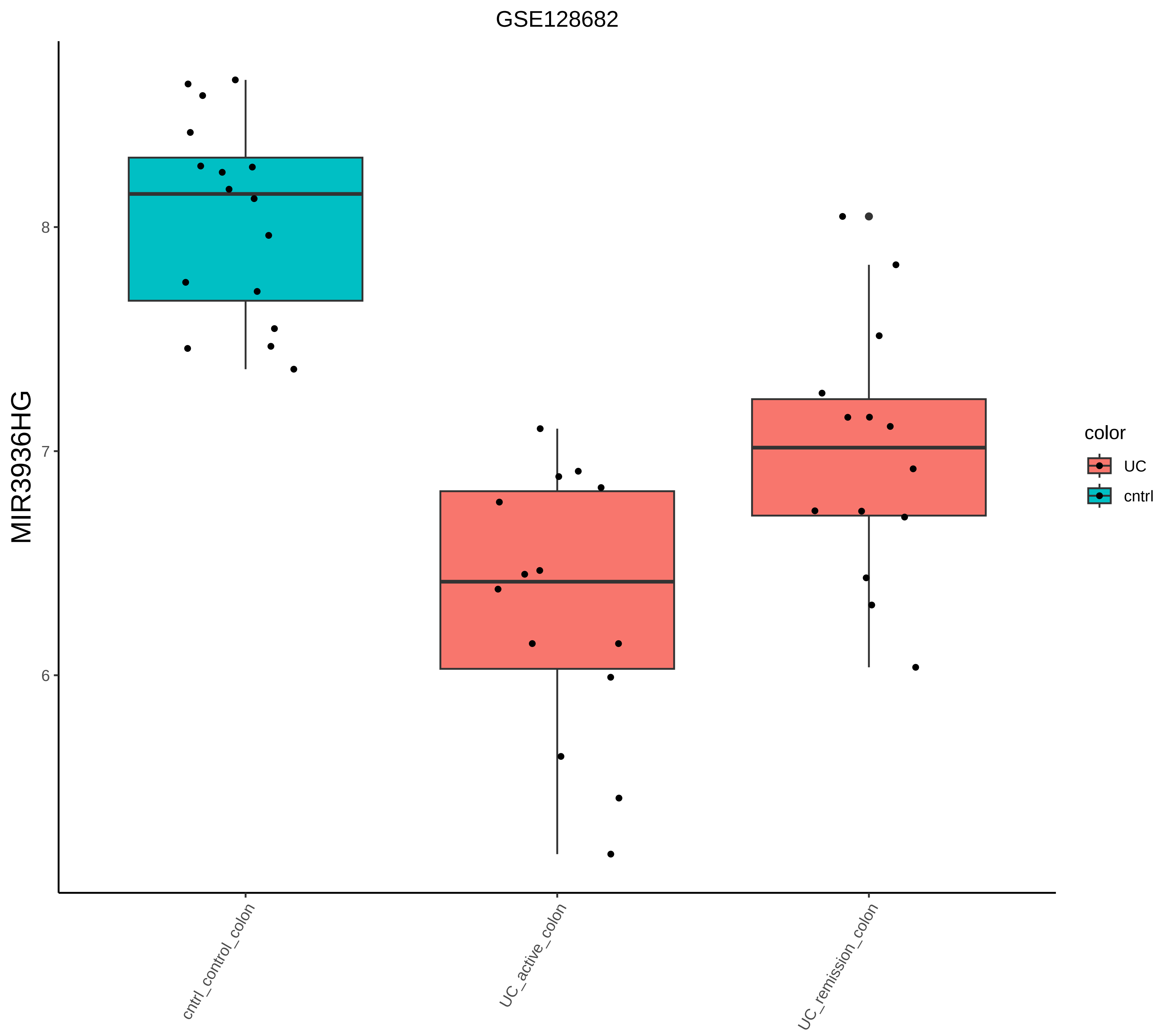

GSE128682
MIR3936HG
8
7
6
cntrl_control_colon
UC_active_colon
UC_remission_colon
color
UC
cntrl

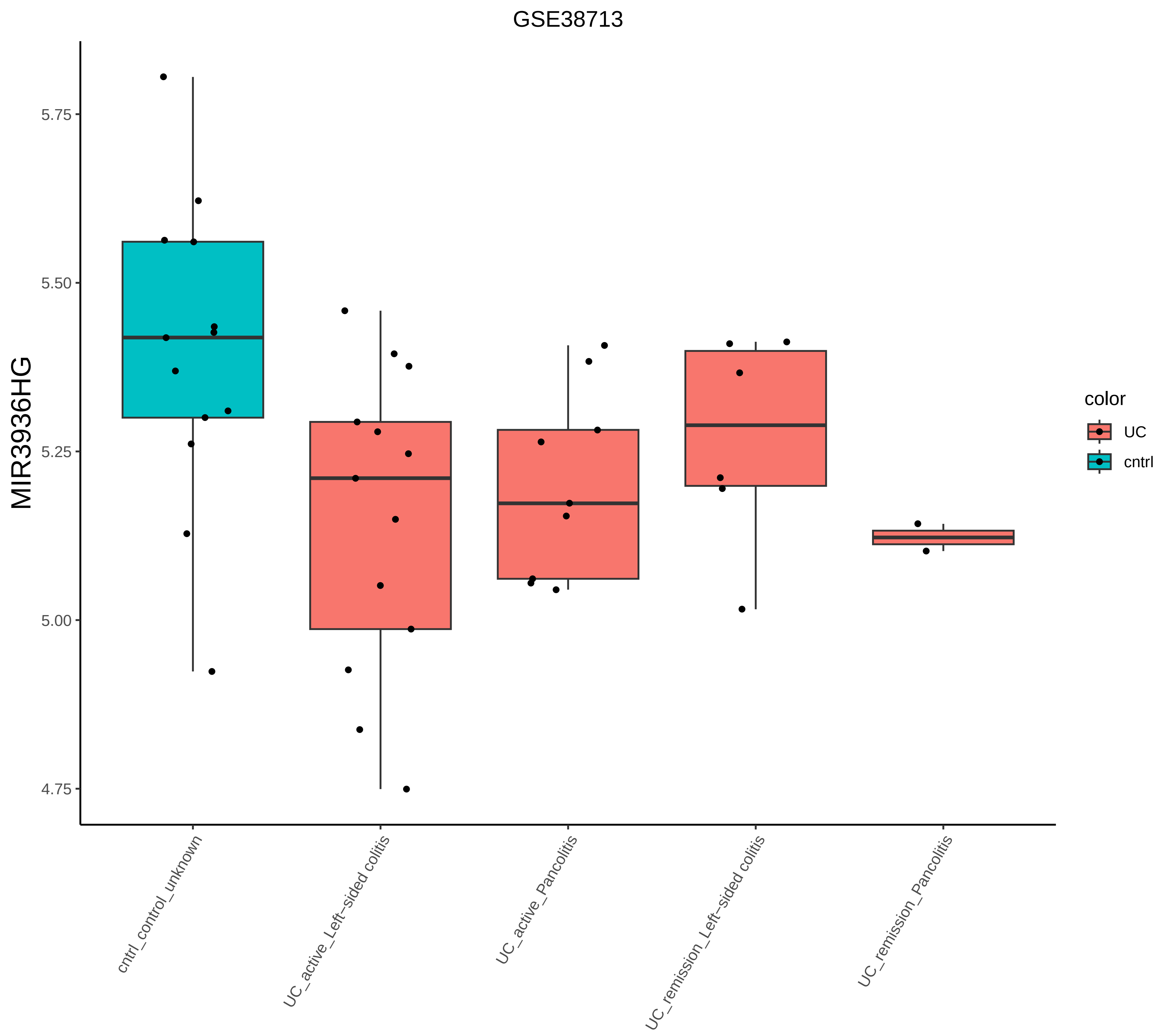

GSE38713
MIR3936HG
5.75
5.50
5.25
5.00
4.75
cntrl_control_unknown
UC_active_Left−sided colitis
UC_active_Pancolitis
UC_remission_Left−sided colitis
UC_remission_Pancolitis
color
UC
cntrl

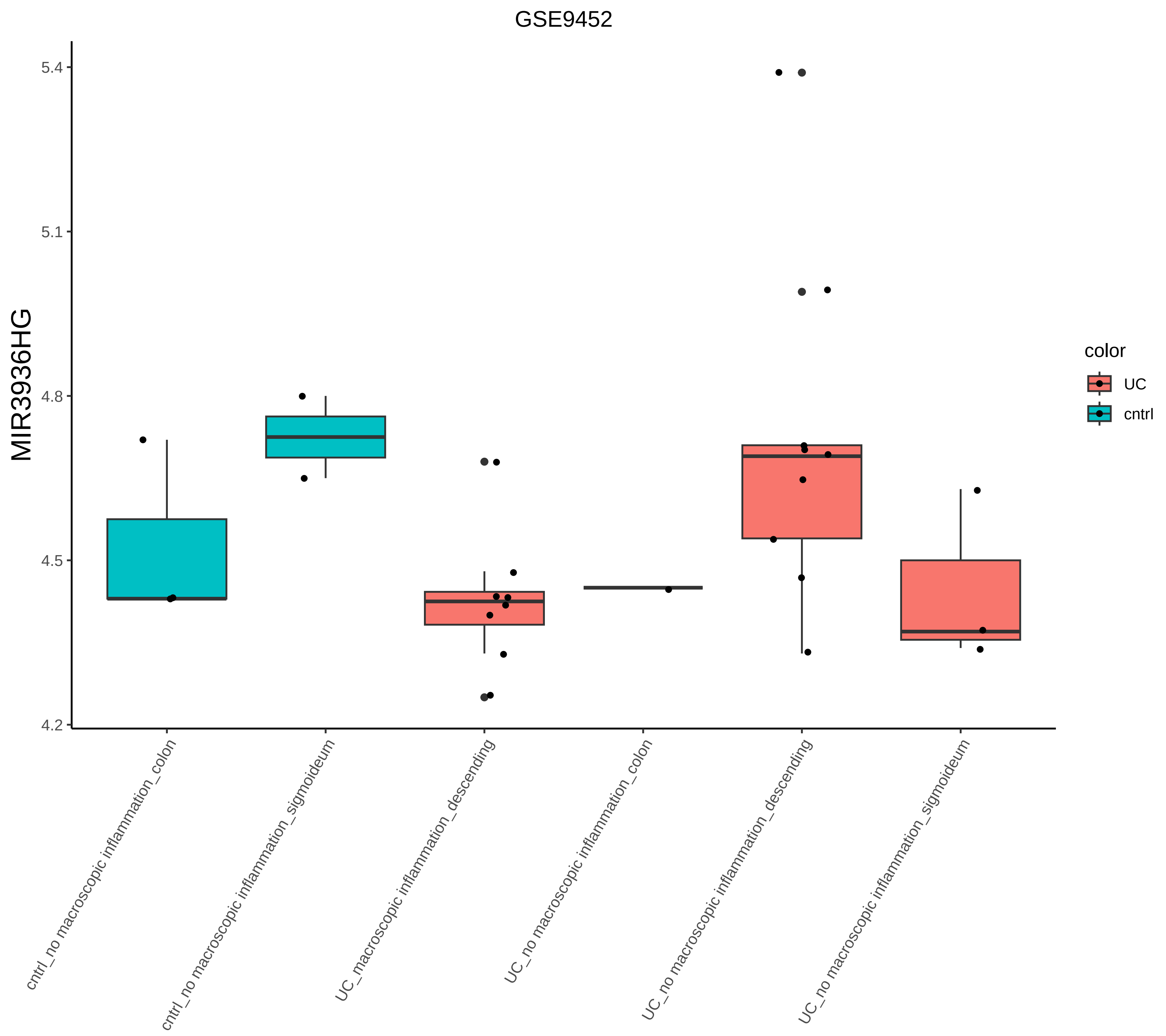

GSE9452
MIR3936HG
5.4
5.1
4.8
4.5
4.2
color
UC
cntrl
cntrl_no macroscopic inflammation_colon
cntrl_no macroscopic inflammation_sigmoideum
UC_macroscopic inflammation_descending
UC_no macroscopic inflammation_colon
UC_no macroscopic inflammation_descending
UC_no macroscopic inflammation_sigmoideum

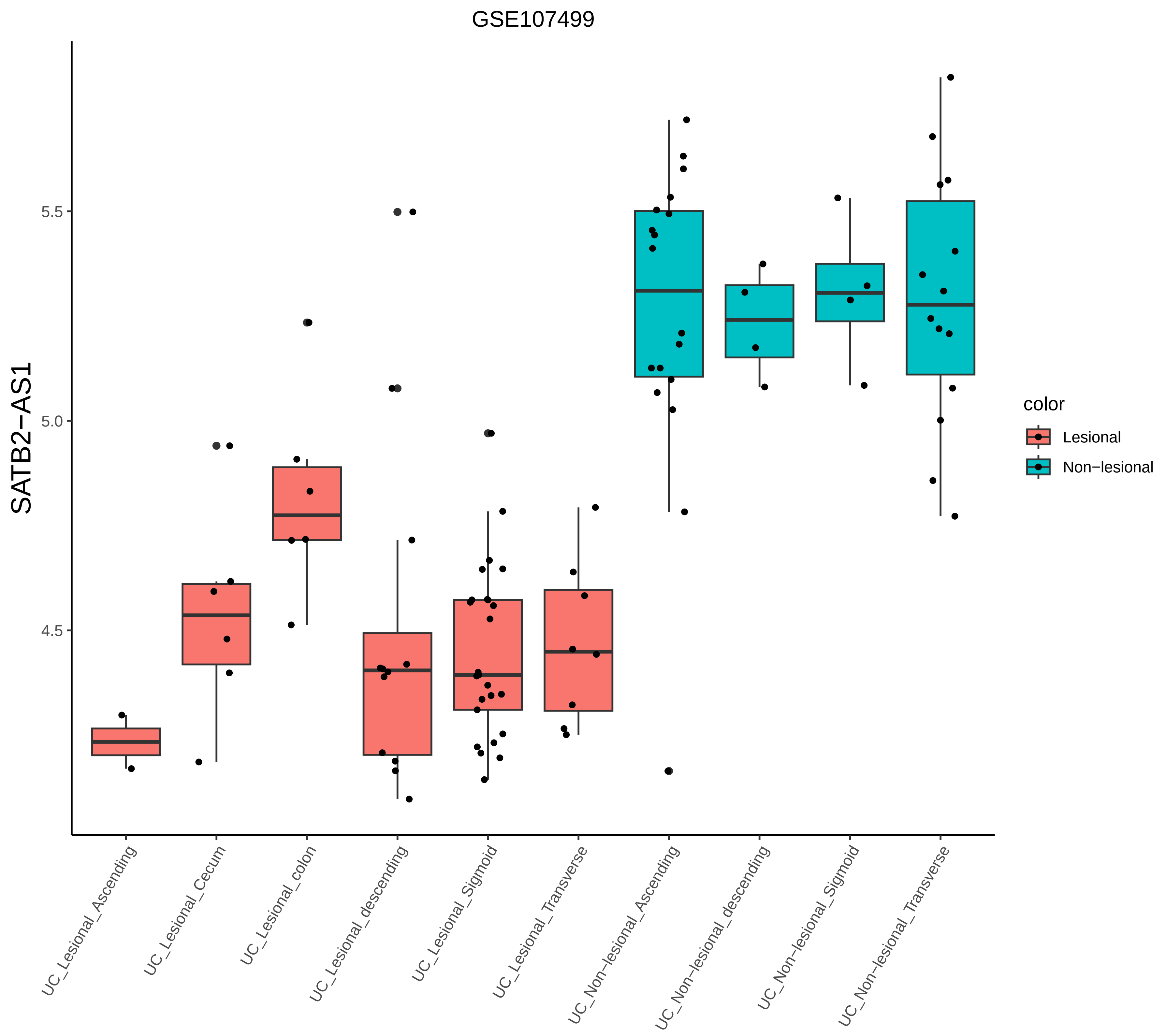
GSE107499
SATB2−AS1
5.5
5.0
4.5
UC_Lesional_Ascending
UC_Lesional_Cecum
UC_Lesional_colon
UC_Lesional_descending
UC_Lesional_Sigmoid
UC_Lesional_Transverse
UC_Non−lesional_Ascending
UC_Non−lesional_descending
UC_Non−lesional_Sigmoid
UC_Non−lesional_Transverse
color
Lesional
Non−lesional

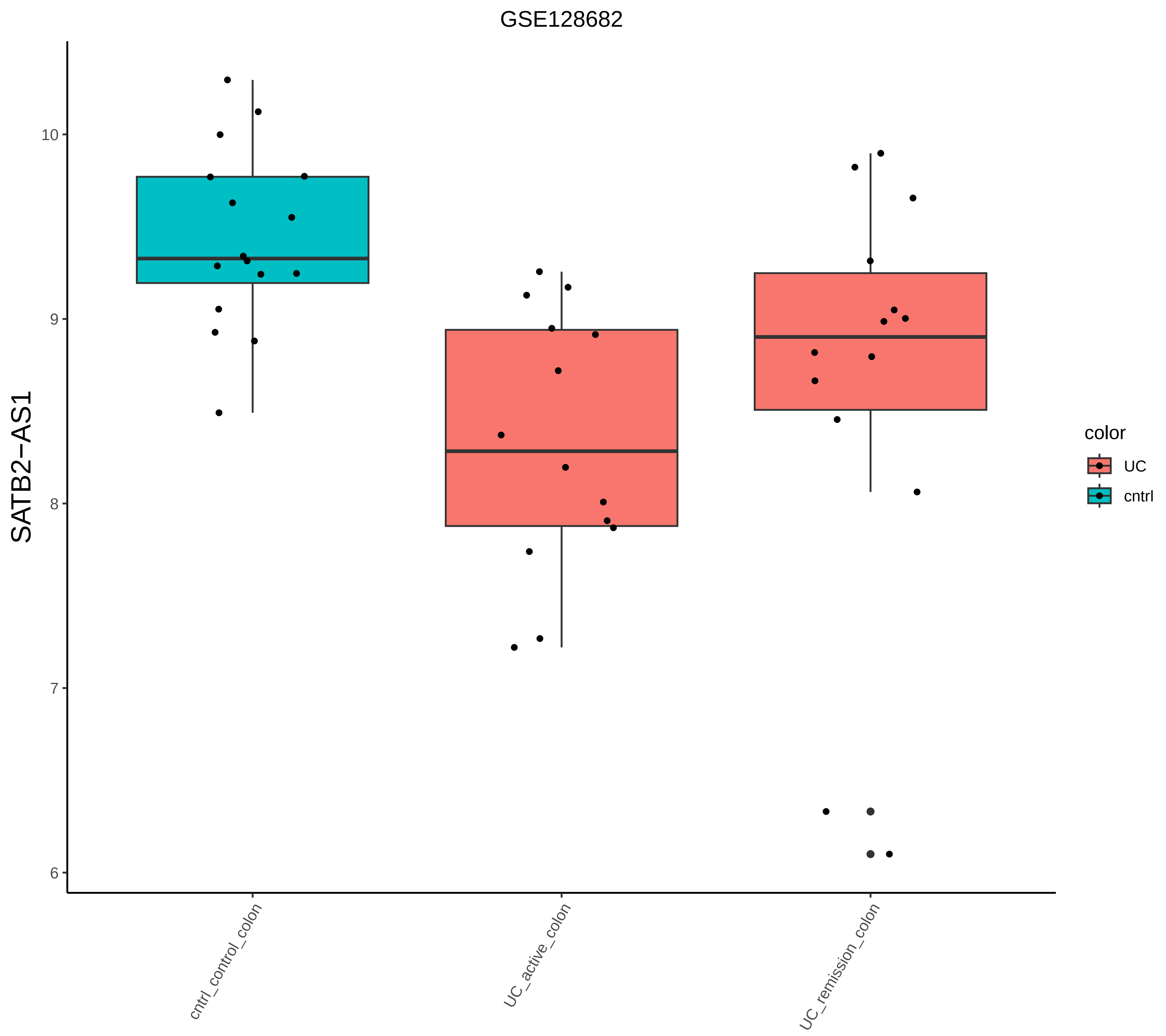
GSE128682
SATB2−AS1
10
9
8
7
6
cntrl_control_colon
UC_active_colon
UC_remission_colon
color
UC
cntrl

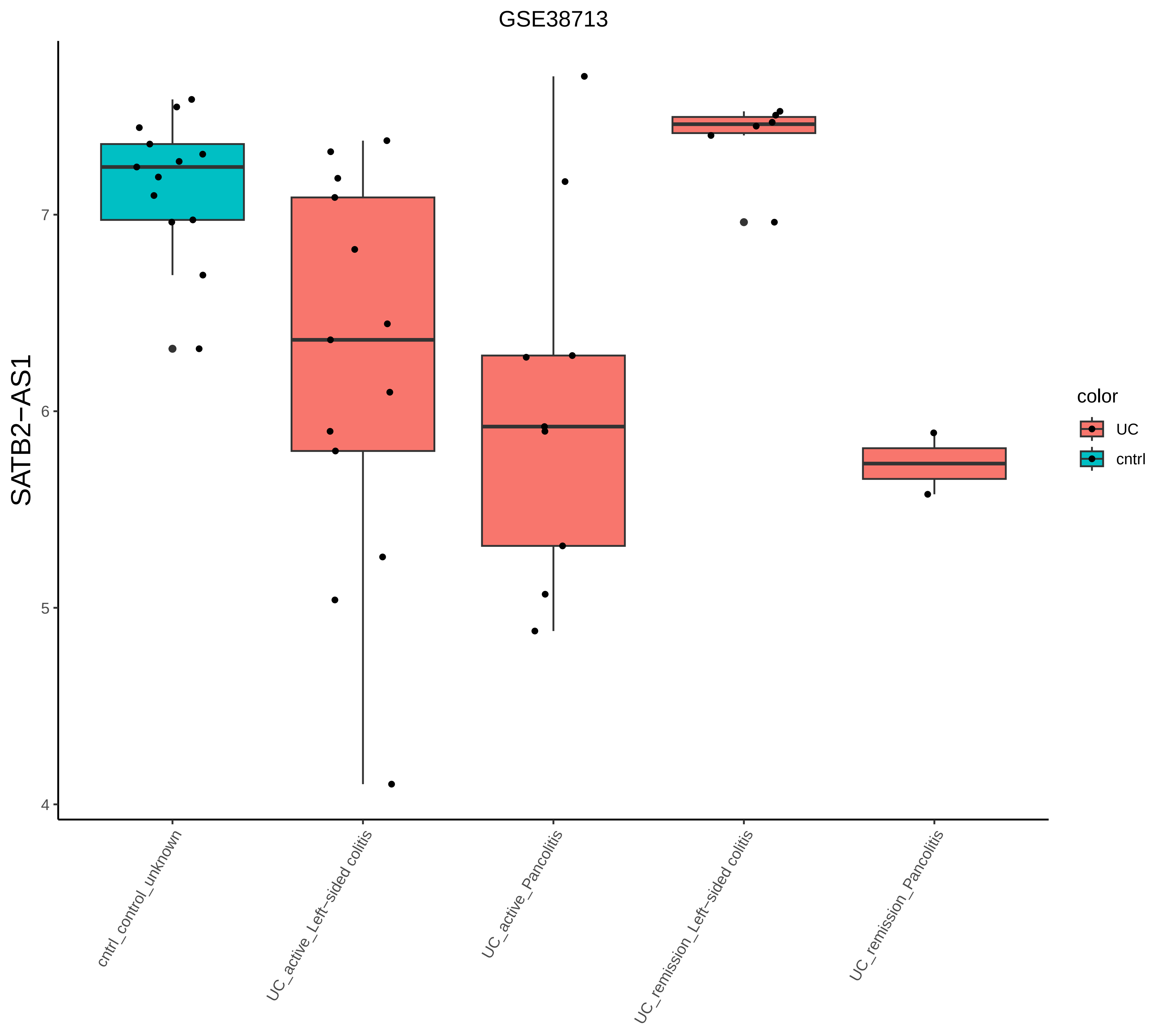
GSE38713
SATB2−AS1
7
6
5
4
cntrl_control_unknown
UC_active_Left−sided colitis
UC_active_Pancolitis
UC_remission_Left−sided colitis
UC_remission_Pancolitis
color
UC
cntrl

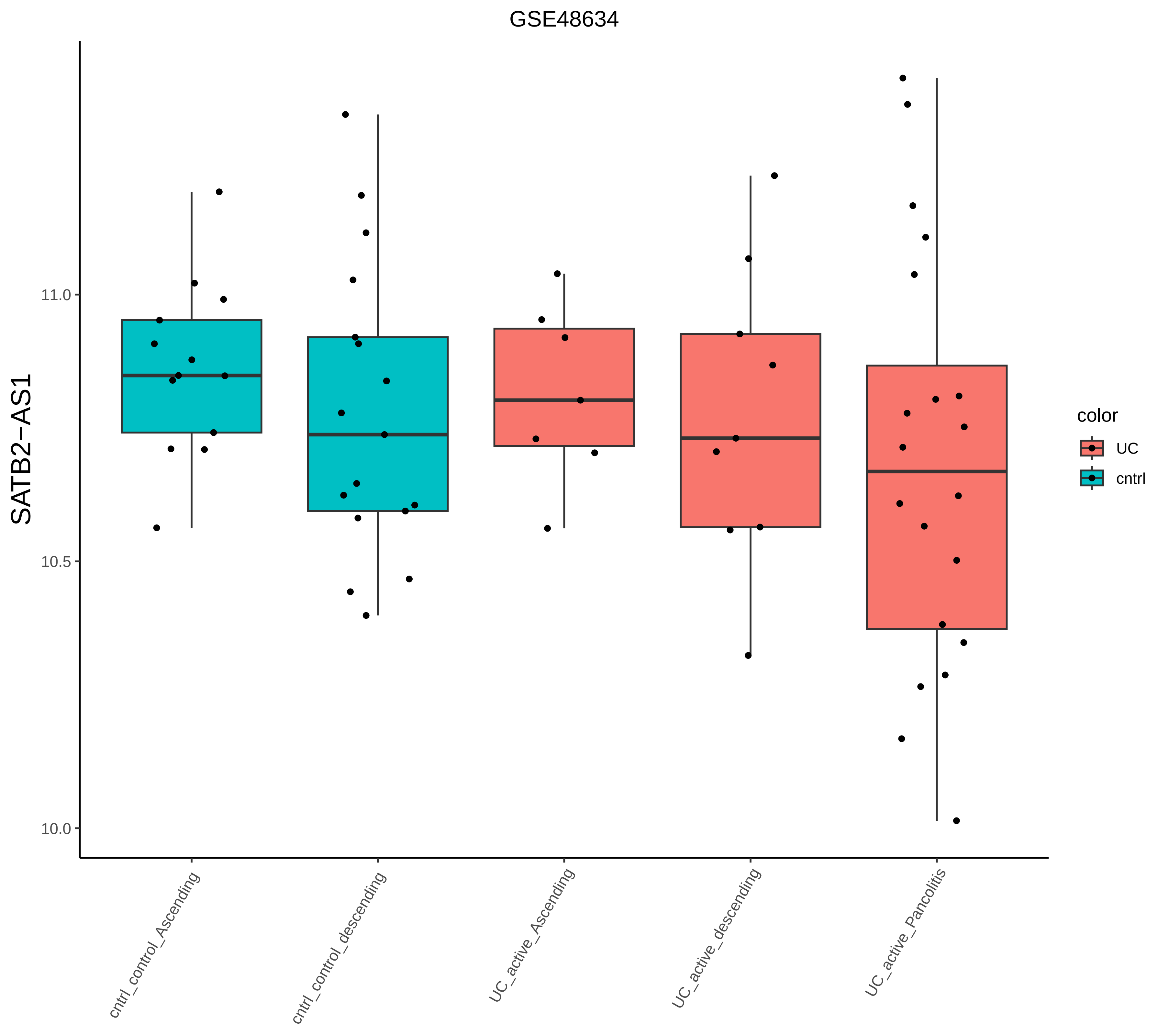

GSE48634
SATB2−AS1
11.0
10.5
10.0
cntrl_control_Ascending
cntrl_control_descending
UC_active_Ascending
UC_active_descending
UC_active_Pancolitis
color
UC
cntrl

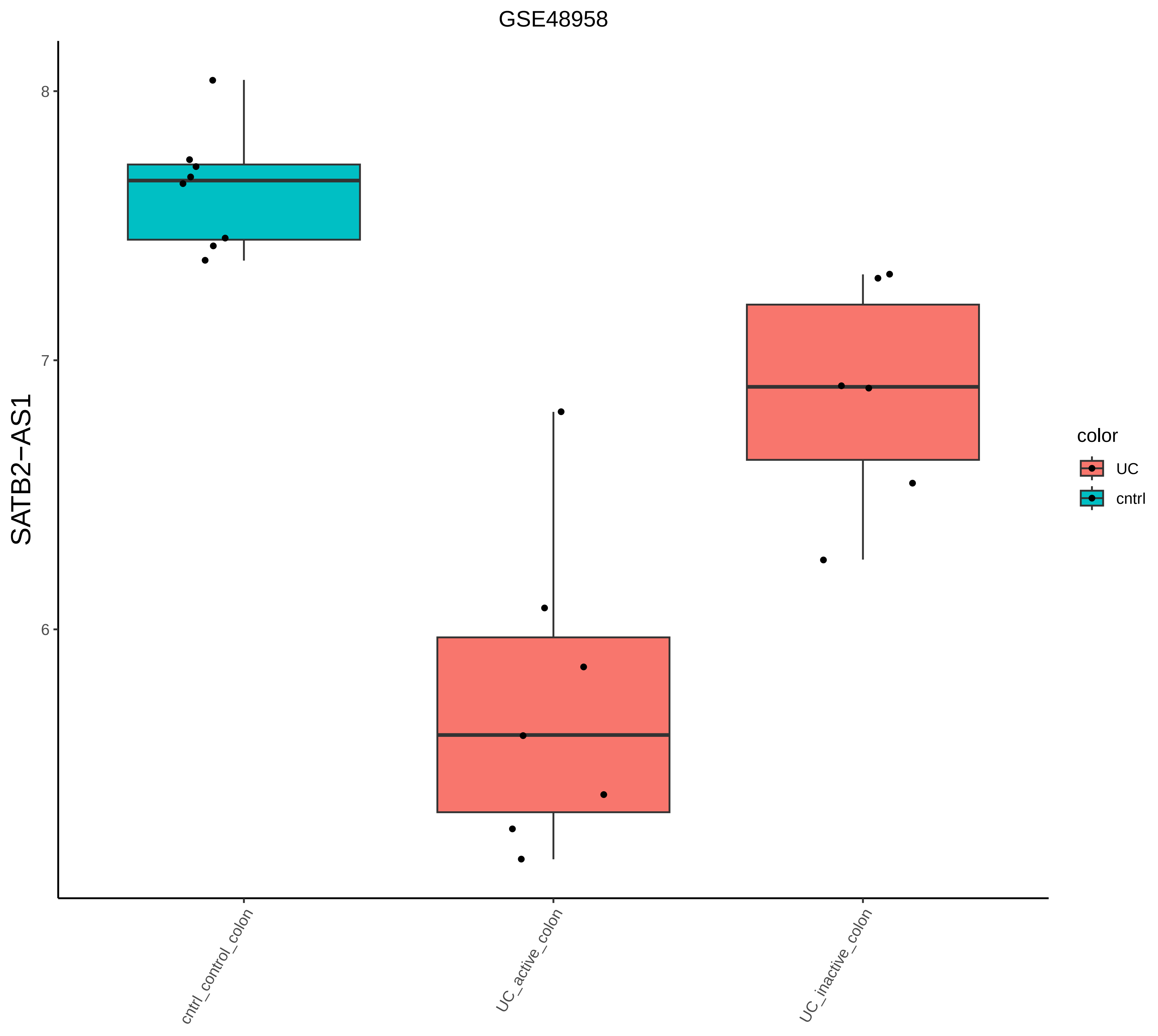
GSE48958
SATB2−AS1
8
7
6
cntrl_control_colon
UC_active_colon
UC_inactive_colon
color
UC
cntrl

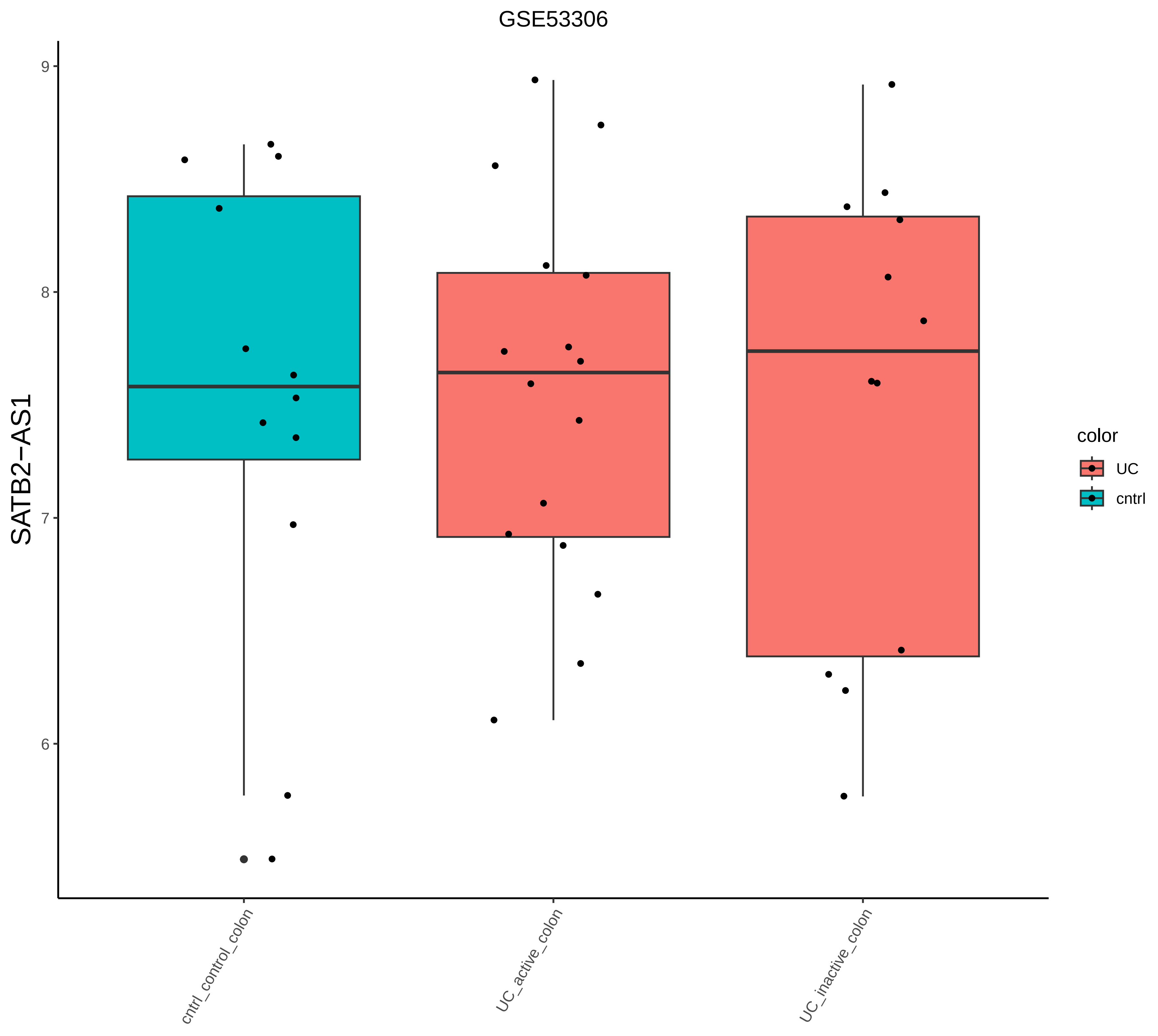
GSE53306
SATB2−AS1
9
8
7
6
cntrl_control_colon
UC_active_colon
UC_inactive_colon
color
UC
cntrl

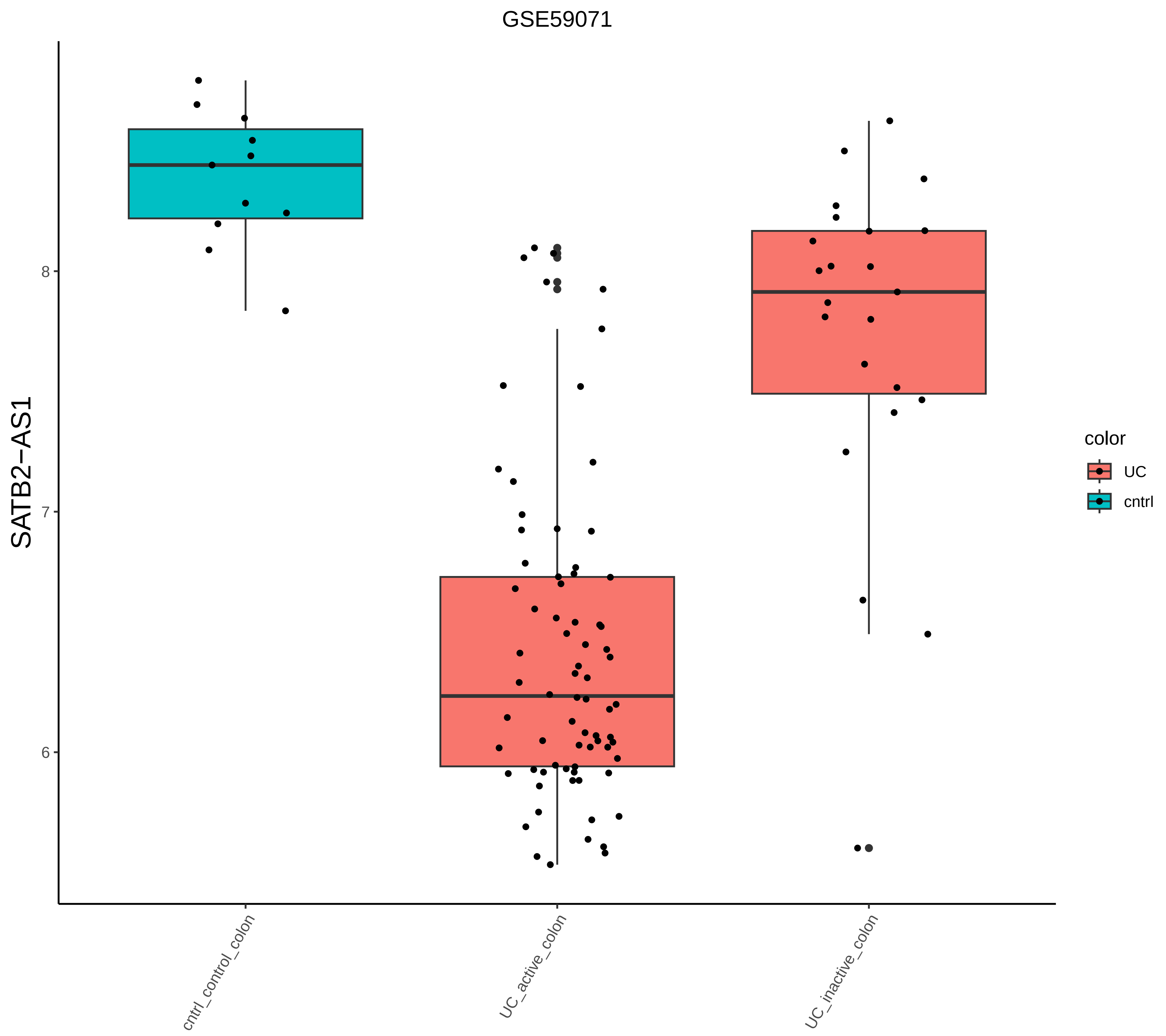

GSE59071
SATB2−AS1
8
7
6
cntrl_control_colon
UC_active_colon
UC_inactive_colon
color
UC
cntrl

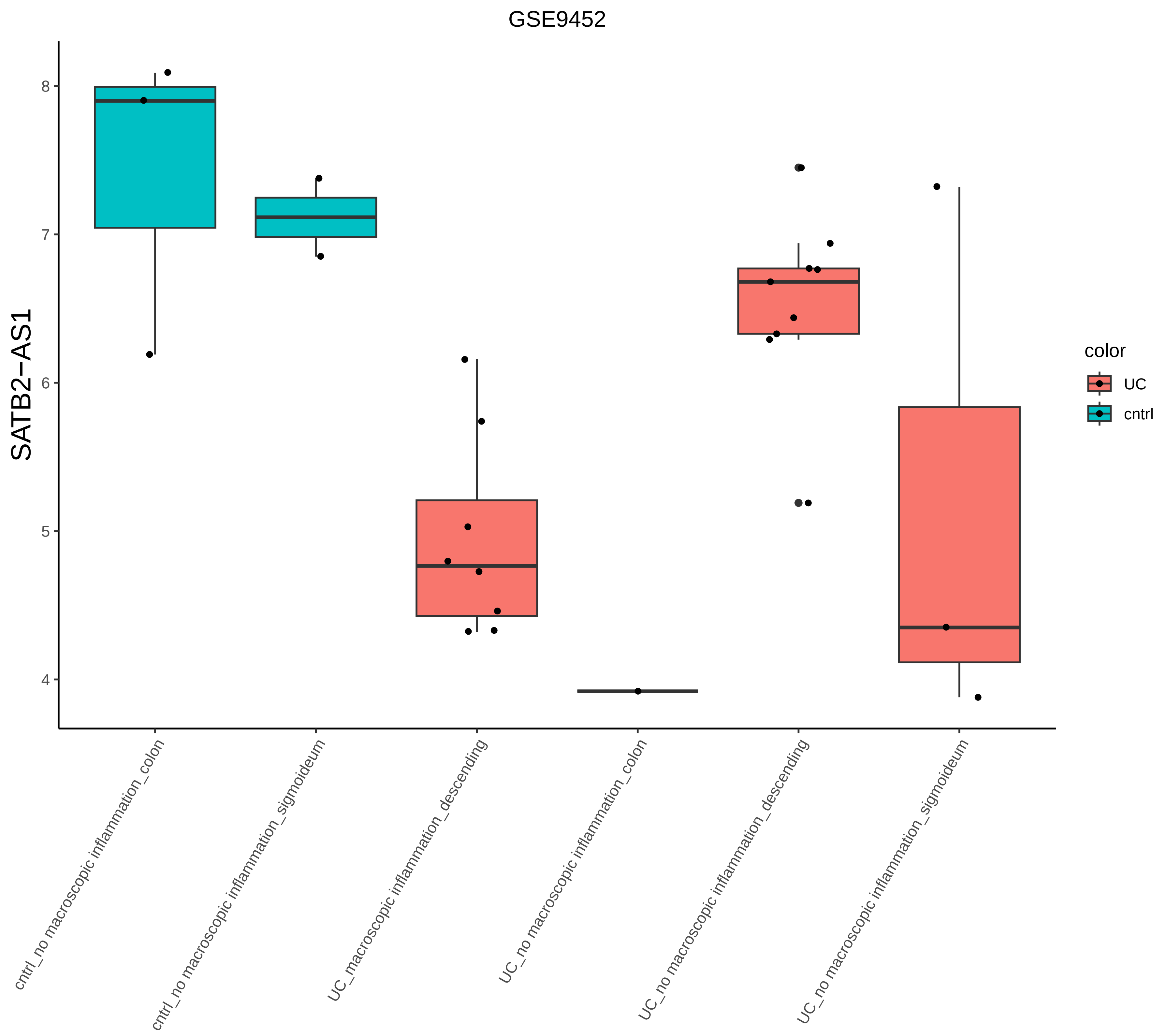

GSE9452
SATB2−AS1
8
7
6
5
4
color
UC
cntrl
cntrl_no macroscopic inflammation_colon
cntrl_no macroscopic inflammation_sigmoideum
UC_macroscopic inflammation_descending
UC_no macroscopic inflammation_colon
UC_no macroscopic inflammation_descending
UC_no macroscopic inflammation_sigmoideum

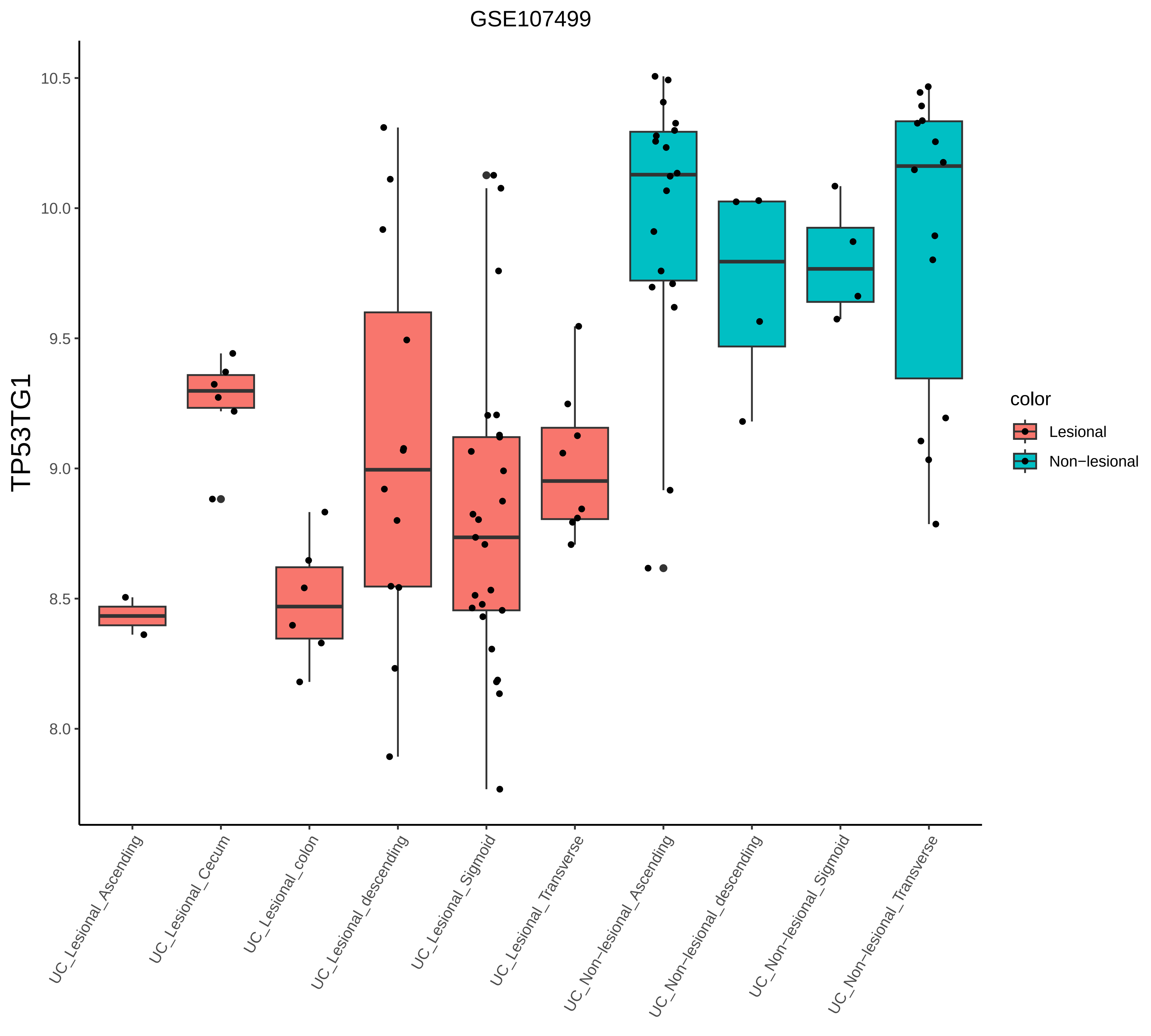

GSE107499
TP53TG1
10.5
10.0
9.5
9.0
8.5
8.0
UC_Lesional_Ascending
UC_Lesional_Cecum
UC_Lesional_colon
UC_Lesional_descending
UC_Lesional_Sigmoid
UC_Lesional_Transverse
UC_Non−lesional_Ascending
UC_Non−lesional_descending
UC_Non−lesional_Sigmoid
UC_Non−lesional_Transverse
color
Lesional
Non−lesional

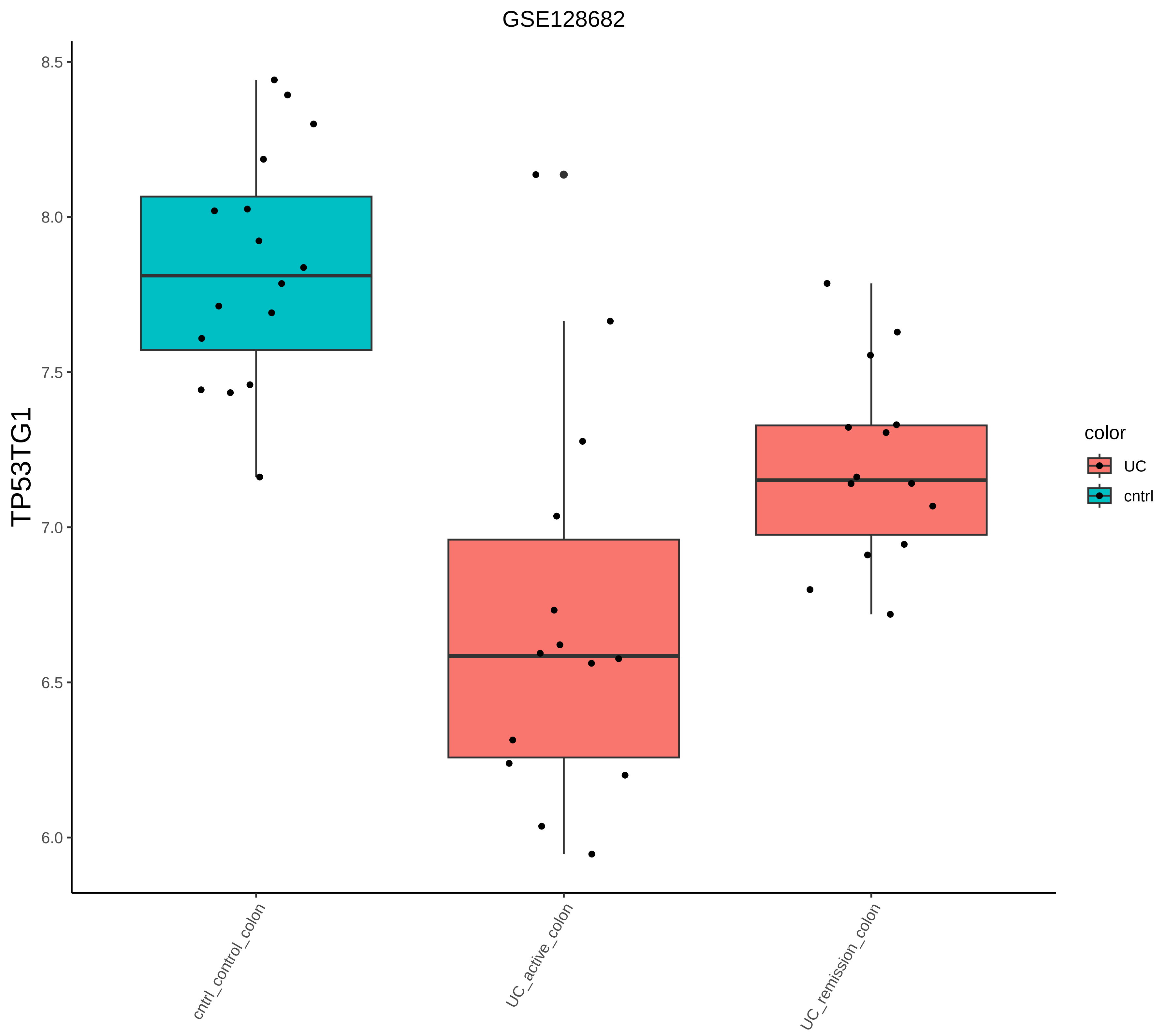
GSE128682
TP53TG1
8.5
8.0
7.5
7.0
6.5
6.0
cntrl_control_colon
UC_active_colon
UC_remission_colon
color
UC
cntrl

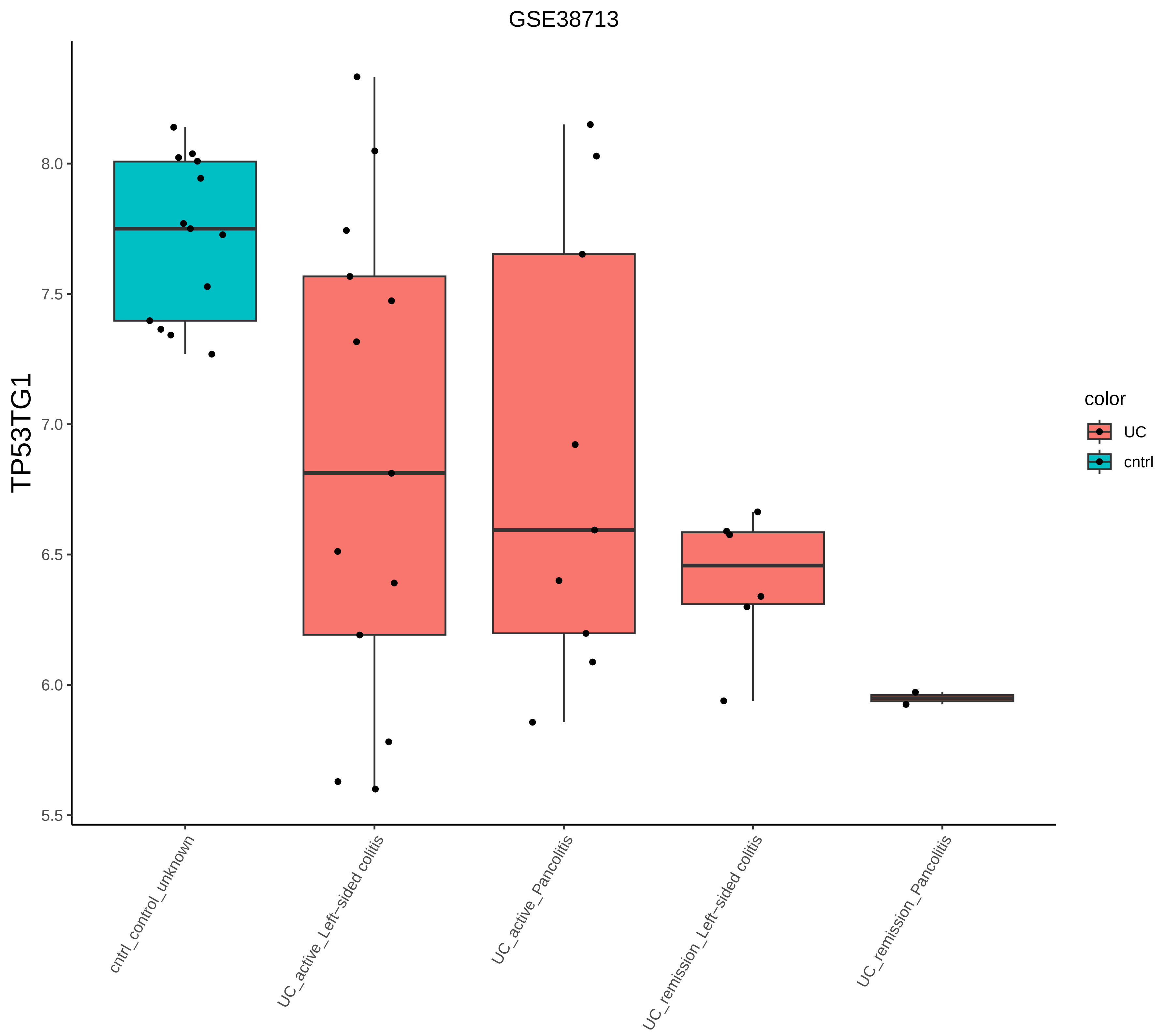

GSE38713
TP53TG1
8.0
7.5
7.0
6.5
6.0
5.5
cntrl_control_unknown
UC_active_Left−sided colitis
UC_active_Pancolitis
UC_remission_Left−sided colitis
UC_remission_Pancolitis
color
UC
cntrl

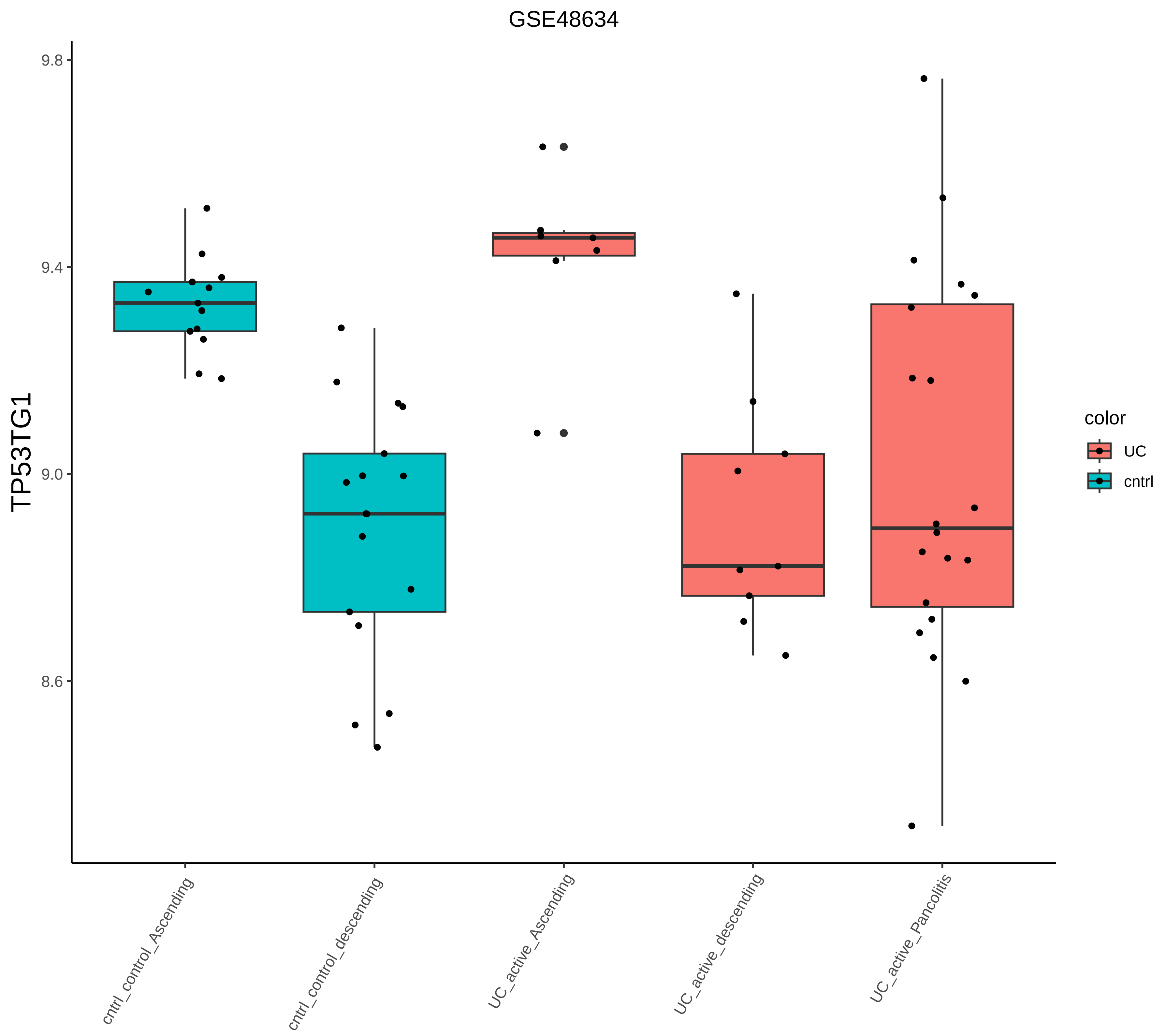

GSE48634
TP53TG1
9.8
9.4
9.0
8.6
cntrl_control_Ascending
cntrl_control_descending
UC_active_Ascending
UC_active_descending
UC_active_Pancolitis
color
UC
cntrl

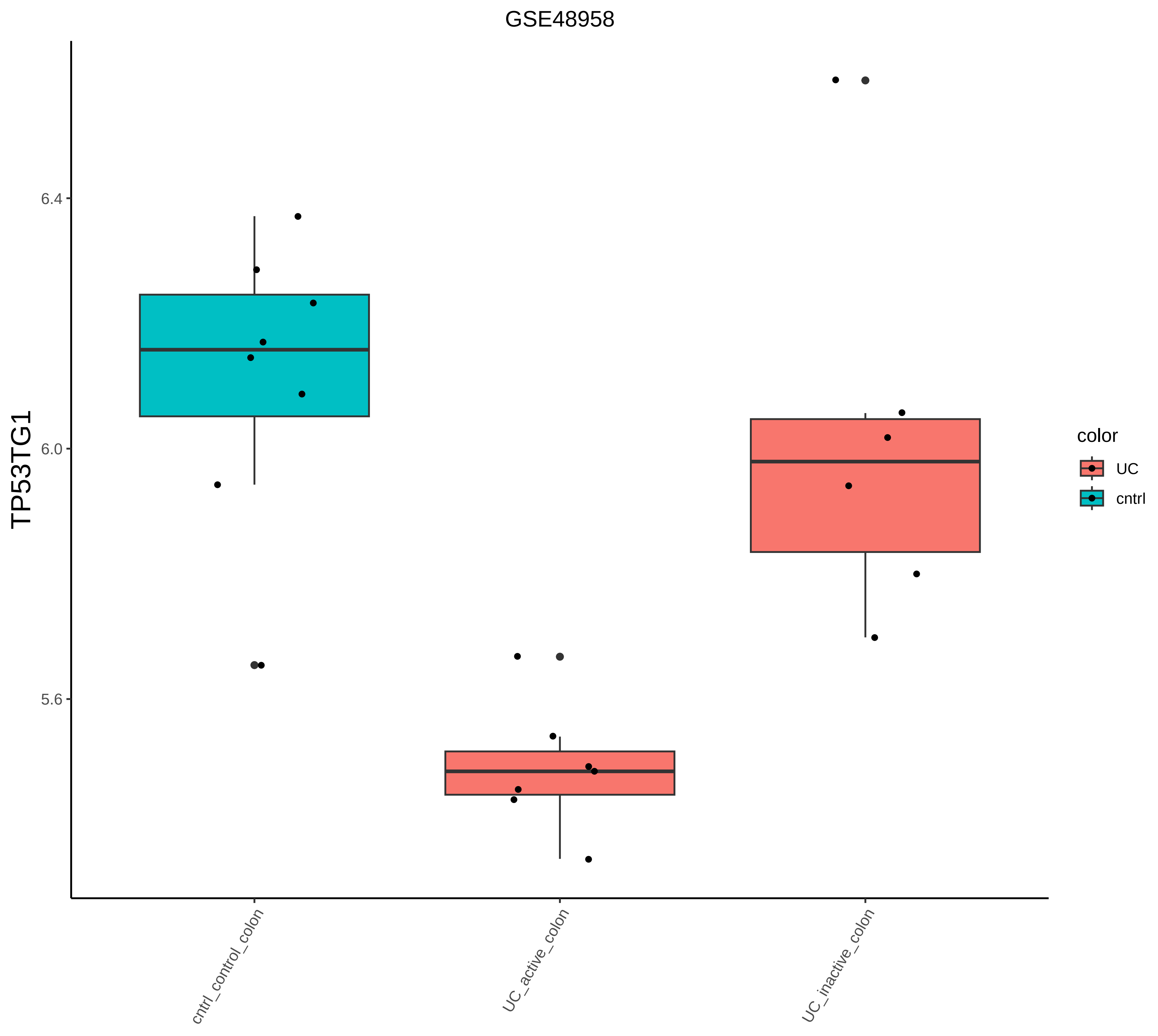

GSE48958
TP53TG1
6.4
6.0
5.6
cntrl_control_colon
UC_active_colon
UC_inactive_colon
color
UC
cntrl

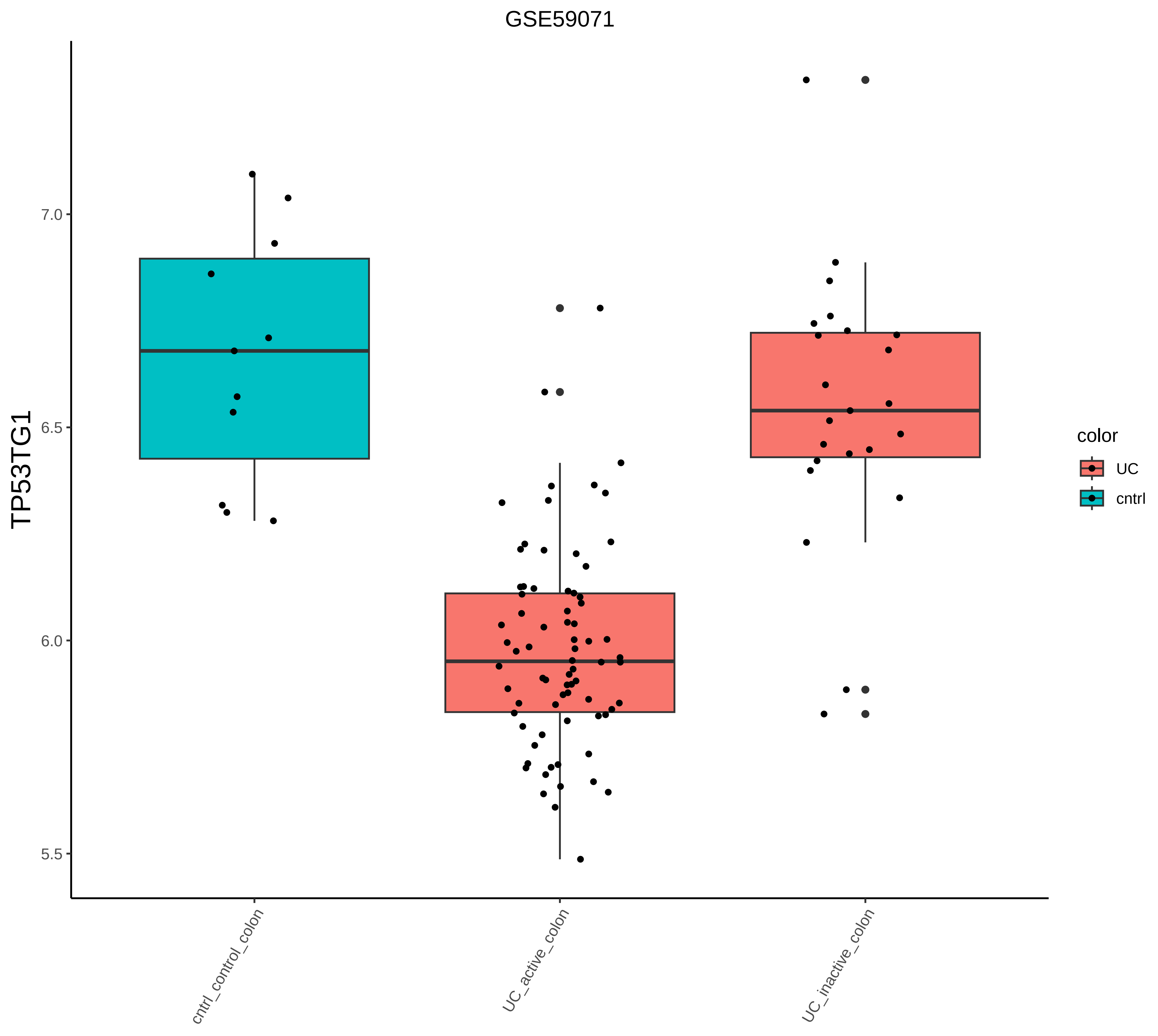

GSE59071
TP53TG1
7.0
6.5
6.0
5.5
cntrl_control_colon
UC_active_colon
UC_inactive_colon
color
UC
cntrl

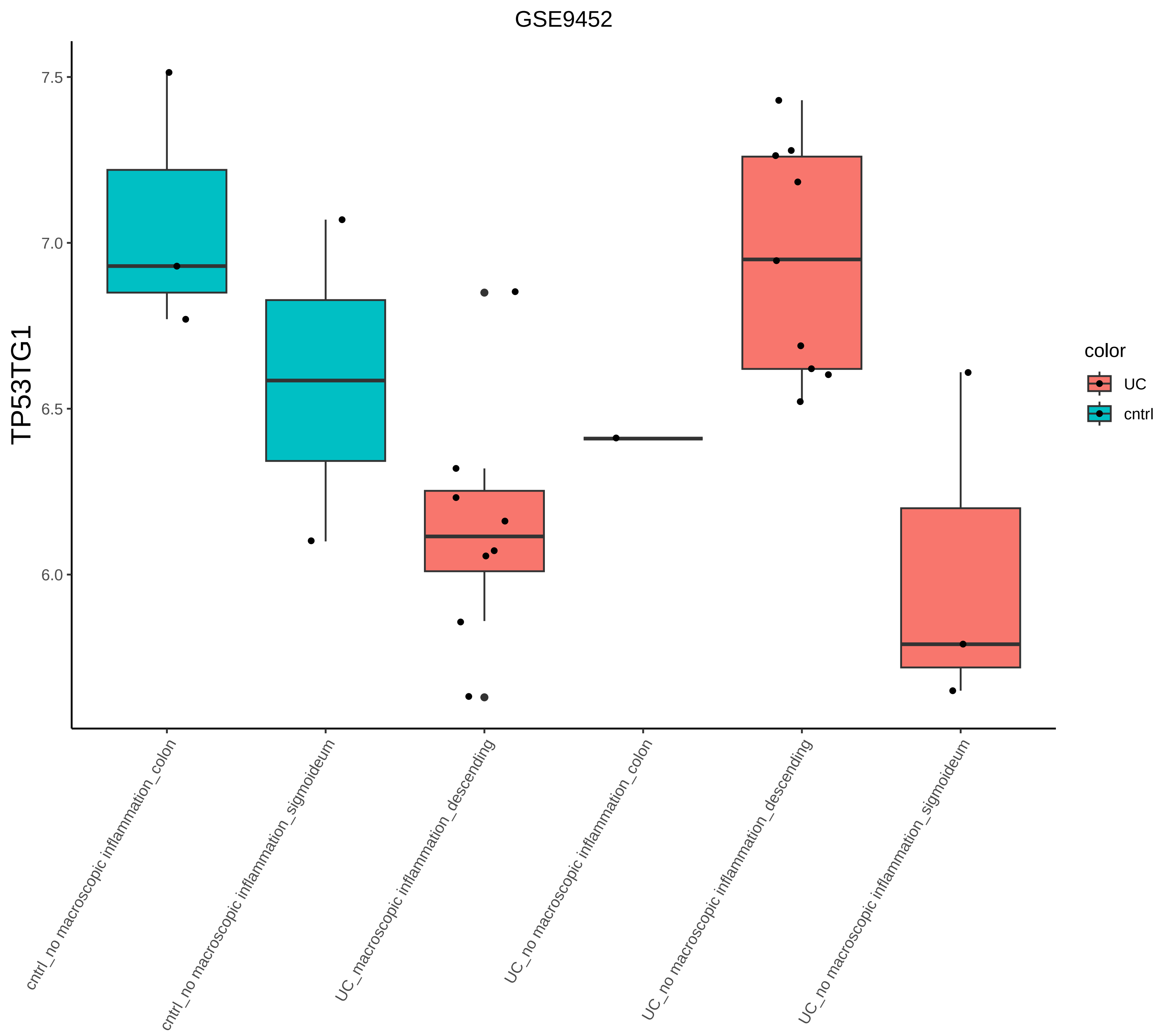

GSE9452
TP53TG1
7.5
7.0
6.5
6.0
color
UC
cntrl
cntrl_no macroscopic inflammation_colon
cntrl_no macroscopic inflammation_sigmoideum
UC_macroscopic inflammation_descending
UC_no macroscopic inflammation_colon
UC_no macroscopic inflammation_descending
UC_no macroscopic inflammation_sigmoideum

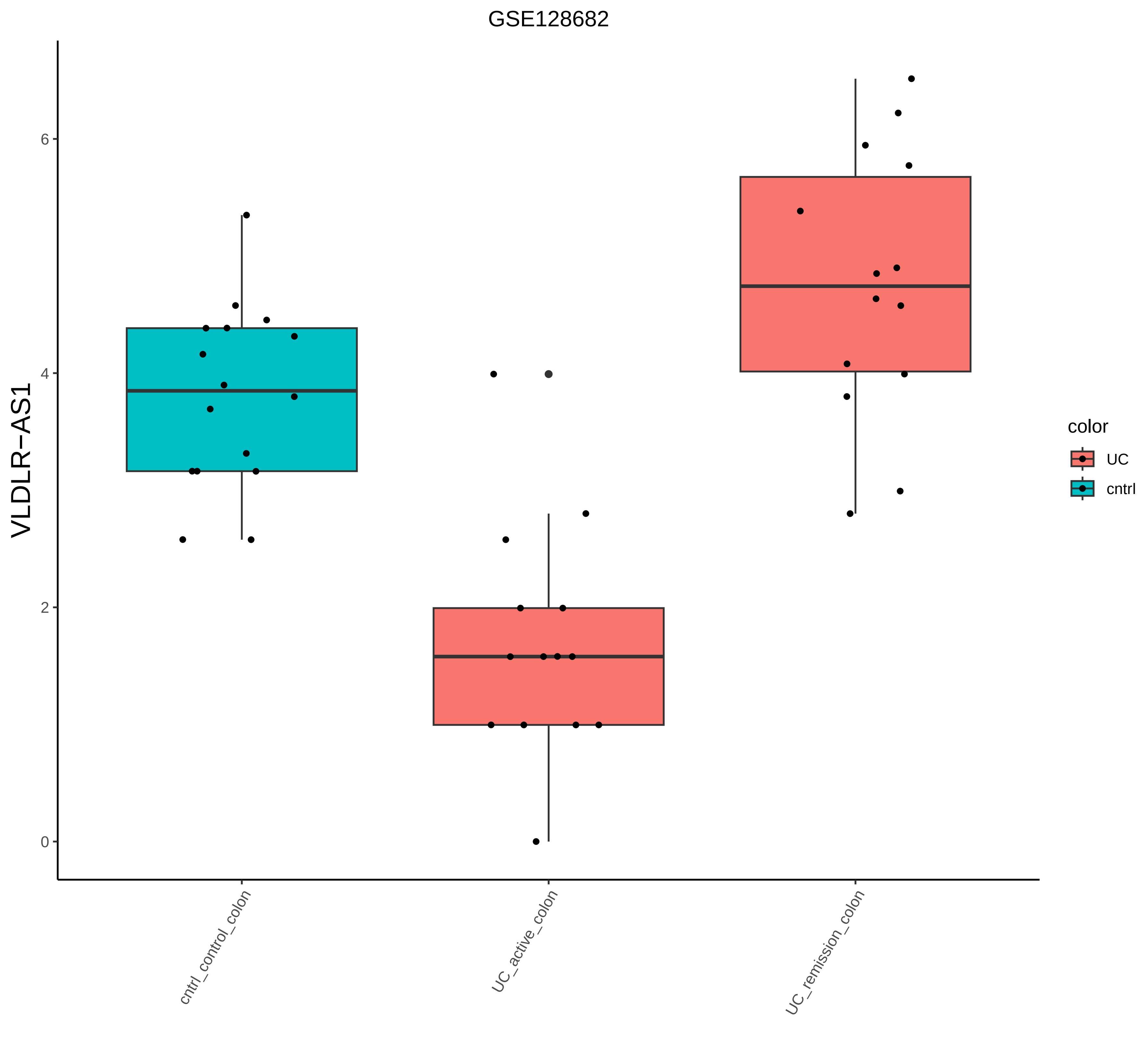

GSE128682
VLDLR−AS1
0
2
4
6
cntrl_control_colon
UC_active_colon
UC_remission_colon
color
UC
cntrl

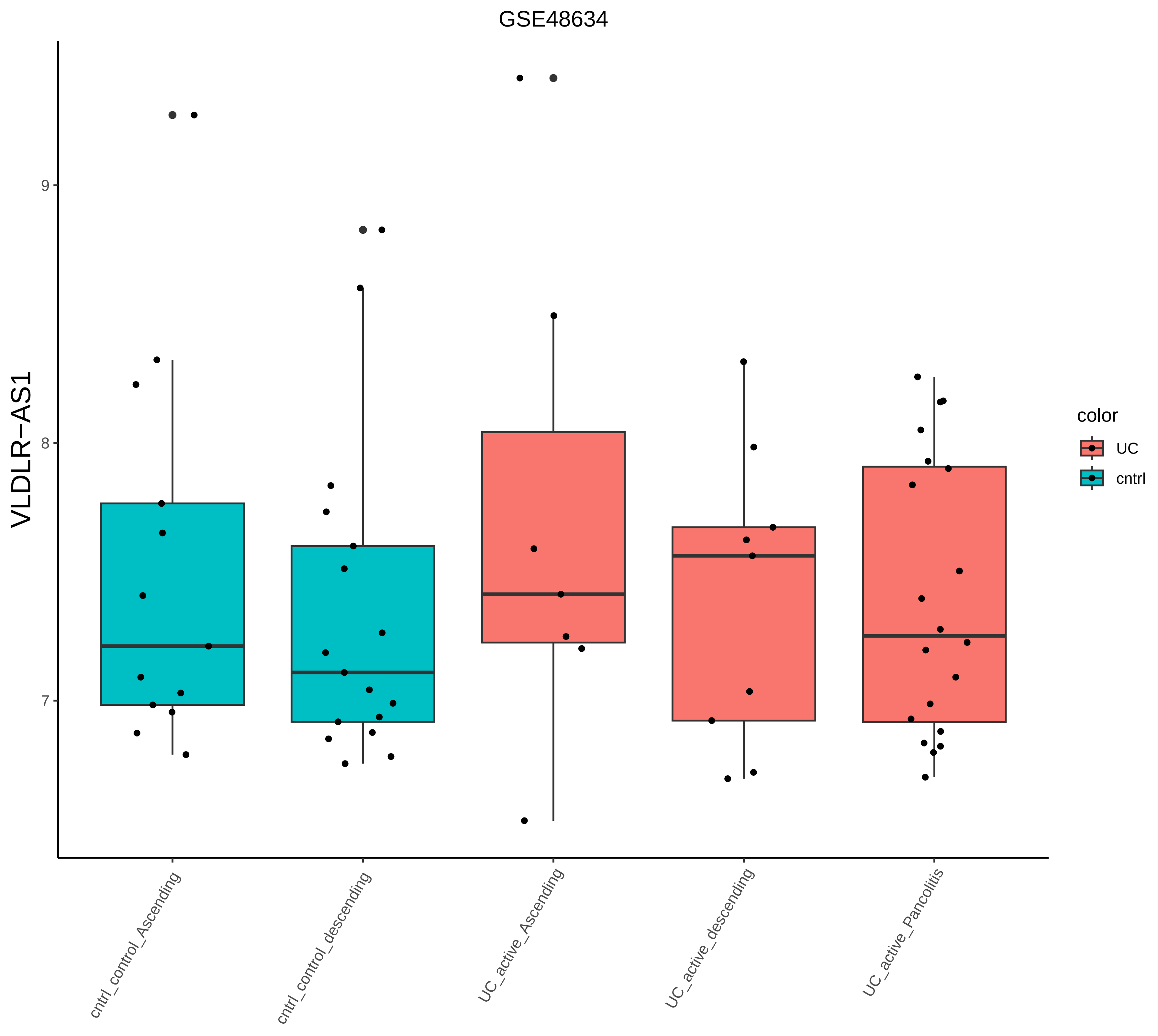

GSE48634
VLDLR−AS1
9
8
7
cntrl_control_Ascending
cntrl_control_descending
UC_active_Ascending
UC_active_descending
UC_active_Pancolitis
color
UC
cntrl

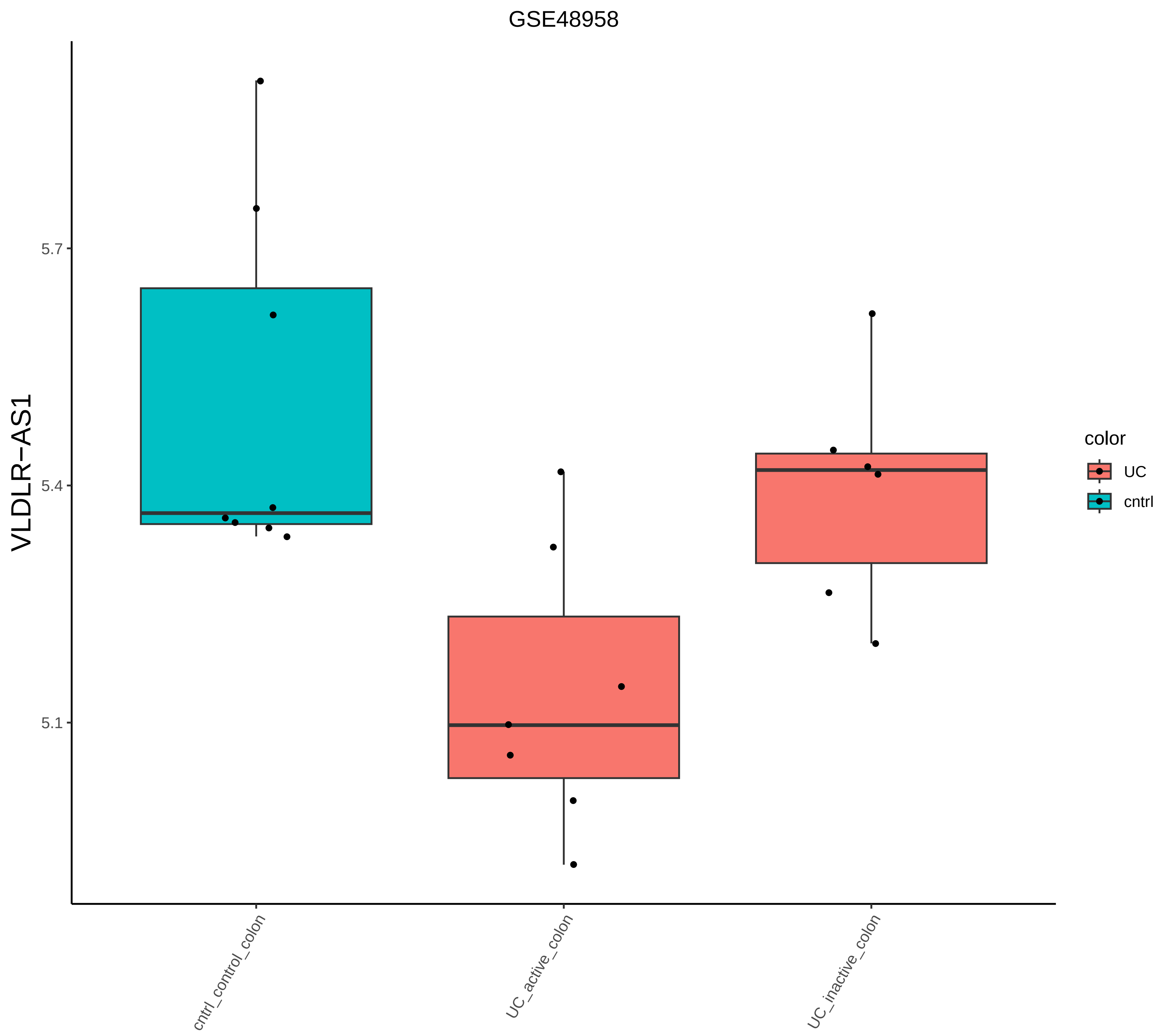
GSE48958
VLDLR−AS1
5.7
5.4
5.1
cntrl_control_colon
UC_active_colon
UC_inactive_colon
color
UC
cntrl

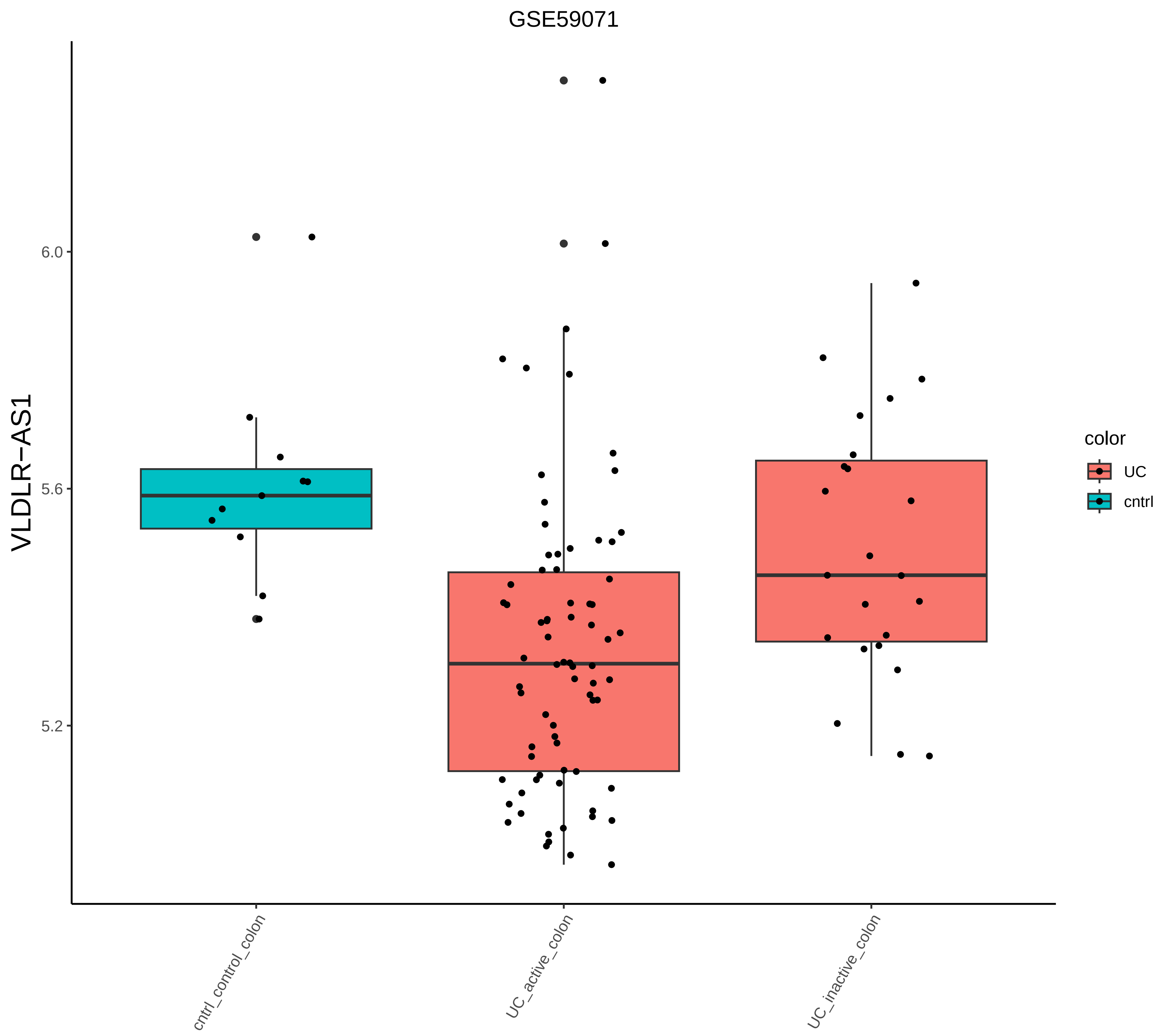

GSE59071
VLDLR−AS1
6.0
5.6
5.2
cntrl_control_colon
UC_active_colon
UC_inactive_colon
color
UC
cntrl

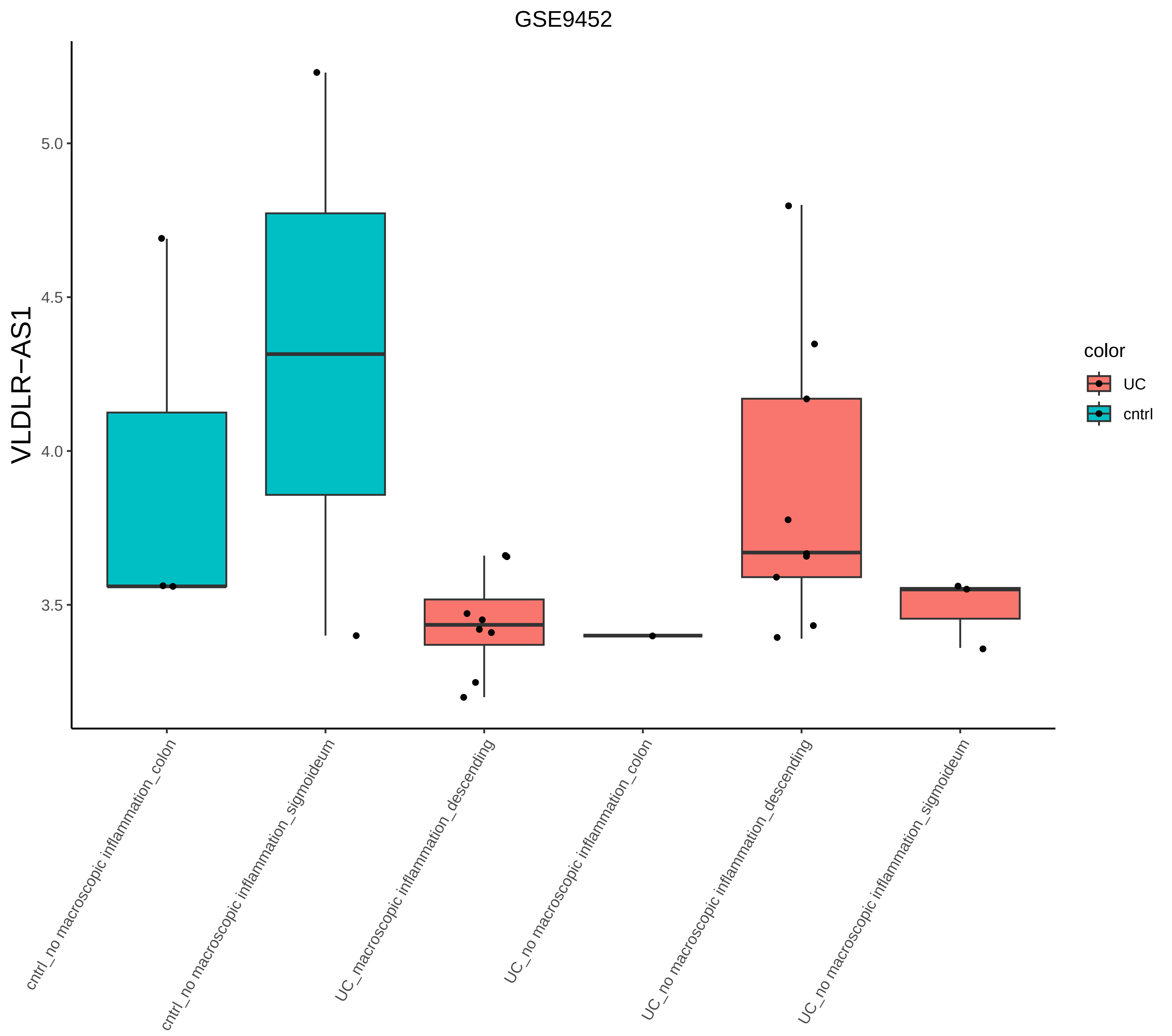
GSE9452
VLDLR−AS1
5.0
4.5
4.0
3.5
cntrl_no macroscopic inflammation_colon
cntrl_no macroscopic inflammation_sigmoideum
UC_macroscopic inflammation_descending
UC_no macroscopic inflammation_colon
UC_no macroscopic inflammation_descending
UC_no macroscopic inflammation_sigmoideum
color
UC
cntrl
