# Supplementary material for: Challenges in Defining a Reference Set of Differentially Expressed lncRNAs in Ulcerative Colitis by Meta-Analysis
Source: Curr Issues Mol Biol. 2024 Apr 5;46(4):3164–74. doi: 10.3390/cimb46040198 (PMC11049510; doi:10.3390/cimb46040198)
Supplement: Supplementary file 1 [file cimb-46-00198-s001.zip › Supplementary files/Table S1.pdf]

GEO series Accession Number	Description	Reference
GSE109142	Dataset comprises a total of 226 samples. Among these, 105 samples correspond to pre-response, 101 pre-resistant, and 20non-IBD controls. Pre-response and pre-resistant were considered as active UC. Data contains mild, moderate and sever UC based on PUCAI score and Mayo endoscopy sub score. The disease location for data was proctosigmoiditis (14), left-sided colitis (44), and extensive/pancolitis/un-assessable (167).	[1]
GSE128682	Dataset comprises a total of 44 samples including 14 treatment naïve UC patients, 14 UC remission patients, and 16 non-IBD controls. The 14 treatment naïve UC samples were considered as active UC and control samples as non-IBD controls. Remission samples were removed from the study. Data contains mild to moderate UC samples based on UC disease activity index (UCDAI) endoscopy sub score. Biopsies were taken from the sigmoid part of the colon.	[2]
GSE206285	Dataset comprises a total of 568 samples. Among these, 550 samples correspond to patients diagnosed with UC, while the remaining 18 samples represent non- IBD controls. The 550 UC sample considered as active UC.	[3]
GSE87466	Dataset comprises a total of 108 samples. Among these, 87 samples correspond to patients diagnosed with UC, while the remaining 21 samples represent non-IBD controls. Disease activity in the UC patients was assessed using the Mayo score. Intestinal biopsies of distal colon from adult patients with extensive (n = 27) or limited (n = 60) UC. The 87 UC sample considered as active UC.	[4]
GSE92415	Dataset comprises a total of 183 samples. Among these, 162 samples correspond to patients diagnosed with moderate to severe Ulcerative Colitis (UC), while the remaining 21 samples represent non- IBD controls. Disease activity in the UC patients was assessed using the Mayo score and Endoscopy sub score. The 162 UC sample considered as active UC.	[5]
GSE107499	Dataset comprises a total of 119 samples including 75 lesional and 45 non-lesional, we define lesional sample as an active UC since lesional sample were biopsy tissues taken from inflamed area (according to GEO).	**NA**
GSE47908	Dataset comprises a total of 60 samples. Among these, 45 samples correspond to patients with UC, while the remaining 15 samples represent non- IBD controls. Biopsies sample ware collected from descending colon. Disease activity was assessed using the Mayo score and Mayo endoscopic score. The 45 samples were considered as active UC	[6]
GSE16879	Dataset comprises a total of 133 samples including 73 CD, 12 controls and 48 UC. UC patients including post-resistant (33), post-response (27), pre-resistant (24), and pre-response (37) and 12 control contains 6 sample from Ileum and 6 from colon. Samples from CD, post-Resistant, post-Response, and control from Ileum were removed. Samples from Pre-Resistant and pre-Response were considered as active UC.	[7]
GSE59071	Dataset comprises a total of 116 samples. Among these, 97 samples correspond to patients with active UC (74) and inactive (23), while the remaining 11 samples represent non- IBD controls. Disease activity in the UC patients was assessed using the Mayo score and Endoscopy sub score. Biopsies ware taken from most affected site of the colon (sigmoid or rectum). The disease location for data was left-sided colitis and pancolitis.	[8]

References

1. Haberman, Y.; Karns, R.; Dexheimer, P.J.; Schirmer, M.; Somekh, J.; Jurickova, I.; Braun, T.; Novak, E.; Bauman, L.; Collins, M.H. Ulcerative Colitis Mucosal Transcriptomes Reveal Mitochondriopathy and Personalized Mechanisms Underlying Disease Severity and Treatment Response. *Nat. Commun.* **2019**, *10*, 38.

2. Fenton, C.G.; Taman, H.; Florholmen, J.; Sørbye, S.W.; Paulssen, R.H. Transcriptional Signatures That Define Ulcerative Colitis in Remission. *Inflamm. Bowel Dis.* **2021**, *27*, 94–105.

3. Pavlidis, P.; Tsakmaki, A.; Pantazi, E.; Li, K.; Cozzetto, D.; Digby-Bell, J.; Yang, F.; Lo, J.W.; Alberts, E.; Sa, A.C.C. Interleukin-22 Regulates Neutrophil Recruitment in Ulcerative Colitis and Is Associated with Resistance to Ustekinumab Therapy. *Nat. Commun.* **2022**, *13*, 5820.

4. Li, K.; Strauss, R.; Ouahed, J.; Chan, D.; Telesco, S.E.; Shouval, D.S.; Canavan, J.B.; Brodmerkel, C.; Snapper, S.B.; Friedman, J.R. Molecular Comparison of Adult and Pediatric Ulcerative Colitis Indicates Broad Similarity of Molecular Pathways in Disease Tissue. *J. Pediatr. Gastroenterol. Nutr.* **2018**, *67*, 45–52.

5. Sandborn, W.J.; Feagan, B.G.; Marano, C.; Zhang, H.; Strauss, R.; Johanns, J.; Adedokun, O.J.; Guzzo, C.; Colombel, J.-F.; Reinisch, W. Subcutaneous Golimumab Induces Clinical Response and Remission in Patients with Moderate-to-Severe Ulcerative Colitis. *Gastroenterology* **2014**, *146*, 85–95.

6. Bjerrum, J.T.; Nielsen, O.H.; Riis, L.B.; Pittet, V.; Mueller, C.; Rogler, G.; Olsen, J. Transcriptional Analysis of Left-Sided Colitis, Pancolitis, and Ulcerative Colitis-Associated Dysplasia. *Inflamm. Bowel Dis.* **2014**, *20*, 2340–2352.

7. Arijs, I.; De Hertogh, G.; Lemaire, K.; Quintens, R.; Van Lommel, L.; Van Steen, K.; Leemans, P.; Cleynen, I.; Van Assche, G.; Vermeire, S. Mucosal Gene Expression of Antimicrobial Peptides in Inflammatory Bowel Disease before and after First Infliximab Treatment. *PLoS One* **2009**, *4*, e7984.

8. Vanhove, W.; Peeters, P.M.; Staelens, D.; Schraenen, A.; Van der Goten, J.; Cleynen, I.; De Schepper, S.; Van Lommel, L.; Reynaert, N.L.; Schuit, F. Strong Upregulation of AIM2 and IFI16 Inflammasomes in the Mucosa of Patients with Active Inflammatory Bowel Disease. *Inflamm. Bowel Dis.* **2015**, *21*, 2673–2682.
